# Spectral evidence for irradiated halite on Mars

**DOI:** 10.1038/s41598-024-55979-6

**Published:** 2024-03-06

**Authors:** Michael S. Bramble, Kevin P. Hand

**Affiliations:** Jet Propulsion Laboratory, California Institute of Technology, 4800 Oak Grove Drive, Pasadena, CA 91109 USA

**Keywords:** Planetary science, Geochemistry, Mineralogy, Astronomy and planetary science, Planetary science, Astrobiology, Inner planets

## Abstract

The proposed chloride salt-bearing deposits on Mars have an enigmatic composition due to the absence of distinct spectral absorptions for the unique mineral at all wavelengths investigated. We report on analyses of remote visible-wavelength spectroscopic observations that exhibit properties indicative of the mineral halite (NaCl) when irradiated. Visible spectra of halite are generally featureless, but when irradiated by high-energy particles they develop readily-identifiable spectral alterations in the form of color centers. Consistent spectral characteristics observed in the reflectance data of the chloride salt-bearing deposits support the presence of radiation-formed color centers of halite on the surface of Mars. We observe a seasonal cycle of color center formation with higher irradiated halite values during winter months, with the colder temperatures interpreted as increasing the formation efficiency and stability. Irradiated halite identified on the surface of Mars suggests that the visible surface is being irradiated to the degree that defects are forming in alkali halide crystal structures.

Putative chloride salt-bearing deposits have been identified on the surface of Mars in orbital data (Fig. 1) on the basis of an absence of spectral absorption features and the presence of distinct spectral slopes at infrared wavelengths^1,2^. These proposed chloride deposits were characterized by a distinct thermal infrared spectrum with reduced spectral contrast near 1000 cm^-1^ leading to a featureless emissivity spectrum that slopes towards lower wavenumbers^2^. Visible to near-infrared (VNIR) reflectance spectra of the chloride deposits revealed spectral signatures consistent with, but not unique to, chlorides^3,4^. VNIR data ruled out sulfides^3^ and oxychlorine species or hydrated chloride species^5^, and ratioed spectra produced a lack of VNIR spectral features^2^. The combination of spectral characteristics, or lack thereof, were argued to necessitate something other than the common minerals identified on Mars^2^.

Laboratory spectroscopic investigations^6,7^ and geochemical modeling appear to favor halite (NaCl) as the akali halide “chloride” for the chloride salt-bearing deposits. Thermal infrared analyses suggest a halite abundance of $\sim$ 10 to 25% via laboratory and model studies of two-component mixtures with martian regolith^7^, and the near-infrared spectra of the deposits can be explained by mixtures of anhydrous chloride salts and silicates^3,6^. However, direct evidence of halite has not been identified with Mars orbital spectroscopy as halite is spectrally featureless at VNIR wavelengths^8^. Despite the rigor of the cited work, the primary mineral producing the observed unique spectral characteristics has remained enigmatic.

However, when irradiated by energetic particles, alkali halides (including halite) develop readily identifiable absorption features by trapping electrons in halogen vacancies^9,10^. These color centers aid in remote compositional analysis as they provide distinct spectral features for mineral species that are otherwise spectrally inactive^11,12^. Key radiation-formed color centers for halite include a 0.46 $\upmu$m F center and 0.72 $\upmu$m M center (Fig. 2a)^9,10^. On the surface of Mars, galactic cosmic rays, solar energetic particles, and possibly energetic solar ultraviolet photons could create the discoloration and spectral features known to arise in alkali halides^13–15^. Also, the source of radiation need not be external to Mars, as electrostatic discharge (ESD) generated by martian dust activities may form color centers in halite^16^.

The orbital images of chloride salt-bearing deposits exhibit a range of geologic contexts. These include occurrences in paleolake basins and in the vicinity of valley network terrains^2^, as well as a correlation with inverted channels and terminations at fan deposits^17^. The deposits commonly appear in local topographic lows^2,18^, and their morphological characteristics suggest that they drape the local topography^5^. Polygonal fracturing is observed at the deposits and has been interpreted as desiccation fractures or salt-related polygons^1,4^. The spectroscopic signatures suggest the deposits are well indurated but friable^7^. The majority of observations remain consistent with one of the original formation hypotheses: that these features formed via the ponding and evaporation of surface runoff or discharged groundwater^2^. Other formation hypotheses include volcanic sources, playa environments, hydrothermal brines, deep water lakes^19^, or an icy top-down melting process paired with concentration via seasonal sublimation and dehydration^20^.

In this contribution, we investigate the visible and near-infrared wavelength characteristics of the chloride deposits using a suite of images collected by the Compact Reconnaissance Imaging Spectrometer for Mars (CRISM). We apply spectroscopic data analysis methods to the images to characterize the spectral properties of the chloride deposits and the possibility of halite contributing to the spectral continuum. We then investigate these data to see if the spectral variation of the chloride deposits could be explained by the presence of radiation-formed color centers in halite. The central analysis of our work is the spectral modeling of these images using two different techniques. The first is factor analysis and target transformation and the second is linear least-squares spectral unmixing. These modeling efforts are focused on identifying the presence of irradiated halite and, if so, its spatial distribution and spectral fraction. We then investigate the temporal dimension of our observation suite to investigate whether seasonal phenomena may be impacting the presence and persistence of radiation color centers.

The composition of these deposits is important to identify, as knowing the salt composition will allow for the investigation of the provenance of this mineralogy and its relationship with the aqueous composition and evolution of the fluids that formed the deposits. These deposits encrypt information about a watery past on Mars unlike any other environment explored on the surface to date, and they may represent the last significant surface-water-related deposits^5^. As such, if life did ever exist on the martian surface, these deposits may have been the last refuge for surface life on Mars.

## Results

### Visible spectral properties of the chloride deposits

Reflectance spectra of the chloride deposits commonly exhibit the highest reflectance values at all wavelengths in the visible range for our CRISM images. While the visible reflectance spectra of the chloride deposits exhibit the ubiquitous martian red-sloped spectrum resulting from ferric iron transitions in the 0.40–0.75 $\upmu$m range^21,22^, they have unique attributes. The long-wavelength negative slope towards 1 $\upmu$m is shallower or absent for the chloride deposits in comparison to other spectra pulled from a given image, as the chloride deposit reflectance trends towards a flat spectrum from $\sim$ 0.8 to 1.0 $\upmu$m (Fig. 2b). Furthermore, the short-wavelength component commonly has a steeper drop in reflectance towards shorter wavelengths than the rest of the materials in a CRISM scene (Fig. 2c). While not direct spectral evidence of irradiated halite, these spectral characteristics provide what may be indirect evidence for halite in these deposits. The greater the spectral fraction of irradiated NaCl in our modeling presented below, the greater the spectra exhibit these characteristics. These characteristics also match those observed with simple linear mixing in reflectance where irradiated halite was added in increasing abundance to CRISM spectra of non-chloride deposit terrains (Supplementary Figure S1).

Other minerals that exhibit broad spectral absorptions at visible wavelengths can be reviewed and excluded using the CRISM browse products and parameter images. For example, the BD530_2 parameter correlates with nanophase or crystalline ferric oxides^23^. While this parameter is always positive in our CRISM images, the highest values in the scene are anti-correlated with the chloride browse product, and the chloride deposits commonly have the lowest values within a CRISM image (Supplementary Data Set S1). This correlates with the spectral trend towards an absence of a 1 $\upmu$m absorption, which would also indicate the relative absence of iron-bearing minerals, and points towards a non-iron-bearing phase causing the short-wavelength absorption.

### Evidence for irradiated halite color centers in the chloride salt-bearing deposits

To investigate the presence of irradiated halite in the chloride salt-bearing deposits, we performed a factor analysis and target transformation analysis of our CRISM image suite (Fig. 3). These methods have previously been applied in mineralogical analyses of CRISM spectral data^24–27^, and they excel at isolating independently variable spectral components that are in complex spectral convolutions and whose spectral signatures are not present in the original signal due to the convolutions or low abundances. Our factor analysis probed 50 by 50 pixel frames that rastered across our entire CRISM data set, and the target transformation consisted of a least-squares fit to irradiated halite laboratory endmembers.

The factor analysis and target transformation analysis identified positive detections for irradiated halite and the locations of the positive detections spatially correlated with those identified as the chloride salt-bearing deposits via the CRISM browse products^23^ (Fig. 4a,b). Plotting the positive detections reveal an absorption with a minima near 0.45 $\upmu$m that matches the shape and position of the F center of irradiated halite (Fig. 4c). This analysis also revealed broad slopes downward from higher wavelengths towards a minimum near 0.72 $\upmu$m that are paired with the absorption near 0.45 $\upmu$m. The combination of these two absorption features may be indicative of irradiated halite with both F and M centers (Fig. 4d). The factor analysis and target transformation detections for the entire CRISM image suite investigated are listed in Supplementary Table S1.

To investigate the possibility of irradiated halite detections at greater spatial resolution, we applied a pixel-scale spectral unmixing model to each CRISM image. Our spectral modeling identifies irradiated halite as a mineral contributing to the spectral variation at the chloride salt-bearing deposits. Approximately half the 85 images investigated had spectral fractions of irradiated NaCl $\ge$ 10% (Fig. 5, Supplementary Table S1, Data Set S1). The maximum value modeled for the chloride deposits using the whole data set was 30%, and mean values are in the 5% range. These irradiated halite detections are contained within previously identified chloride deposits when compared to a global data base^2^ and using the CRISM chloride browse products for confirmation at higher spatial resolution. The modeled values for irradiated halite align with those predicted for the abundance of the mineral phase contributing the unique spectral characteristics to the chloride salt-bearing deposits as calculated using laboratory and remote sensing data^6,7^. Spectra calculated using the fractional components of the modeled endmembers exhibit the expected characteristics having the highest albedo at all wavelengths in a given scene, and stronger absorption at shorter wavelengths, as well as weaker absorption near 1 $\upmu$m (Fig. 2d).

Our 720/790 nm ratio images demonstrate that the spectral absorption, or relative reflectance drop at $\sim$ 0.72 $\upmu$m is spatially correlated with the chloride deposits (Fig. 5c). The ratio image spatially correlates in most cases with the CRISM browse products for the chloride deposits, though occasionally it also correlates with intense yellow colors when present in the chloride browse products (Supplementary Table S1). We report these findings here as they corroborate our other observations to suggest the presence of irradiated halite. However, given the CRISM calibration issues with the visible to near infrared detector due to a filter boundary and the overlapping wavelength location of this boundary and the M center, our confidence in the M center detections is lower in comparison to the spectral modeling results presented above.

When our factor analysis and target transformation results, the unmixing model results, and the observed visible-wavelength spectral characteristics are combined, interpreting the presence of irradiated halite to explain these phenomena becomes the most likely explanation. In summary, several independent lines of evidence support the presence of irradiated halite in the chloride salt-bearing deposits. They include the following: (1) the increased absorption at low wavelengths ($\sim$ 0.45 $\upmu$m), (2) the lack of $\sim$ 1 $\upmu$m iron absorption, and (3) the anti-correlation of the chloride deposits and the 0.53 $\upmu$m iron absorption. (4) The identification of broad absorption near 0.45 $\upmu$m (and 0.45 $\upmu$m absorptions paired with a second broad feature near 0.72 $\upmu$m) in the factor analysis and target transformation analysis. (5) The favoring of irradiated halite in our spectral unmixing results and at the expected fractional proportions. (6) The consistent spatial correlation of both the unmixing results and the factor analysis and target transformation results with the chloride deposits as identified by the CRISM chloride browse products. Lastly, (7) the tentative evidence for the presence of irradiated halite M centers observed both in the factor analysis and target transformation as well as the 720/790 nm reflectance ratio images.

### Temporal components may reveal a seasonal influence

The CRISM observations span over two Mars years, and thus we investigated whether there is a temporal component to our spectral modeling results, which could implicate a larger planetary phenomena. Plotting our modeled spectral fraction of irradiated halite as a function of martian sub-solar longitude ($L_{s}$) reveals that values are elevated for the first half of the martian year (Fig. 6a). Both the maximum and mean are elevated for $L_{s}$ of 0–180 relative to 180–360. Note that all of our images were collected in the southern hemisphere, as reflected by the mean central latitude of the data set being 27.3 ^∘^S with a standard deviation of 11.2^∘^ (Fig. 3). Therefore, all of our seasonal considerations assume a location in the southern hemisphere.

Plotting our modeling results as a function of calendar year does not present any additional trends apart from the seasonal influence (Supplementary Figure S2). The images we investigated spanned from 2007 to 2011. If the exposed halite-bearing surfaces were developing color centers as a result of electrically charged dust, perhaps one would expect modeled halite results to drop over time as Mars was engulfed in a global dust storm at the start of our data temporal range in 2007. We see no such trends in our data.

About a fifth of observations consist of repeat measurements where some portion of two CRISM footprints overlap. These observations follow the above seasonal trend (Supplementary Figure S3), where the images taken during southern spring or summer have lower irradiated halite spectral fractions and faded spectral fraction maps when compared to observations at lower solar longitudes. These observations suggest that a fundamental property related to the modeling of irradiated halite is changing at these locations throughout the year. We interpret this as changing intensity of the radiation-formed color centers growing when the surface is colder and annealing later when the surface is warmer. Other factors could also be contributing to this trend such as uncalibrated changes in atmospheric conditions or observation parameters such as temporal changes in solar incidence angle due to the Sun-synchronous orbit of the Mars Reconnaissance Orbiter. However, the consistency across observation sites suggests factors such as variations in atmospheric opacity or solar incidence angle are unlikely causes.

## Discussion

The presence of radiation-formed color centers on the surface of Mars would not be surprising as surface measurements portray a radiation environment elevated in high-energy particles^28–31^, in addition to the possibility of ESD color center formation^16^. As color centers can form in the presence of galactic cosmic rays or solar ultraviolet flux, they do not require the high radiation environment of Europa where they were first investigated in detail^11,12,32^. Martian surface conditions provide an intermediary environment between the airless surface of Ceres, where irradiated halite has been identified^33^, and the relatively shielded surface of Earth.

Our observation of elevated irradiated halite spectral model values for southern hemisphere observations in the first half of the martian year ($L_{S}$ = 0^∘^–180^∘^) may allow us to constrain the mechanisms responsible for color center formation. We first need to consider whether we are seeing a seasonal component because of the changing solar flux or due to atmospheric/surface factors changing the efficiency of color center production. Despite seasonal differences due to inclination, the solar irradiation, including the ultraviolet radiation dose, is greater at higher $L_{S}$ in the southern mid-latitudes due to the impact of the relatively high orbital eccentricity^34^. If color center formation was solely driven by solar ultraviolet flux, we would expect to see the inverse of our observations. These observations appear to counter ultraviolet irradiation as the radiation source forming the color centers. As Mars reaches perihelion ($L_{S}$ = 250 ^∘^; Fig. 6b) there is generally greater global atmospheric opacity ($\tau$) at higher $L_{S}$, peaking just beyond perihelion^34,35^ (Fig. 6c). Therefore, when we are observing relatively elevated irradiated halite values, the atmosphere is at its lowest opacity and the planet is closer to aphelion. Similarly, these observations may also rule out ESD as the radiation source, as with the increased atmospheric opacity the dust activity increases, which is perhaps the time one would expect to see greater color center formation from ESD, the opposite of our observations.

The second significant component to consider is the surface temperature, as color center formation is more efficient at lower temperatures and thermal annealing is slower^12^. All of our observations are in the southern hemisphere, and thus they follow the southern seasonal changes, with lower temperatures observed for the southern autumn and winter ($L_{S}$ = 0^∘^–180^∘^). Modeling the surface temperature at our CRISM observations as a function of elevation, slope, albedo, opacity, and thermal inertia^36,37^, we see that the individual and mean temperatures follow this trend (Fig. 6d).

The third significant component to consider is the radiation source. The two main sources to consider are galactic cosmic rays (GCRs) and solar energetic particles (SEPs). The Mars Science Laboratory (MSL) radiation assessment detector (RAD) observations suggest that GCRs are the main component of high-energy particle radiation^28,31^. The globally lower atmospheric opacity at low $L_{S}$ could imply that more GCR energy is reaching the surface, and thus creating more color centers within the chloride deposits. While factors such as the solar modulation were found to significantly affect radiation dose rates measured by RAD, seasonal pressure changes had measurable effects on the data as well^31,38^. This low $L_{S}$ timing would coincide with decreased surface temperature. These observations could favor GCRs as the radiation source, however, while the GCR flux would be similar at Mars^39^ and Ceres^40^ with both having energies reaching > 10^13^ eV, the combination of dose rate with other factors such as surface erosion may suggest that GCRs are insufficient for color center production at Mars.

Conversely, while sporadic, the instantaneous dose rate of SEPs may be orders of magnitude greater than the background GCR dose rate^41,42^. These events are emitted by the Sun and can cause a rapid increase in the surface radiation environment for several days. Furthermore, when interacting with the atmosphere, both GCRs and SEPs create secondary particles via a nuclear cascading process that will also irradiate the surface regolith^31,43^.

The same temperature effect would apply with solar ultraviolet flux with lower temperature increasing the efficiency of color center production. The ultraviolet flux is also 4 orders of magnitude higher than the GCR flux at the uppermost surface, and the ultraviolet energy is fully absorbed in the top $\sim$ 1 mm of the surface^44,45^. The inverse trend of irradiated halite spectral fractions with the seasonal ultraviolet dose rate at the surface^34^ leads us to focus our attention on other sources. The color centers we observed may have been recently formed by SEP events, and then they persist with the seasonal variation explained by variations caused by the interplay of thermal annealing and the combined incident radiation dose from GCRs, SEPs, secondaries, and ultraviolet flux.

As seasonal temperature variation may be a factor in color center formation, temperature effects in general warrant discussion, as temperature affects the production and stability of radiation-formed color centers. A negative correlation between higher temperature during irradiation and deeper absorption bands has been experimentally observed, and was also expected theoretically, as thermal decay of color centers (e.g., annealing) should be faster at higher temperatures^12^. During this decaying process, the band minima of the F and M centers were observed to shift to shorter wavelengths^12^, but the features may also shift as a function of temperature^46^, but this is not fully understood.

Seasonal temperature variations at the sites we analyzed reach up to $\sim$ 100 K (Fig. 6d). While the highest of these temperatures ($\ge$ 300 K) are beyond the range that has been explored experimentally, a reduction in the efficiency of color center formation over temperature steps of $\sim$ 20 K have been observed over experimental heating runs from 100 to 290 K^12^, and many of our observations are within this range.

Of the three irradiated NaCl endmembers employed in our spectral unmixing modeling, the model results favor the higher radiation dose endmember of Ref.^12^ and the endmember of Ref.^11^. Both of these endmembers have well-formed M centers, whereas the lower radiation dose endmember of Ref.^12^ was less favored. Additionally, our 720/790 ratio image analysis suggests that M centers may be present and explain the spectral variation at several of the chloride deposits investigated. Incident visible solar photons have the capability of suppressing color center formation in halite possibly counteracting the production of color centers via incident radiation of other forms^32,46,47^, and photo-bleaching would likely remove the M center first and then the F center absorption^46^.

The observation of radiation-formed color centers would suggest that the accumulation of radiation damage in the surface-exposed halite crystals outpaces the erosion rate. Estimated erosion rates from the Mars Exploration Rovers can range from $\sim$ 0.01 to 10 nm per year^48^. While these values may not apply to the chloride deposits due to the different compositions, the deposits themselves have been described as well indurated but friable^7^. Incident cosmic rays penetrate several orders of magnitude deeper than this, and they can damage materials in the upper few tens of centimeters^14^. As the top tens of centimeters may be significantly radiation processed on the order of 30,000 years^14,49^, the erosion of the surface likely cannot keep pace with the accumulating radiation damage. We note that decay of ^40^K may also be implicated in the formation of color centers in salt deposits, and this is a topic of ongoing work.

We can conclude that a degree of radiation damage is likely accumulating in these deposits, and the rate and dose of the applied radiation is enough to exceed the competing factors of thermal annealing, photo-bleaching, and erosion.

Our work provides a suite of evidence suggesting that halite, identified via radiation damage defects, is the unique mineral contributing the enigmatic spectral properties of the chloride deposits. The presence and abundance of halite in these alkali halide-bearing deposits would suggest a rock composition unlike any other formation yet explored on Mars. The aqueous events from which the deposits were derived must have had waters rich in sodium and chloride. The simplest source for the sodium would be the weathering of basaltic rocks, as they lead to quick leaching and mobilization of sodium^50^, and the bulk of the material comprising these deposits is likely basaltic in nature^7^. This leaching was observed in situ with the Wet Chemistry Laboratory on the Phoenix Mars Lander where sodium and magnesium cations dominated the leached cations^51^.

These alkali halide-bearing deposits of Mars are the chemical remnants of the weathering of basaltic rocks that was relatively prolonged in comparison to other weathered rock formations on Mars with more modest evidence for chemical evolution. Thin coatings of halite can obscure other minerals at thermal infrared wavelengths and affect modeled spectra in a non-linear way^52^, though this phenomenon is not as well understood at visible wavelengths.

The widespread nature of these deposits across the southern highlands, as well as the uniqueness of the alkali halide composition and abundance, may point to the mechanism involved in their formation. The presence and abundance of sodium chloride, which has a long residence time in aqueous solutions undergoing water–rock interaction^53,54^, may implicate large, long-lived bodies of water in contact with a geologically active crust^53,54^. Differentiating whether the observed halite was derived from large salty ancient lakes on Mars, or evaporitic sequences forming in freshwater lakes, will likely require in situ measurements.

Future in situ exploration of these deposits could greatly improve our understanding of the aqueous geochemical history and habitability of Mars and greatly advance our understanding of radiation processes altering materials on the surface. A landed scientific mission to explore the composition and geological history of these deposits would yield insights into the history of surface water activity on Mars, confirm the composition of an enigmatic landform, and visit a locale of significant interest for astrobiology^55^.

Irradiated halite on the surface of Mars further complicates the already complicated picture of the surface environment, in particular for the preservation of organics, biosignatures, or other evidence for life. More work is needed in investigating this topic as we interrogate the surface of Mars and search for signs of life in the Solar System.

## Online methods

### Spectroscopic images

Our analyses were performed on Compact Reconnaissance Imaging Spectrometer for Mars (CRISM) targeted empirical records (TER) data^56,57^. These data are derived from targeted spectroscopic observations and have had standardized post-processing steps applied. These steps calibrate the illumination and observation geometry as well as apply corrections for atmospheric gas absorptions and instrument artifacts. All 642 globally mapped chloride deposits^2^ were surveyed to identify corresponding CRISM TER observations (Fig. 3). This resulted in 85 CRISM images that were the focus of our analyses (Fig. 3, Supplementary Table S1).

CRISM spectral data were collected with a visible to near-infrared detector “S” (0.362–1.053 $\upmu$m) and an infrared detector “L” (1.002–3.920 $\upmu$m)^56^. TER data are available as joined “J” data products that incorporate data from both detectors in a single image cube^57^. As we were investigating the presence and distribution of radiation-formed color centers, we focused on the shorter wavelength component before the detector join. Data from the L detector were largely ignored for our analyses, with the exception of occasional inspection of the $\sim$ 2 to 2.5 $\upmu$m range to confirm the absence of narrow vibrational features from mineral such as phyllosilicates or carbonates. Data for our spectral modeling was taken solely from the wavelength range of the S detector as the discontinuity between the two detectors, the correction factors to compensate for said discontinuity, as well as the overlap in spectral values for the two detectors would negatively impact spectral modeling efforts.

We also probed the data using custom summary parameters, spectral curve fitting, and image ratios to search for evidence of radiation-induced color centers. We additionally investigated images of Jezero crater and icy surfaces of the northern polar deposits. None of these results are presented here.

### Spectral endmembers

Mineral endmembers employed in our spectral modeling analyses were chosen to mimic the mineral diversity expected to contribute absorption features at visible wavelengths, as highlighted through the Minerals Identified through CRISM Analysis (MICA) library^23,58^. With the exception of the irradiated phases, all laboratory mineral endmember spectra were taken from the Reflectance Experiment Laboratory (RELAB)^59,60^ spectral library. The laboratory spectra used are listed in Supplementary Table S2.

We utilized three laboratory spectra of irradiated halite in our analyses that represent variation in irradiation conditions and doses. The first spectrum was irradiated for 1254 min by 10 keV electrons with a current of 1 ${\upmu }$A^11^. This spectrum has prominent color centers at 0.46 $\upmu$m and 0.72 $\upmu$m assigned to irradiated NaCl and a third prominent absorption at 0.83 $\upmu$m assigned to potassium impurities in the sample. The second and third irradiated NaCl spectra were from a time series suite that were irradiated by 10 keV electrons with a current of 0.250 ${\upmu }$A^12^. The first was taken from the start of the time series at 60 min and the second was taken near the end of the time series at approximately 4725 min. These specific spectra from the time series were chosen such that the first spectrum displayed a well-formed F center (0.46 $\upmu$m) and the second spectrum displayed both a well-formed F center and a M center (0.72 $\upmu$m). All three spectra are of 300 $\upmu$m NaCl grains measured under a vacuum of $\sim$ 10^-8^ torr and at a temperature of 100 K.

We incorporated four non-physical endmembers in the spectral unmixing modeling. They included positive and negative slopes across the whole spectral range, and neutral bright (unit reflectance) and dark (zero reflectance) spectral contributors. These endmembers were included to account for residual spectral and calibration effects, including shading and shadowing, particle size effects, differences in spectral contrast between the mineral samples, and residual calibration factors. Our spectral modelling approach also included the use of an in-scene endmember that consisted of the mean of the detector column for a given pixel. This endmember aids the spectral model in accounting for residual uncalibrated image data factors, or spectral processes.

While halite is geochemically preferred to be the predominant chloride salt in martian deposits^7^, we also unmixed each CRISM image using an irradiated sylvite (KCl) endmember from Ref.^61^. Irradiation experiments demonstrate that sylvite also forms readily identifiable color centers at $\sim$ 0.55 and $\sim$ 0.83 $\upmu$m^11,61^. A null result for the detection of irradiated sylvite was observed in both the factor analysis and target transformation analysis as well as the spectral unmixing analysis.

Our initial analysis techniques (curve fitting, band ratios) never suggested the presence of irradiated sylvite. No positive detections were observed for irradiated sylvite in any of the factor analysis and target transformation models. Furthermore, in our spectral unmixing, irradiated sylvite was never favored by the model and no spectral fraction was assigned to this endmember. As there were no positive detections, this also means that there were no false positive detections, which increases confidence in the positive detections of irradiated halite using factor analysis and target transformation.

We conclude that irradiated sylvite is not likely present, the single laboratory endmember we used in our analyses is not representative of irradiated martian sylvite, or other factors complicate its detection. One such factor that could complicate its detection is the similarity in wavelength position and absorption shape between radiation-formed color centers in sylvite and ferric iron absorption features. For example, ferric iron has absorptions at $\sim$ 0.53 $\upmu$m and 0.86 $\upmu$m which coincides with F and M center locations in sylvite^62,63^. An example of these absorption features being used to characterize iron bearing materials on Mars includes the use of band depth at 0.55 $\upmu$m to identify ferric iron with OMEGA data^22^, and positive values in every pixel have been observed^22^, matching what we saw with our KCl color center band depth search. Additionally, analyses using OMEGA applied a 0.88/0.78 $\upmu$m ratio to map nanophase ferric oxides on a global scale^64^. Further evidence can be observed in selection of camera filters at 0.53 $\upmu$m and 0.86 $\upmu$m to capture iron oxides and iron charge transfer absorption^65^.

### Factor analysis and target transformation

Factor analysis and target transformation methods^66^ have previously been applied in mineralogical analyses of CRISM spectral data^24–27^. Summarized here, these methods first involve a factor analysis that calculates a series of orthogonal eigenvectors and the associated eigenvalues from a group of CRISM spectral data. The target transformation then consists of linear fits of the eigenvectors to laboratory endmember spectra to test for the presence of the spectral endmember in the original mixed data. These methods can isolate endmembers that are in complex spectral convolutions and whose spectral signatures are not readily visible in the original data due to the convolutions or low abundances. For a detailed description of factor analysis and target transformation methods and their application to the CRISM data set, refer to Ref.^24^.

For our factor analysis, we calculated the first ten eigenvectors and eigenvalues as these generally contain the majority of the spectral variation present in the data^24^. The calculation was performed on a spatial subset of CRISM spectral data over the wavelength range of 436–977 nm. This provides 73 wavelength channels for factor analysis, and maximizes wavelength coverage of the radiation-formed color centers in salt minerals while avoiding confounding CRISM calibration factors with the crossover to the infrared detector.

As the derivation of the eigenvectors requires the tandem analysis of all the measured spectra in a scene, one cannot spatially pinpoint a specific location for a mineral detection. A common approach to circumvent this issue is to spatially subset the image data in an iterative manner^26,27^. We applied a variation of this method where our factor analysis and target transformation calculations were performed on a 50 by 50 pixel subset of a given CRISM image, and this subset frame rastered across the image performing the calculations on each 50 by 50 pixel square.

For our target transformation, we performed a unconstrained linear least-squares spectral fraction estimation of the CRISM spectra using a suite of laboratory endmembers^27,67^. The laboratory endmembers utilized were the three irradiated halite endmembers and the irradiated sylvite endmember. All calculations were performed using single-scattering albedo spectra corrected for the viewing geometry of each pixel^68^, and both the modeled and laboratory spectra were normalized to reduce the influence of spectral scales on the fit^27^. The root-mean-square error (RMSE) of the target transformation was determined, and if the RMSE of a particular estimation fell below an empirically-determined^27^ threshold of $1.5\times 10^{-4}$, it was considered a positive detection.

### Spectral unmixing

We applied a Hapke^68,69^ radiative transfer model to convert both CRISM and laboratory reflectance data to single scattering albedo (SSA) using the reflectance data and viewing geometry. The model incorporated Hapke’s second-order approximation of the Chandrasekhar H-function^70^ and assumed that there was negligible contribution from the opposition effect and that the phase function was isotropic. The SSA laboratory spectra were linearly resampled to the CRISM wavelengths and used to unmix the CRISM image cubes into a new image cube of the spectral fraction of each endmember at each pixel using a linear least-squares algorithm. The algorithm applied non-negative and sum-to-one constraints. The wavelengths for the spectral modeling were clipped to focus on visible and near-infrared wavelengths and the S detector data (0.436–0.899 $\upmu$m).

To quantify the confidence on the model results for the irradiated phases we applied a statistical test following the approach of refs.^33,71^ to constrain whether the addition of the irradiated endmember improved the fit with statistical significance. In this method, the entire CRISM image cube was unmixed first using the laboratory mineral and non-physical endmembers, with the exception of the irradiated laboratory mineral spectra. Then the entire image cube was unmixed a second time using the previous endmembers plus the addition of the spectrum of one of the irradiated phase to the endmember suite. A F-test was performed comparing the two sets of model results at each pixel and only if the test found that the additional endmember improved the unmixing fit at the 99% confidence level (p = 0.01) was the irradiated phase included in the model result.

Each CRISM image was modeled a total of six times. Two endmember suites were utilized, one with a total of 12 endmembers and one with a total of 17 endmembers, with the latter suite including four additional phyllosilicates and a sulfate. Each CRISM image was modeled using both endmember suites, and for each suite the model was run three times, during which the irradiated halite laboratory spectrum was iteratively changed. All data sets produced were inter-compared. Unless otherwise specified, the results and discussion above refer to the data produced using the 12 endmember suite. Spectral model results were numerically analyzed and cross-compared in camera space, and were visualized in map space and compared to imagery of the martian surface.

As we are modeling the amount of irradiated halite on the basis of radiation-formed color centers, it is possible that halite without color centers is mixed into the spectrum that we are not detecting. Some fraction of non-irradiated halite could be raising the overall albedo, and the fraction of irradiated halite could be responsible for the spectral changes. Therefore, the model results should be considered as lower bounds as the combined irradiated and non-irradiated halite contributions could compose a greater fractional component.

### 720/790 nm ratio image

Our search of the CRISM data for spectral signatures of irradiated halite’s 0.72 $\upmu$m M center in the chloride deposits was complicated by numerous factors. The most significant being that the M center unfortunately crosses a range of CRISM wavelengths (0.61–0.71 $\upmu$m) where black paint was applied to reduce erroneous scattered light at the boundary between two filters^56^. This wavelength range is shaded grey in Figs. 2 and 4. The data are cut from the image cubes during calibration, thus hindering the investigation of this feature. Additionally, an uncalibrated spike in the data commonly peaks at $\sim$ 0.729 $\upmu$m. Applying spectral ratios can mitigate this issue as the spiking occurs in all lines for a given column. Lastly, the steep roll-off in reflectance across this whole spectral range due to the iron transitions hinders many continuum removal techniques. Nonetheless, spectra within this range covering the deposits, when normalized to the mean of the pixel’s column, commonly exhibit lower reflectance around 0.72 $\upmu$m than the continuum.

To investigate the CRISM data for evidence of halite M centers, we examined the roll-off in reflectance towards lower wavelengths at the location of the M center. We termed this quantity the “720/790 nm ratio”. The steps to calculate these spectral parameter images were as follows. First the entire CRISM image was converted to single-scattering albedo. Each spectrum in the image cube was then divided by the mean spectrum of the column in which the pixel resides. Two images were then created using these ratioed image cubes, each consisting of the mean of three bands near the M center of irradiated halite (channels: 0.71620, 0.72272, and 0.72925 $\upmu$m) or at the higher-wavelength plateau away from the M center but nearby the feature (0.78798, 0.79451, and 0.80104 $\upmu$m) and prior to the location of sylvite M centers. The 720/790 nm ratio image was then produced by taking one minus the ratio of these two images (i.e., 1–720/790 nm) to produce the strength of the decrease in reflectance between $\sim$ 0.72 and $\sim$ 0.79 $\upmu$m. We note that this quantity is non-unique to irradiated halite, though not many common minerals identified at Mars are expected to bear spectral absorption features at these specific wavelengths^23^. Data collected by OMEGA would not be hindered by the CRISM artifact located at M center wavelengths, but given the comparative low spatial resolution of the observations covering the chloride deposits, we opted not to explore that data set for M center spectral signatures.

**Figure 1:**
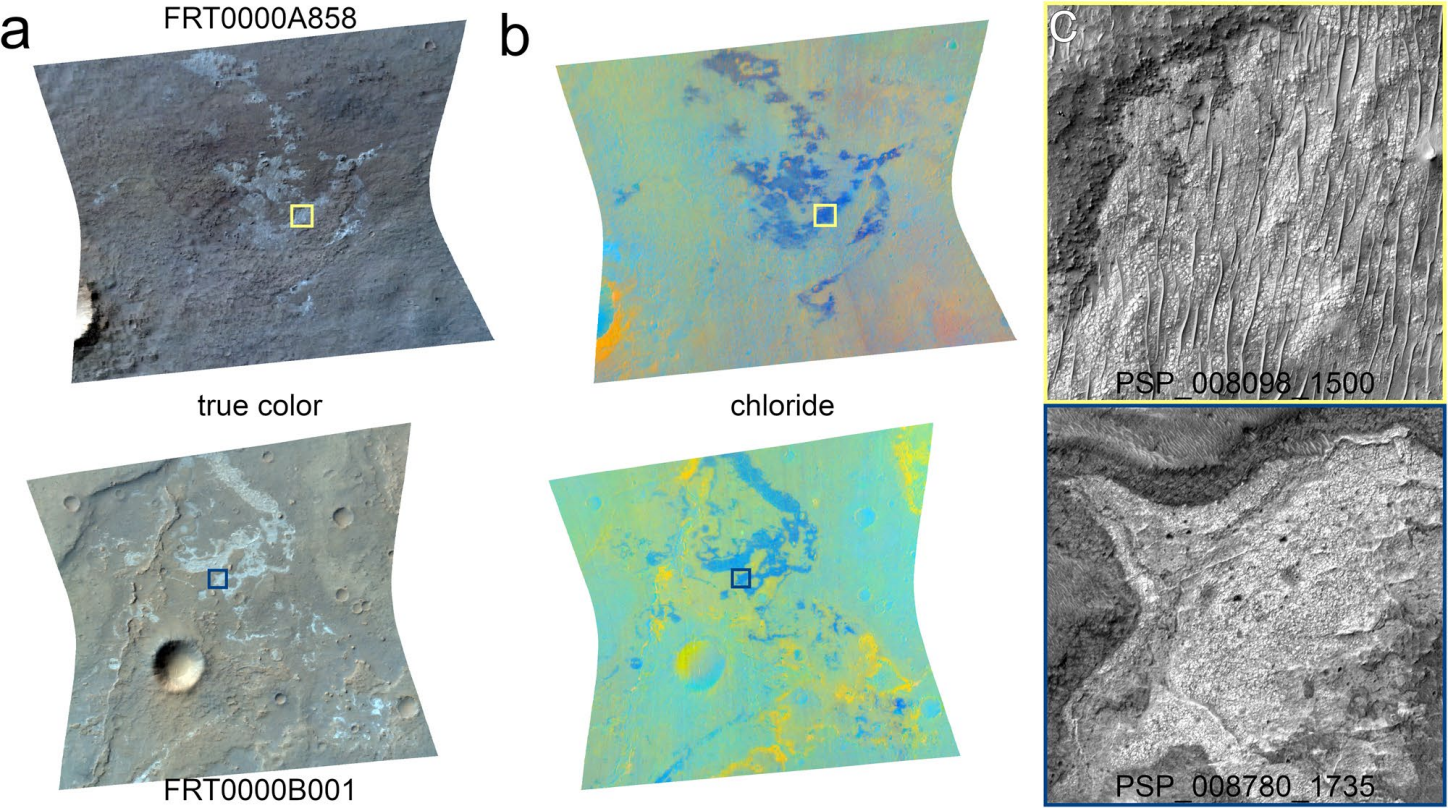
Chloride salt-bearing deposits. (**a**) True color and (**b**) chloride browse products for CRISM images FRT0000A858 and FRT0000B001. The true color image is an enhanced natural color representation of the scene, and the chloride browse product shows information related to inferred chloride deposits and spatially associated hydrated mineral deposits^23^. The chloride deposits are light toned in true color and appear blue in the chloride browse products. The CRISM images are $\sim$ 10 km wide at their narrowest. (**c**) HiRISE images of strongly blue-colored regions exhibit a light-toned fractured and blocky appearance. The colored boxes correspond to the location of the HiRISE subframes within the CRISM images.

**Figure 2:**
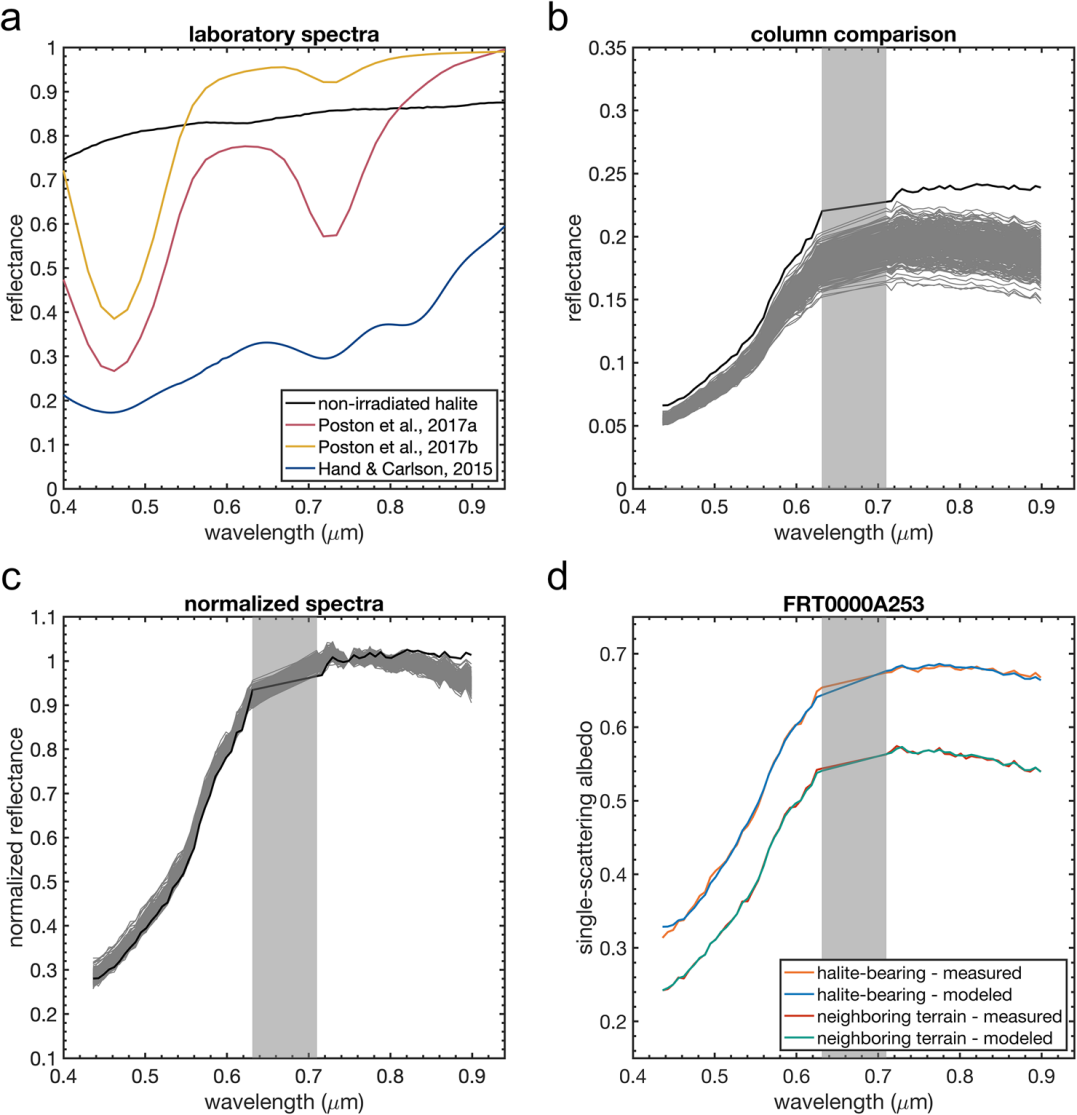
Spectral characteristics supporting the presence of irradiated halite. (**a**) Laboratory reflectance spectra of halite. Non-irradiated halite (RELAB spectrum C1JBG60A) is generally featureless at visible wavelengths. Halite exposed to high-energy electrons are shown with F and M centers at 0.46 and 0.72 $\upmu$m, respectively. The three irradiated spectra represent increasing radiation doses, with the “a” higher and “b” lower dose endmembers from Ref.^12^ being lower than the endmember from Ref.^11^. (**b**) CRISM reflectance spectrum of the chloride deposit (black) compared to all other spectra of the same detector column (53) for image FRT0000C595 (grey). (**c**) The same spectra as in (**b**) but normalized at 0.75 $\upmu$m. (**d**) Modeled and measured spectra from image FRT0000A253 of a irradiated halite-bearing pixel (404,126) and a neighboring terrain (404,62) in the same pixel column. The halite-bearing spectral model consists of 16% irradiated NaCl, 12% nanophase hematite, 1% olivine, and 71% of the column endmember.

**Figure 3:**
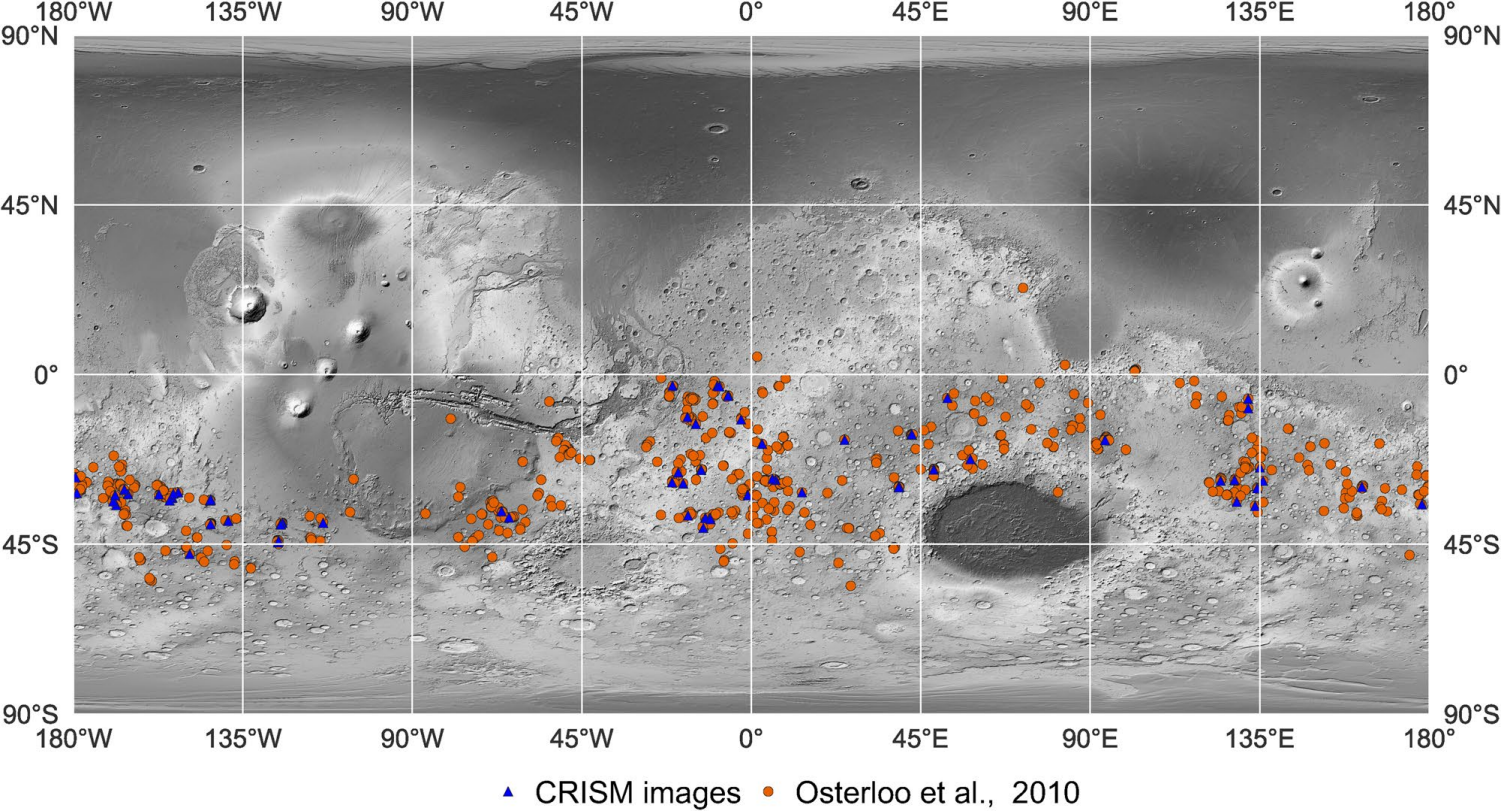
Locations of the chloride salt-bearing deposits and the CRISM images analyzed in this study. Map markers denote the locations of (orange) chloride deposits mapped by Ref.^2^ and (blue) the CRISM Targeted Empirical Records images used in this study. The basemap is a greyscale rendering of the Mars Orbiter Laser Altimeter (MOLA) global color shaded relief map^72^.

**Figure 4:**
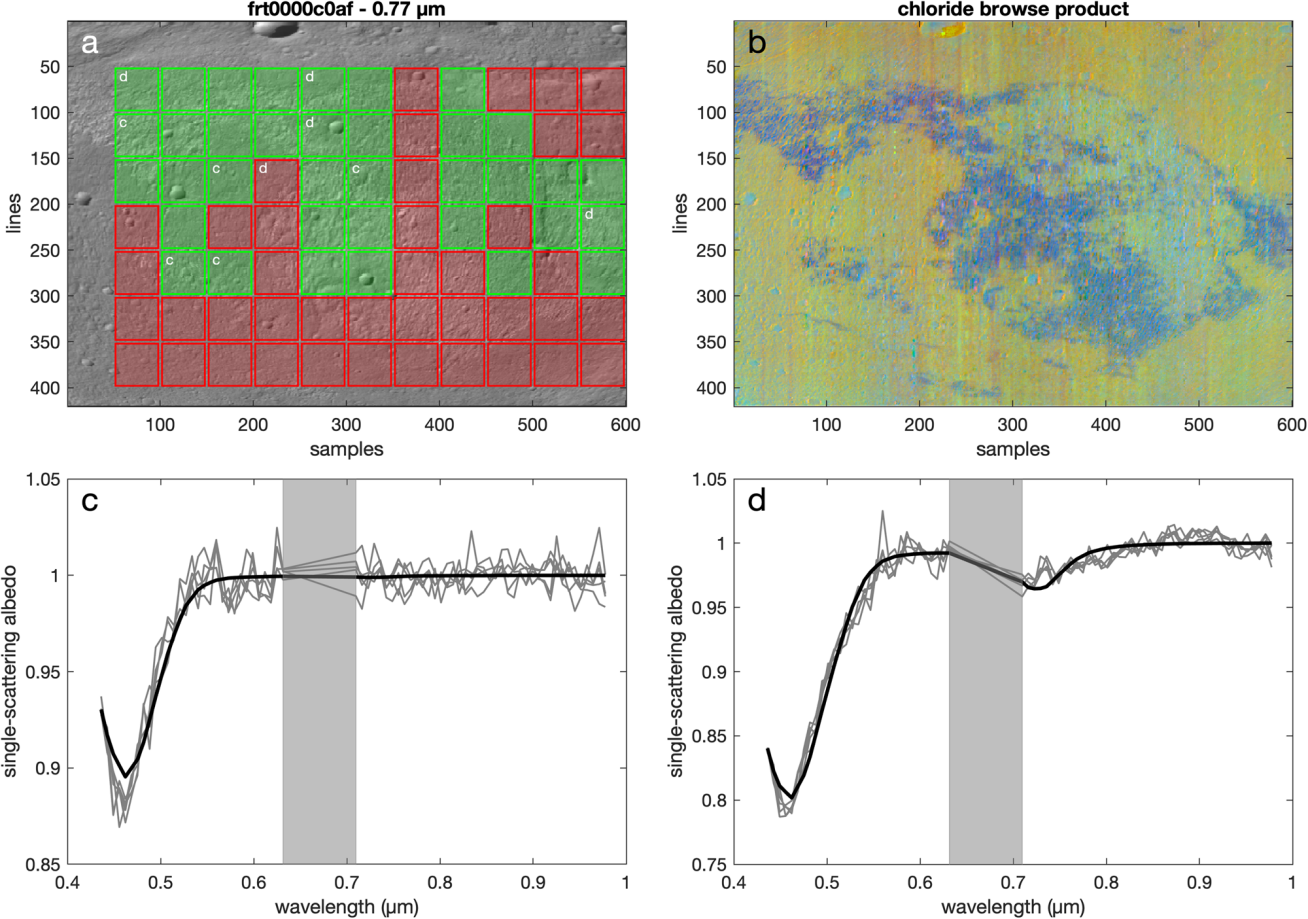
Factor analysis and target transformation (FATT) analyses suggest the presence of irradiated halite. (**a**) Eigenvectors and eigenvalues were calculated for 50 by 50 pixel squares rastering across each CRISM image. The FATT analyses were performed on these squares and if a positive match was detected (i.e., exceeding RMSE threshold), the square was colored green. Red denotes a null detection. Here, the FATT results are shown for CRISM image FRT0000C0AF and for the 60 min irradiation endmember from Ref.^12^ mapped over the corresponding 0.77 $\upmu$m reflectance band. Letters ‘c’ and ‘d’ within the image denote the extraction locations of the 5 spectra in subframes (**c**,**d**), respectively. While the positive detections for the endmembers in (**c**,**d**) are co-located with the chloride deposits, in-scene and endmember spectral variations may lead to positive detections for the different endmembers not always co-locating with each other. (**b**) The FATT positive detections spatially correlate with regions of the surface where the CRISM chloride browse products^23^ suggest the presence of the salt-bearing deposits (blue). (**c**) Five positive detections of the low dose endmember (in grey) from Ref.^12^ taken from FATT analysis of FRT0000C0AF compared to the laboratory spectrum itself (in black). A broad absorption near 0.45 $\upmu$m matches the position and shape of the F center absorption for irradiated halite. (**d**) Five positive detections of the high dose endmember from Ref.^12^ taken from FATT analysis of FRT0000C0AF compared to the laboratory spectrum itself. Despite the noise in the CRISM eigenvector spectra, broad positive slopes from 0.72 $\upmu$m towards longer wavelengths may be suggestive of irradiated halite M center absorption when observed in conjunction with the F center.

**Figure 5:**
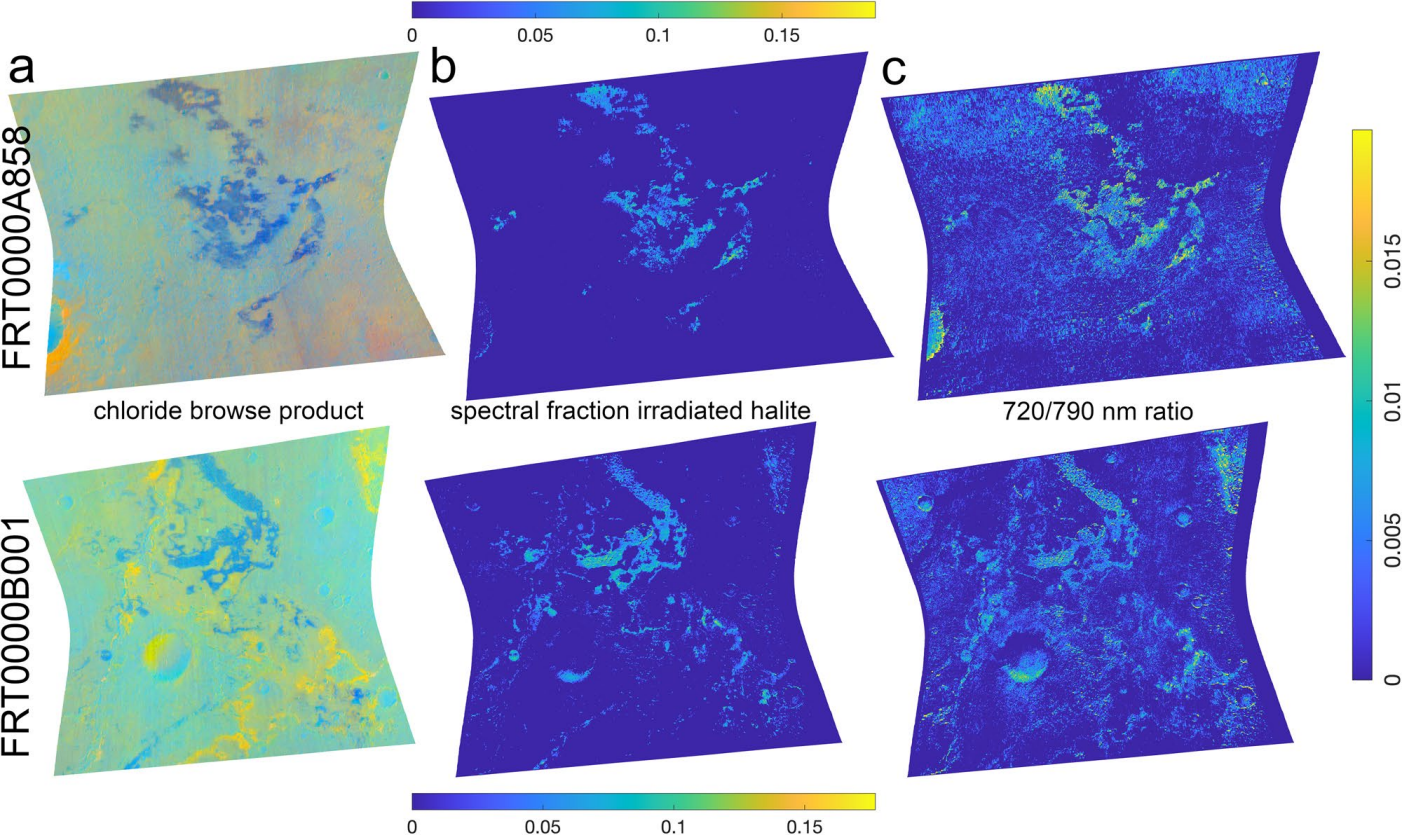
Spectral unmixing suggests irradiated halite in the chloride deposits. (**a**) CRISM images FRT0000A858 and FRT0000B001 (as in Fig. 1) were collected of chloride salt-bearing deposits. (**b**) Our spectral modeling shows spectral fractions in excess of 15% for irradiated halite at the chloride deposits in these images. (**c**) The 720/790 nm ratio images, as a proxy for NaCl M center absorption, indicate increased absorption, though the spatial distributions are not as spatially constrained to the chloride deposits as the model results in (**b**).

**Figure 6:**
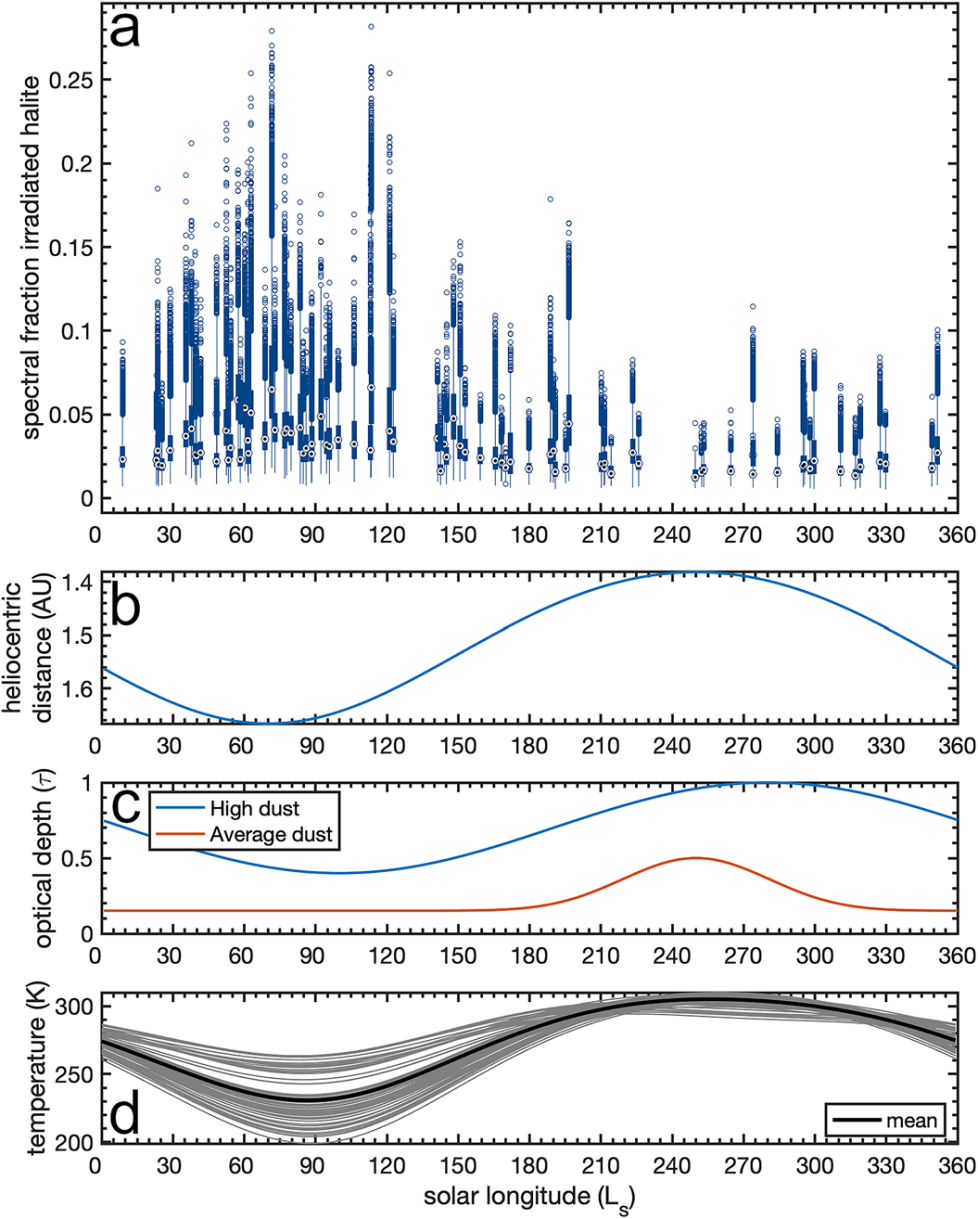
Comparison of irradiated halite model results to seasonal phenomena at Mars. (**a**) Box plots of the spectral model results for every CRISM observation using the 12 endmember results as a function of the solar longitude during the observation. Spectral fractions of irradiated halite appear to be elevated at lower solar longitudes. The (**b**) heliocentric distance of Mars somewhat leads the trend of a (**c**) high dust scenario for the optical opacity, whereas the average dust scenario sees a pulse of increased optical depth near perihelion^34^. (**d**) Surface temperature models^36^ calculated for all observation coordinates exhibit the lowest temperatures when the irradiated halite spectral fractions are the highest.

### Supplementary Information

Supplementary Information 1.

Supplementary Information 2.

## Data Availability

All data utilized in this study can be found in the National Aeronautics and Space Administration’s Planetary Data System (PDS). The CRISM Targeted Empirical Records (TER) data are available through the PDS Geosciences Node at https://pds-geosciences.wustl.edu/missions/mro/crism.htm. Laboratory spectral data were utilized in this study from the Reflectance Experiment Laboratory (RELAB) and are available through the PDS Geosciences Node Spectral Library https://pds-speclib.rsl.wustl.edu/. The accompanying Supporting Information includes a supplementary data set contains the spectral modeling results discussed in the main text as well as companion data to aid the interpretation of the reader.
