# Supplementary material for: Spectral evidence for irradiated halite on Mars
Source: Sci Rep. 2024 Mar 6;14:5503. doi: 10.1038/s41598-024-55979-6 (PMC10917766; doi:10.1038/s41598-024-55979-6)
Supplement: Supplementary file 1 — Supplementary Information 1. [file 41598_2024_55979_MOESM1_ESM.pdf]

Supplementary Information for Spectral evidence for irradiated halite on Mars

Michael S. Bramble[1,*] **and Kevin P. Hand**[1]

[1]Jet Propulsion Laboratory, California Institute of Technology, 4800 Oak Grove Drive, Pasadena, CA 91109, USA
[*]michael.s.bramble@jpl.nasa.gov

ABSTRACT

This supplementary information contains two tables, four figures, and one data set. These items are described immediately below and presented on the following pages or companion data set.

• Supplementary Table S1 lists the complete suite of CRISM images analyzed in this study as well as the date of acquisition and the latitude and longitude of each measurement. The factor analysis and target transformation results are presented for each image and each irradiated halite endmember. As a proxy for the strength of the spectral fraction model results for irradiated halite, the maximum unmixed spectral fraction is shown for both the models using the 12 and 17 endmember suites, respectively. Qualitative image results are also included comparing the chloride browse products to the 720/790 nm ratio image.

• Supplementary Table S2 lists the laboratory spectra used in the spectral modeling. They are divided into the set of 12 and set of 17 endmembers that were employed in two separate modeling efforts of the entire set of CRISM observations.

• Supplementary Figure S1 consists of a set of simple linear numerical mixtures of CRISM and laboratory reflectance spectra depicting the reflectance spectra trends associated with increasing fraction of irradiated halite.

• Supplementary Figure S2 consists of a graphical depiction of the maximum and mean model results for irradiated halite calculated for each observation with each halite endmember modeled separately and in a group of 17 or 12 endmembers.

• Supplementary Figure S3 depicts spectral model results of repeat CRISM observations. The unmixing results for of two sets of two observations to depict temporal trends in the variation of the spectral fraction of irradiated halite between lower and higher Solar longitude values.

• Supplementary Figure S4 displays the laboratory endmembers utilized in the spectral unmixing.

• Supplementary Data Set S1 consists of a page for each analyzed CRISM observation where several relevant map-projected images are shown. Included are the true color browse product, the chloride browse product, the BD530_2 parameter image, the Fe minerals version 2 browse product, the spectral fraction map for irradiated halite for each endmember from their separate runs, and the 720/790 nm ratio image. For the chloride browse product, the chloride deposits have a positive near-infrared spectral slope, therefore the chlorides often appear blue. Yellow and green colors can be indicative of hydrated minerals. For the BD530_2 parameter image, the 530 nm band depth is displayed using the CRISM band parameter calculation[1]. For the Fe minerals version 2 browse product, olivine and pyroxene are primarily detected, but nanophase ferric oxide and crystalline ferric or ferrous minerals are also highlighted. Green colors can indicate coarser-grained Fe-bearing minerals, blue colors can indicate dust-free or more mafic surfaces, and red colors can indicate nanophase ferric oxides.

Supplementary Table S1

Appearing on the subsequent pages.

Supplementary Table S1. CRISM images investigated and spectral modeling results. The complete suite of CRISM images analyzed in this study are listed along with the date of acquisition and the center latitude and longitude of the image. For each image, whether a positive detection (Y) or no detections (N) detection was observed for irradiated halite in the factor analysis and target transformation (FATT) analysis is recorded. The results are shown for the three irradiated halite (NaCl) endmembers investigated, consisting of ("^{a}") the endmember from ref.[2], ("^{b}") the higher and ("^{c}") the lower radiation dose endmembers from ref.[3]. For the positive detection, the number of 50 by 50 pixel frames detected of each endmember is given in parentheses. A qualitative comment on whether the spatial correlation between the positive detections and blue coloration in the chloride image is given with 'strong', 'good', or 'weak'. As a proxy for the strength of the spectral fraction unmixing model results for irradiated halite, the maximum unmixed spectral fraction is shown for both the models using the 12 and 17 endmember suites, respectively. The maximum spectral fractions are shown for the three irradiated halite endmembers employed. Qualitative image results are also included comparing the chloride browse products to the 720/790 nm ratio image. The presence and intensity of the blue color in the chloride image ("blue color") described. The spatial correlation of this blue color with higher values in the 720/790 nm ratio image ("blue corr.") is qualitatively described. Additionally, the correlation of higher values with intense yellow or orange colors in the 720/790 nm ratio image ("ye/or corr.") is also qualitatively described. All three of these columns present their comparison on an increasing intensity/correlation scale of "no" $<$ "weak" $<$ "yes" $<$ "strong".

				FATT detections				12 endmembers			17 endmembers			720/790 nm ratio image		
**image**	**date**	**latitude**	**longitude**	**NaCl[a]**	**NaCl[b]**	**NaCl[c]**	**corr.**	**NaCl[a]**	**NaCl[b]**	**NaCl[c]**	**NaCl[a]**	**NaCl[b]**	**NaCl[c]**	**blue color**	**blue corr.**	**ye/or corr.**
HRL00007C95	2007-09-20	-32.69	-153.74	N	Y (4)	Y (13)	good	0.00	0.07	0.00	0.00	0.05	0.00	yes	no	yes
FRT000081B1	2007-10-08	-33.28	-143.65	N	Y (3)	Y (6)	good	0.08	0.10	0.09	0.08	0.09	0.00	weak	weak	weak
HRL000082DA	2007-10-13	-27.25	-179.47	N	Y (2)	Y (7)	weak	0.05	0.07	0.06	0.00	0.05	0.00	weak	yes	yes
FRT00009042	2007-12-27	-18.44	2.73	N	N	N	n/a	0.09	0.10	0.09	0.08	0.08	0.05	strong	strong	no
FRT00009A5E	2008-01-26	-13.13	-14.75	N	Y (2)	Y (2)	weak	0.07	0.11	0.07	0.06	0.07	0.03	weak	no	weak
FRT00009AAA	2008-01-27	-33.42	-143.77	N	Y (31)	Y (7)	strong	0.18	0.16	0.00	0.15	0.07	0.00	weak	no	weak
FRT00009ACE	2008-01-28	-5.61	-6.12	N	N	Y (2)	good	0.09	0.10	0.05	0.07	0.08	0.00	weak	no	weak
FRT00009BA2	2008-01-31	-11.28	-17.00	N	N	N	n/a	0.07	0.08	0.00	0.06	0.07	0.03	weak	weak	no
FRT00009D2C	2008-02-08	-33.12	-143.56	N	Y (18)	Y (4)	strong	0.12	0.23	0.05	0.10	0.10	0.00	weak	weak	no
FRT0000A102	2008-02-22	-32.75	-154.11	Y (1)	Y (2)	Y (12)	strong	0.19	0.21	0.11	0.19	0.19	0.11	yes	weak	no
FRT0000A253	2008-02-27	-31.80	-157.31	Y (6)	Y (45)	Y (47)	strong	0.21	0.16	0.09	0.17	0.12	0.09	yes	no	weak
FRT0000A347	2008-03-02	-31.97	-0.92	N (0)	Y (1)	N (0)	weak	0.15	0.13	0.07	0.13	0.07	0.00	weak	no	no
FRT0000A385	2008-03-03	-18.39	2.87	N	Y (11)	Y (2)	good	0.13	0.28	0.08	0.09	0.06	0.00	weak	no	no
FRT0000A4A8	2008-03-07	-34.84	133.79	N	Y (5)	N	good	0.12	0.13	0.00	0.11	0.09	0.00	weak	no	no
FRT0000A858	2008-04-18	-29.72	39.11	N	Y (3)	Y (5)	strong	0.19	0.18	0.16	0.20	0.24	0.22	strong	strong	weak
FRT0000A8DF	2008-04-21	-34.65	-168.64	N	Y (3)	N	weak	0.20	0.19	0.00	0.16	0.08	0.00	weak	no	no
FRT0000A8F0	2008-04-21	-17.33	24.78	N	Y (1)	N	weak	0.10	0.18	0.07	0.07	0.00	0.00	yes	no	no
FRT0000A941	2008-04-24	-32.74	-154.11	N	N	Y (5)	strong	0.25	0.24	0.09	0.21	0.14	0.12	strong	weak	no
FRT0000ABC2	2008-05-07	-29.77	162.13	N	Y (4)	N	good	0.14	0.16	0.00	0.17	0.16	0.10	weak	no	no
FRT0000AD32	2008-06-01	-25.94	-19.42	N	Y (1)	N	weak	0.15	0.19	0.12	0.15	0.18	0.13	yes	no	no
FRT0000B001	2008-06-10	-6.42	131.95	Y (3)	Y (36)	Y (25)	strong	0.18	0.19	0.12	0.15	0.15	0.15	strong	strong	weak
FRT0000B1BD	2008-06-16	-3.05	-9.05	N	N	N	n/a	0.10	0.11	0.10	0.06	0.05	0.00	weak	weak	no
FRT0000B2D0	2008-06-21	-3.17	-8.48	N	Y (2)	Y (1)	good	0.10	0.12	0.05	0.09	0.08	0.00	yes	yes	yes
FRT0000B4BF	2008-06-30	-29.76	39.32	N	N	N	n/a	0.18	0.20	0.13	0.17	0.15	0.07	strong	strong	no
FRT0000B60B	2008-07-05	-28.87	-18.11	N	N	N	n/a	0.12	0.10	0.00	0.11	0.08	0.00	weak	weak	no
FRT0000B694	2008-07-08	-8.97	131.93	Y (1)	Y (7)	Y (3)	weak	0.13	0.16	0.12	0.11	0.10	0.06	yes	weak	weak
FRT0000B977	2008-07-16	-29.90	39.13	N	N	N	n/a	0.09	0.09	0.05	0.08	0.08	0.00	yes	weak	no
FRT0000C08D	2008-08-15	-25.03	48.50	N	Y (4)	Y (9)	good	0.20	0.23	0.14	0.19	0.16	0.11	yes	yes	weak
FRT0000C0AF	2008-08-16	-28.19	135.90	Y (7)	Y (35)	Y (36)	strong	0.28	0.30	0.13	0.24	0.25	0.12	strong	strong	no
FRT0000C4C8	2008-09-01	-24.61	135.06	Y (1)	Y (8)	Y (8)	good	0.25	0.29	0.08	0.23	0.18	0.08	yes	yes	no
FRT0000C595	2008-09-05	-25.22	-13.40	Y (1)	Y (6)	Y (2)	strong	0.14	0.15	0.06	0.13	0.12	0.00	weak	weak	no
HRL0000CEE3	2008-10-13	-31.43	-179.12	N	Y (2)	Y (9)	strong	0.09	0.10	0.09	0.09	0.09	0.05	strong	strong	no
HRL0000CF71	2008-10-16	-6.25	52.07	N	N	N	n/a	0.06	0.08	0.00	0.06	0.06	0.00	weak	weak	no
FRT0000D02E	2008-10-19	-37.97	-10.99	N	N	N	n/a	0.07	0.08	0.06	0.06	0.07	0.07	weak	weak	no
HRL0000D086	2008-10-21	-30.15	134.31	N	Y (12)	Y (1)	good	0.12	0.22	0.07	0.10	0.10	0.00	weak	no	no
HRL0000D1C7	2008-10-27	-38.83	-138.87	N	Y (2)	Y (19)	strong	0.14	0.20	0.10	0.14	0.14	0.12	strong	strong	no
HRL0000D2F1	2008-11-01	-33.67	-169.47	Y (6)	Y (24)	Y (20)	strong	0.15	0.19	0.05	0.13	0.11	0.08	weak	weak	no
FRT0000D3E9	2008-11-05	-33.73	128.90	N	N	N	n/a	0.08	0.14	0.07	0.06	0.06	0.00	weak	weak	no
FRT0000D6B7	2008-11-17	-33.10	-143.56	N	Y (7)	Y (4)	good	0.06	0.13	0.07	0.07	0.08	0.06	weak	no	no
FRT000101DE	2008-12-25	-13.13	-14.74	N	N	N	n/a	0.06	0.07	0.05	0.05	0.06	0.04	weak	no	no
FRT00010758	2009-01-10	-31.25	-152.19	Y (1)	Y (34)	Y (38)	good	0.18	0.12	0.12	0.17	0.12	0.09	yes	weak	weak
FRT000107CA	2009-01-12	-32.00	-168.94	N	Y (11)	Y (7)	good	0.08	0.10	0.05	0.08	0.08	0.05	yes	yes	yes

				FATT detections				12 endmembers			17 endmembers			720/790 nm ratio image		
image	date	latitude	longitude	NaCl[a]	NaCl[b]	NaCl[c]	corr.	NaCl[a]	NaCl[b]	NaCl[c]	NaCl[a]	NaCl[b]	NaCl[c]	blue color	blue corr.	ye/or corr.
HRL0001082C	2009-01-13	-22.47	58.15	N	N	Y (1)	weak	0.07	0.08	0.07	0.07	0.08	0.05	weak	yes	no
FRT000109DF	2009-01-21	-40.65	-12.75	N	N	N	n/a	0.00	0.05	0.00	0.05	0.05	0.03	weak	weak	no
FRT00010A4E	2009-01-23	-38.83	-139.04	Y (3)	Y (4)	Y (55)	strong	0.16	0.15	0.10	0.17	0.13	0.12	strong	strong	no
HRS00011174	2009-02-15	-31.65	-165.53	N	Y (7)	Y (8)	good	0.07	0.11	0.00	0.05	0.00	0.00	yes	no	yes
FRT00011206	2009-02-17	-44.46	-125.73	Y (1)	Y (1)	Y (1)	weak	0.06	0.08	0.00	0.05	0.04	0.00	weak	no	no
FRT00011228	2009-02-17	-17.41	93.91	N	Y (6)	Y (5)	weak	0.07	0.07	0.00	0.06	0.05	0.02	yes	yes	weak
FRT00011399	2009-02-21	-27.94	6.42	N	N	N	n/a	0.00	0.08	0.07	0.03	0.03	0.00	weak	weak	no
FRT00011815	2009-03-08	-38.84	-138.90	Y (1)	Y (9)	Y (39)	strong	0.08	0.10	0.06	0.08	0.08	0.06	strong	strong	no
FRT000118DA	2009-03-12	-37.88	-64.34	N	N	N	n/a	0.05	0.08	0.00	0.05	0.05	0.03	yes	strong	no
FRT000121F7	2009-04-19	-31.27	13.46	N	Y (2)	N	weak	0.00	0.09	0.08	0.04	0.03	0.02	weak	no	no
FRT00012362	2009-04-23	-28.60	-21.06	N	N	N	n/a	0.00	0.06	0.00	0.04	0.04	0.02	weak	weak	no
FRT000123D1	2009-04-24	-39.36	-124.48	N	Y (3)	Y (2)	good	0.00	0.06	0.05	0.03	0.03	0.02	weak	weak	weak
FRT00012A09	2009-05-12	-25.21	48.43	N	Y (5)	Y (4)	good	0.05	0.06	0.00	0.04	0.04	0.02	weak	weak	weak
FRT00012E40	2009-05-27	-39.63	-124.98	N	N	N	n/a	0.00	0.06	0.00	0.03	0.03	0.02	weak	no	no
FRT00012E44	2009-05-27	-31.55	-153.50	Y (10)	Y (28)	Y (41)	strong	0.11	0.11	0.06	0.08	0.07	0.04	yes	no	yes
FRT000132F0	2009-06-13	-44.09	-125.55	N	N	N	n/a	0.00	0.05	0.00	0.04	0.05	0.03	weak	no	no
FRT000136E6	2009-06-30	-28.04	128.35	N	Y (8)	Y (5)	good	0.09	0.10	0.09	0.09	0.09	0.07	weak	weak	weak
FRT00013706	2009-07-01	-39.70	-143.56	N	N	Y (5)	good	0.07	0.09	0.00	0.07	0.08	0.05	weak	weak	weak
FRT00013825	2009-07-05	-47.57	-149.17	Y (1)	Y (2)	N	weak	0.00	0.07	0.00	0.04	0.05	0.03	yes	strong	no
HRL00013922	2009-07-08	-11.94	-2.72	N	N	Y (7)	strong	0.09	0.12	0.06	0.09	0.10	0.06	yes	strong	weak
FRT00013F5F	2009-07-27	-39.79	-124.95	N	Y (3)	Y (5)	good	0.07	0.07	0.00	0.06	0.07	0.03	weak	no	yes
FRT000165AA	2010-02-07	-5.59	-6.02	N	N	N	n/a	0.07	0.10	0.00	0.06	0.06	0.03	weak	no	yes
FRT000165C7	2010-02-07	-39.33	-113.72	N	N	Y (1)	weak	0.16	0.13	0.06	0.14	0.12	0.04	yes	weak	no
FRT00016930	2010-02-16	-38.03	-12.23	Y (1)	Y (5)	Y (3)	weak	0.22	0.18	0.00	0.16	0.20	0.14	weak	weak	no
FRT00016A23	2010-02-18	-5.66	-6.29	N	N	N	n/a	0.09	0.09	0.00	0.08	0.06	0.02	weak	no	yes
FRT00016AF8	2010-02-20	-27.65	5.61	N	Y (8)	N	good	0.14	0.18	0.08	0.12	0.08	0.00	yes	no	no
FRT00016E3C	2010-02-27	-36.23	-66.32	N	Y (1)	Y (1)	weak	0.20	0.14	0.09	0.19	0.10	0.00	strong	strong	no
FRT00016EE8	2010-03-01	-5.65	-6.29	N	Y (1)	N	good	0.10	0.10	0.00	0.08	0.08	0.00	weak	no	yes
FRT000179D3	2010-04-01	-33.44	-154.50	N	Y (4)	Y (13)	strong	0.28	0.29	0.12	0.28	0.17	0.15	yes	strong	no
FRT00017AFB	2010-04-04	-30.54	-166.52	N	Y (1)	Y (1)	weak	0.17	0.17	0.05	0.15	0.11	0.05	yes	weak	no
FRT00017EA3	2010-04-13	-27.93	128.33	N	Y (11)	N	good	0.20	0.22	0.00	0.18	0.16	0.00	yes	weak	no
FRT00017F7B	2010-04-15	-30.54	-166.51	N	N	N	n/a	0.15	0.15	0.05	0.14	0.11	0.05	yes	weak	no
FRT0001871B	2010-05-01	-5.65	-6.16	N	N	N	n/a	0.09	0.10	0.00	0.08	0.08	0.00	yes	no	weak
FRT00018AF5	2010-05-09	-2.96	-20.94	Y (3)	Y (7)	Y (8)	strong	0.12	0.14	0.07	0.13	0.11	0.00	weak	weak	yes
FRT000195D7	2010-06-18	-15.97	42.42	N	N	N	n/a	0.17	0.16	0.06	0.15	0.14	0.00	yes	yes	no
HRS0001B704	2010-10-17	-28.14	124.61	Y (1)	Y (9)	Y (10)	weak	0.11	0.20	0.00	0.13	0.13	0.09	no	no	yes
FRT0001B804	2010-10-22	-37.36	-16.82	N	Y (1)	Y (1)	weak	0.05	0.09	0.00	0.00	0.06	0.00	no	no	no
FRT0001B81A	2010-10-22	-34.40	178.07	N	Y (4)	Y (2)	good	0.06	0.08	0.00	0.06	0.06	0.05	yes	yes	no
FRT0001B8C9	2010-10-25	-30.50	-166.50	N	Y (3)	Y (2)	good	0.00	0.07	0.00	0.00	0.00	0.00	weak	no	no
HRL0001B96E	2010-10-29	-15.93	42.69	N	Y (3)	Y (5)	good	0.10	0.11	0.07	0.09	0.09	0.00	yes	yes	weak
FRT0001EC37	2011-06-28	-25.35	-13.29	N	Y (4)	Y (5)	good	0.06	0.07	0.00	0.06	0.06	0.00	yes	yes	no
FRT0001FD35	2011-08-23	-38.38	-10.83	N	N	N	n/a	0.06	0.13	0.00	0.06	0.13	0.00	weak	weak	no

				FATT detections				12 endmembers			17 endmembers			720/790 nm ratio image		
**image**	**date**	**latitude**	**longitude**	**NaCl[a]**	**NaCl[b]**	**NaCl[c]**	**corr.**	**NaCl[a]**	**NaCl[b]**	**NaCl[c]**	**NaCl[a]**	**NaCl[b]**	**NaCl[c]**	**blue color**	**blue corr.**	**ye/or corr.**
FRT0001FDFD	2011-08-27	-33.41	-154.49	Y (2)	Y (11)	Y (36)	strong	0.10	0.11	0.07	0.10	0.11	0.10	strong	strong	no

Supplementary Table S2

Spectrum ID	Sample ID	Type	Mineralogy
**12 endmember set**			
N/A	N/A	N/A	4 non-physical endmembers
CAPO05	PO-EAC-005	Nesosilicates	Olivine (Fayalite)
C1PO50	PO-EAC-050	Nesosilicates	Olivine (Forsterite)
C2PP21	PP-CMP-021	Inosilicates	Clinopyroxene
CAPP47	PP-EAC-047-A	Inosilicates	Orthopyroxene
C1JB708	JB-JLB-708	Iron Oxide	Synthetic hematite
C1JB763	JB-JLB-763	Iron Oxide	Nanophase hematite
N/A	N/A	N/A	Mean of the detector column
N/A	N/A	Halide	Irradiated halite
**17 endmember set (in addition to the above)**			
C1EA14	EA-EAC-014	Phyllosilicate	Chlorite
C1JB791A	JB-JLB-791-A	Phyllosilicate	Nontronite
CAIL02	IL-EAC-002	Phyllosilicate	Illite
CASR13	SR-EAC-013	Phyllosilicate	Serpentine
C1JBB78	JB-JLB-B78	Sulfate	Jarosite

Supplementary Table S2. Laboratory spectra utilized in the spectral modeling. Visible to near-infrared laboratory spectra employed in the linear unmixing of CRISM image cubes. The entire image suite was modeled both using a set of 12 and a set of 17 endmembers. The irradiated halite endmember was iterated across the three different laboratory endmembers discussed in the text in three different modeling instances. The data produced using the three different halite endmembers and the two total sets of endmembers were inter-compared. The Spectrum and Sample IDs refer to entries in the RELAB data base. A plot displaying the laboratory endmember spectra can be found in Supplementary Figure S4.

Supplementary Figure S1

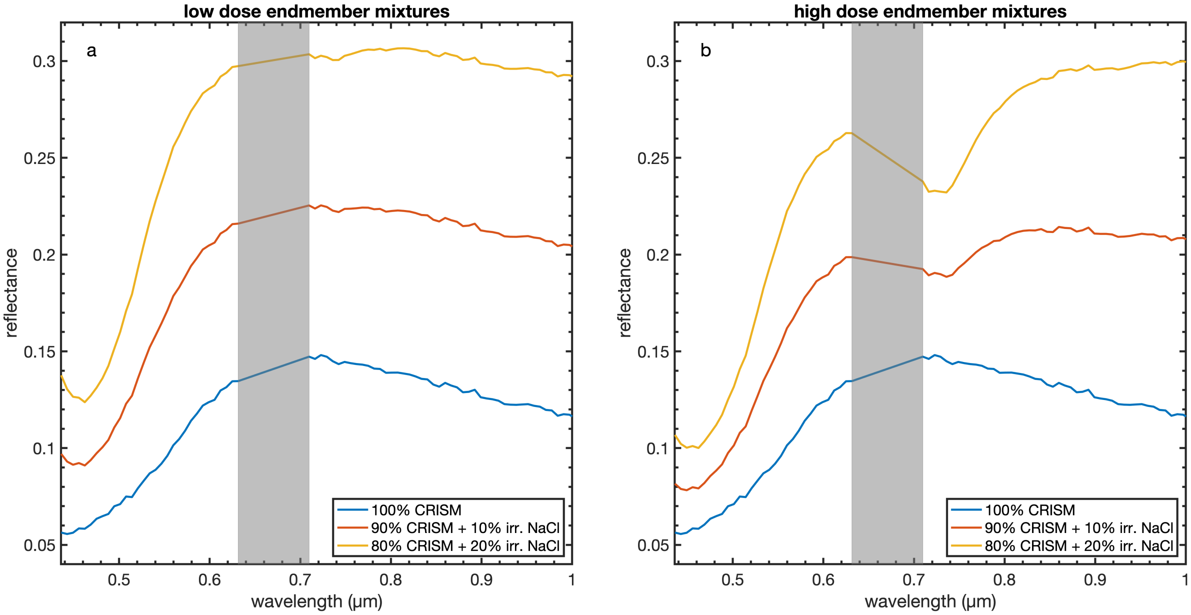

Supplementary Figure S1. Linear mixtures showing the effect of increasing irradiated halite. A set of simple linear numerical mixtures of CRISM reflectance spectra and laboratory reflectance spectra of irradiated halite. The CRISM spectrum ("100% CRISM") consists of a 3 by 3 pixel averaged reflectance spectrum taken from (x,y) position of (238,342) in CRISM image FRT0000A858_07_IF164J_MTR3. This location is taken away from the chloride salt-bearing deposits as identified using the chloride browse product and is intended as a substitute for a generic martian regolith spectrum. Numerical mixtures containing 10% and 20% irradiated halite are shown for the (a) lower radiation dose and (b) higher radiation dose endmembers taken from ref.[3]. These spectra display the reflectance spectral trends discussed in the main text. With increasing fraction of irradiated halite, the long-wavelength negative slope towards 1 μm grows shallower, and the short-wavelength component sees a steeper drop in reflectance towards shorter wavelengths. The overall albedo increases with the increasing irradiated halite.

Supplementary Figure S2

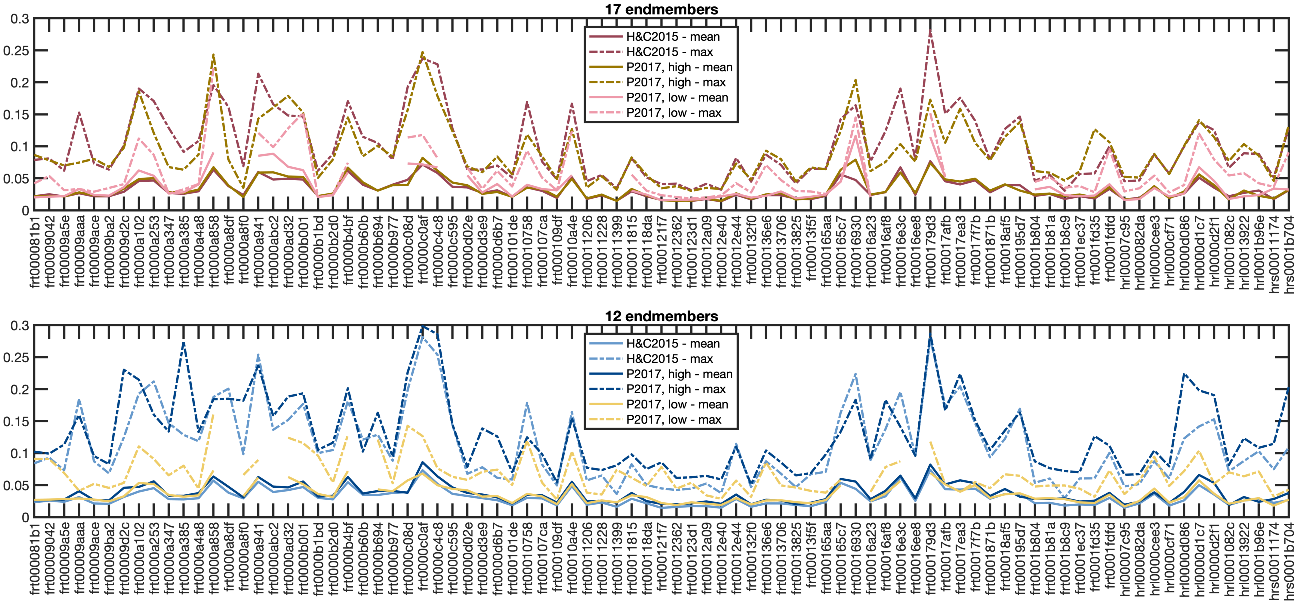

Supplementary Figure S2. The mean and maximum spectral fraction of irradiated halite for each CRISM observation investigated. The spectral fraction of irradiated halite is shown for each of the three irradiated halite endmembers (each of which were modeled individually in separate spectral models). The results are plotted as a function of image number which doubles as a chronological order. The results are shown for the results using the suite of 17 endmembers (top) and results using the suite of 12 endmembers (bottom).

Supplementary Figure S3

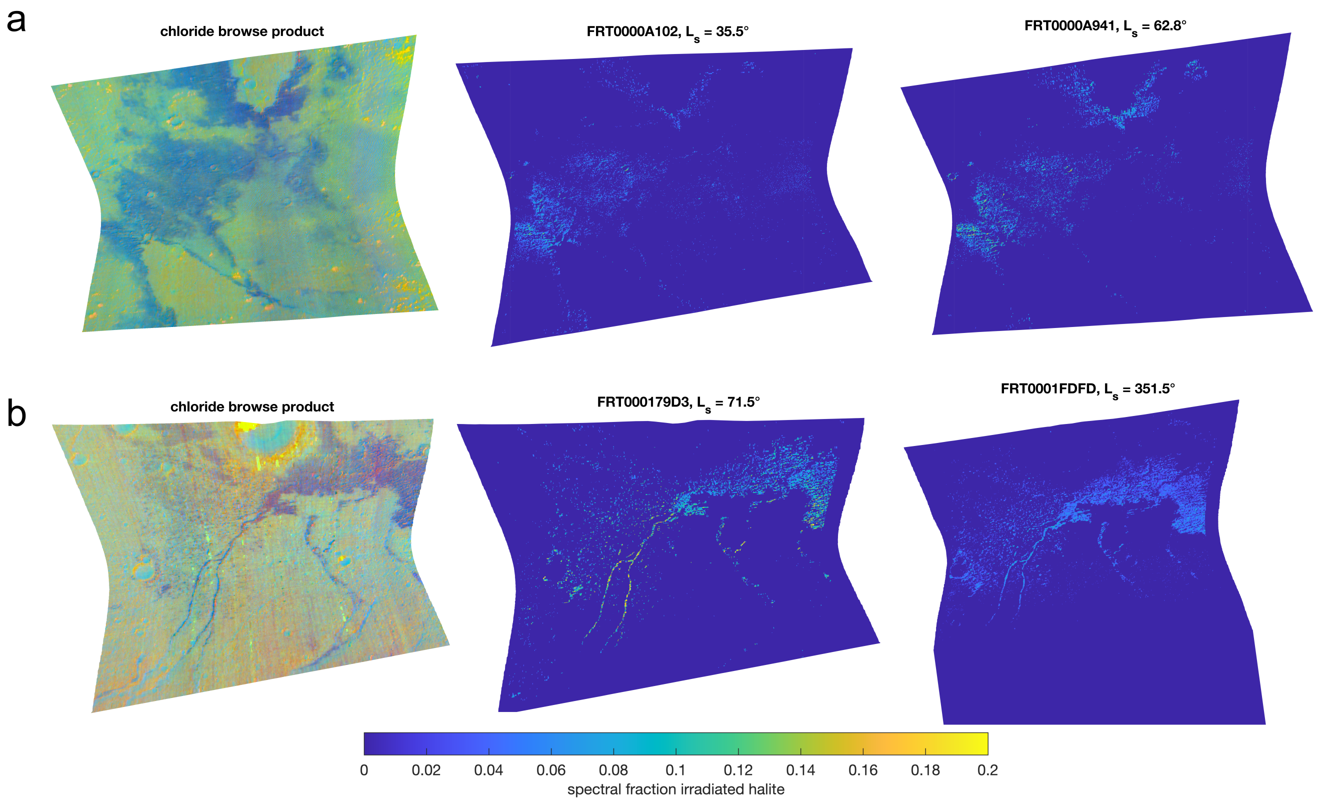

Supplementary Figure S3. Spectral model results of repeat CRISM observations. The spectral model results of two sets of two observations are shown along with their chloride browse product for comparison. (a) The first set consists of two observations (FRT0000A102, FRT0000A941) both taken during low Solar longitudes with lower surface temperatures and no significant change is observed in the modeled irradiate halite between the two observations. (b) Whereas the second set has one taken from low Solar longitude (FRT000179D3) and one at the end of southern summer (FRT0001FDFD) where the modeled irradiated halite values have lessened.

Supplementary Figure S4

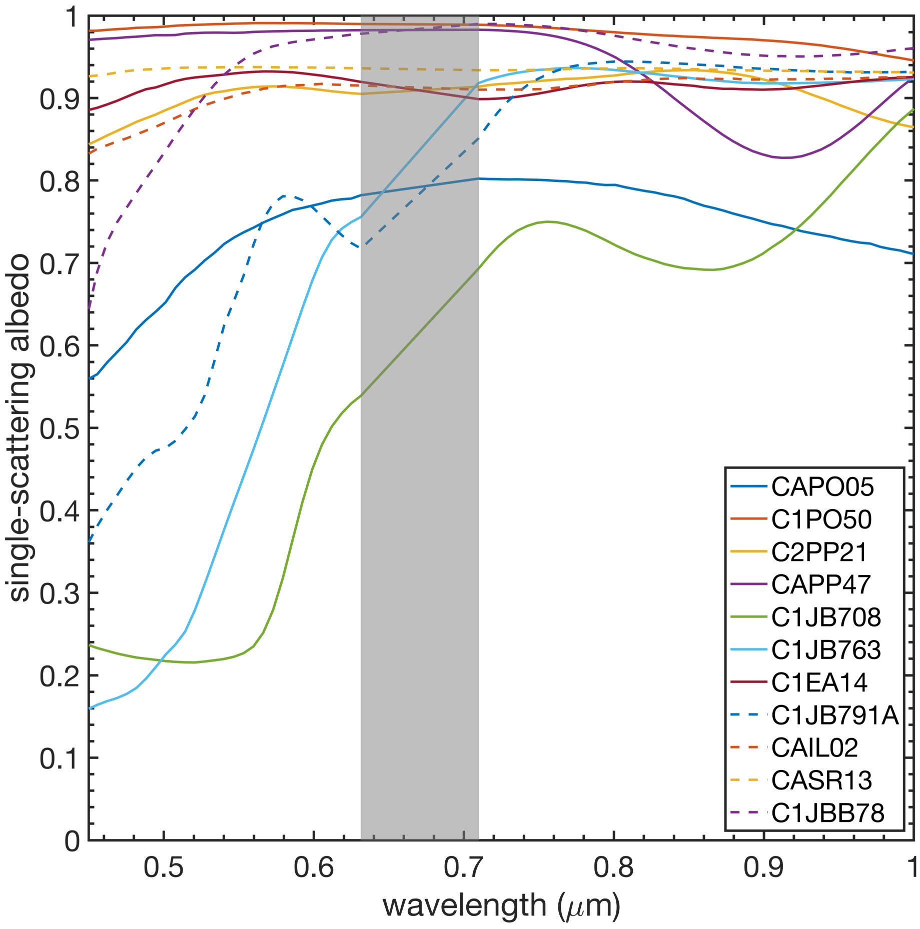

Supplementary Figure S4. Laboratory endmembers. Single-scattering albedo spectra of the laboraotry endmembers utilized in the spectral unmixing methods. Spectra can be identified by referencing the Spectrum ID to the Sample ID in Supplementary Table S2.

References

1. Viviano, C. E. *et al.* Revised crism spectral parameters and summary products based on the currently detected mineral diversity on mars. *J. Geophys. Res. Planets* **119**, 1403–1431 (2014).

2. Hand, K. & Carlson, R. Europa's surface color suggests an ocean rich with sodium chloride. *Geophys. Res. Lett.* **42**, 3174–3178 (2015).

3. Poston, M. J., Carlson, R. W. & Hand, K. P. Spectral behavior of irradiated sodium chloride crystals under europa-like conditions. *J. Geophys. Res. Planets* **122**, 2644–2654 (2017).
