# Supplementary material for: Spectral evidence for irradiated halite on Mars
Source: Sci Rep. 2024 Mar 6;14:5503. doi: 10.1038/s41598-024-55979-6 (PMC10917766; doi:10.1038/s41598-024-55979-6)

frt0000a4a8

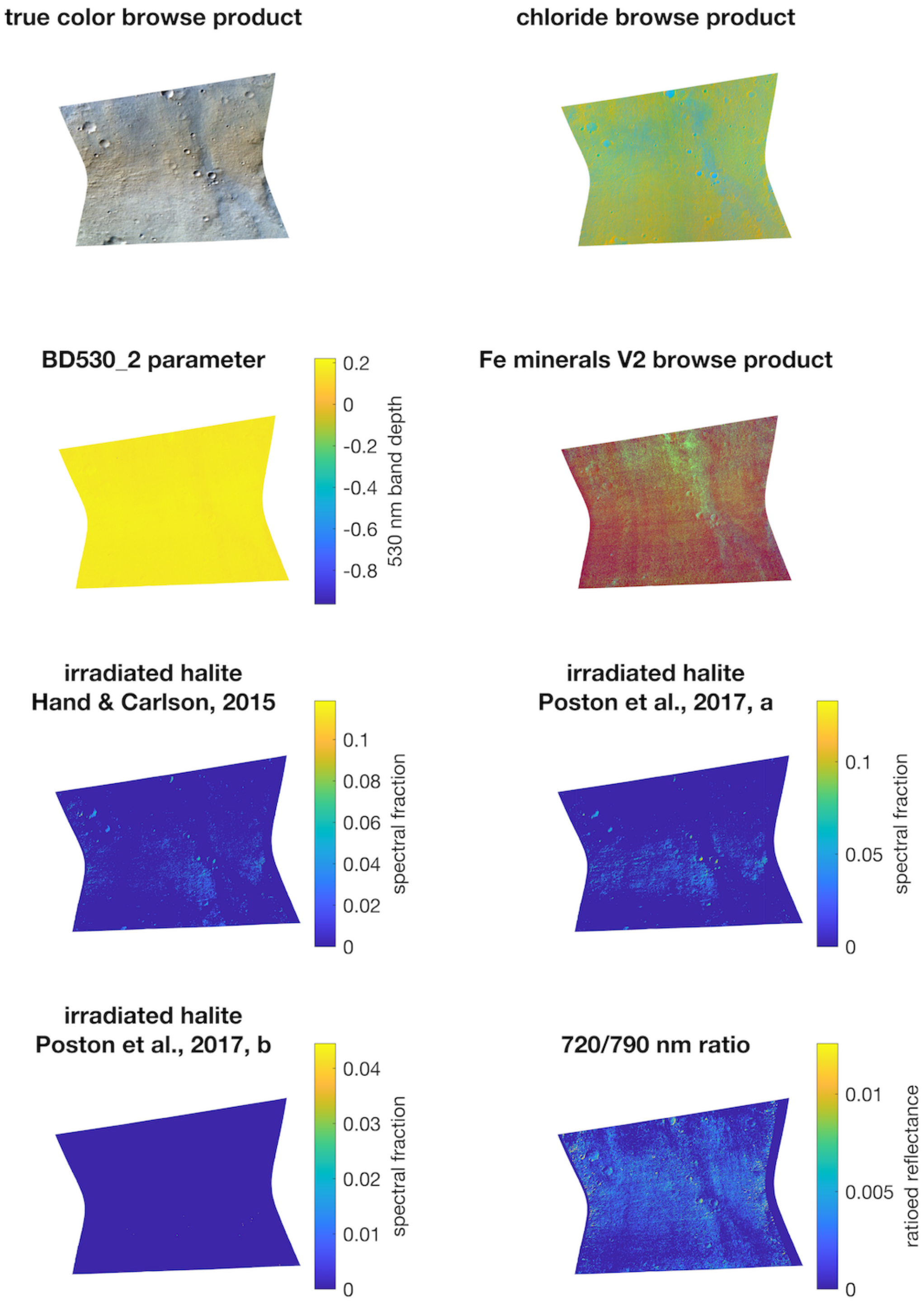

frt0000a8df

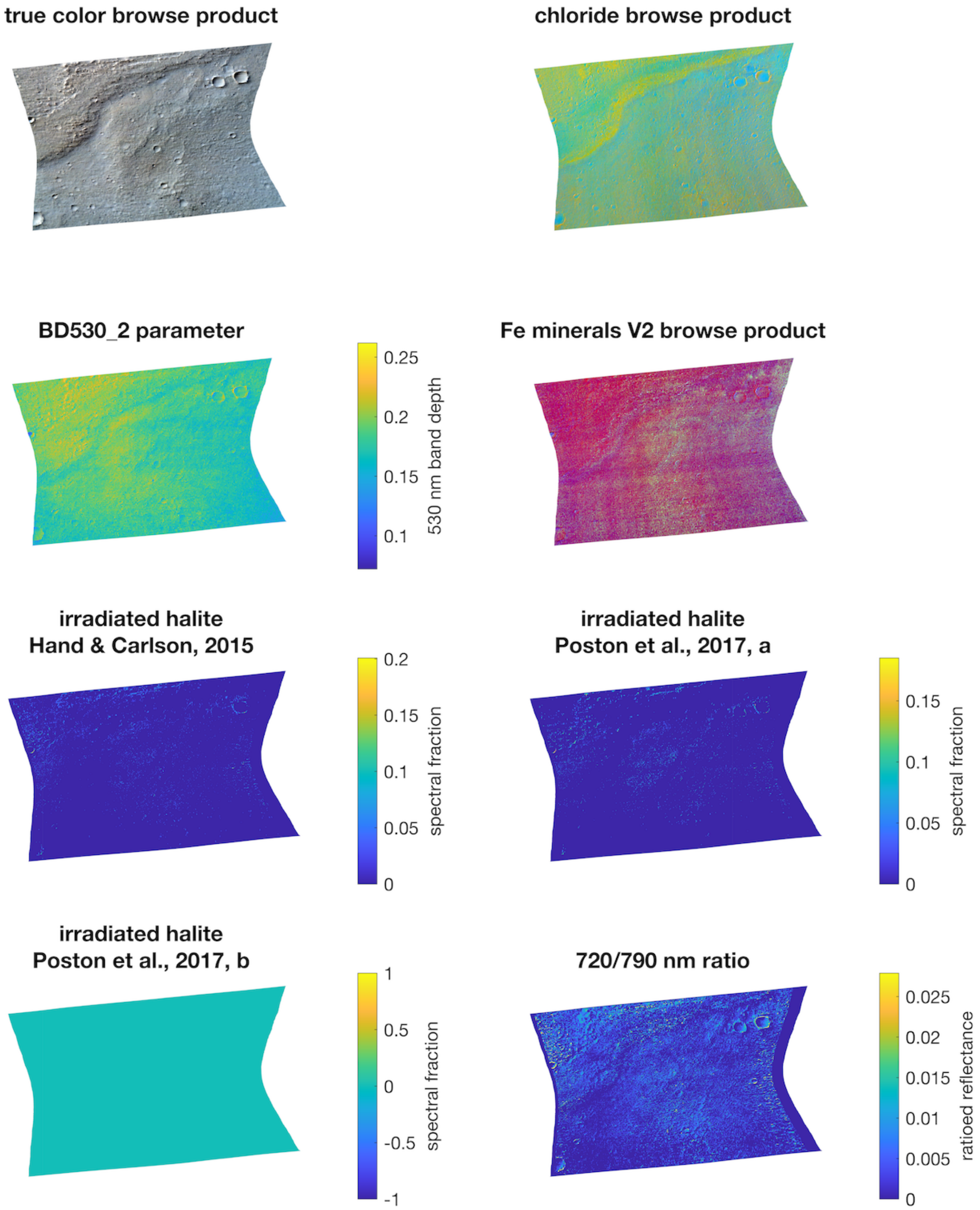

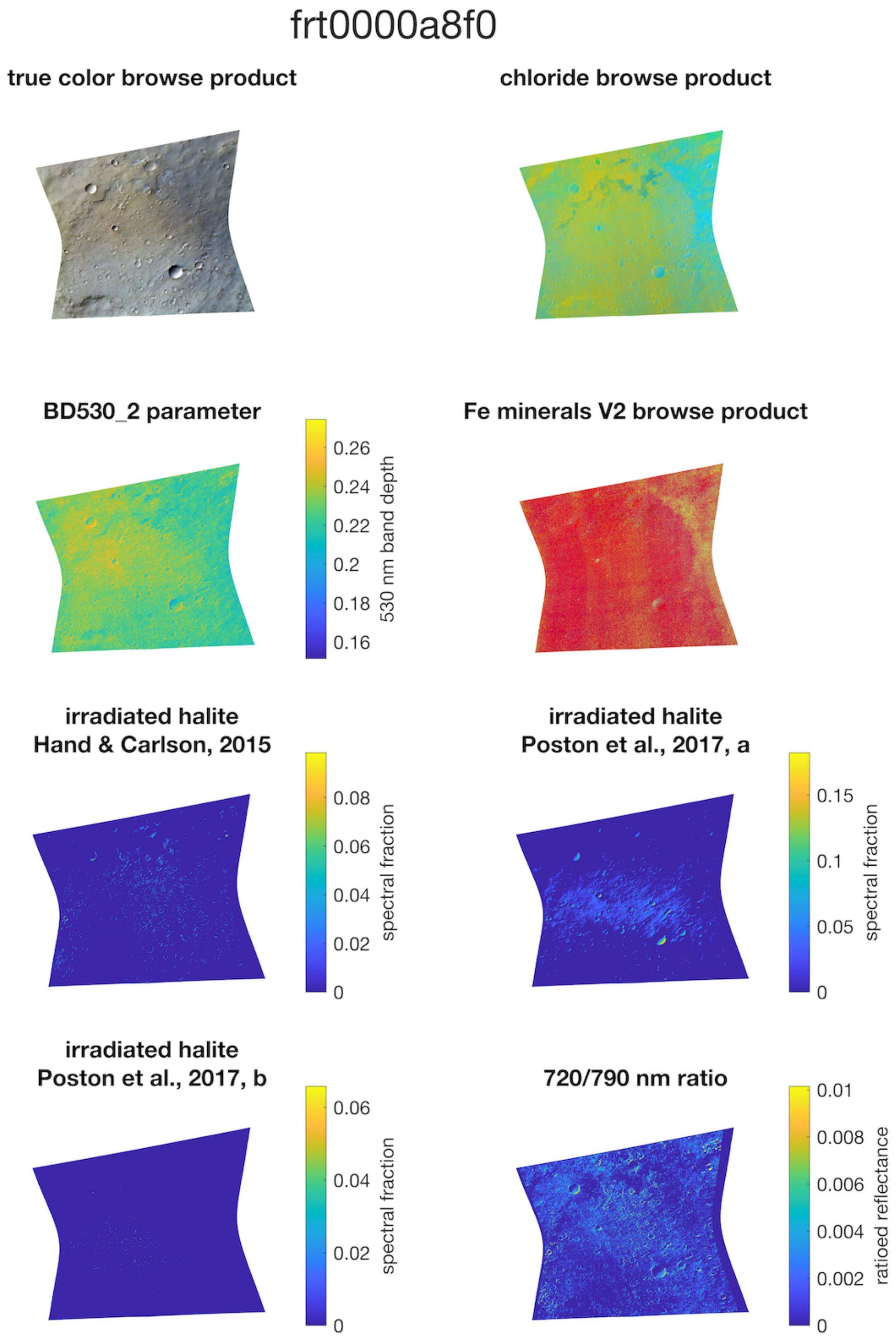

frt0000a8f0
true color browse product
chloride browse product
BD530_2 parameter
0.26
0.24
0.22
0.2
0.18
0.16
530 nm band depth
Fe minerals V2 browse product
irradiated halite
Hand & Carlson, 2015
0.08
0.06
0.04
0.02
0
spectral fraction
irradiated halite
Poston et al., 2017, a
0.15
0.1
0.05
0
spectral fraction
irradiated halite
Poston et al., 2017, b
0.06
0.04
0.02
0
spectral fraction
720/790 nm ratio
0.01
0.008
0.006
0.004
0.002
0
ratioed reflectance

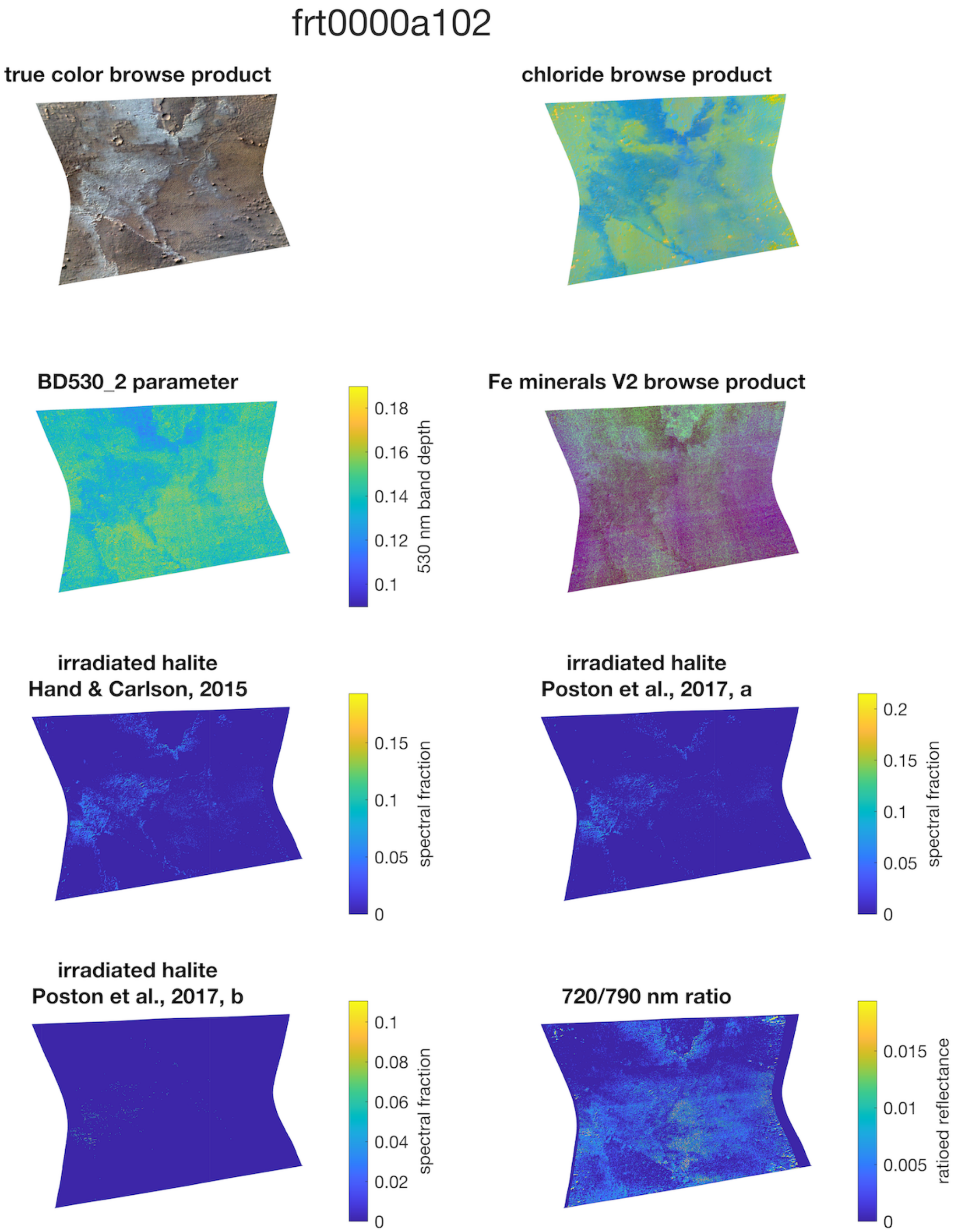
frt0000a102
true color browse product
chloride browse product
BD530_2 parameter
530 nm band depth
0.18
0.16
0.14
0.12
0.1
Fe minerals V2 browse product
irradiated halite
Hand & Carlson, 2015
spectral fraction
0.15
0.1
0.05
0
irradiated halite
Poston et al., 2017, a
spectral fraction
0.2
0.15
0.1
0.05
0
irradiated halite
Poston et al., 2017, b
spectral fraction
0.1
0.08
0.06
0.04
0.02
0
720/790 nm ratio
ratioed reflectance
0.015
0.01
0.005
0

frt0000a253

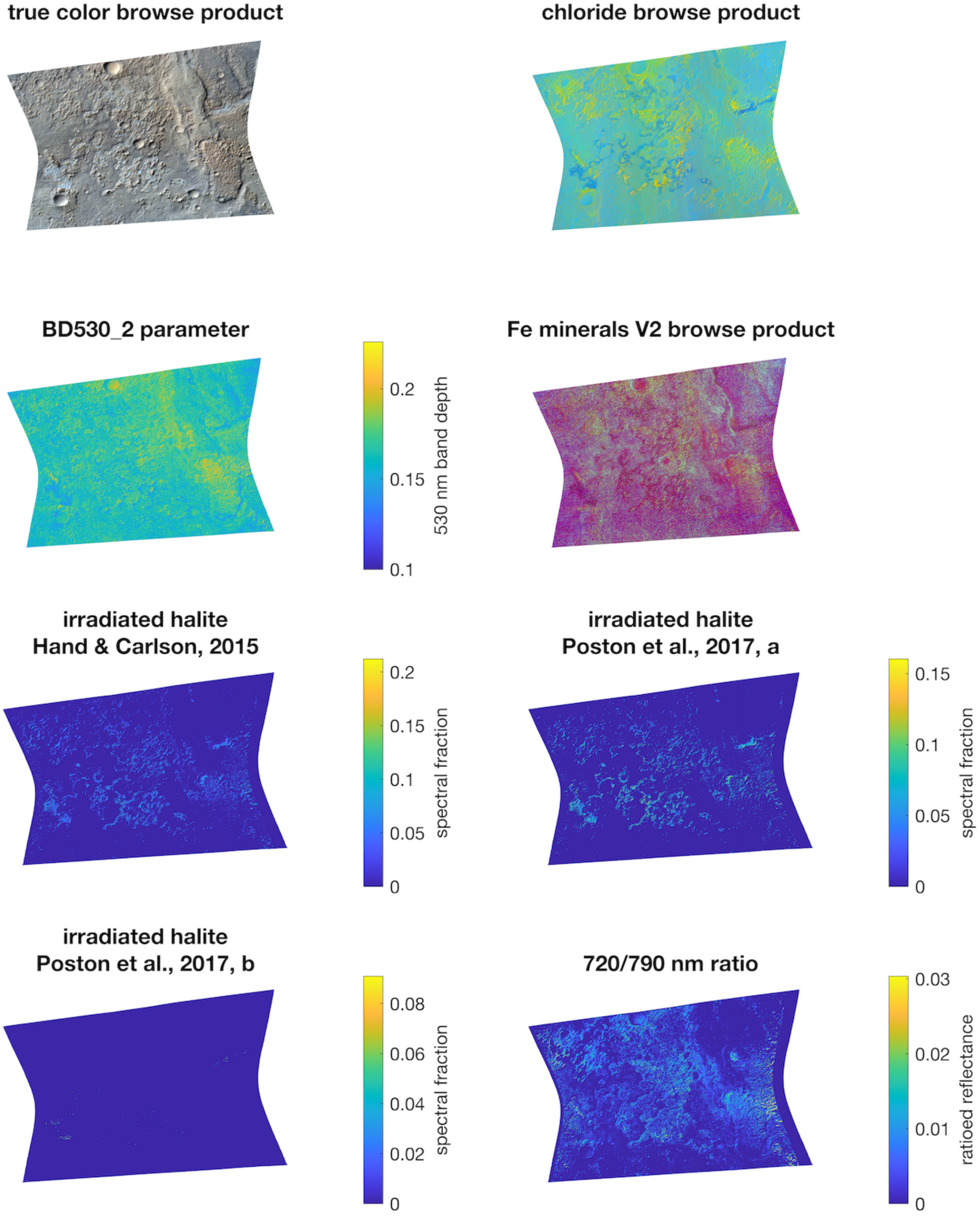

frt0000a347

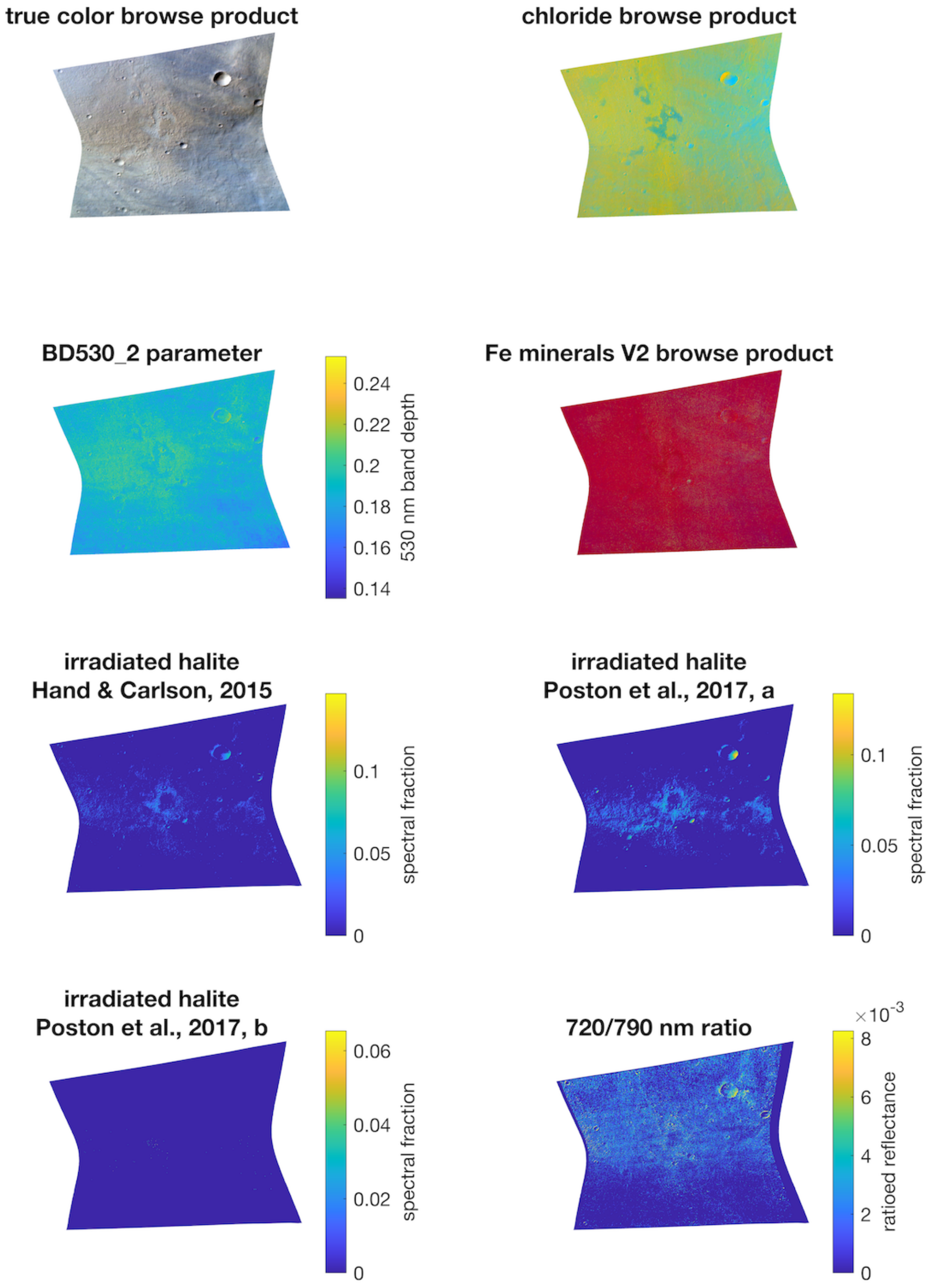

frt0000a385

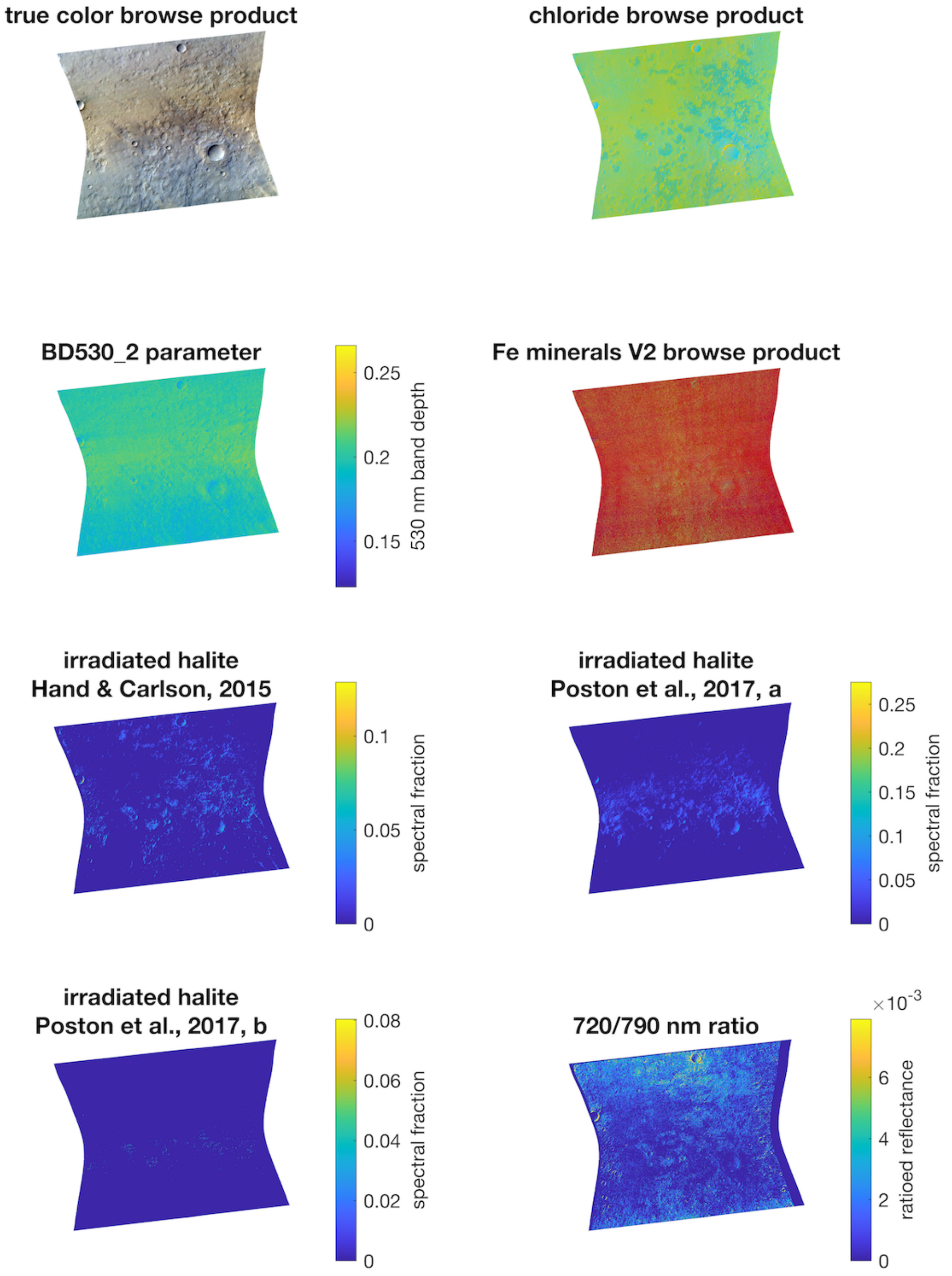

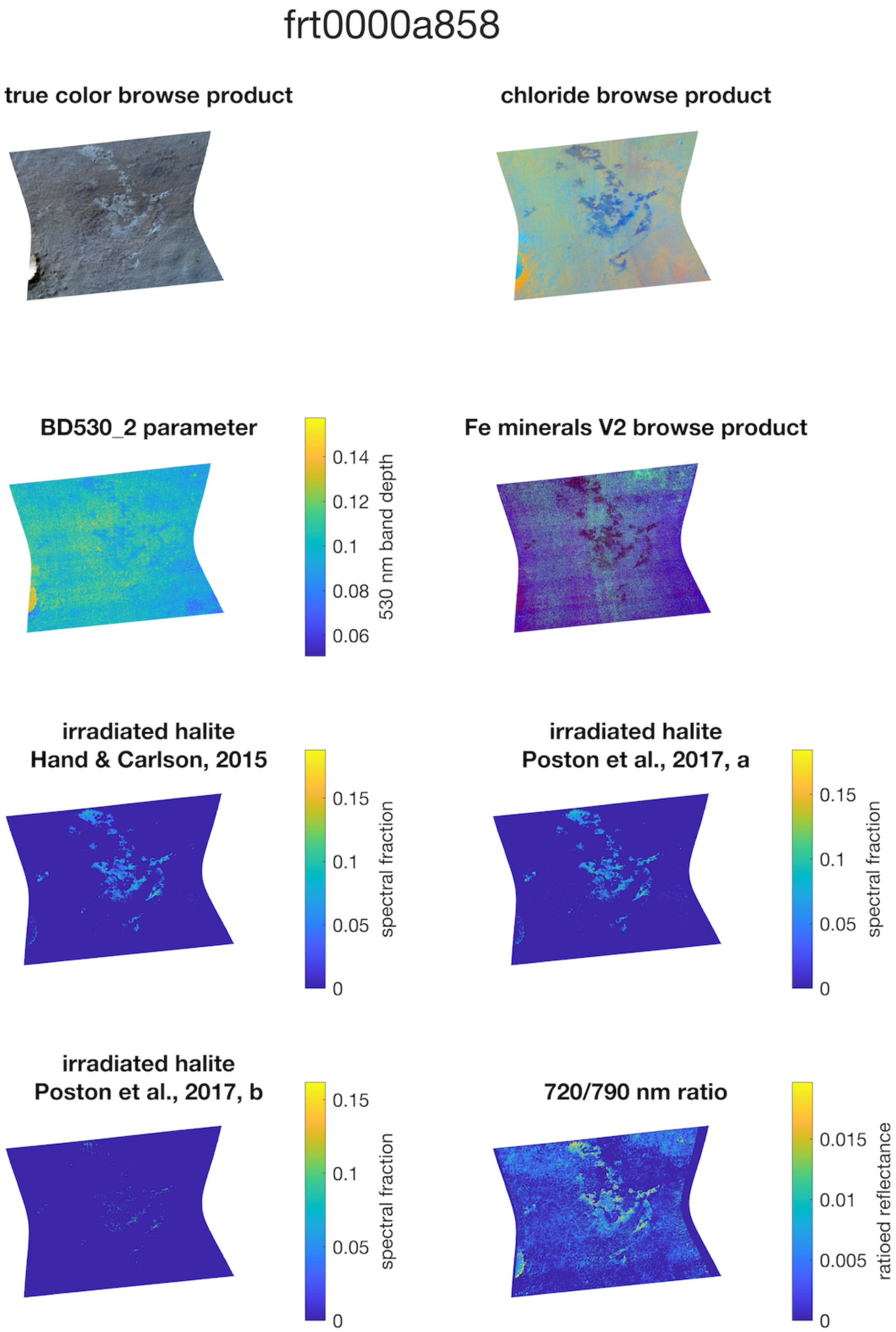
frt0000a858
true color browse product
chloride browse product
BD530_2 parameter
530 nm band depth
0.14
0.12
0.1
0.08
0.06
Fe minerals V2 browse product
irradiated halite
Hand & Carlson, 2015
spectral fraction
0.15
0.1
0.05
0
irradiated halite
Poston et al., 2017, a
spectral fraction
0.15
0.1
0.05
0
irradiated halite
Poston et al., 2017, b
spectral fraction
0.15
0.1
0.05
0
720/790 nm ratio
ratioed reflectance
0.015
0.01
0.005
0

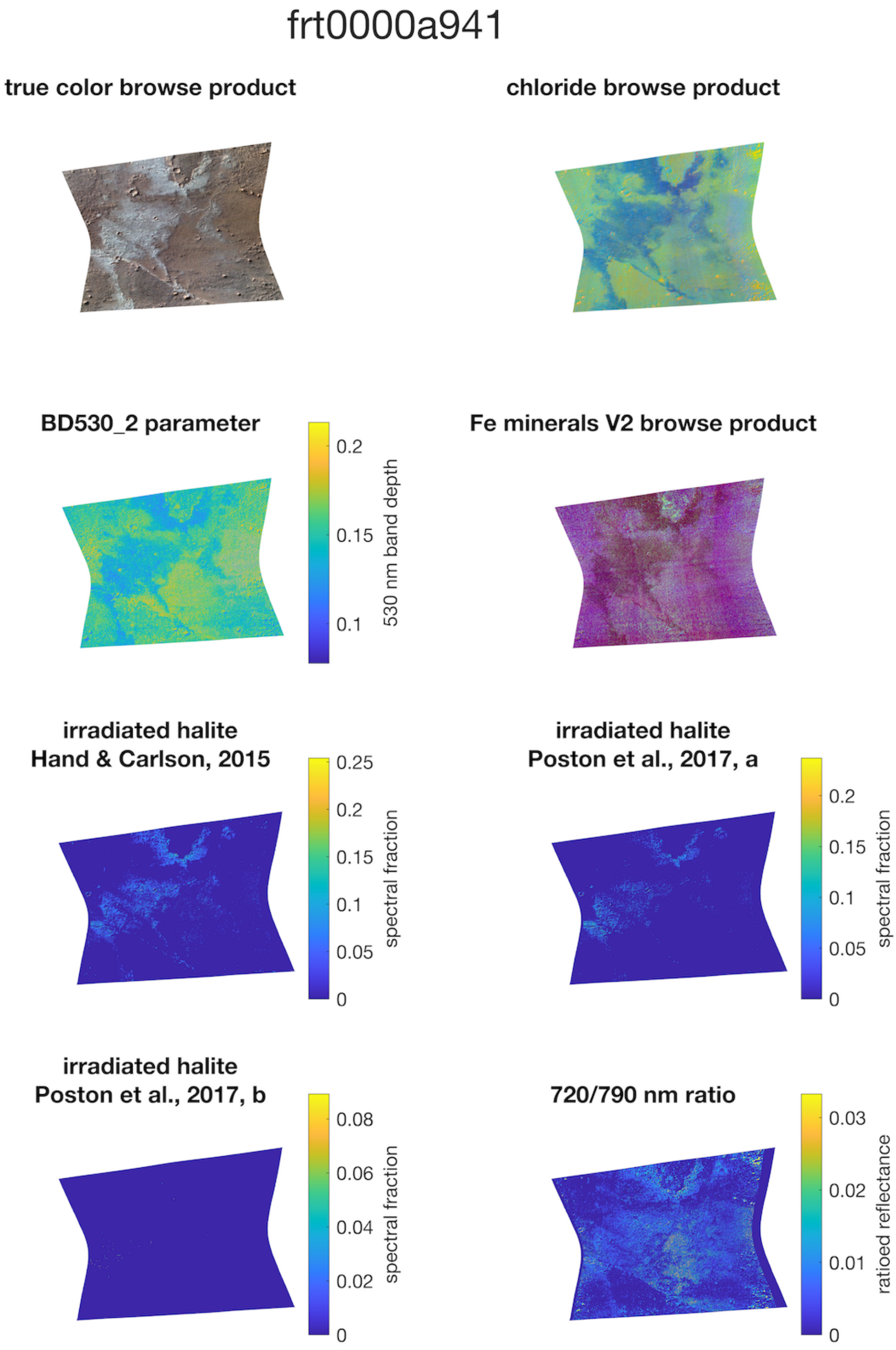
frt0000a941
true color browse product
chloride browse product
BD530_2 parameter
0.2
0.15
0.1
530 nm band depth
Fe minerals V2 browse product
irradiated halite
Hand & Carlson, 2015
0.25
0.2
0.15
0.1
0.05
0
spectral fraction
irradiated halite
Poston et al., 2017, a
0.2
0.15
0.1
0.05
0
spectral fraction
irradiated halite
Poston et al., 2017, b
0.08
0.06
0.04
0.02
0
spectral fraction
720/790 nm ratio
0.03
0.02
0.01
0
ratioed reflectance

frt0000abc2

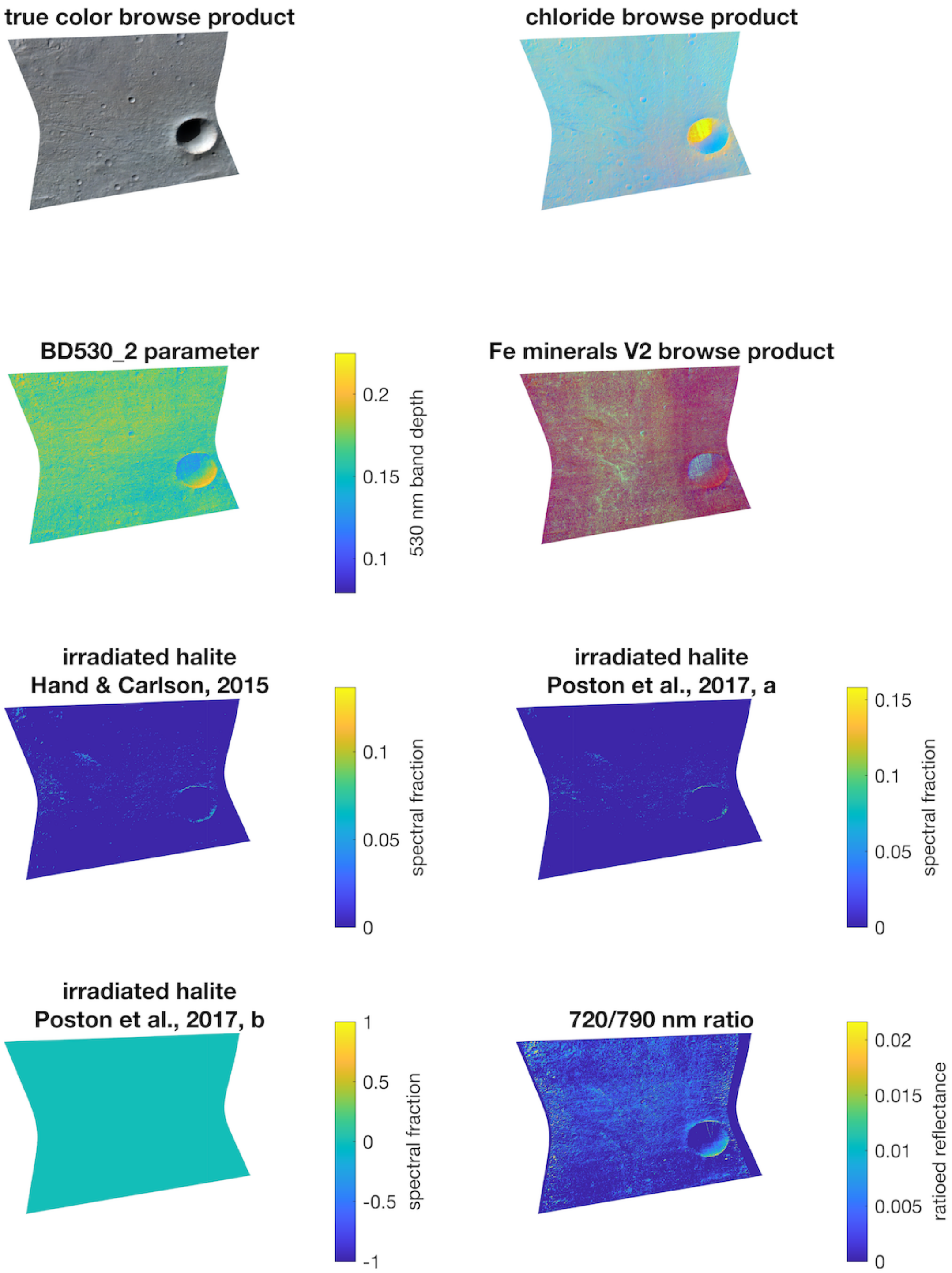

frt0000ad32

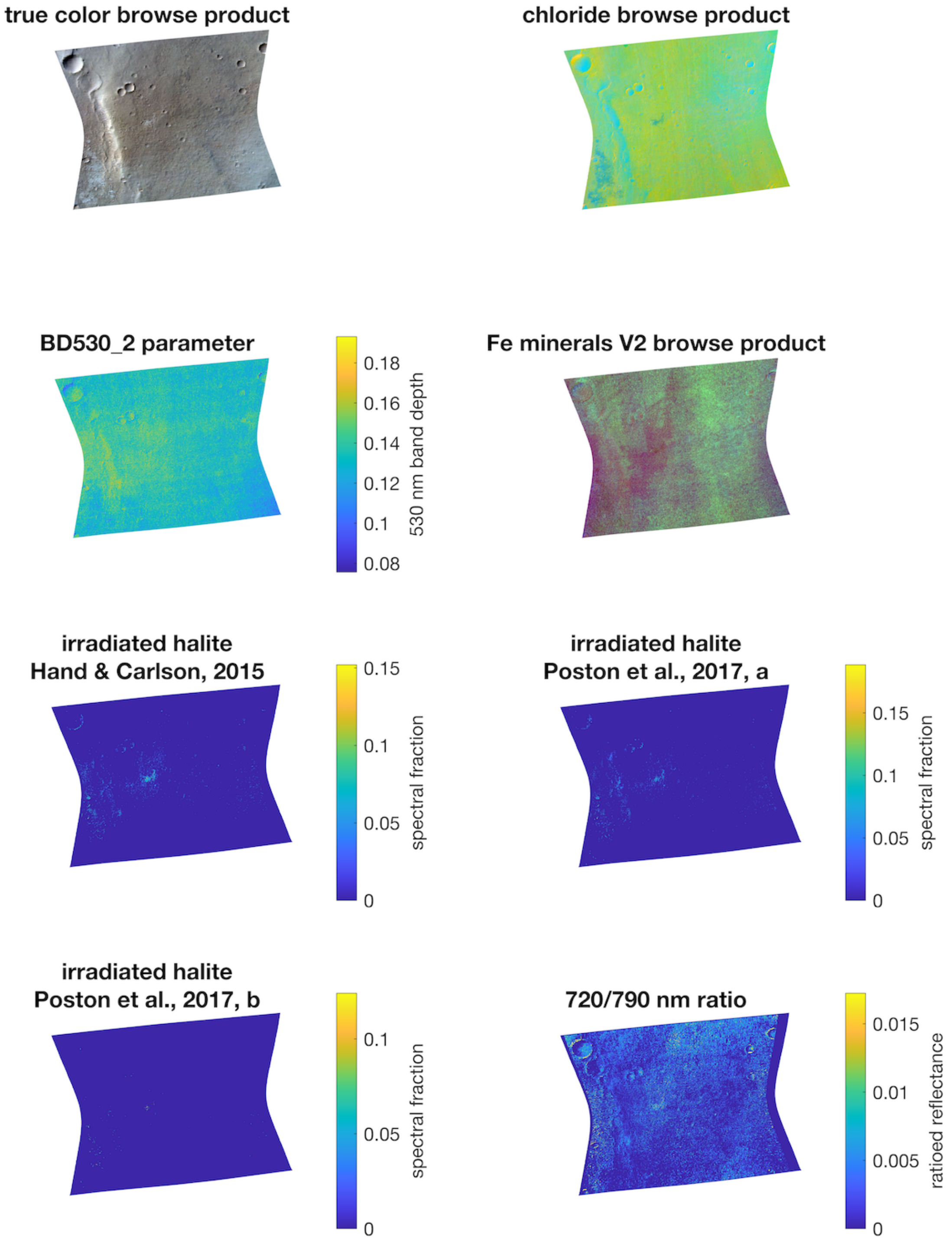

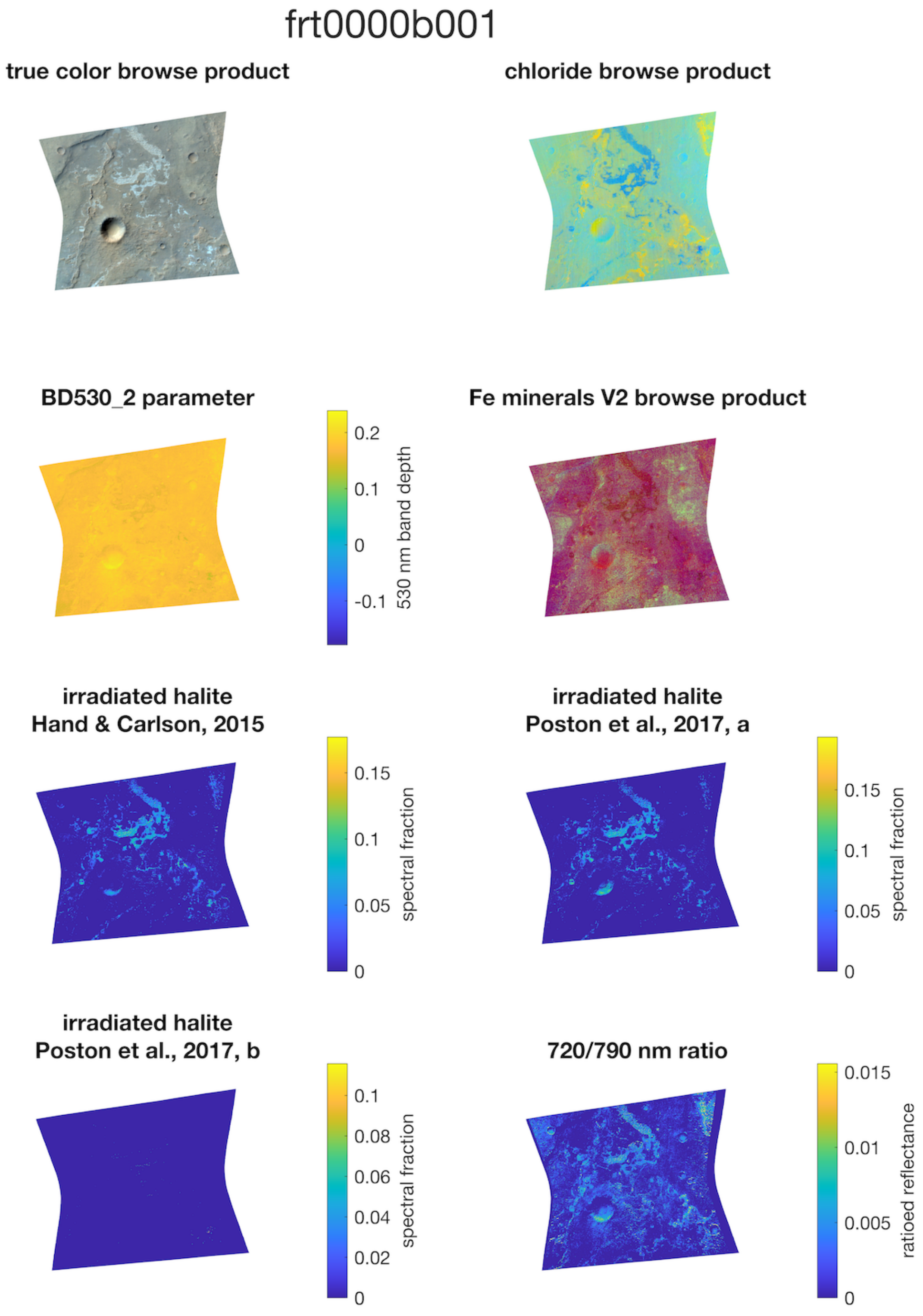
frt0000b001
true color browse product
chloride browse product
BD530_2 parameter
0.2
0.1
0
-0.1
530 nm band depth
Fe minerals V2 browse product
irradiated halite
Hand & Carlson, 2015
0.15
0.1
0.05
0
spectral fraction
irradiated halite
Poston et al., 2017, a
0.15
0.1
0.05
0
spectral fraction
irradiated halite
Poston et al., 2017, b
0.1
0.08
0.06
0.04
0.02
0
spectral fraction
720/790 nm ratio
0.015
0.01
0.005
0
ratioed reflectance

frt0000b1bd

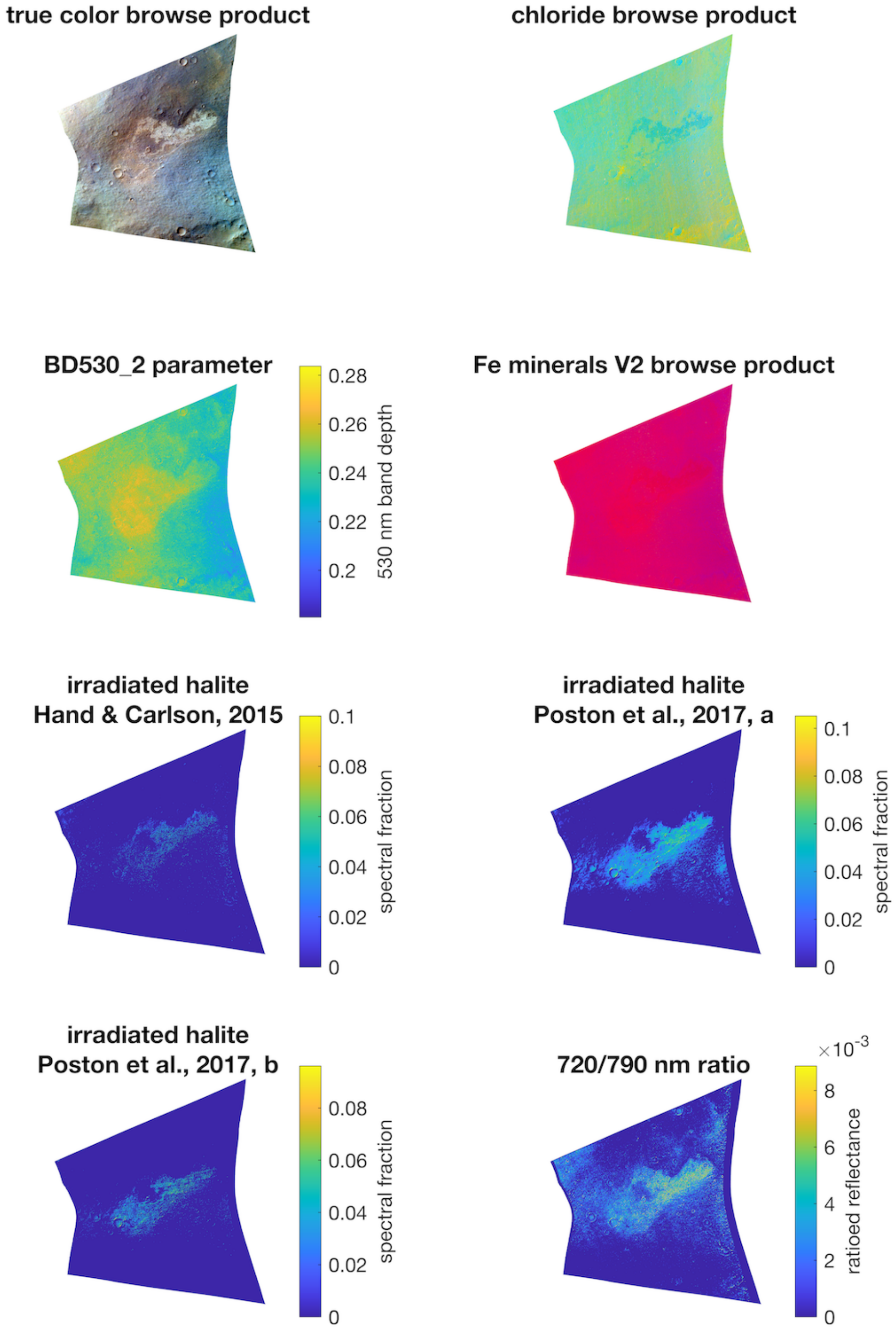

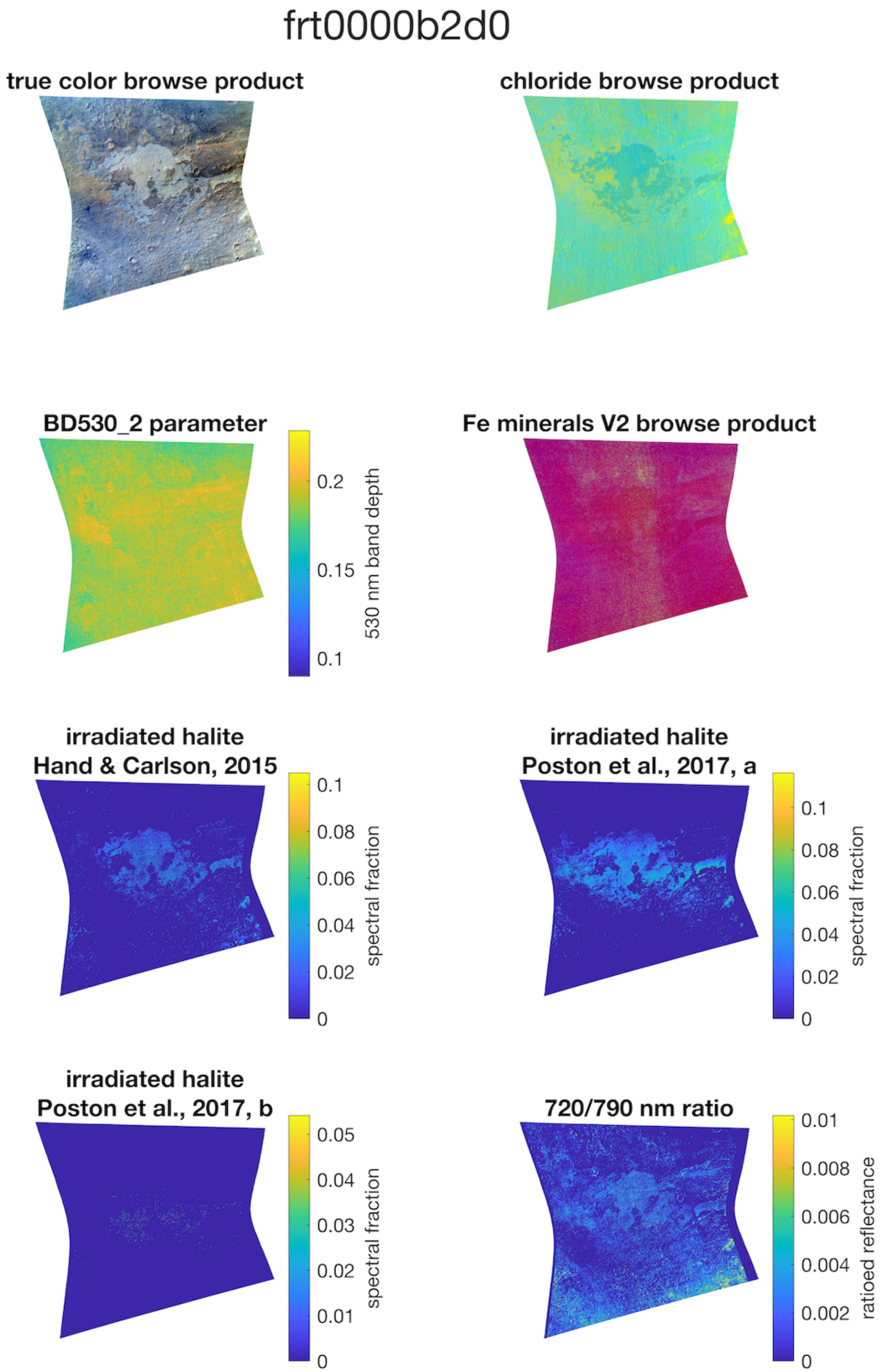

frt0000b2d0
true color browse product
chloride browse product
BD530_2 parameter
0.2
0.15
0.1
530 nm band depth
Fe minerals V2 browse product
irradiated halite
Hand & Carlson, 2015
0.1
0.08
0.06
0.04
0.02
0
spectral fraction
irradiated halite
Poston et al., 2017, a
0.1
0.08
0.06
0.04
0.02
0
spectral fraction
irradiated halite
Poston et al., 2017, b
0.05
0.04
0.03
0.02
0.01
0
spectral fraction
720/790 nm ratio
0.01
0.008
0.006
0.004
0.002
0
ratioed reflectance

frt0000b4bf

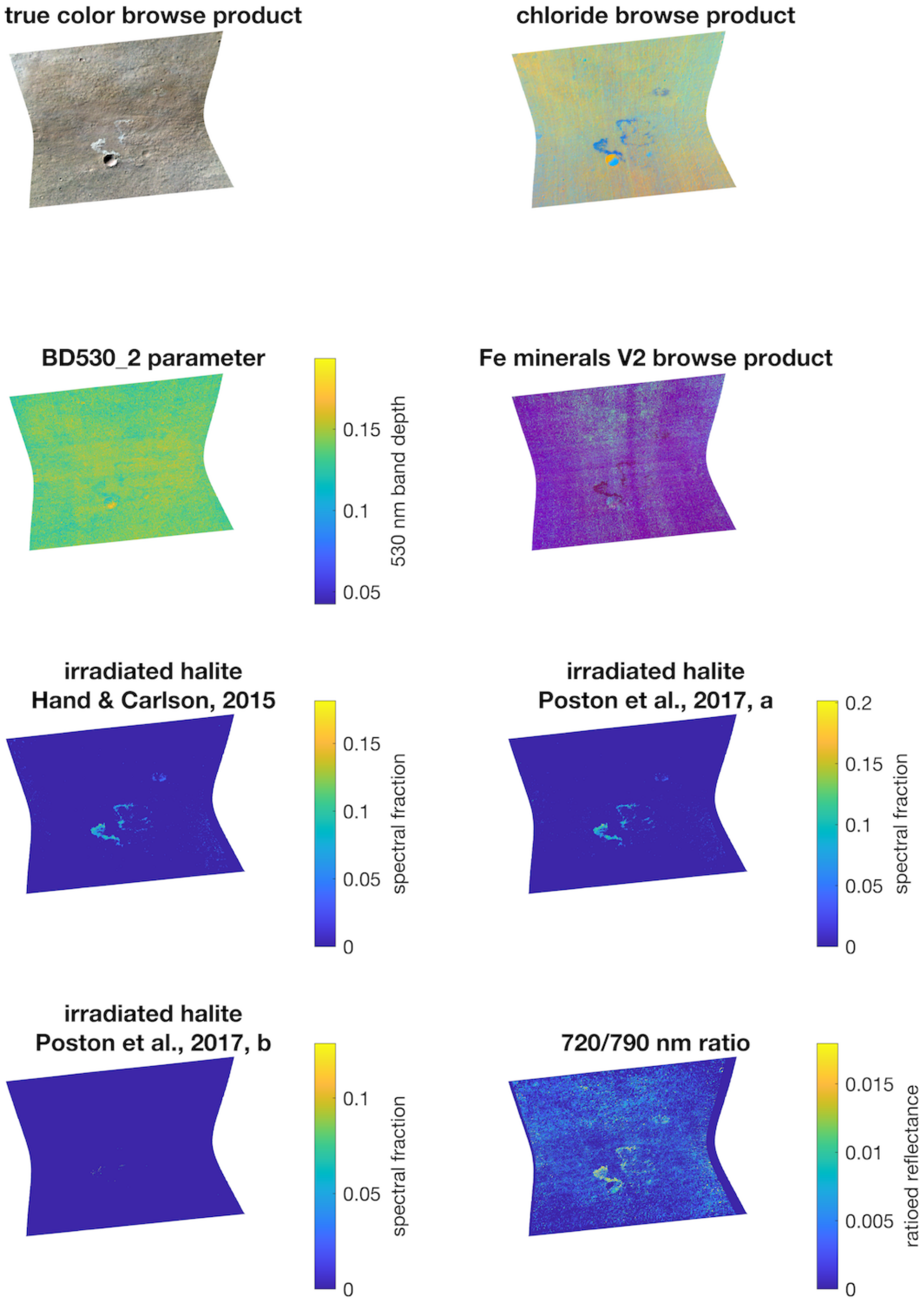

frt0000b60b

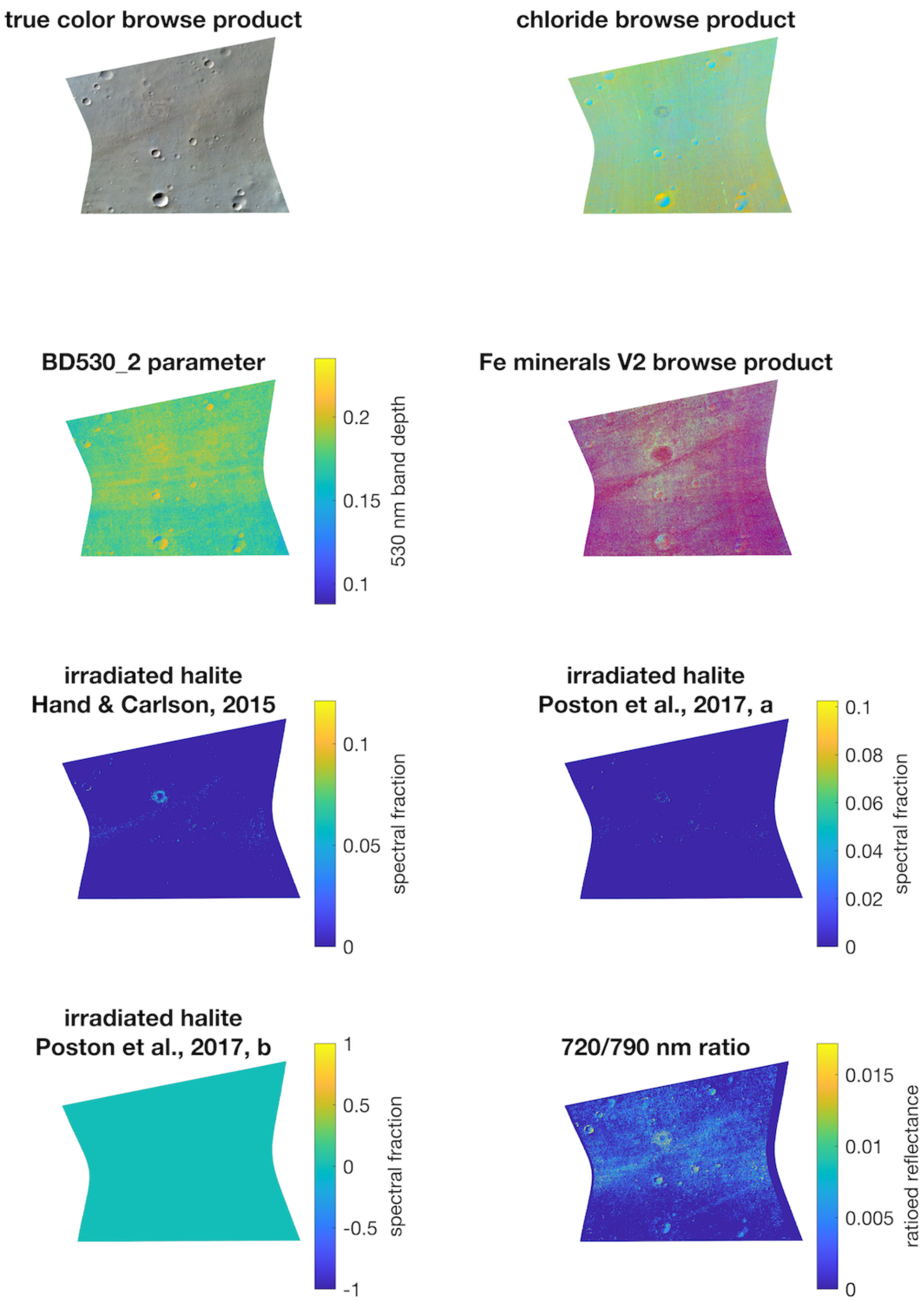

frt0000b694

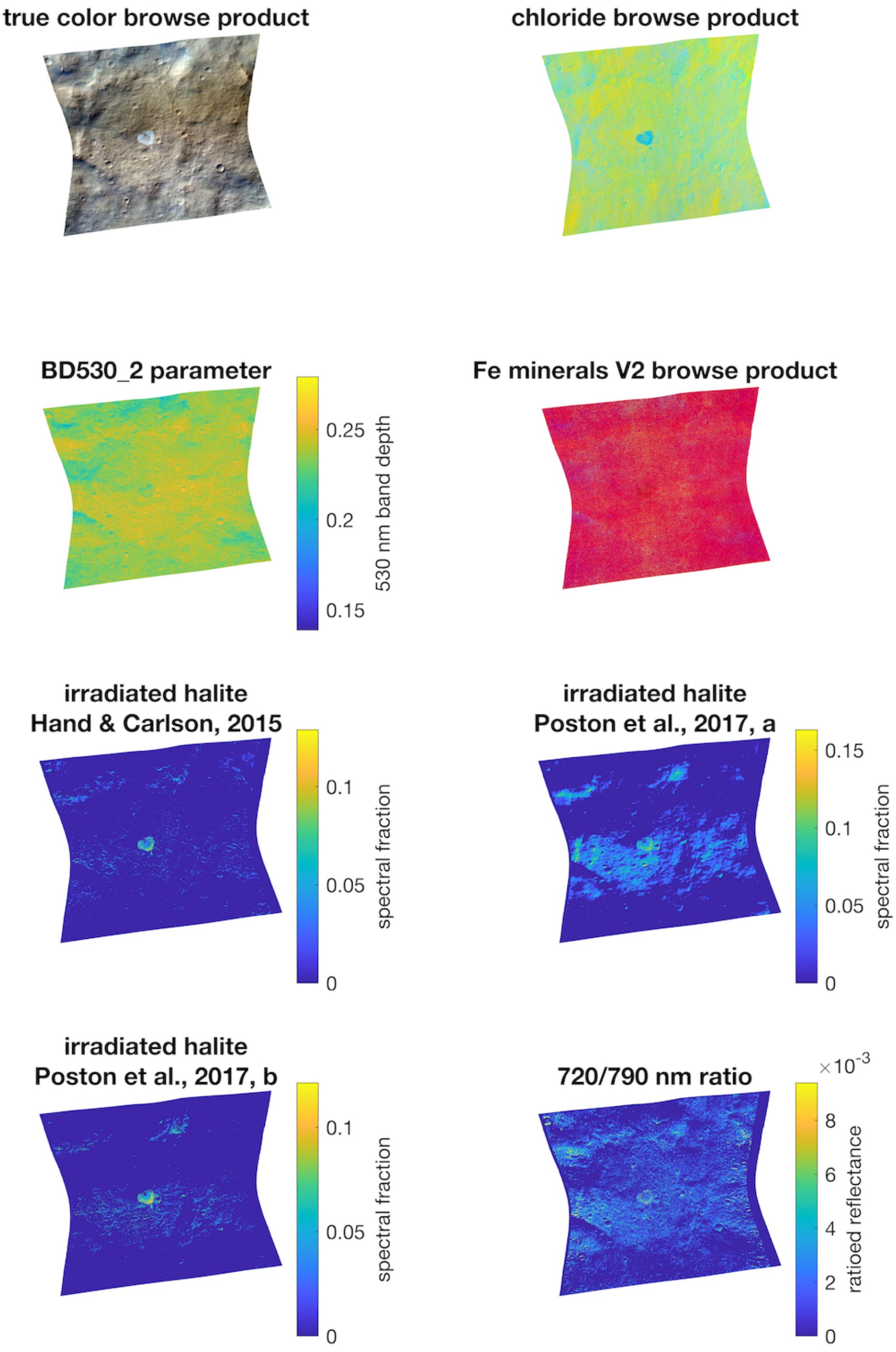

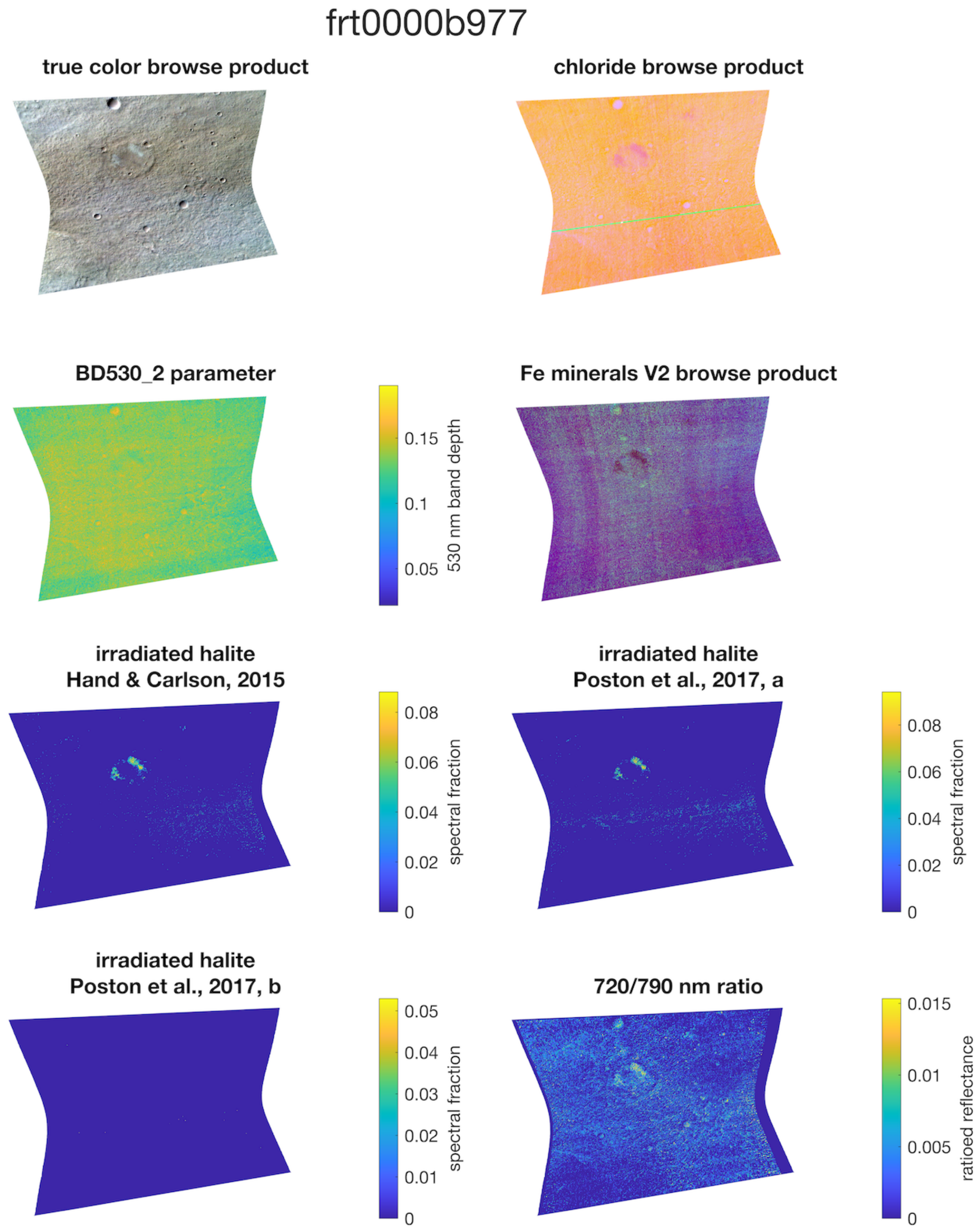

frt0000b977
true color browse product
chloride browse product
BD530_2 parameter
530 nm band depth
0.15
0.1
0.05
Fe minerals V2 browse product
irradiated halite
Hand & Carlson, 2015
spectral fraction
0.08
0.06
0.04
0.02
0
irradiated halite
Poston et al., 2017, a
spectral fraction
0.08
0.06
0.04
0.02
0
irradiated halite
Poston et al., 2017, b
spectral fraction
0.05
0.04
0.03
0.02
0.01
0
720/790 nm ratio
ratioed reflectance
0.015
0.01
0.005
0

frt0000c0af

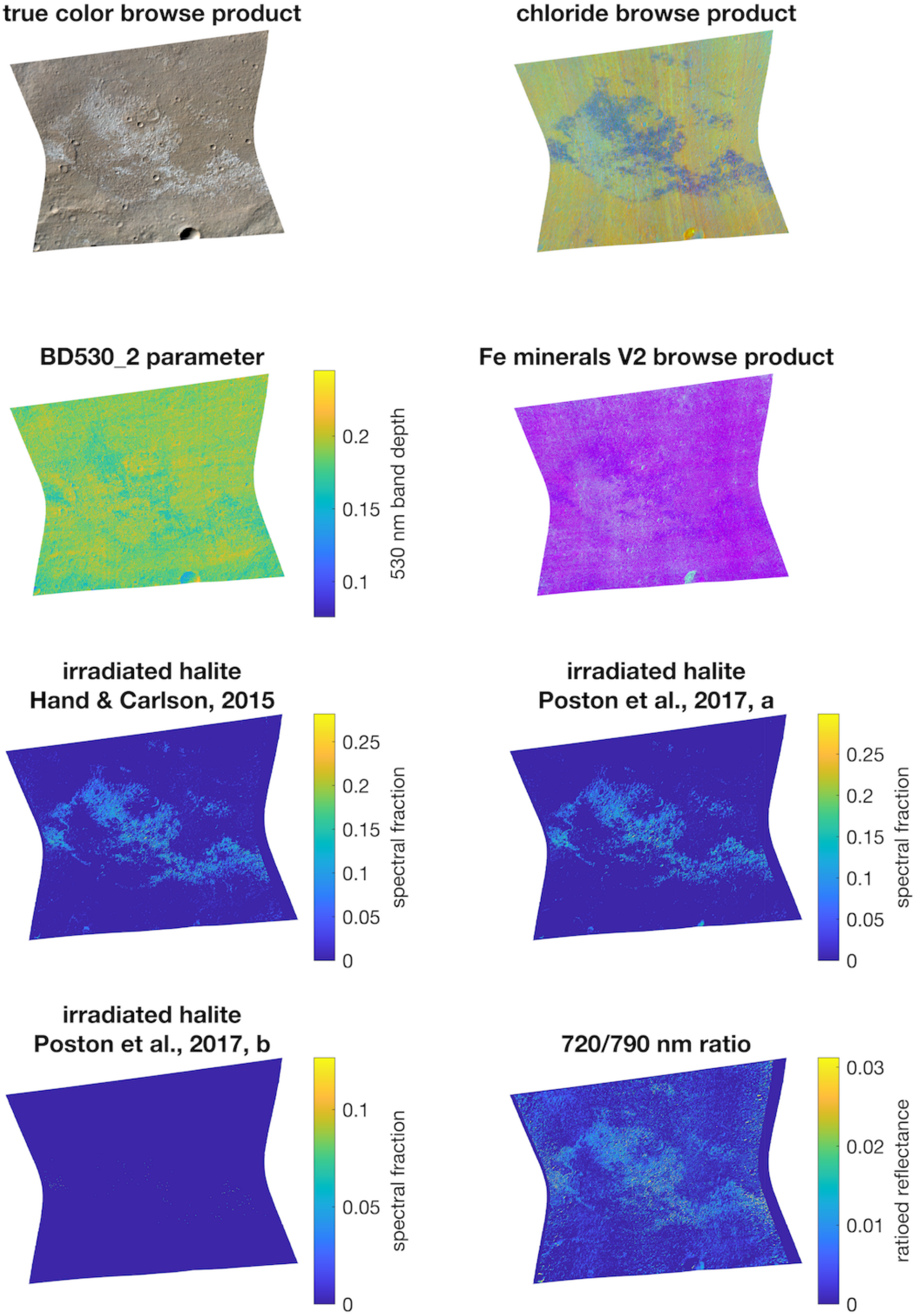

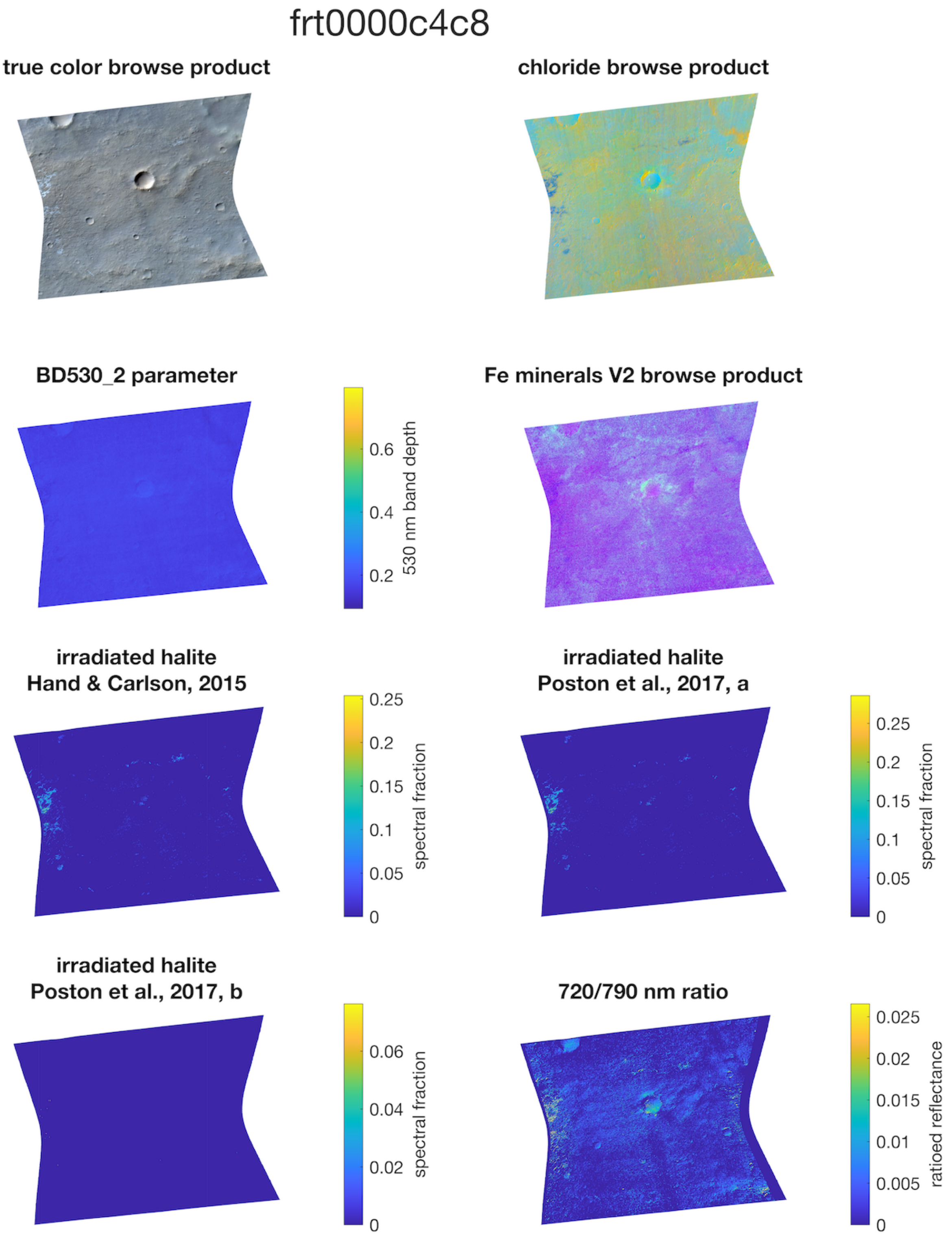
frt0000c4c8
true color browse product
chloride browse product
BD530_2 parameter
0.6
0.4
0.2
530 nm band depth
Fe minerals V2 browse product
irradiated halite
Hand & Carlson, 2015
0.25
0.2
0.15
0.1
0.05
0
spectral fraction
irradiated halite
Poston et al., 2017, a
0.25
0.2
0.15
0.1
0.05
0
spectral fraction
irradiated halite
Poston et al., 2017, b
0.06
0.04
0.02
0
spectral fraction
720/790 nm ratio
0.025
0.02
0.015
0.01
0.005
0
ratioed reflectance

frt0000c08d

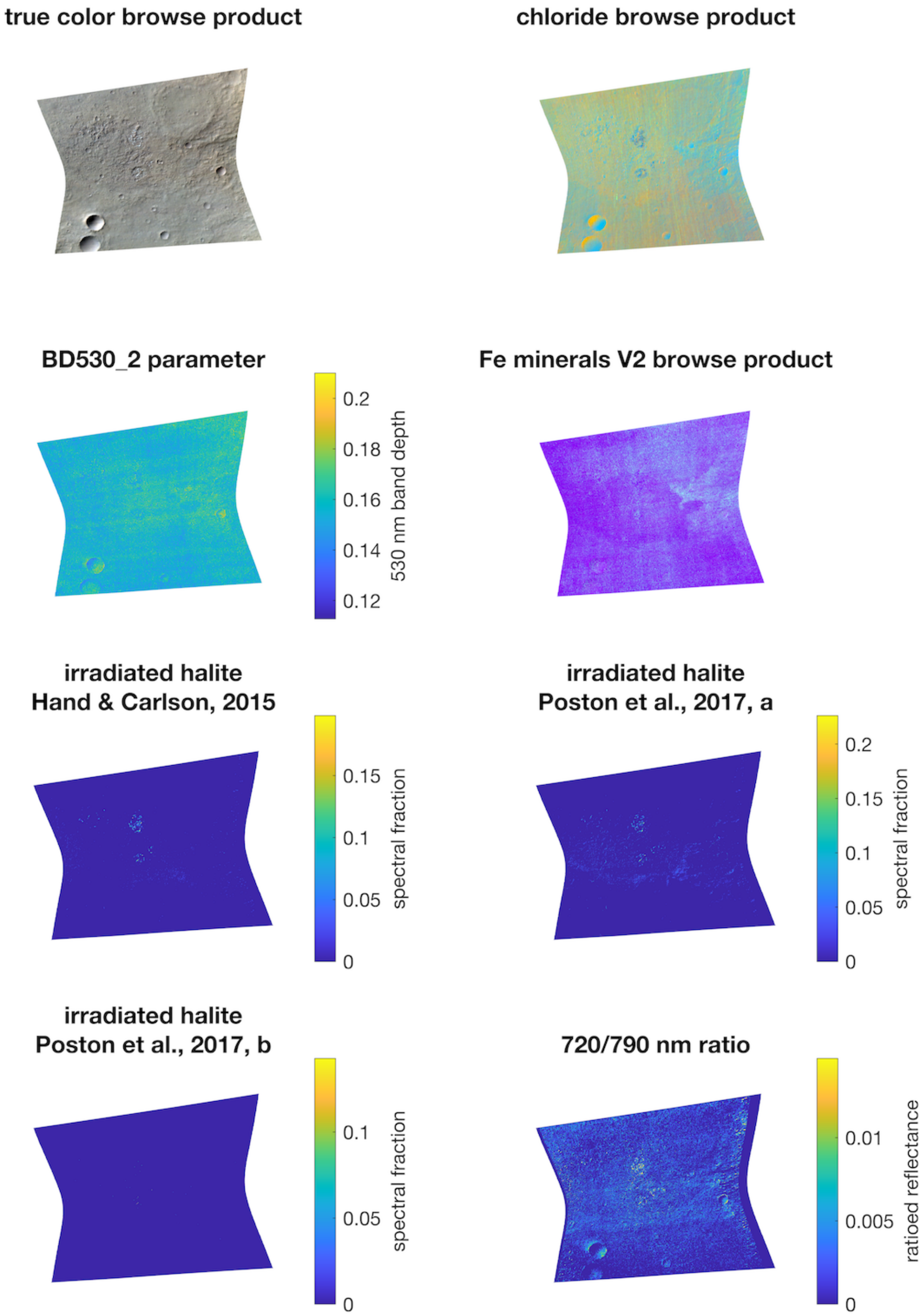

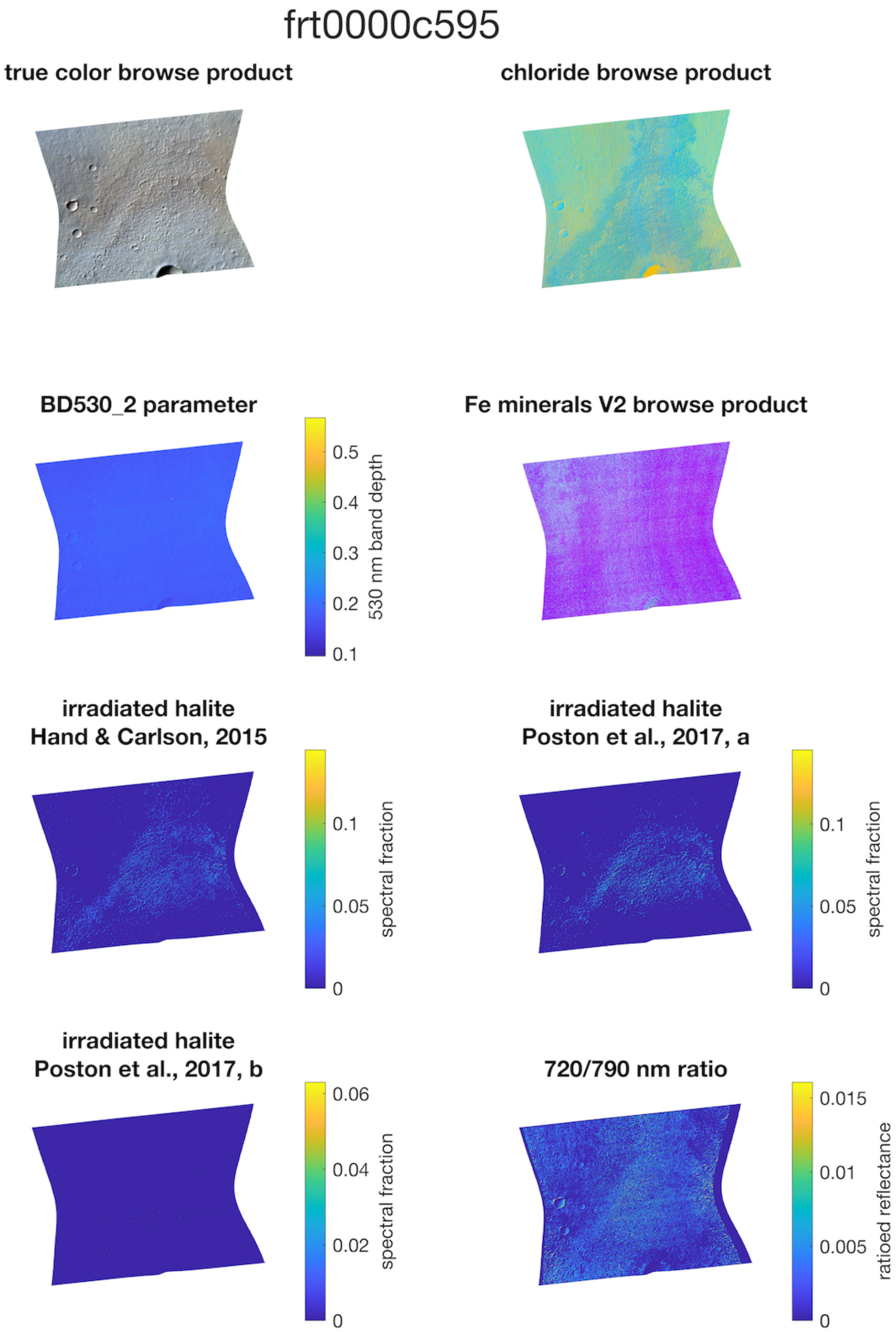

frt0000c595
true color browse product
chloride browse product
BD530_2 parameter
0.5
0.4
0.3
0.2
0.1
530 nm band depth
Fe minerals V2 browse product
irradiated halite
Hand & Carlson, 2015
0.1
0.05
0
spectral fraction
irradiated halite
Poston et al., 2017, a
0.1
0.05
0
spectral fraction
irradiated halite
Poston et al., 2017, b
0.06
0.04
0.02
0
spectral fraction
720/790 nm ratio
0.015
0.01
0.005
0
ratioed reflectance

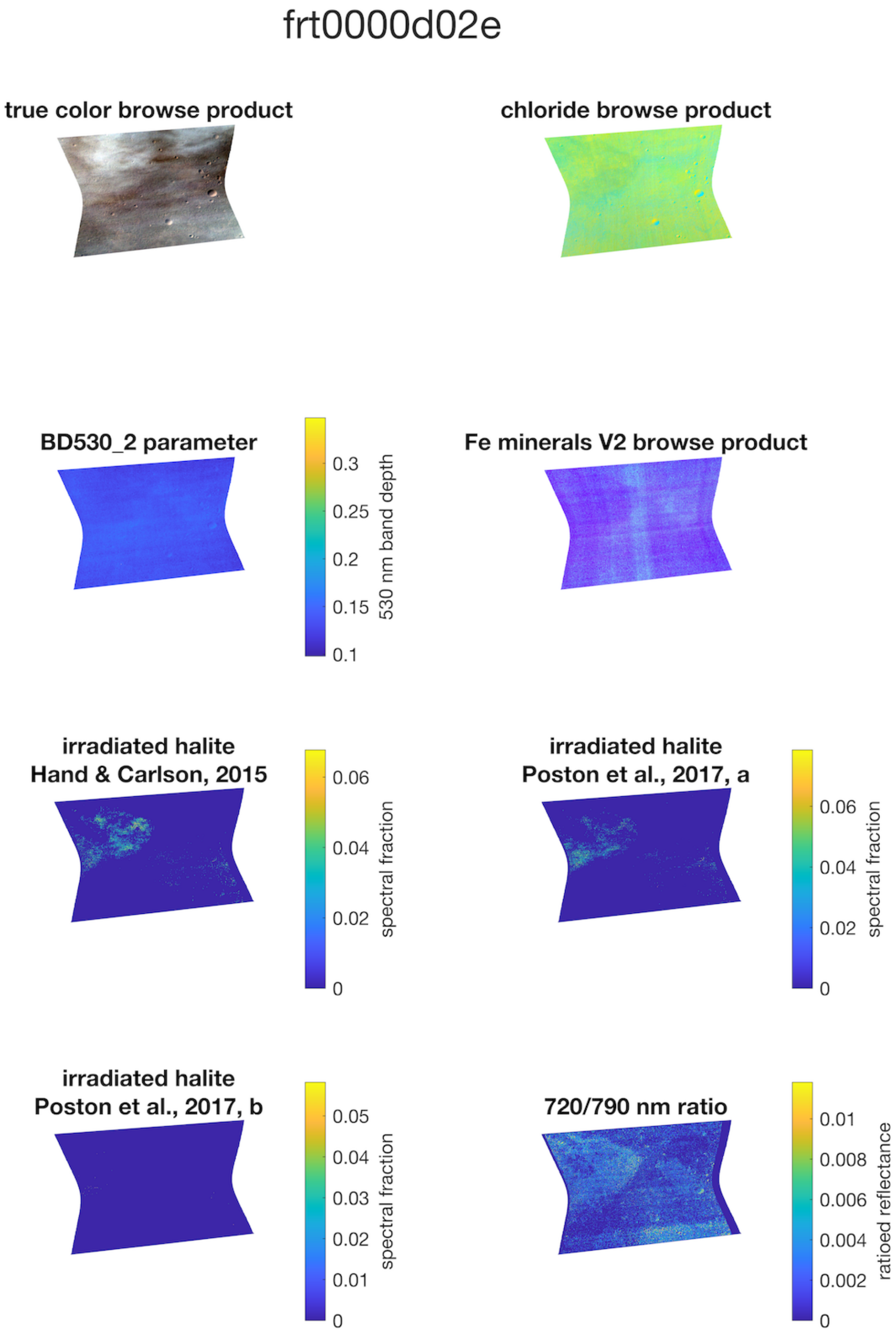
frt0000d02e
true color browse product
chloride browse product
BD530_2 parameter
0.3
0.25
0.2
0.15
0.1
530 nm band depth
Fe minerals V2 browse product
irradiated halite
Hand & Carlson, 2015
0.06
0.04
0.02
0
spectral fraction
irradiated halite
Poston et al., 2017, a
0.06
0.04
0.02
0
spectral fraction
irradiated halite
Poston et al., 2017, b
0.05
0.04
0.03
0.02
0.01
0
spectral fraction
720/790 nm ratio
0.01
0.008
0.006
0.004
0.002
0
ratioed reflectance

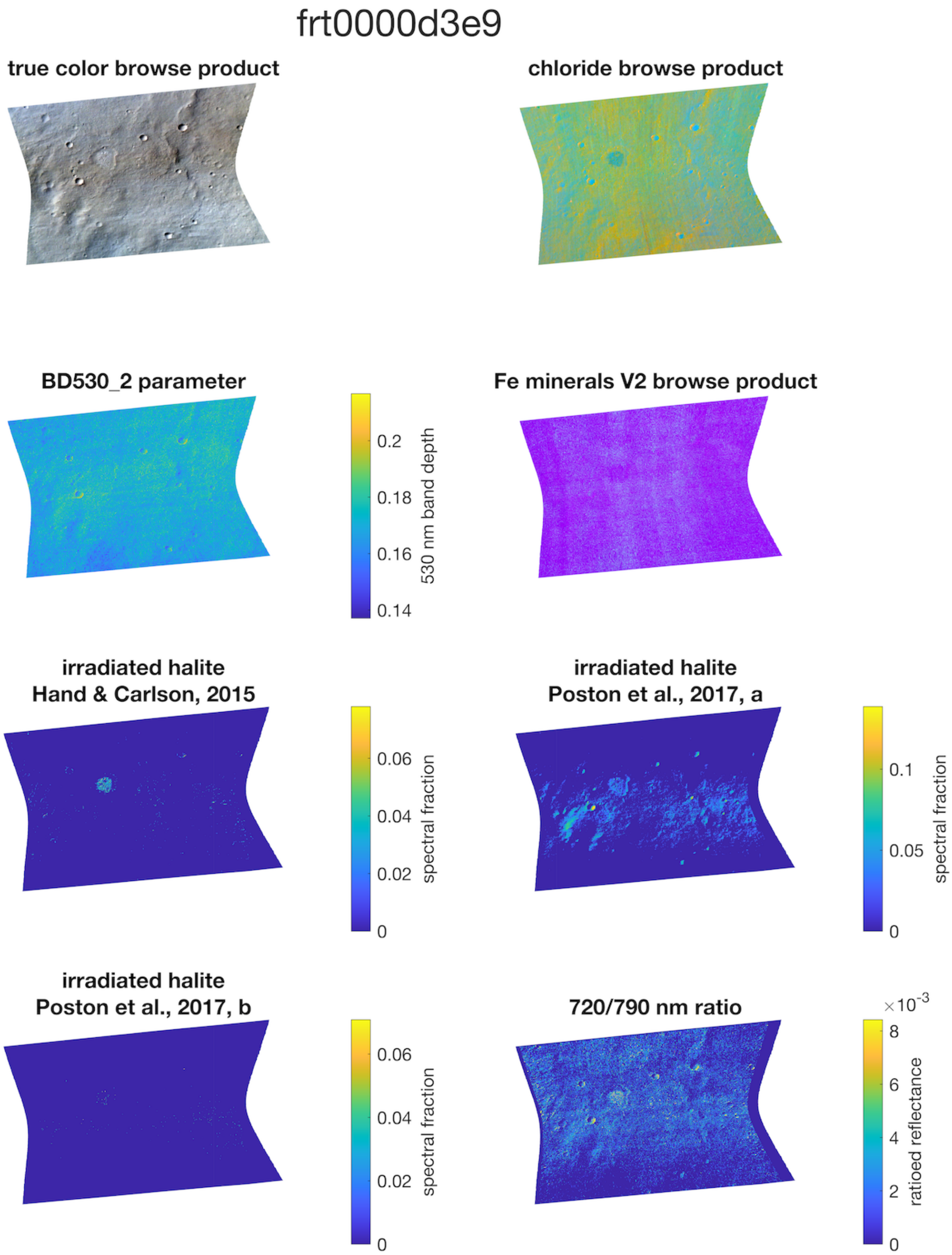
frt0000d3e9
true color browse product
chloride browse product
BD530_2 parameter
0.2
0.18
0.16
0.14
530 nm band depth
Fe minerals V2 browse product
irradiated halite
Hand & Carlson, 2015
0.06
0.04
0.02
0
spectral fraction
irradiated halite
Poston et al., 2017, a
0.1
0.05
0
spectral fraction
irradiated halite
Poston et al., 2017, b
0.06
0.04
0.02
0
spectral fraction
720/790 nm ratio
×10-3
8
6
4
2
0
ratioed reflectance

frt0000d6b7

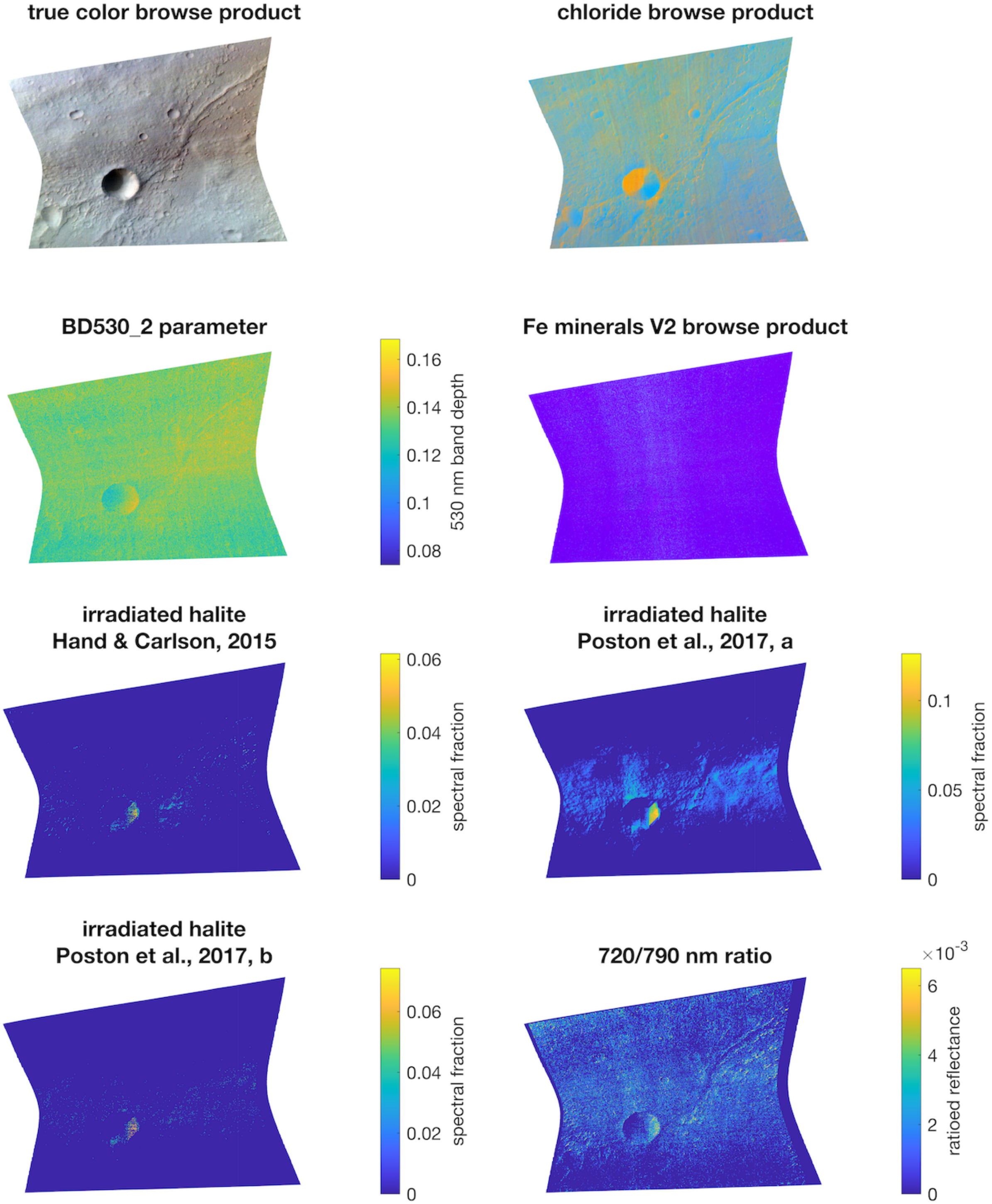

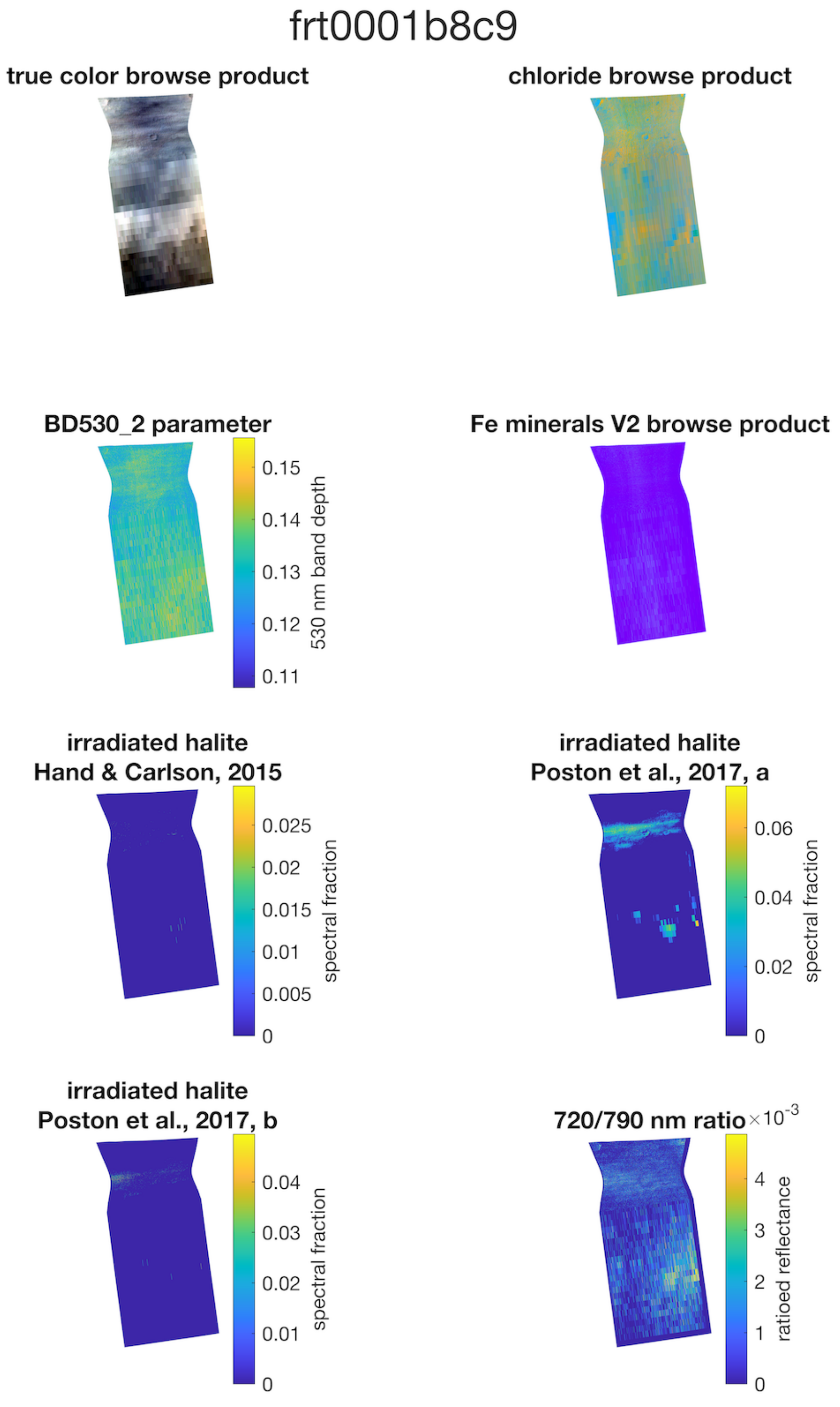
frt0001b8c9
true color browse product
chloride browse product
BD530_2 parameter
530 nm band depth
0.15
0.14
0.13
0.12
0.11
Fe minerals V2 browse product
irradiated halite
Hand & Carlson, 2015
spectral fraction
0.025
0.02
0.015
0.01
0.005
0
irradiated halite
Poston et al., 2017, a
spectral fraction
0.06
0.04
0.02
0
irradiated halite
Poston et al., 2017, b
spectral fraction
0.04
0.03
0.02
0.01
0
720/790 nm ratio
×10^-3
ratioed reflectance
4
3
2
1
0

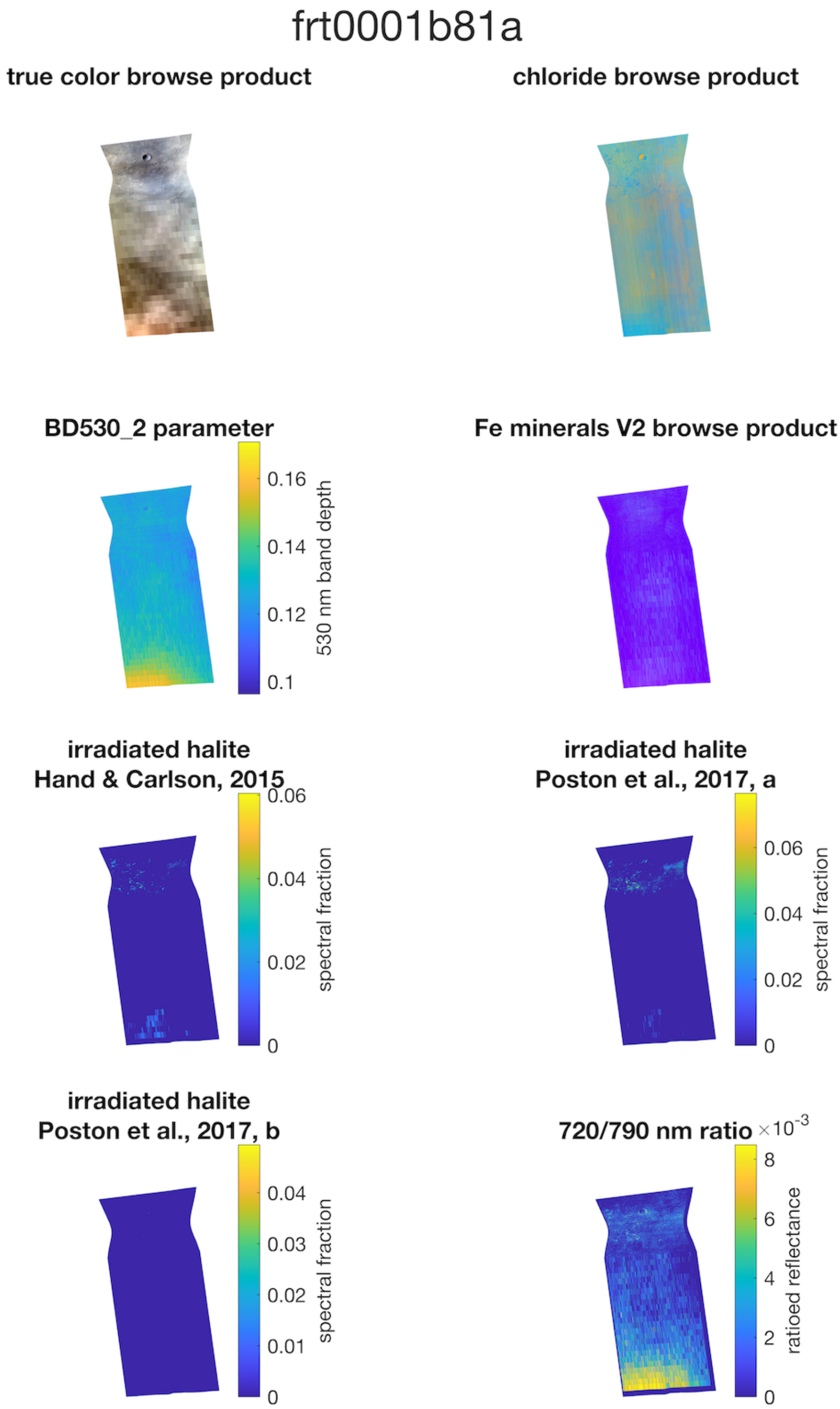
frt0001b81a
true color browse product
chloride browse product
BD530_2 parameter
530 nm band depth
0.16
0.14
0.12
0.1
Fe minerals V2 browse product
irradiated halite
Hand & Carlson, 2015
spectral fraction
0.06
0.04
0.02
0
irradiated halite
Poston et al., 2017, a
spectral fraction
0.06
0.04
0.02
0
irradiated halite
Poston et al., 2017, b
spectral fraction
0.04
0.03
0.02
0.01
0
720/790 nm ratio
×10^-3
ratioed reflectance
8
6
4
2
0

frt0001b804

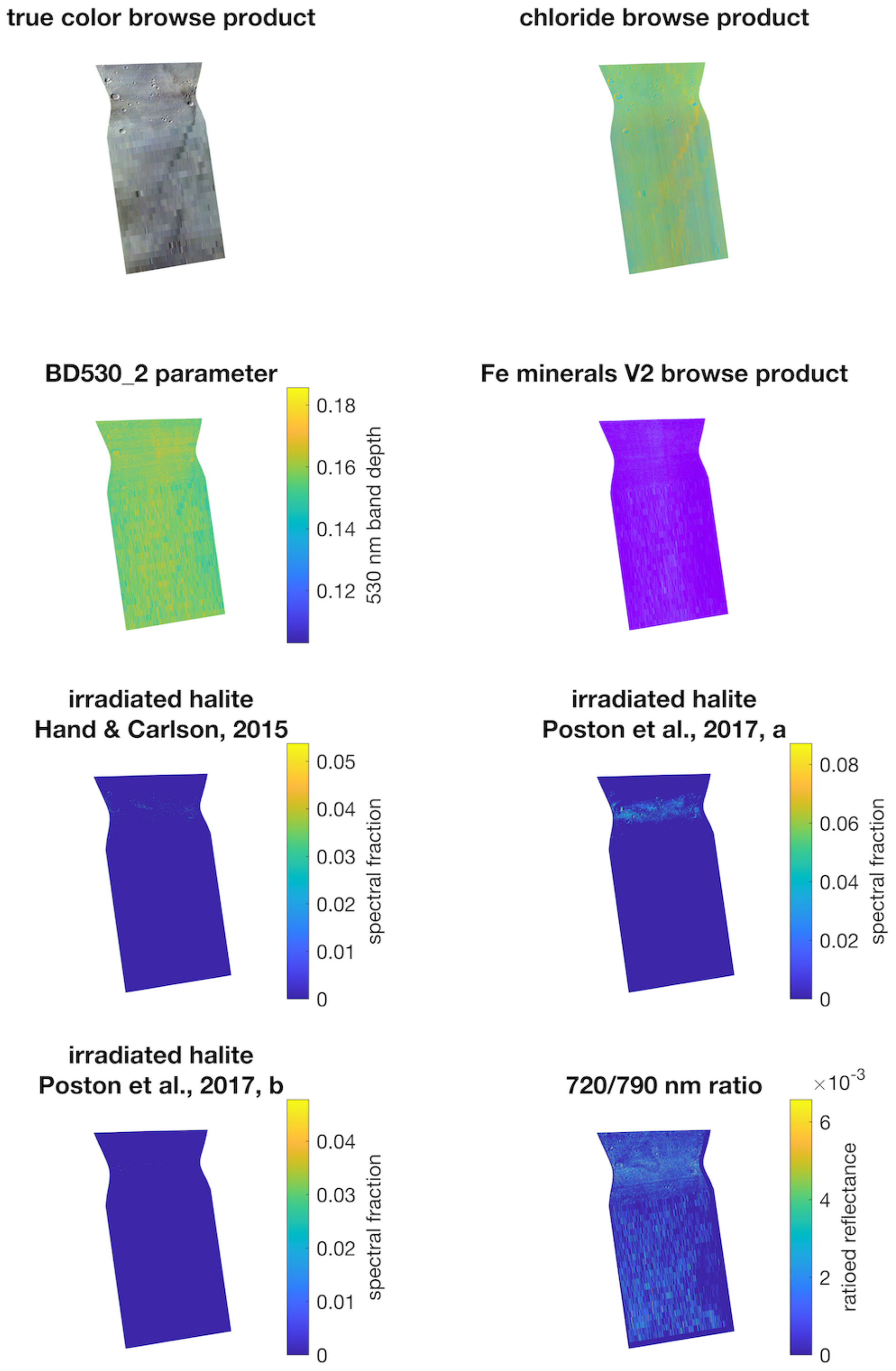

frt0001ec37

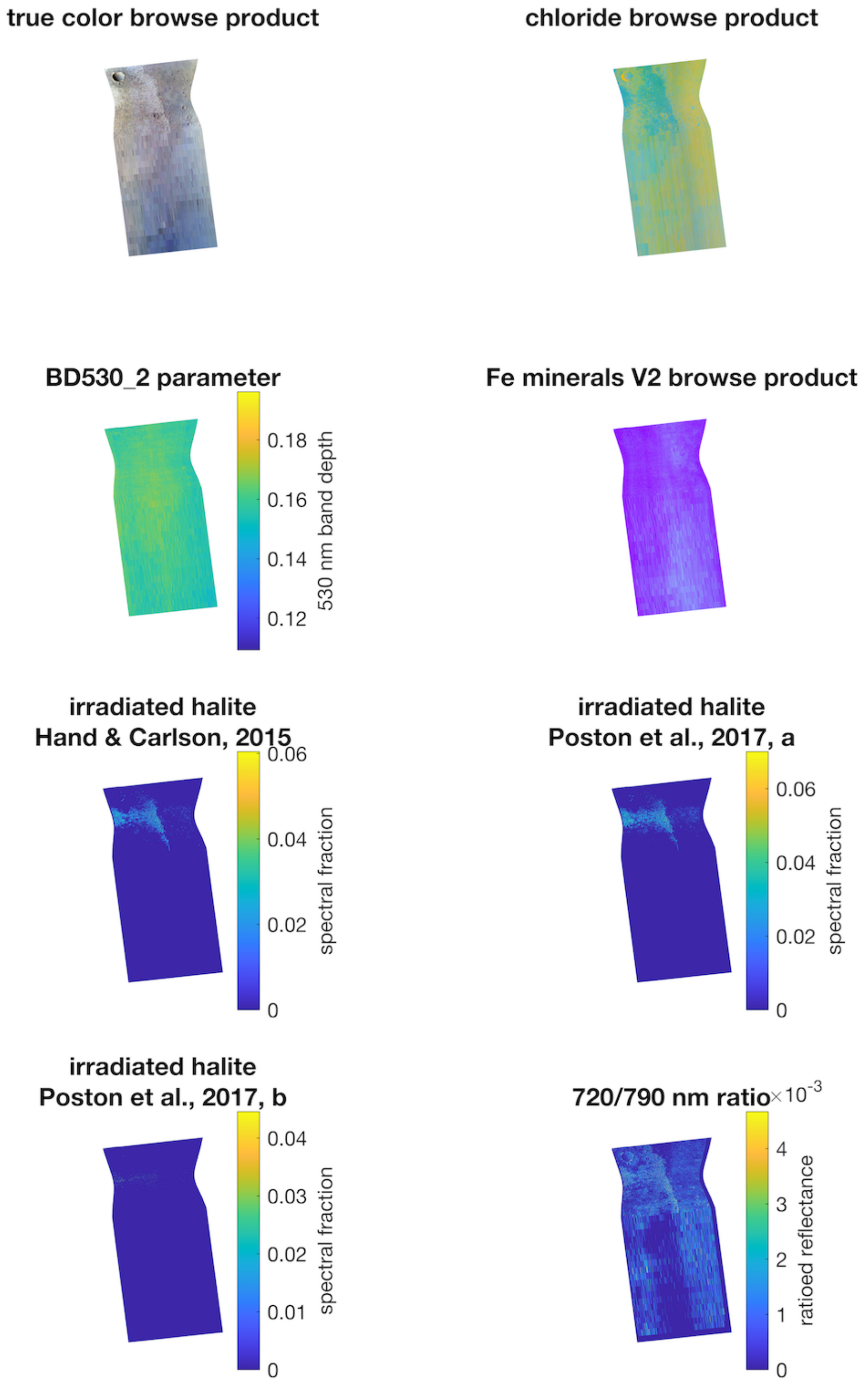

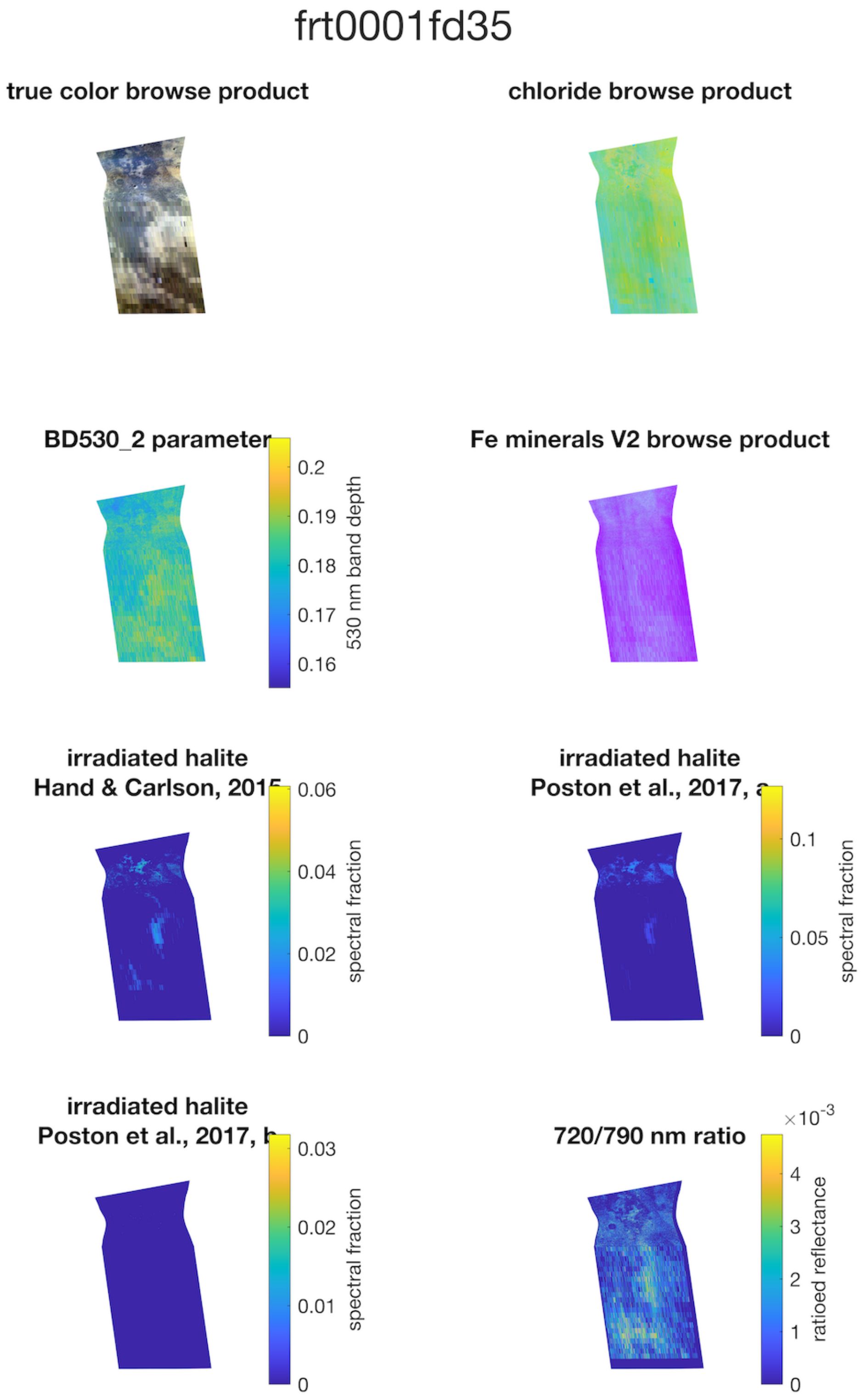

frt0001fd35
true color browse product
chloride browse product
BD530_2 parameter
530 nm band depth
0.2
0.19
0.18
0.17
0.16
Fe minerals V2 browse product
irradiated halite
Hand & Carlson, 2015
spectral fraction
0.06
0.04
0.02
0
irradiated halite
Poston et al., 2017, a
spectral fraction
0.1
0.05
0
irradiated halite
Poston et al., 2017, b
spectral fraction
0.03
0.02
0.01
0
720/790 nm ratio
×10^-3
ratioed reflectance
4
3
2
1
0

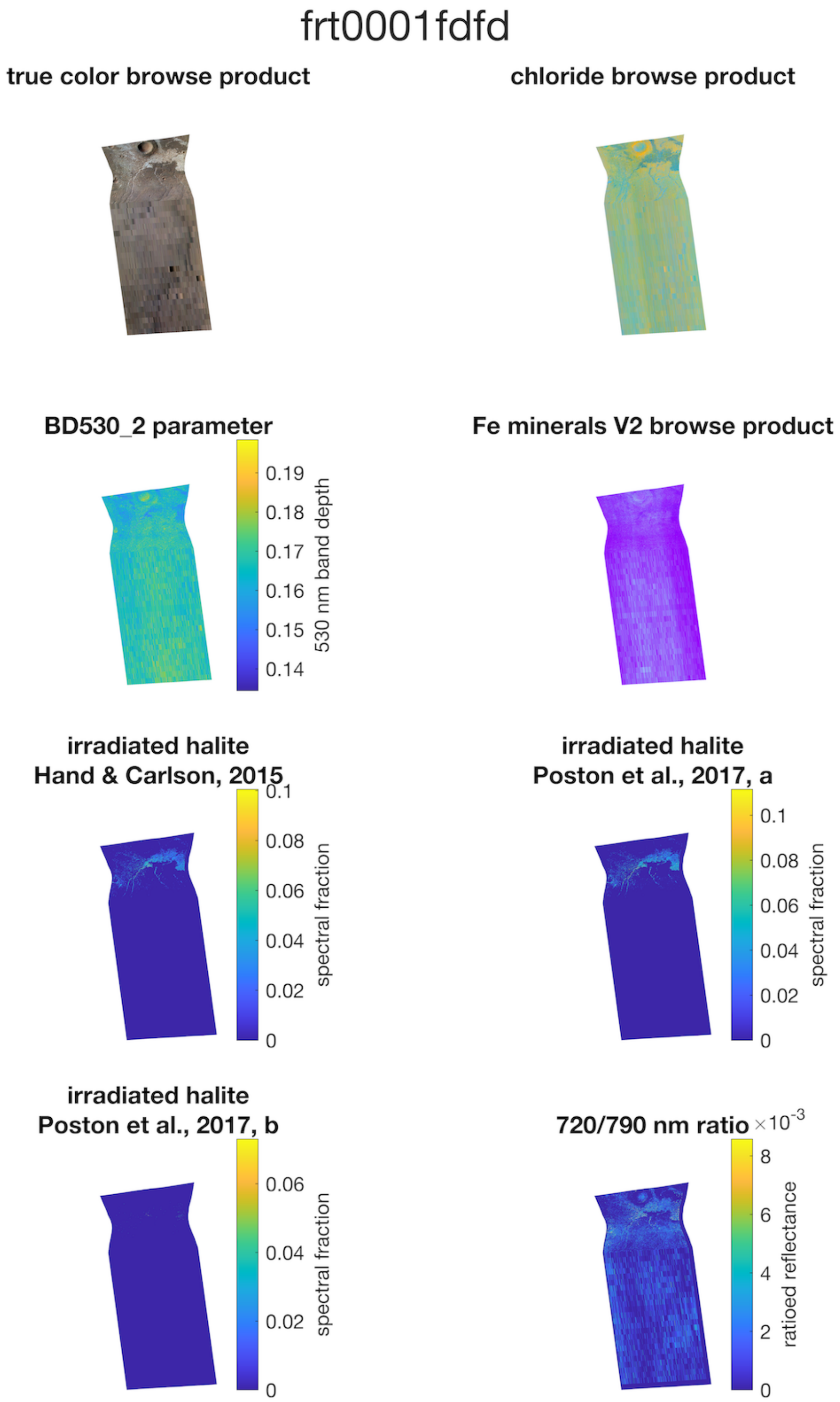
frt0001fdfd
true color browse product
chloride browse product
BD530_2 parameter
0.19
0.18
0.17
0.16
0.15
0.14
530 nm band depth
Fe minerals V2 browse product
irradiated halite
Hand & Carlson, 2015
0.1
0.08
0.06
0.04
0.02
0
spectral fraction
irradiated halite
Poston et al., 2017, a
0.1
0.08
0.06
0.04
0.02
0
spectral fraction
irradiated halite
Poston et al., 2017, b
0.06
0.04
0.02
0
spectral fraction
720/790 nm ratio
×10^-3
8
6
4
2
0
ratioed reflectance

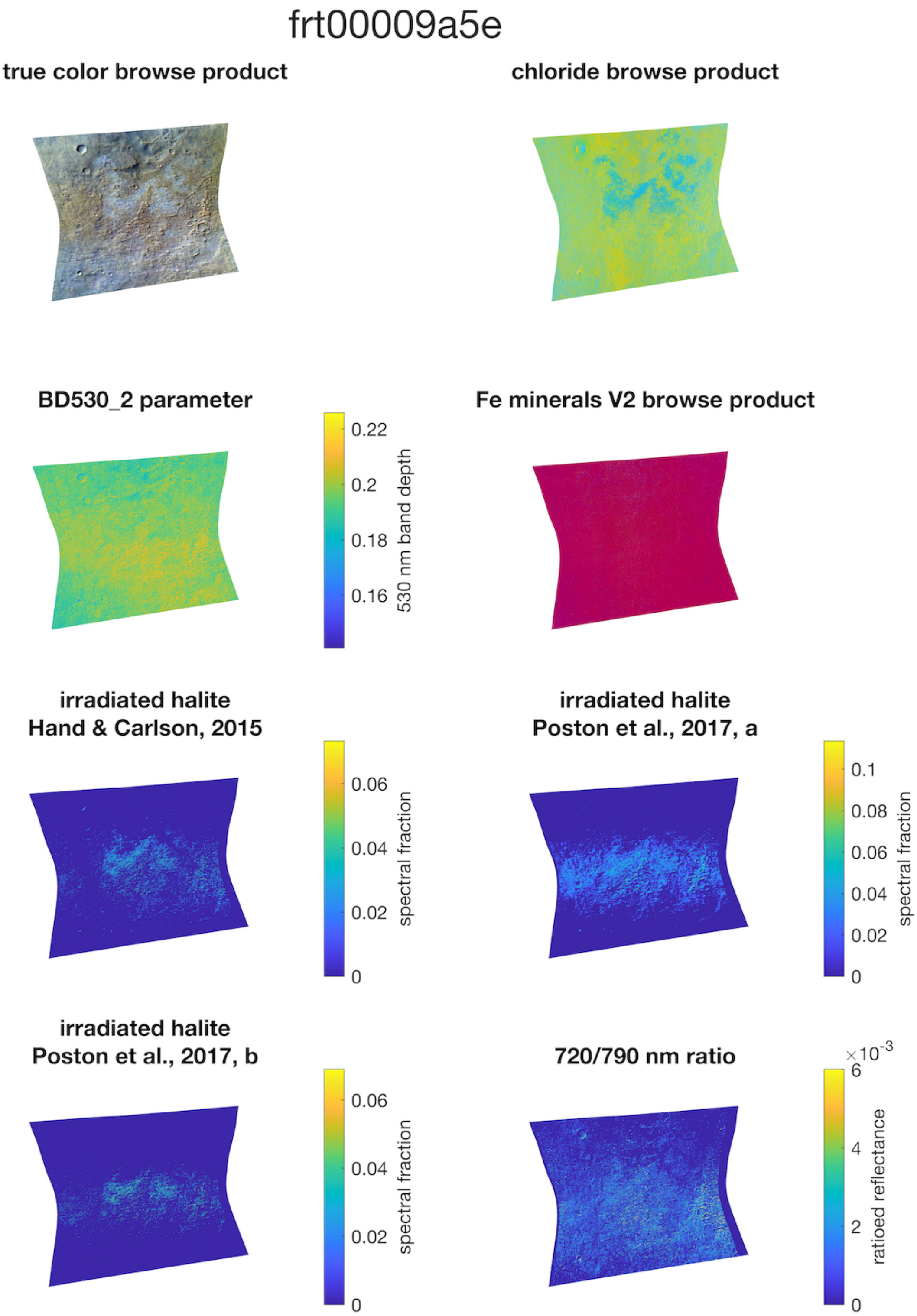
frt00009a5e
true color browse product
chloride browse product
BD530_2 parameter
530 nm band depth
0.22
0.2
0.18
0.16
Fe minerals V2 browse product
irradiated halite
Hand & Carlson, 2015
spectral fraction
0.06
0.04
0.02
0
irradiated halite
Poston et al., 2017, a
spectral fraction
0.1
0.08
0.06
0.04
0.02
0
irradiated halite
Poston et al., 2017, b
spectral fraction
0.06
0.04
0.02
0
720/790 nm ratio
×10^-3
ratioed reflectance
6
4
2
0

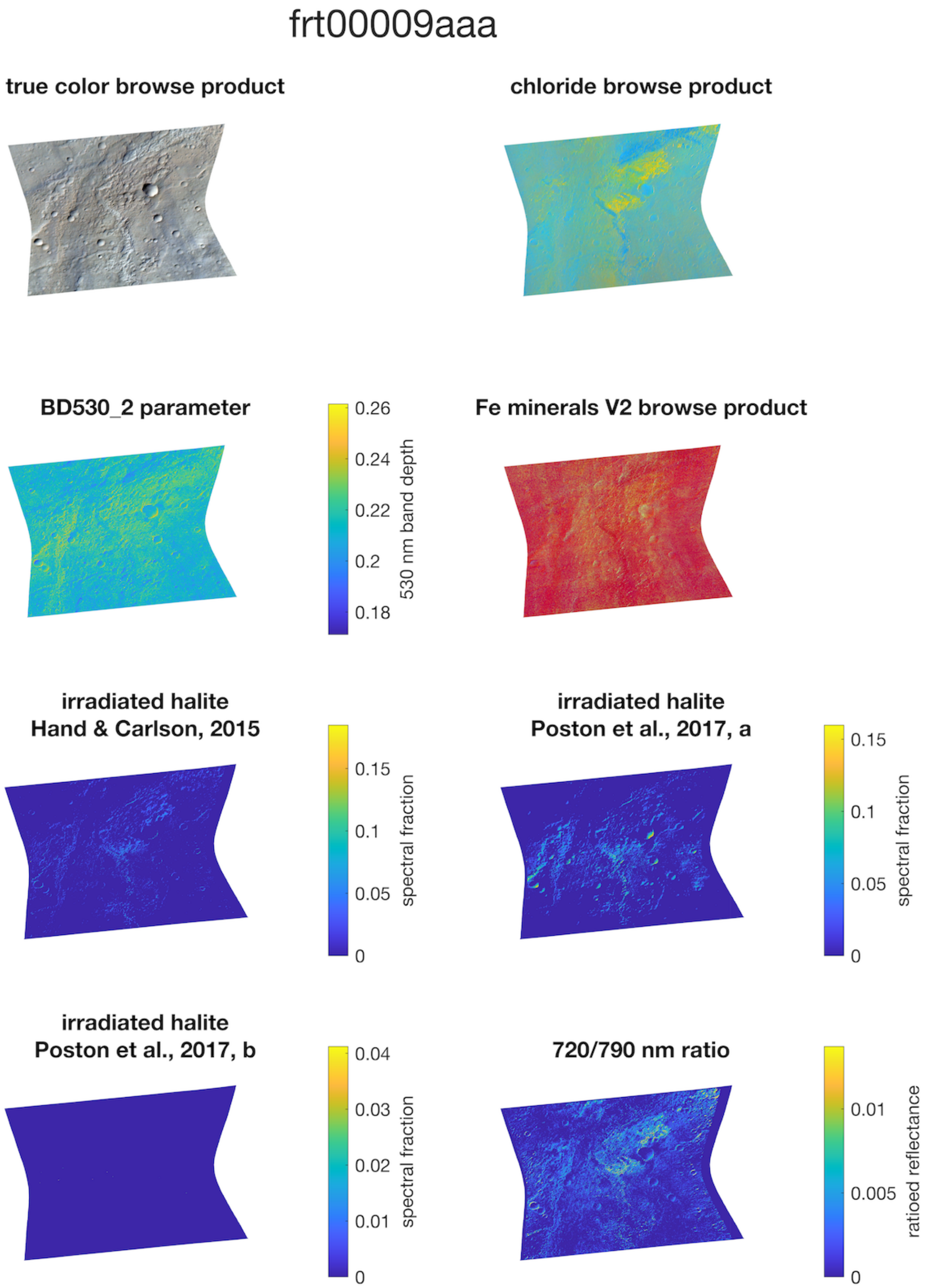
frt00009aaa
true color browse product
chloride browse product
BD530_2 parameter
0.26
0.24
0.22
0.2
0.18
530 nm band depth
Fe minerals V2 browse product
irradiated halite
Hand & Carlson, 2015
0.15
0.1
0.05
0
spectral fraction
irradiated halite
Poston et al., 2017, a
0.15
0.1
0.05
0
spectral fraction
irradiated halite
Poston et al., 2017, b
0.04
0.03
0.02
0.01
0
spectral fraction
720/790 nm ratio
0.01
0.005
0
ratioed reflectance

frt00009ace

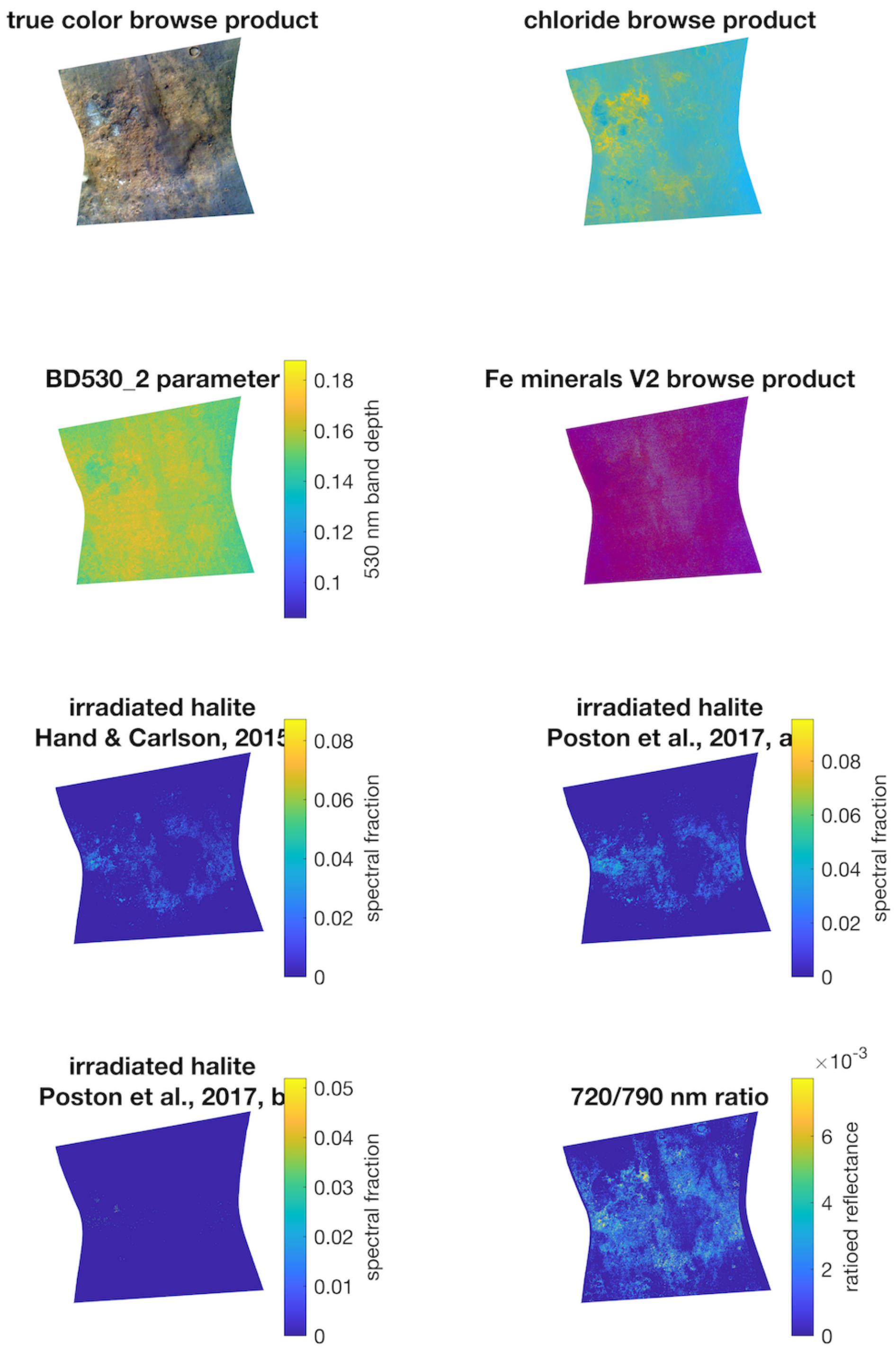

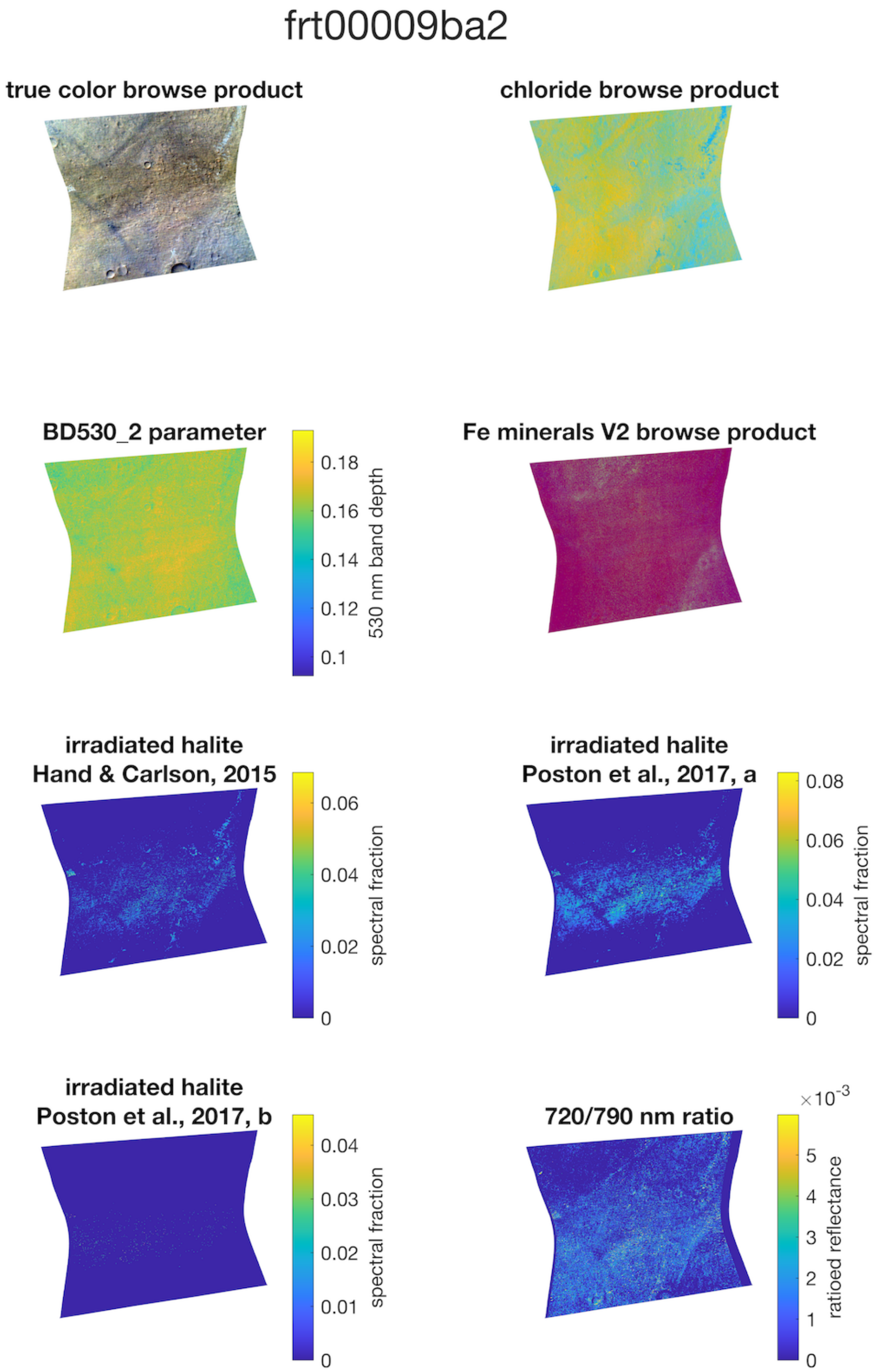

frt00009ba2
true color browse product
chloride browse product
BD530_2 parameter
0.18
0.16
0.14
0.12
0.1
530 nm band depth
Fe minerals V2 browse product
irradiated halite
Hand & Carlson, 2015
0.06
0.04
0.02
0
spectral fraction
irradiated halite
Poston et al., 2017, a
0.08
0.06
0.04
0.02
0
spectral fraction
irradiated halite
Poston et al., 2017, b
0.04
0.03
0.02
0.01
0
spectral fraction
720/790 nm ratio
×10^-3
5
4
3
2
1
0
ratioed reflectance

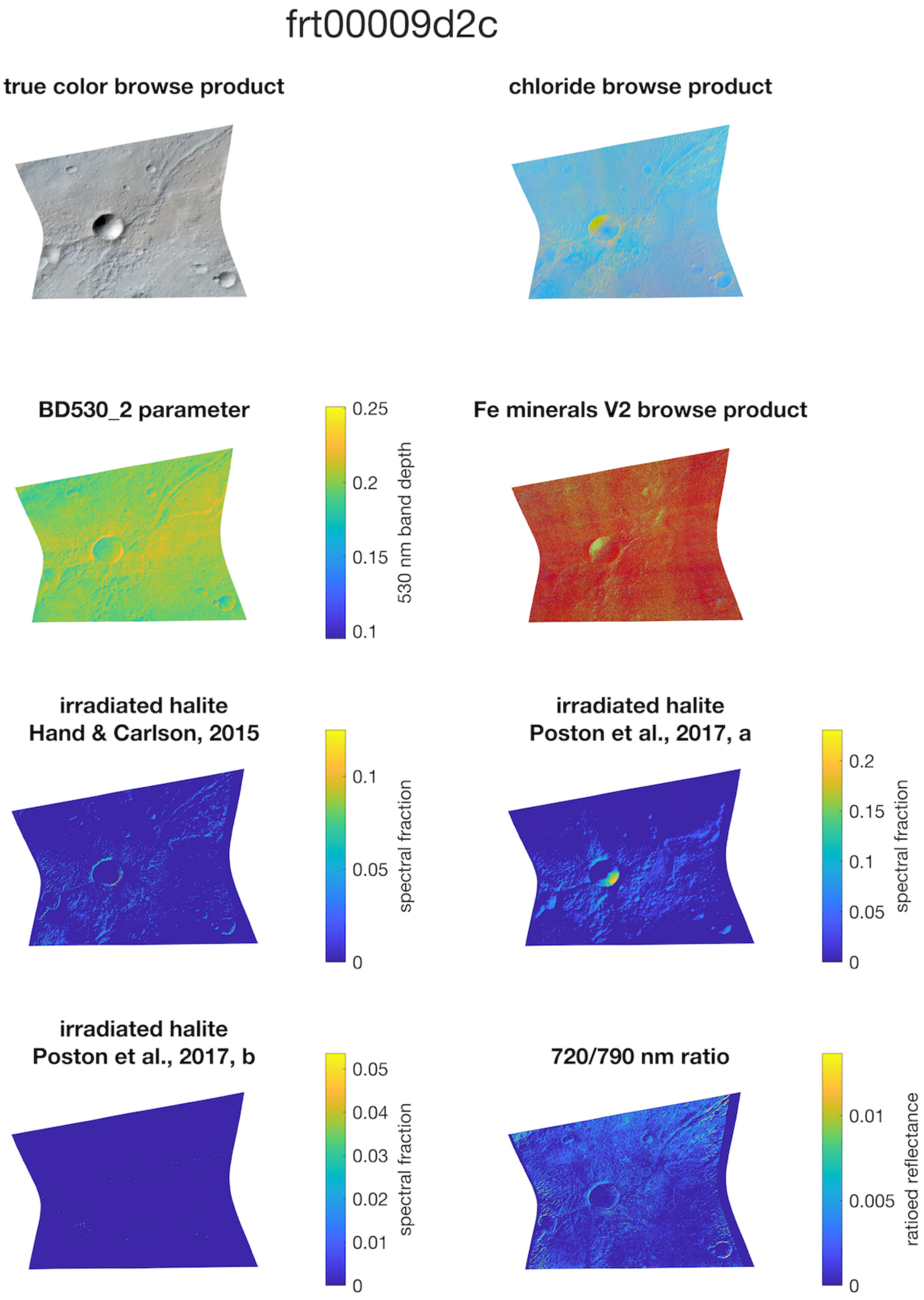
frt00009d2c
true color browse product
chloride browse product
BD530_2 parameter
0.25
0.2
0.15
0.1
530 nm band depth
Fe minerals V2 browse product
irradiated halite
Hand & Carlson, 2015
0.1
0.05
0
spectral fraction
irradiated halite
Poston et al., 2017, a
0.2
0.15
0.1
0.05
0
spectral fraction
irradiated halite
Poston et al., 2017, b
0.05
0.04
0.03
0.02
0.01
0
spectral fraction
720/790 nm ratio
0.01
0.005
0
ratioed reflectance

frt00010a4e

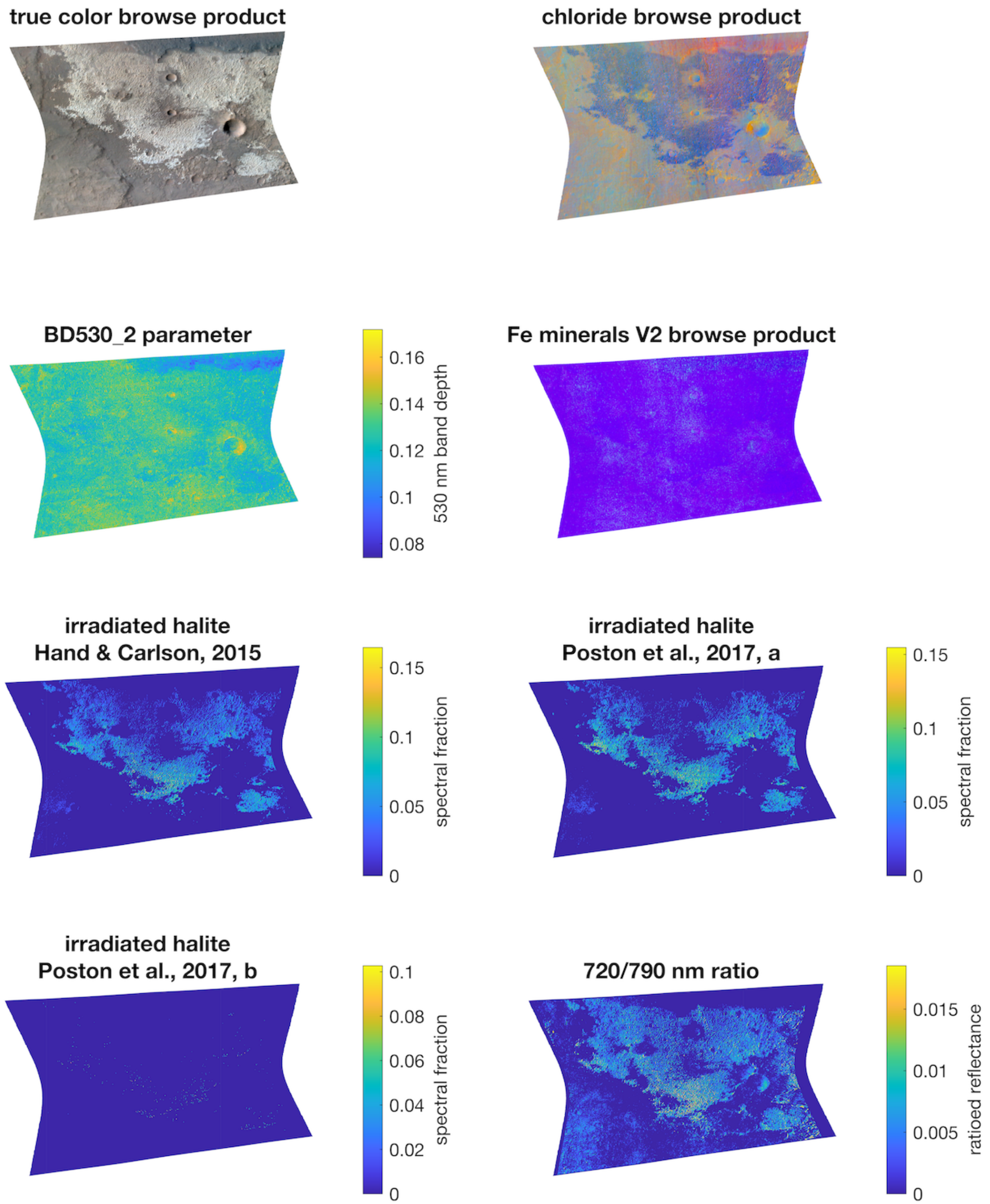

frt00012a09

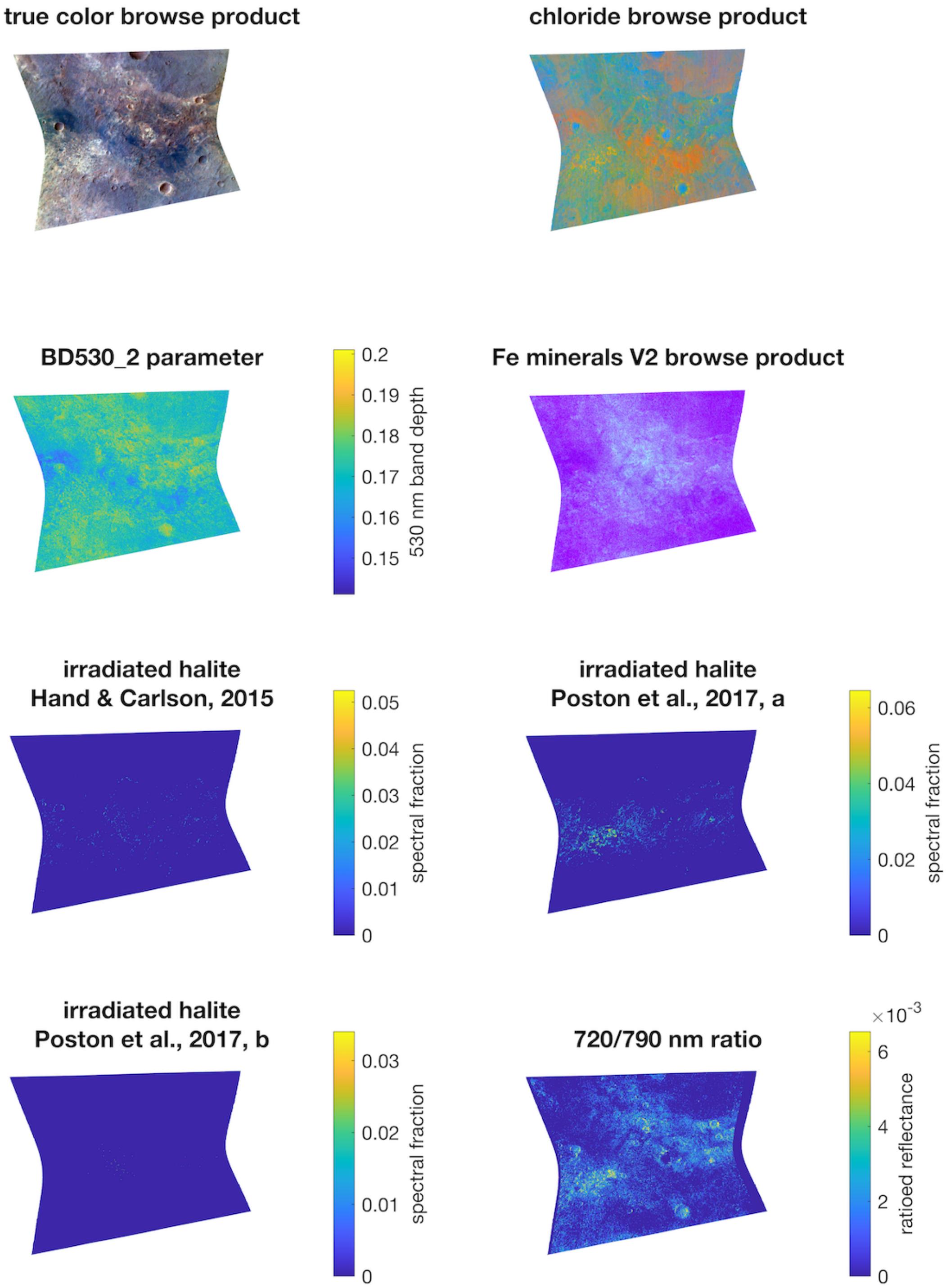

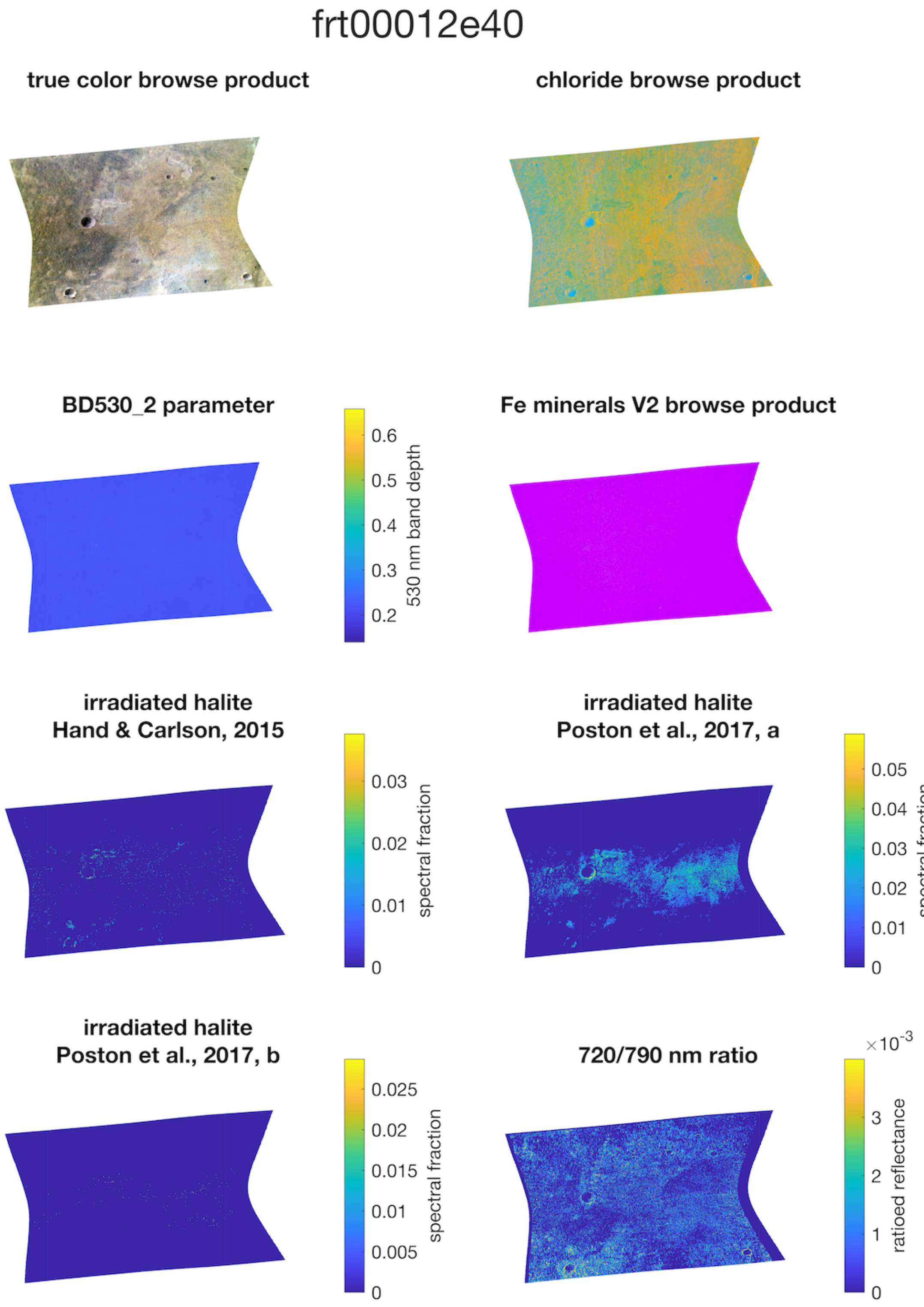
frt00012e40
true color browse product
chloride browse product
BD530_2 parameter
530 nm band depth
0.6
0.5
0.4
0.3
0.2
Fe minerals V2 browse product
irradiated halite
Hand & Carlson, 2015
spectral fraction
0.03
0.02
0.01
0
irradiated halite
Poston et al., 2017, a
0.05
0.04
0.03
0.02
0.01
0
irradiated halite
Poston et al., 2017, b
0.025
0.02
0.015
0.01
0.005
0
720/790 nm ratio
×10^-3
ratioed reflectance
3
2
1
0

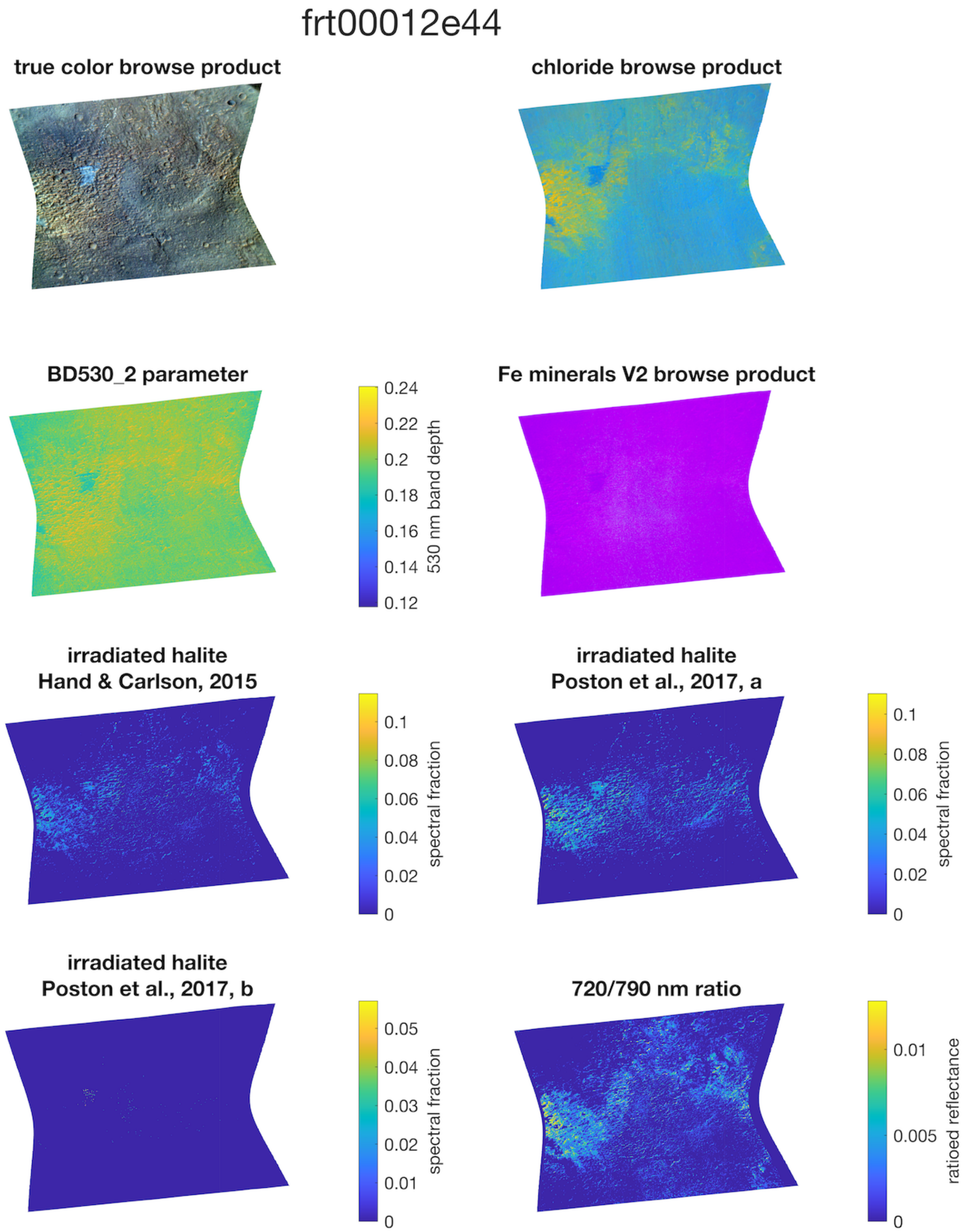
frt00012e44
true color browse product
chloride browse product
BD530_2 parameter
530 nm band depth
0.24
0.22
0.2
0.18
0.16
0.14
0.12
Fe minerals V2 browse product
irradiated halite
Hand & Carlson, 2015
spectral fraction
0.1
0.08
0.06
0.04
0.02
0
irradiated halite
Poston et al., 2017, a
spectral fraction
0.1
0.08
0.06
0.04
0.02
0
irradiated halite
Poston et al., 2017, b
spectral fraction
0.05
0.04
0.03
0.02
0.01
0
720/790 nm ratio
ratioed reflectance
0.01
0.005
0

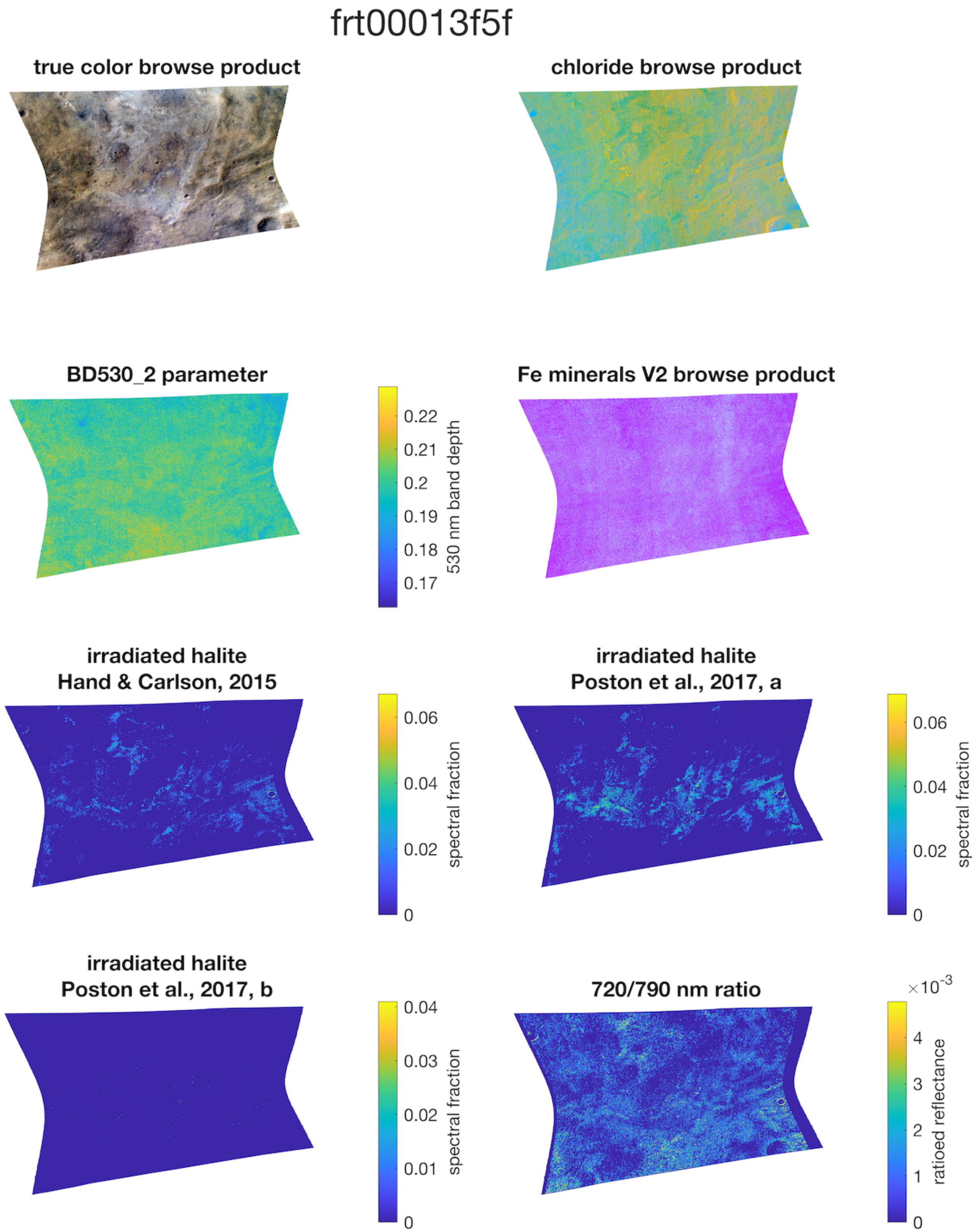

frt00013f5f
true color browse product
chloride browse product
BD530_2 parameter
530 nm band depth
0.22
0.21
0.2
0.19
0.18
0.17
Fe minerals V2 browse product
irradiated halite
Hand & Carlson, 2015
spectral fraction
0.06
0.04
0.02
0
irradiated halite
Poston et al., 2017, a
spectral fraction
0.06
0.04
0.02
0
irradiated halite
Poston et al., 2017, b
spectral fraction
0.04
0.03
0.02
0.01
0
720/790 nm ratio
×10^-3
ratioed reflectance
4
3
2
1
0

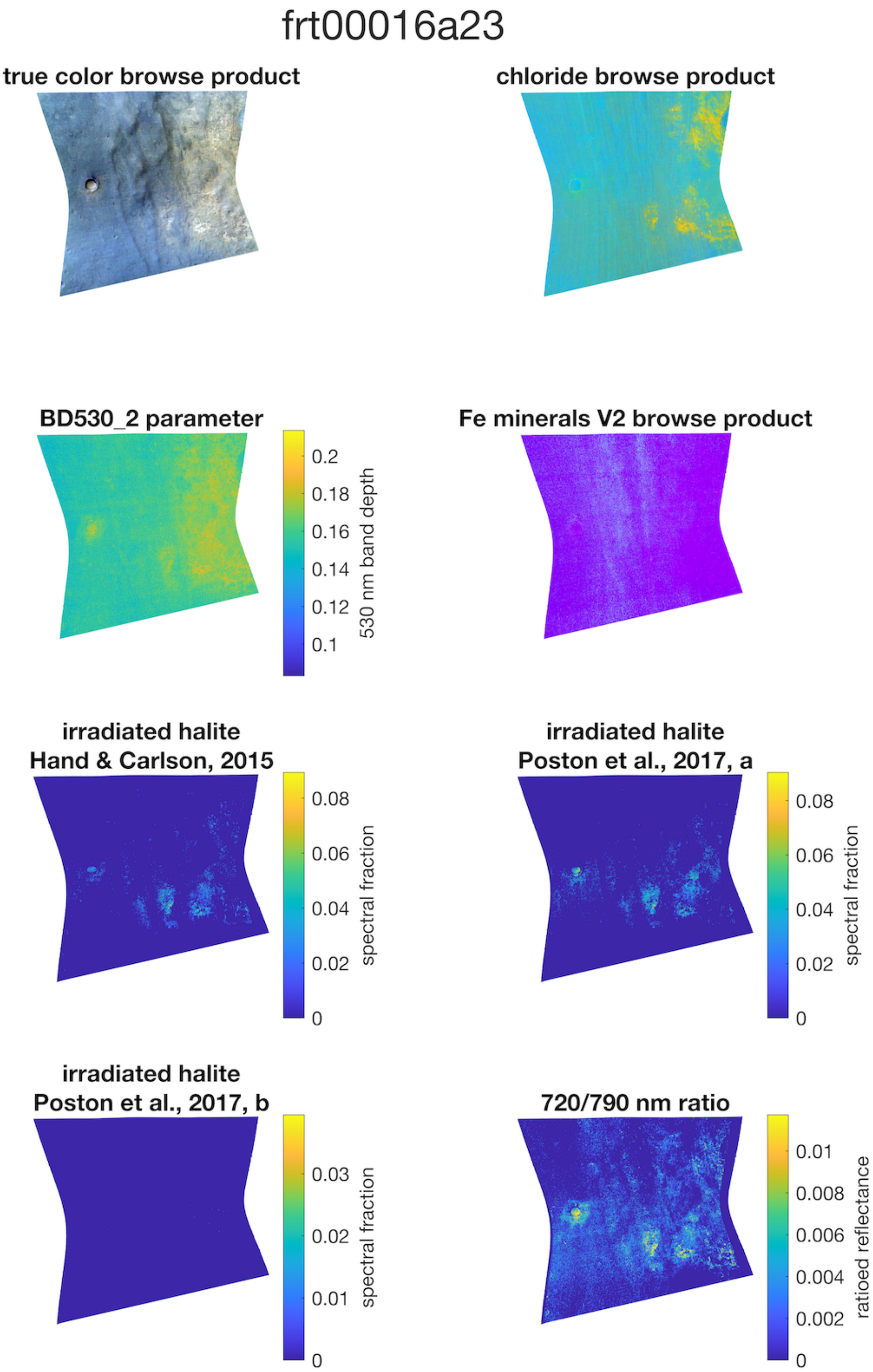

frt00016a23
true color browse product
chloride browse product
BD530_2 parameter
530 nm band depth
0.1
0.12
0.14
0.16
0.18
0.2
Fe minerals V2 browse product
irradiated halite
Hand & Carlson, 2015
spectral fraction
0
0.02
0.04
0.06
0.08
irradiated halite
Poston et al., 2017, a
spectral fraction
0
0.02
0.04
0.06
0.08
irradiated halite
Poston et al., 2017, b
spectral fraction
0
0.01
0.02
0.03
720/790 nm ratio
ratioed reflectance
0
0.002
0.004
0.006
0.008
0.01

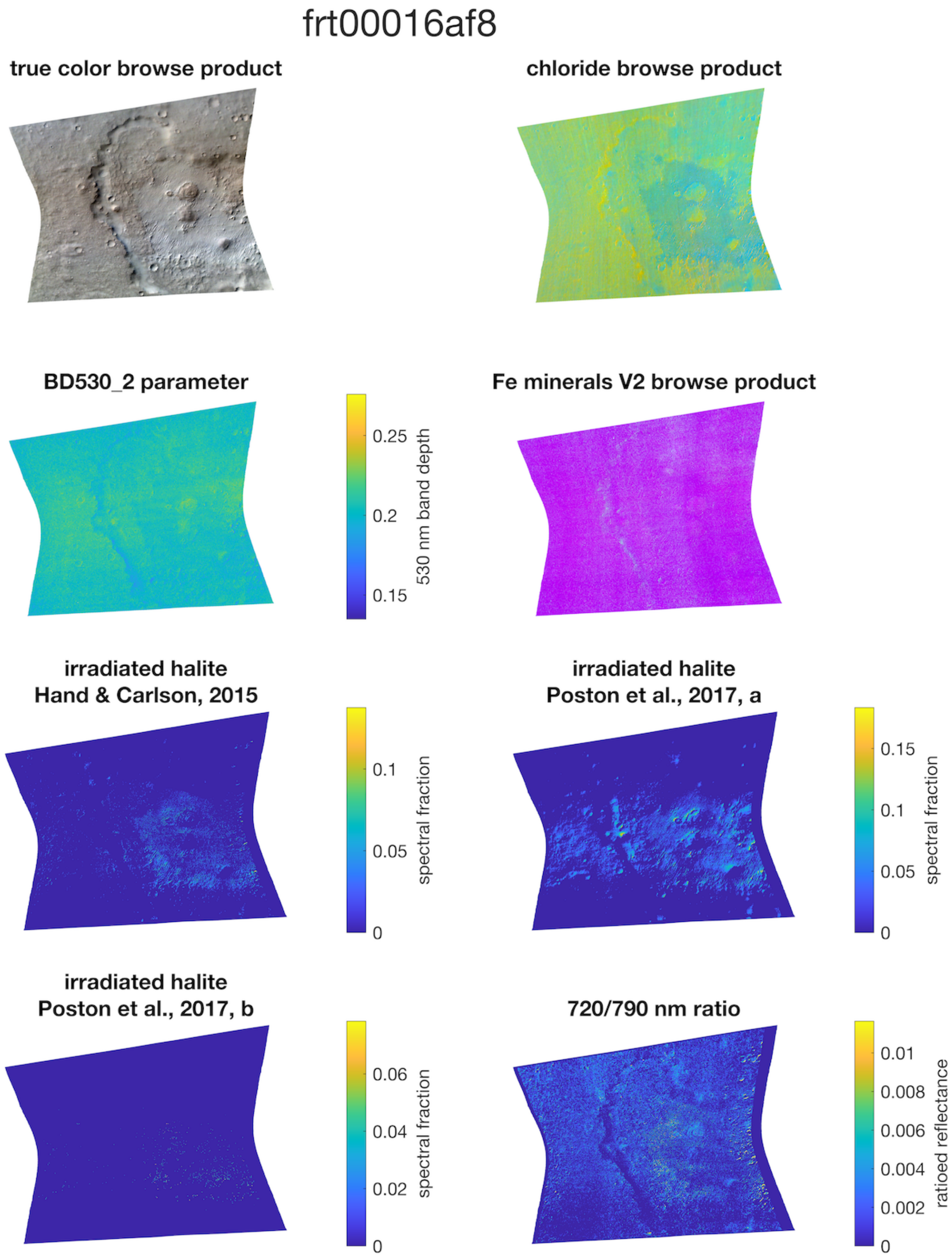
frt00016af8
true color browse product
chloride browse product
BD530_2 parameter
0.25
0.2
0.15
530 nm band depth
Fe minerals V2 browse product
irradiated halite
Hand & Carlson, 2015
0.1
0.05
0
spectral fraction
irradiated halite
Poston et al., 2017, a
0.15
0.1
0.05
0
spectral fraction
irradiated halite
Poston et al., 2017, b
0.06
0.04
0.02
0
spectral fraction
720/790 nm ratio
0.01
0.008
0.006
0.004
0.002
0
ratioed reflectance

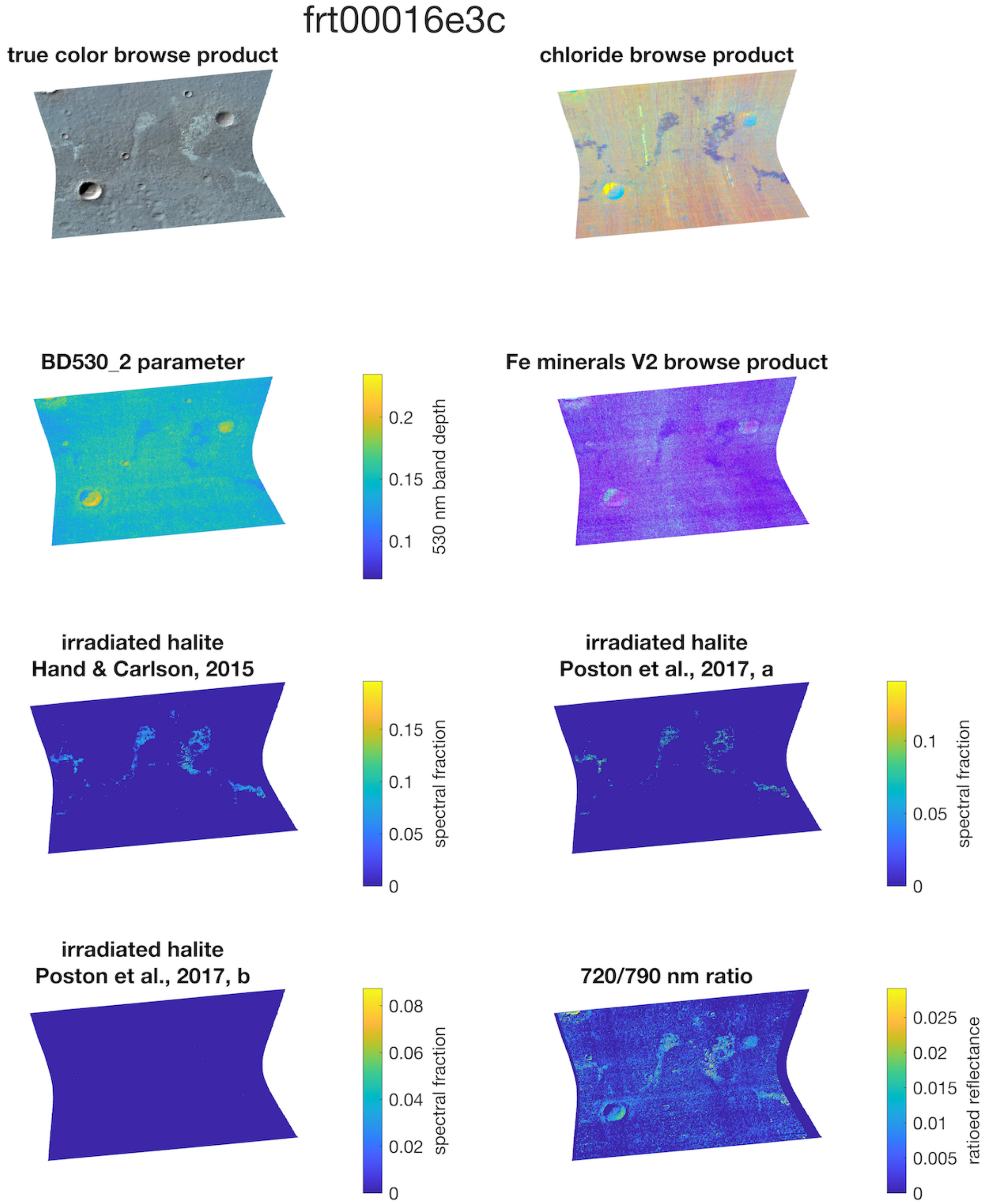
frt00016e3c
true color browse product
chloride browse product
BD530_2 parameter
0.2
0.15
0.1
530 nm band depth
Fe minerals V2 browse product
irradiated halite
Hand & Carlson, 2015
0.15
0.1
0.05
0
spectral fraction
irradiated halite
Poston et al., 2017, a
0.1
0.05
0
spectral fraction
irradiated halite
Poston et al., 2017, b
0.08
0.06
0.04
0.02
0
spectral fraction
720/790 nm ratio
0.025
0.02
0.015
0.01
0.005
0
ratioed reflectance

frt00016ee8

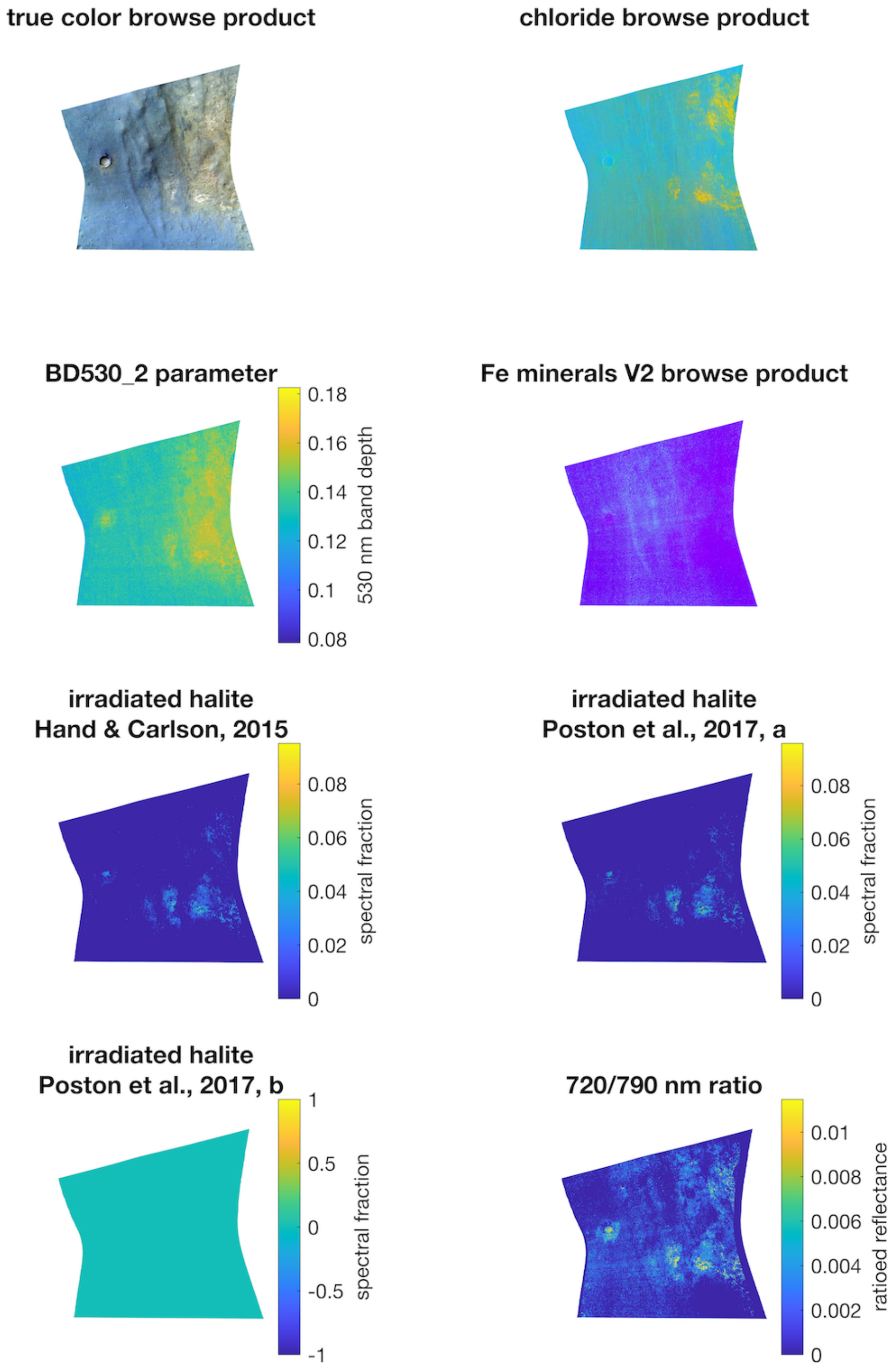

frt00017afb

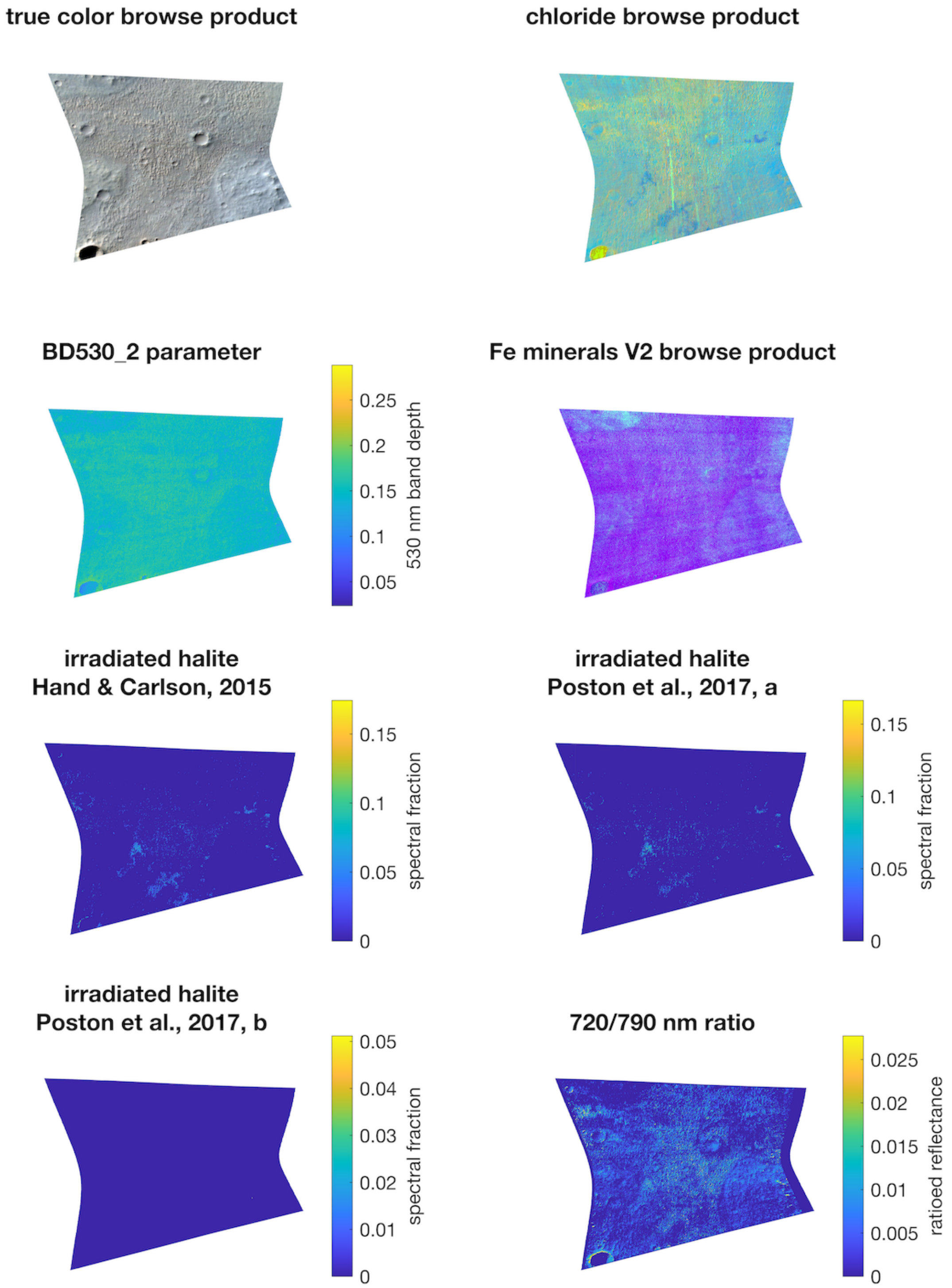

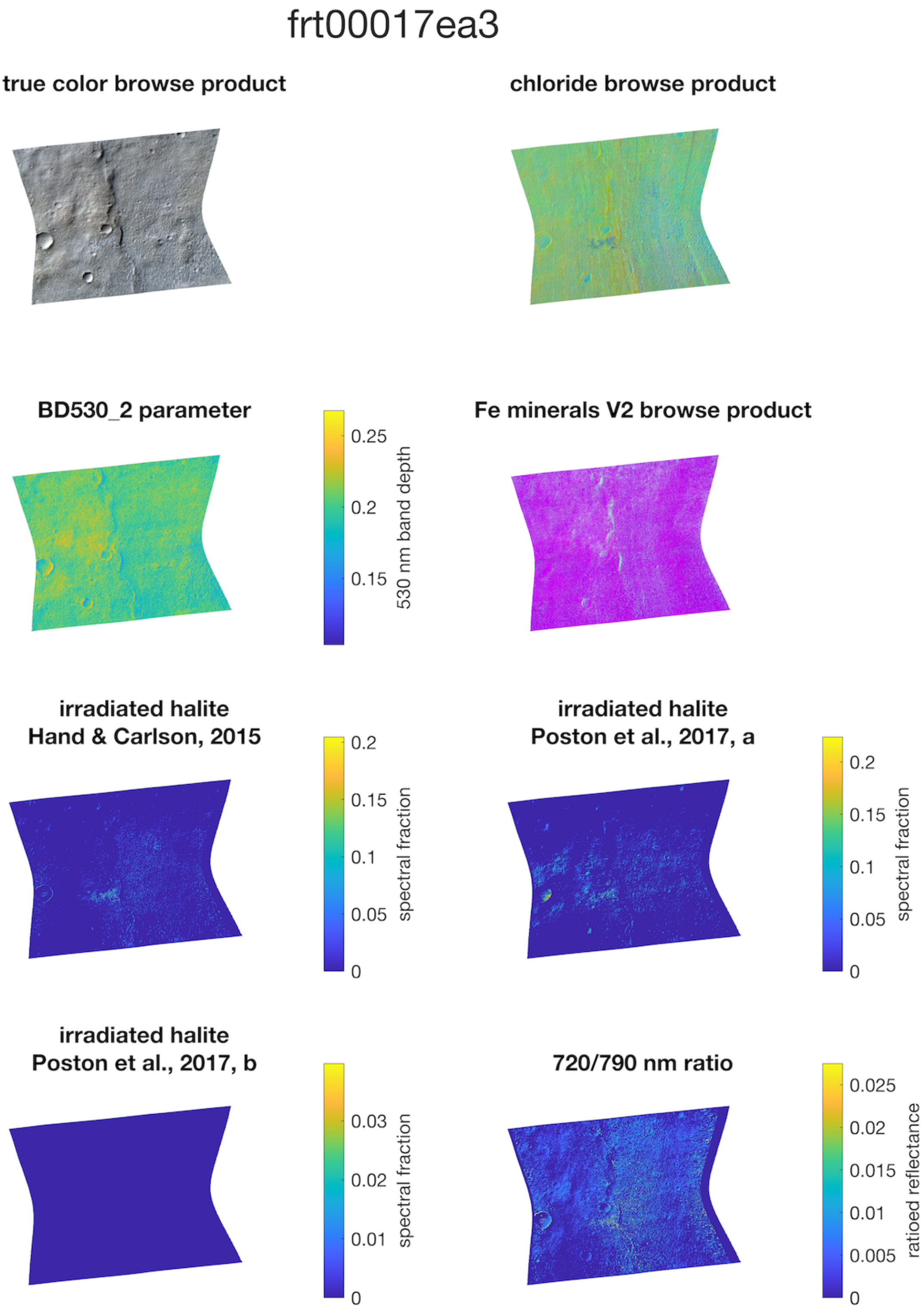
frt00017ea3
true color browse product
chloride browse product
BD530_2 parameter
0.25
0.2
0.15
530 nm band depth
Fe minerals V2 browse product
irradiated halite
Hand & Carlson, 2015
0.2
0.15
0.1
0.05
0
spectral fraction
irradiated halite
Poston et al., 2017, a
0.2
0.15
0.1
0.05
0
spectral fraction
irradiated halite
Poston et al., 2017, b
0.03
0.02
0.01
0
spectral fraction
720/790 nm ratio
0.025
0.02
0.015
0.01
0.005
0
ratioed reflectance

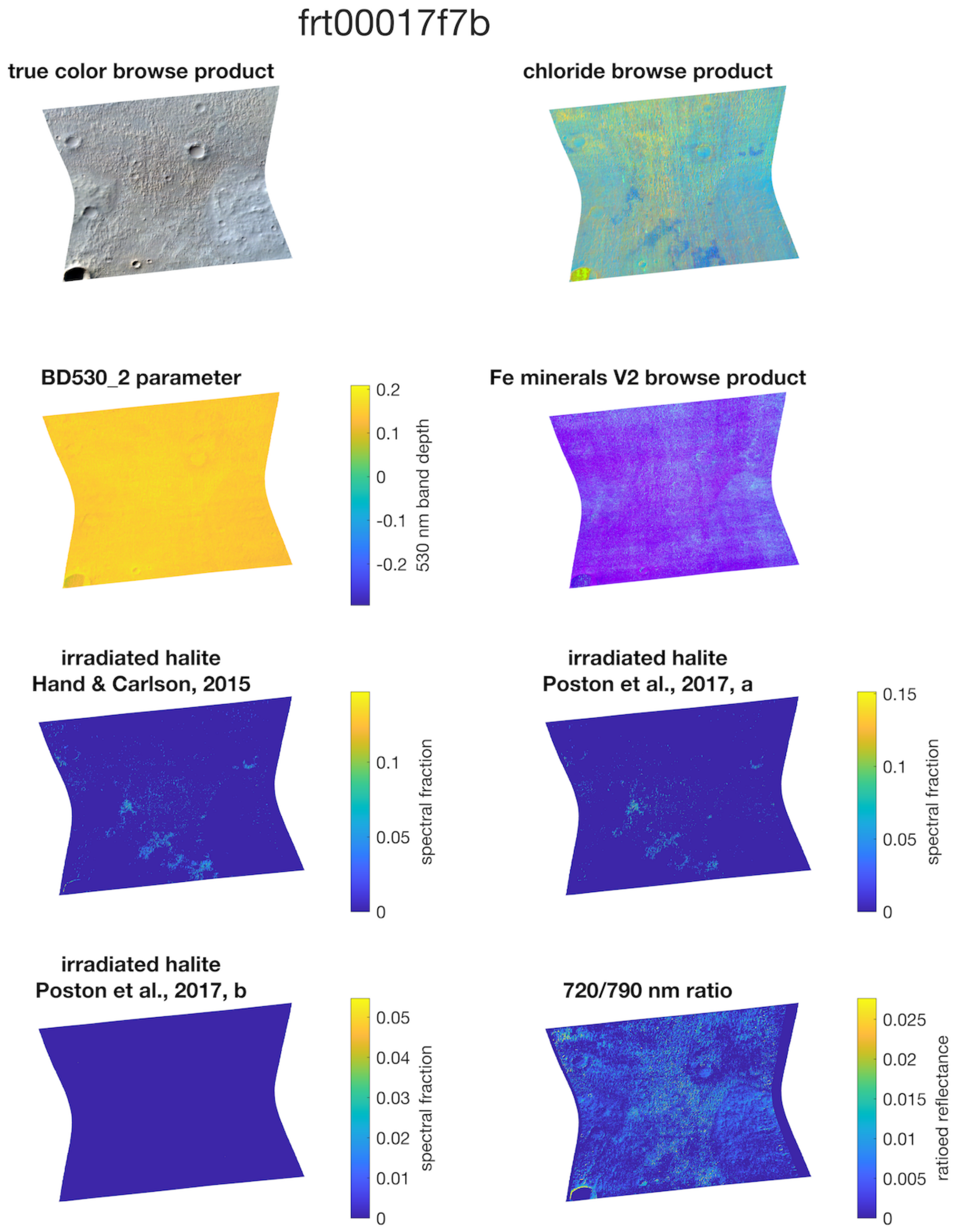
frt00017f7b
true color browse product
chloride browse product
BD530_2 parameter
0.2
0.1
0
-0.1
-0.2
530 nm band depth
Fe minerals V2 browse product
irradiated halite
Hand & Carlson, 2015
0.1
0.05
0
spectral fraction
irradiated halite
Poston et al., 2017, a
0.15
0.1
0.05
0
spectral fraction
irradiated halite
Poston et al., 2017, b
0.05
0.04
0.03
0.02
0.01
0
spectral fraction
720/790 nm ratio
0.025
0.02
0.015
0.01
0.005
0
ratioed reflectance

frt00018af5

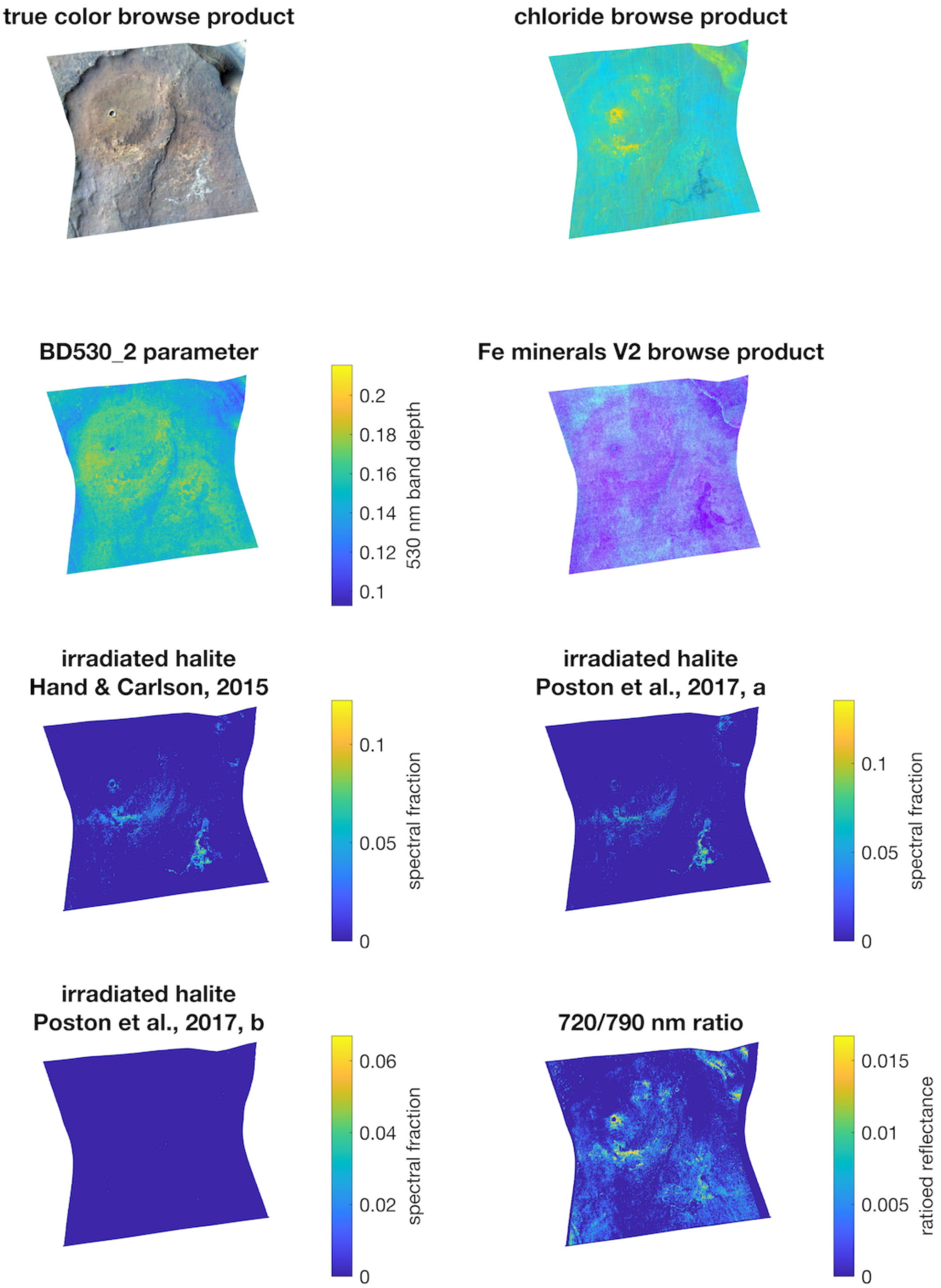

frt000081b1

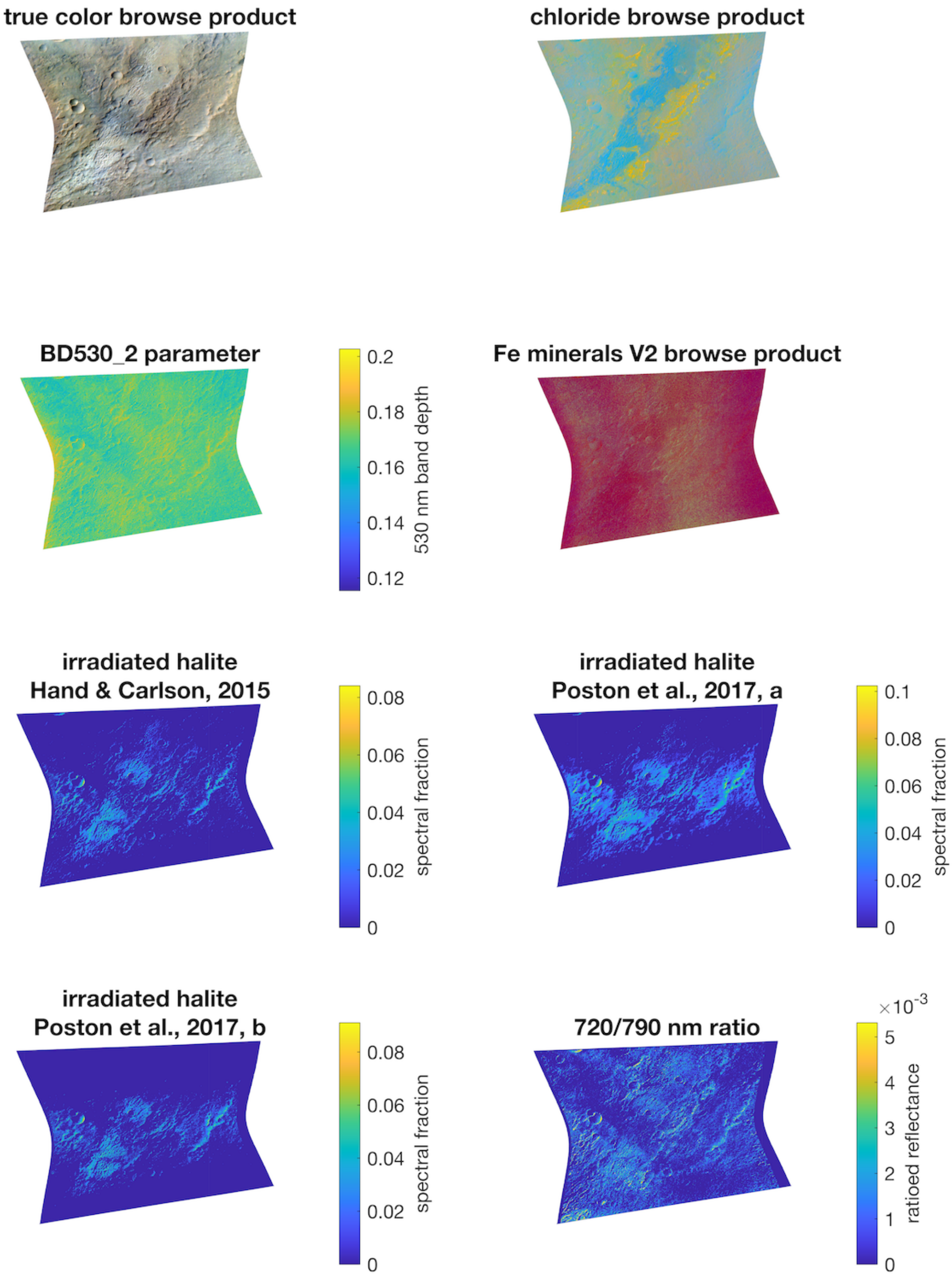

frt000101de

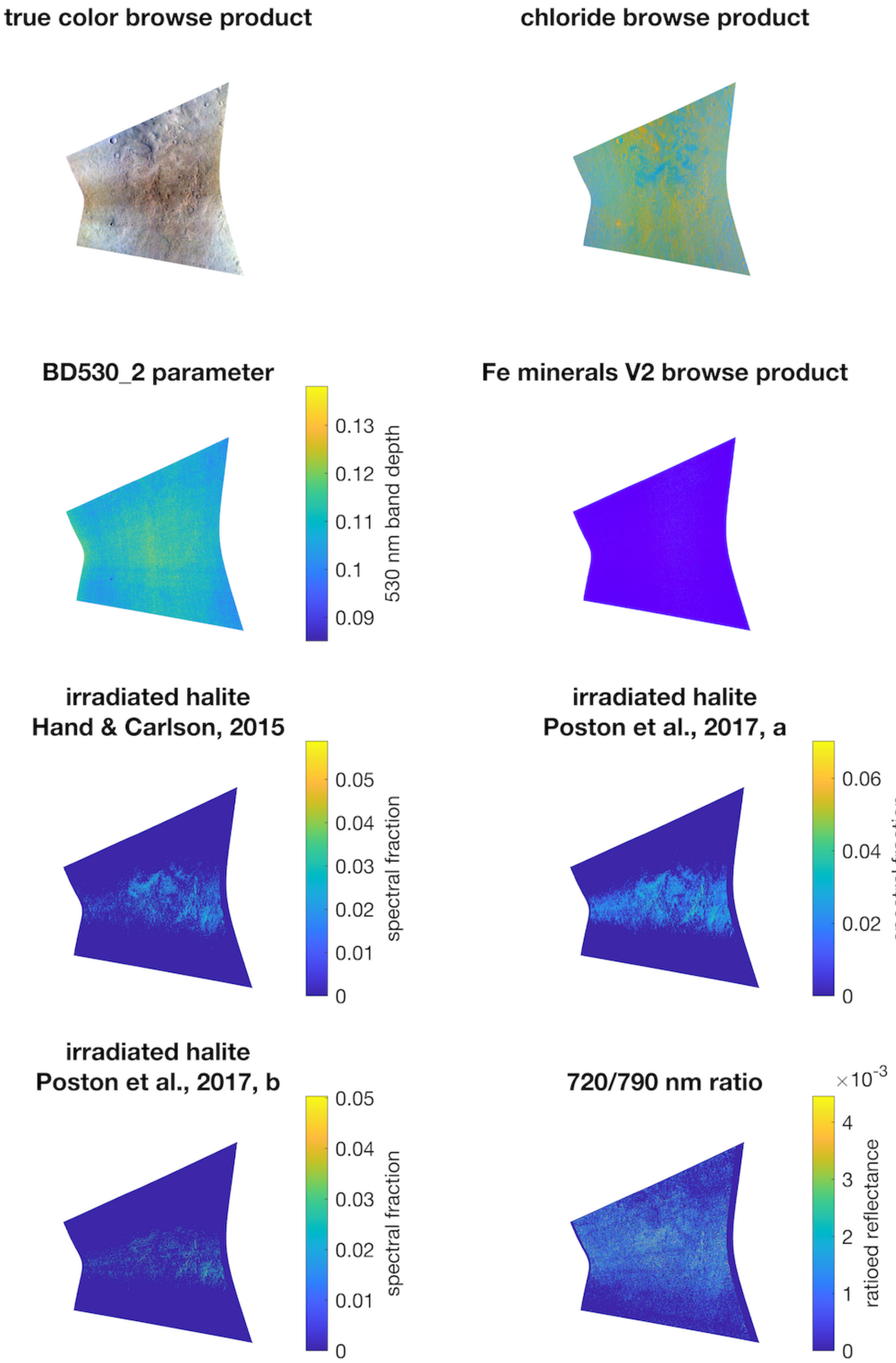

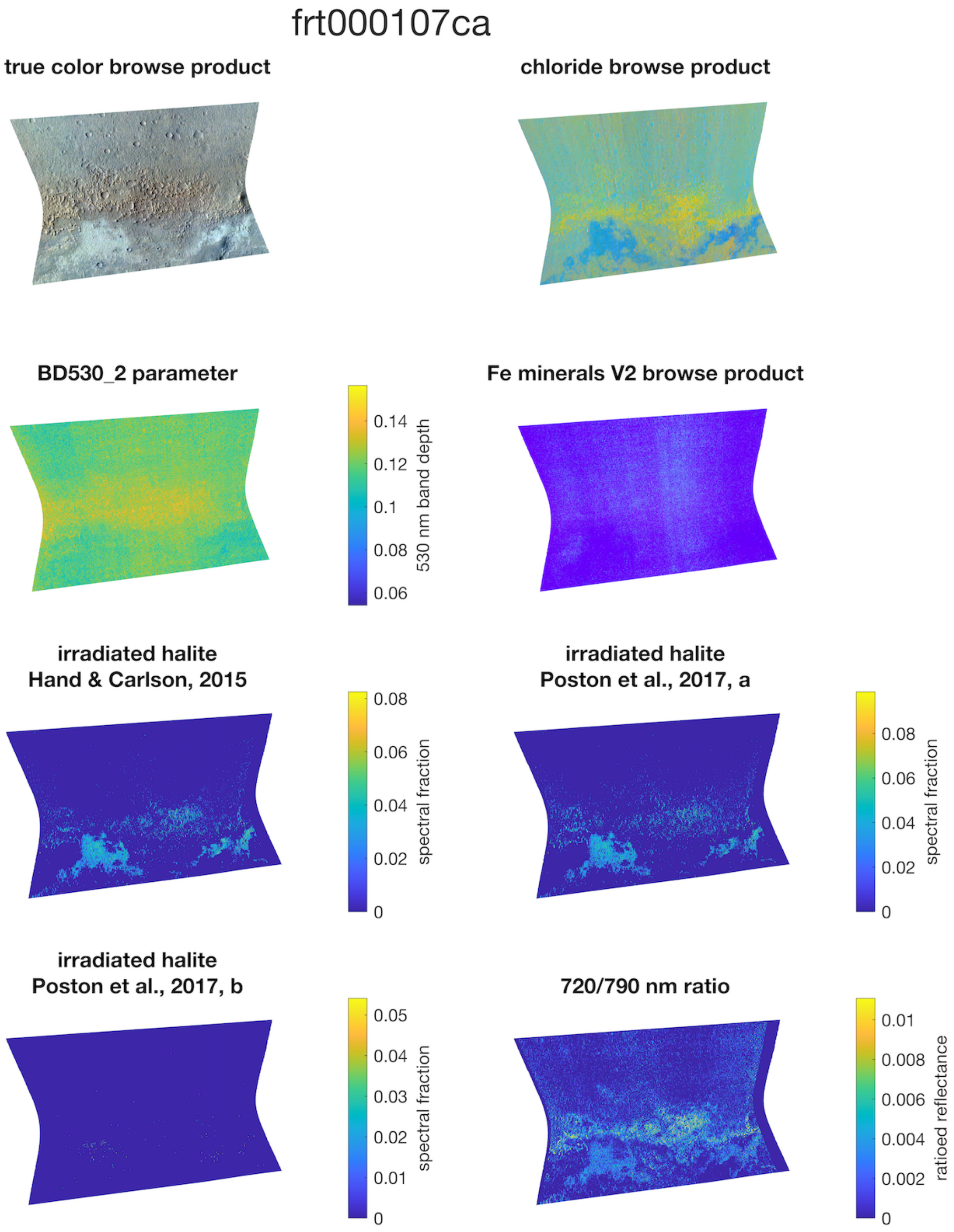
frt000107ca
true color browse product
chloride browse product
BD530_2 parameter
0.14
0.12
0.1
0.08
0.06
530 nm band depth
Fe minerals V2 browse product
irradiated halite
Hand & Carlson, 2015
0.08
0.06
0.04
0.02
0
spectral fraction
irradiated halite
Poston et al., 2017, a
0.08
0.06
0.04
0.02
0
spectral fraction
irradiated halite
Poston et al., 2017, b
0.05
0.04
0.03
0.02
0.01
0
spectral fraction
720/790 nm ratio
0.01
0.008
0.006
0.004
0.002
0
ratioed reflectance

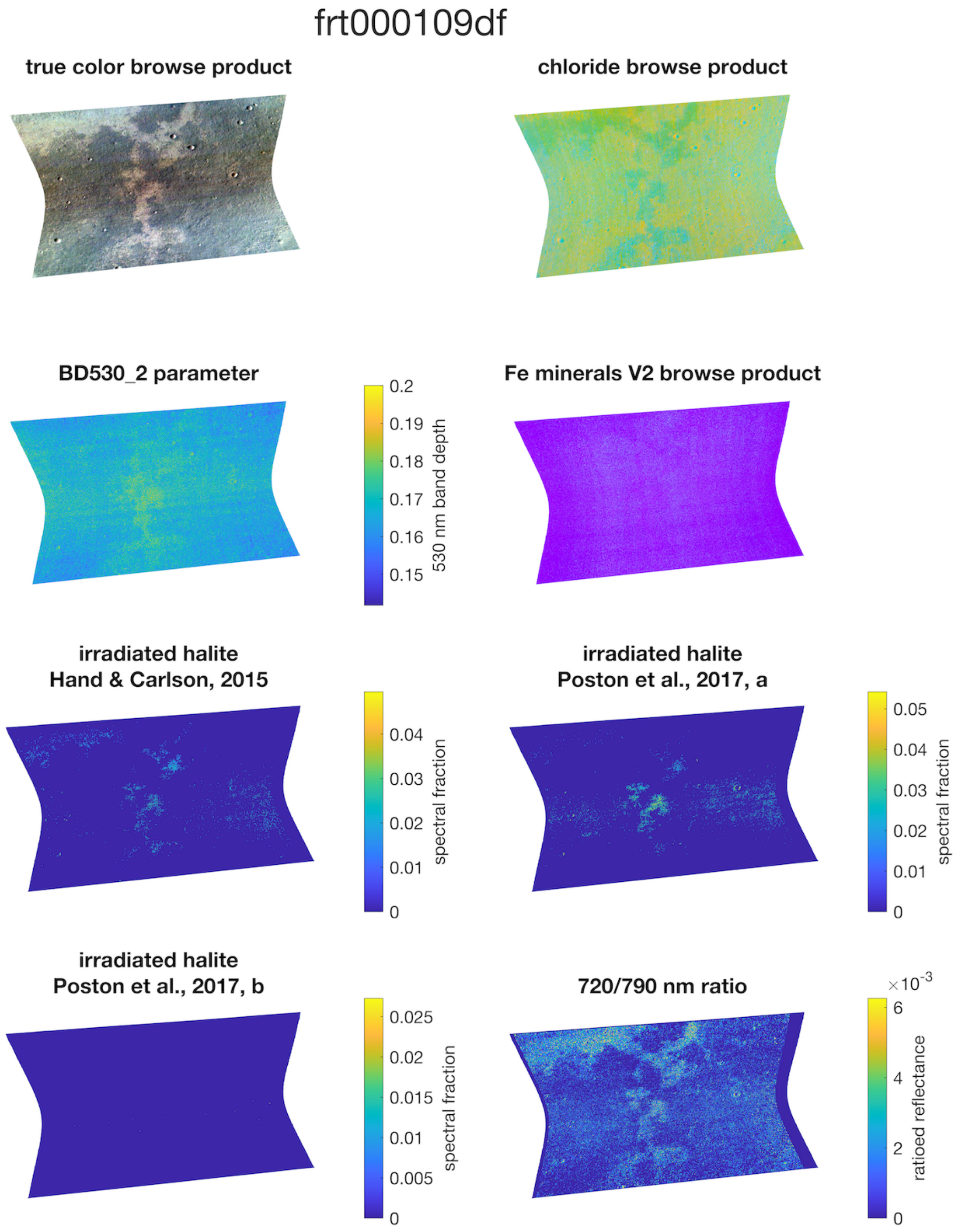

frt000109df
true color browse product
chloride browse product
BD530_2 parameter
530 nm band depth
0.2
0.19
0.18
0.17
0.16
0.15
Fe minerals V2 browse product
irradiated halite
Hand & Carlson, 2015
spectral fraction
0.04
0.03
0.02
0.01
0
irradiated halite
Poston et al., 2017, a
spectral fraction
0.05
0.04
0.03
0.02
0.01
0
irradiated halite
Poston et al., 2017, b
spectral fraction
0.025
0.02
0.015
0.01
0.005
0
720/790 nm ratio
×10^-3
ratioed reflectance
6
4
2
0

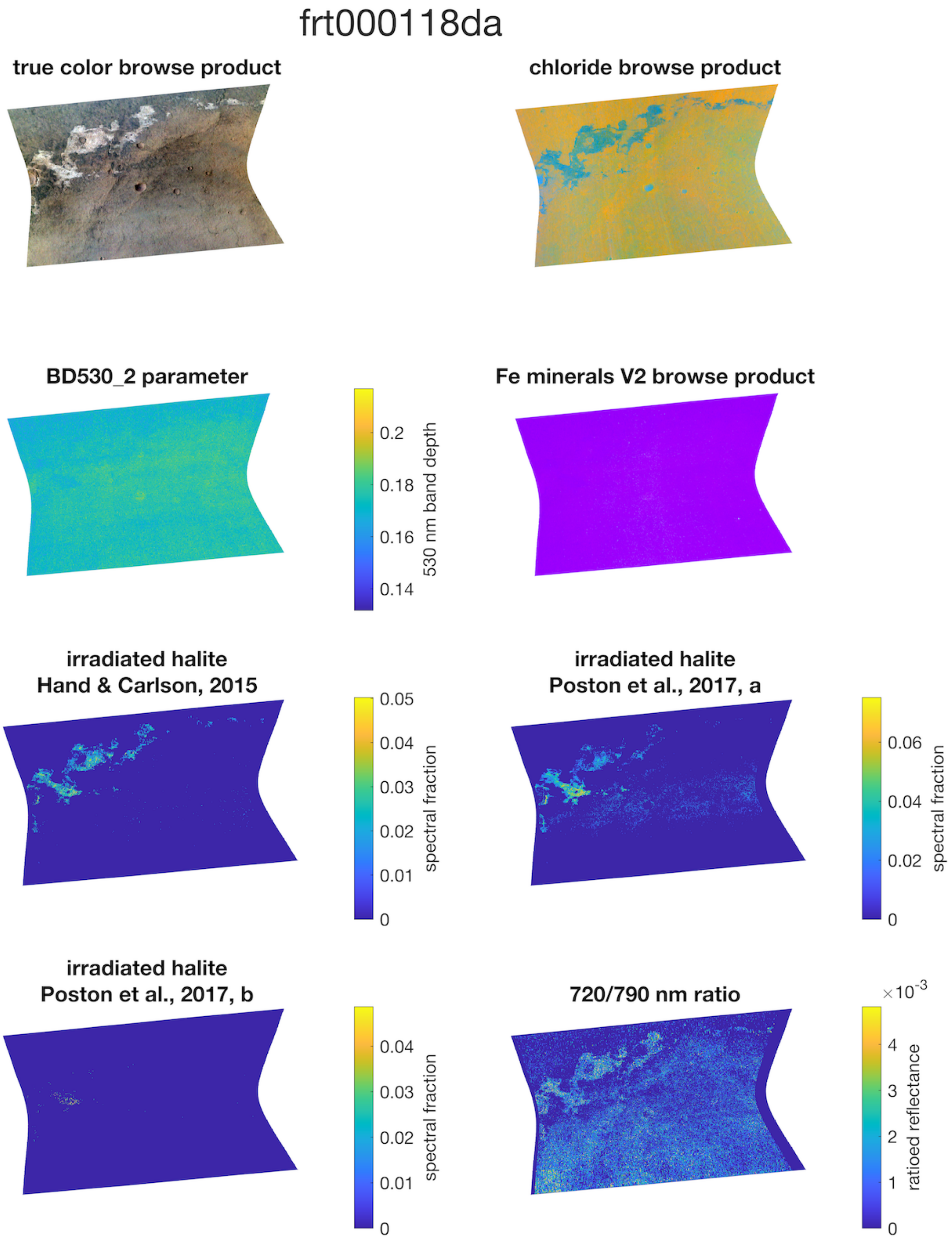
frt000118da
true color browse product
chloride browse product
BD530_2 parameter
0.2
0.18
0.16
0.14
530 nm band depth
Fe minerals V2 browse product
irradiated halite
Hand & Carlson, 2015
0.05
0.04
0.03
0.02
0.01
0
spectral fraction
irradiated halite
Poston et al., 2017, a
0.06
0.04
0.02
0
spectral fraction
irradiated halite
Poston et al., 2017, b
0.04
0.03
0.02
0.01
0
spectral fraction
720/790 nm ratio
×10-3
4
3
2
1
0
ratioed reflectance

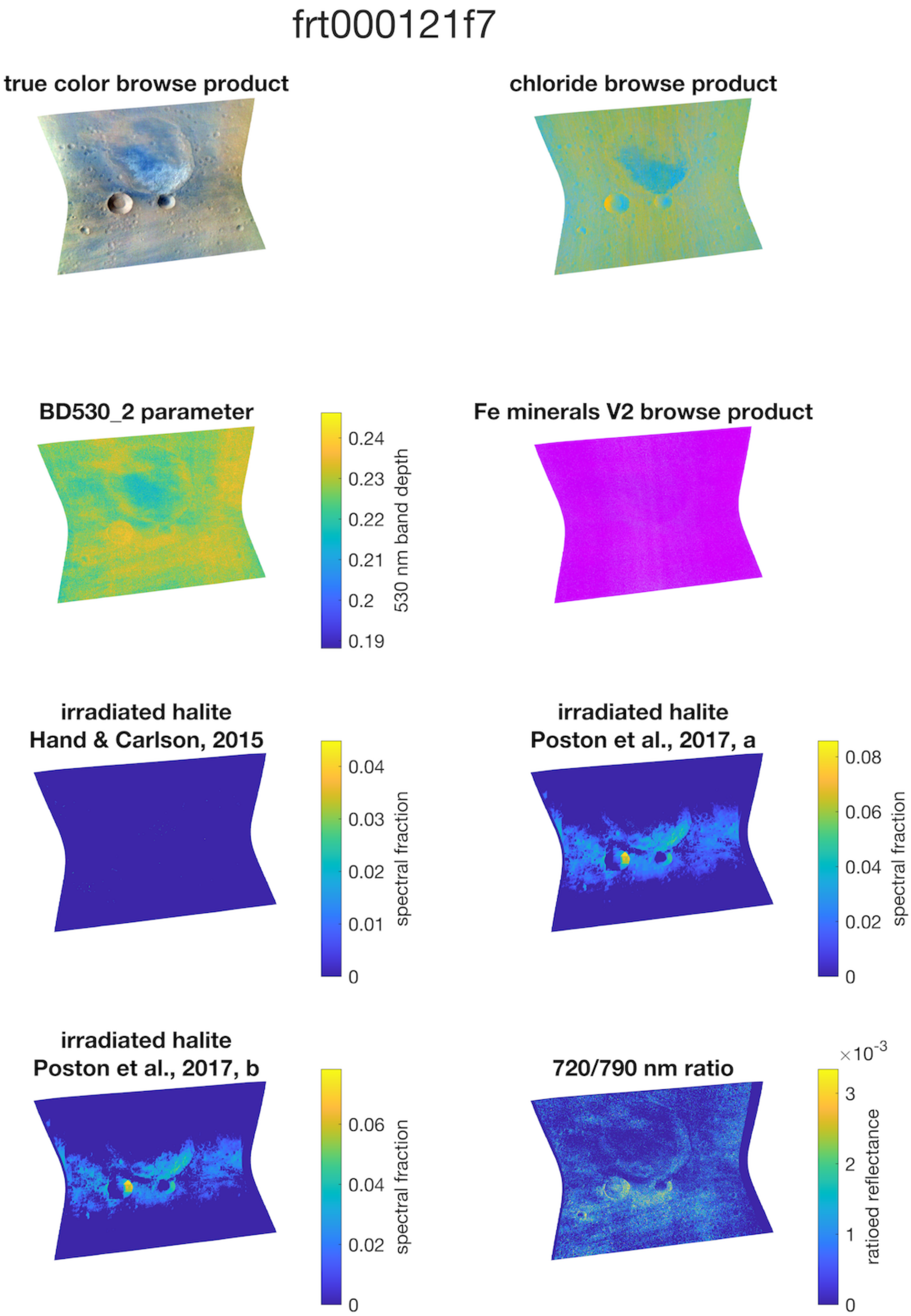

frt000121f7
true color browse product
chloride browse product
BD530_2 parameter
530 nm band depth
0.24
0.23
0.22
0.21
0.2
0.19
Fe minerals V2 browse product
irradiated halite
Hand & Carlson, 2015
spectral fraction
0.04
0.03
0.02
0.01
0
irradiated halite
Poston et al., 2017, a
spectral fraction
0.08
0.06
0.04
0.02
0
irradiated halite
Poston et al., 2017, b
spectral fraction
0.06
0.04
0.02
0
720/790 nm ratio
×10-3
ratioed reflectance
3
2
1
0

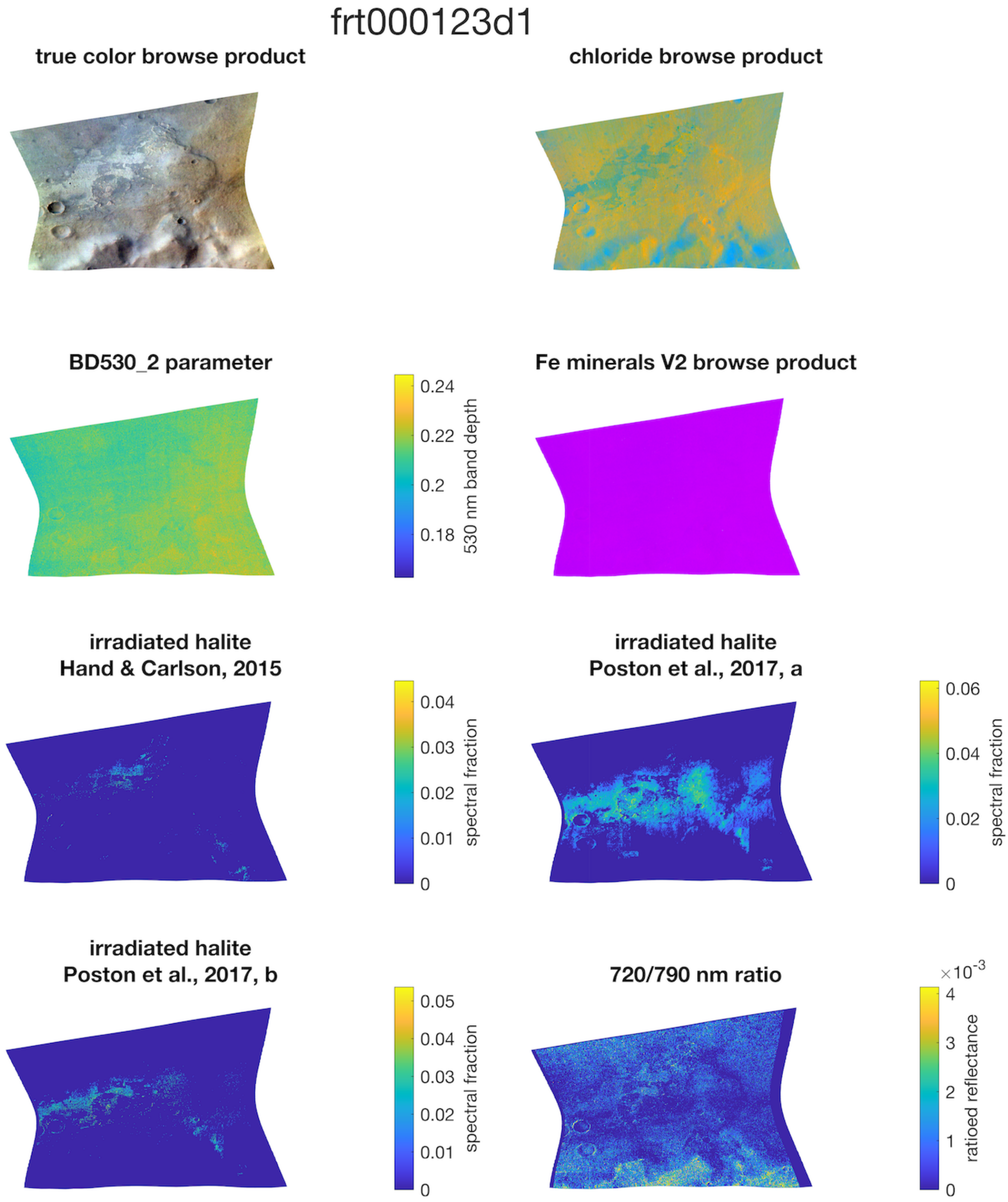
frt000123d1
true color browse product
chloride browse product
BD530_2 parameter
0.24
0.22
0.2
0.18
530 nm band depth
Fe minerals V2 browse product
irradiated halite
Hand & Carlson, 2015
0.04
0.03
0.02
0.01
0
spectral fraction
irradiated halite
Poston et al., 2017, a
0.06
0.04
0.02
0
spectral fraction
irradiated halite
Poston et al., 2017, b
0.05
0.04
0.03
0.02
0.01
0
spectral fraction
720/790 nm ratio
×10-3
4
3
2
1
0
ratioed reflectance

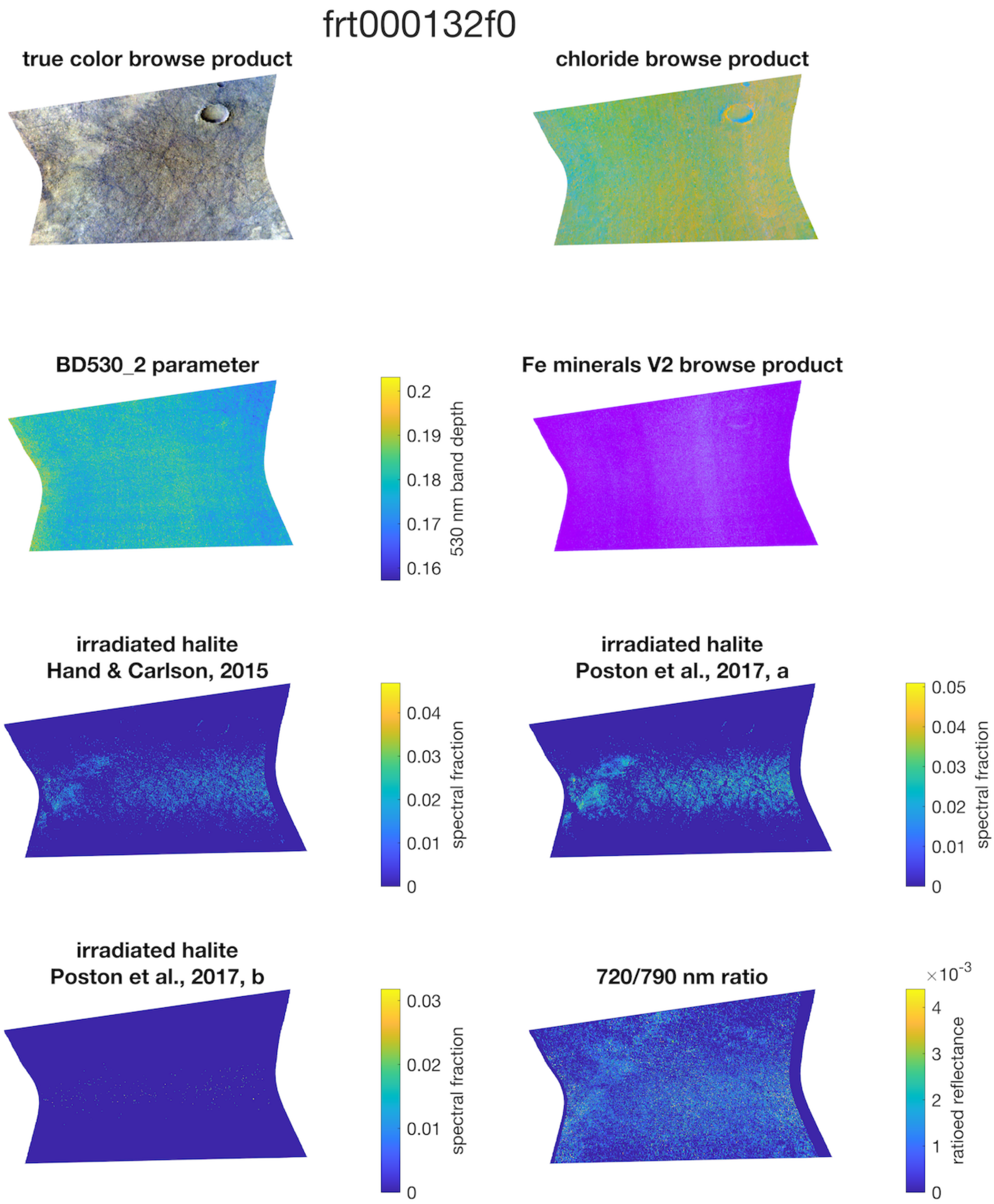
frt000132f0
true color browse product
chloride browse product
BD530_2 parameter
530 nm band depth
0.2
0.19
0.18
0.17
0.16
Fe minerals V2 browse product
irradiated halite
Hand & Carlson, 2015
spectral fraction
0.04
0.03
0.02
0.01
0
irradiated halite
Poston et al., 2017, a
0.05
0.04
0.03
0.02
0.01
0
spectral fraction
irradiated halite
Poston et al., 2017, b
0.03
0.02
0.01
0
spectral fraction
720/790 nm ratio
×10^-3
4
3
2
1
0
ratioed reflectance

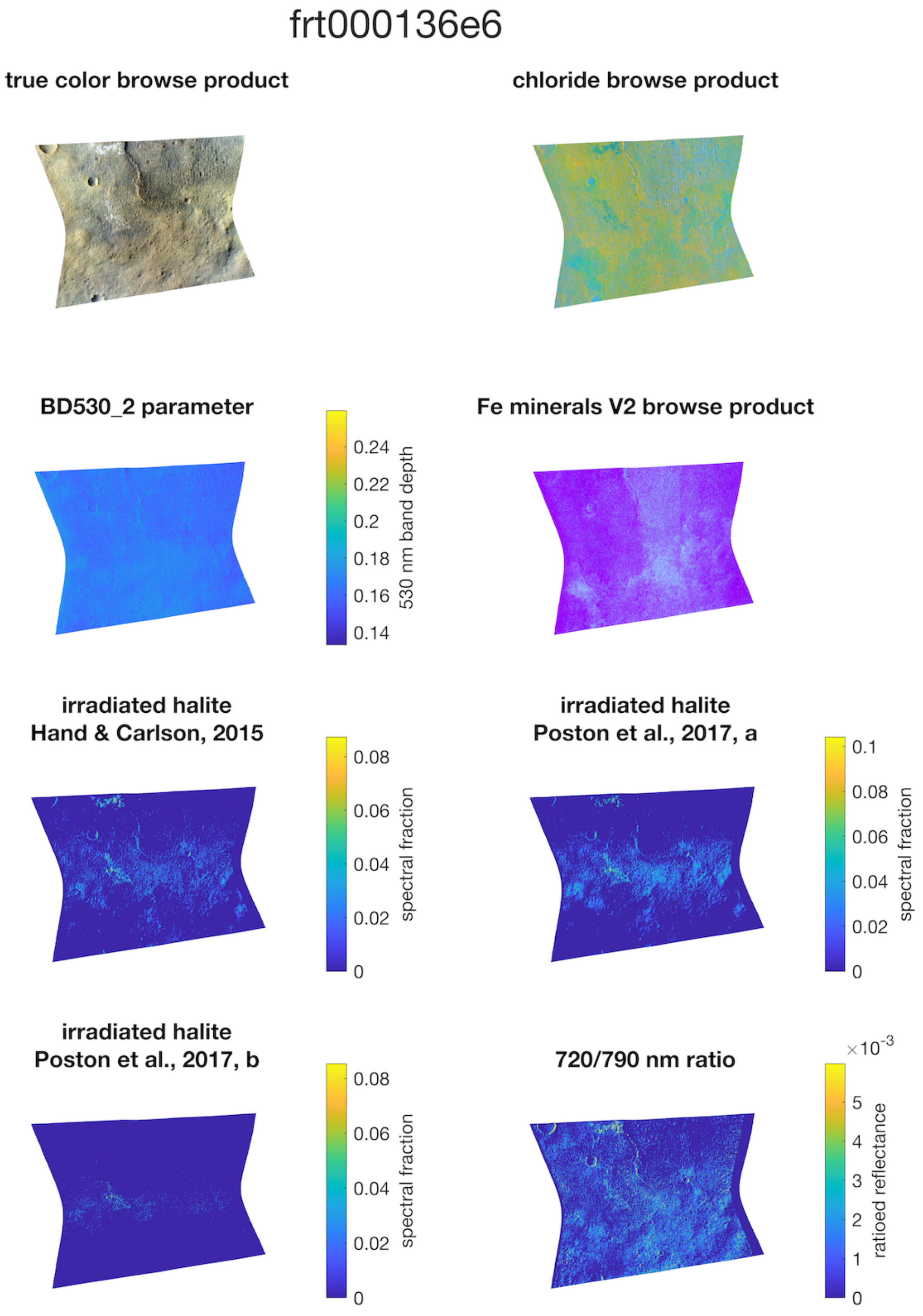

frt000136e6
true color browse product
chloride browse product
BD530_2 parameter
0.24
0.22
0.2
0.18
0.16
0.14
530 nm band depth
Fe minerals V2 browse product
irradiated halite
Hand & Carlson, 2015
0.08
0.06
0.04
0.02
0
spectral fraction
irradiated halite
Poston et al., 2017, a
0.1
0.08
0.06
0.04
0.02
0
spectral fraction
irradiated halite
Poston et al., 2017, b
0.08
0.06
0.04
0.02
0
spectral fraction
720/790 nm ratio
×10^-3
5
4
3
2
1
0
ratioed reflectance

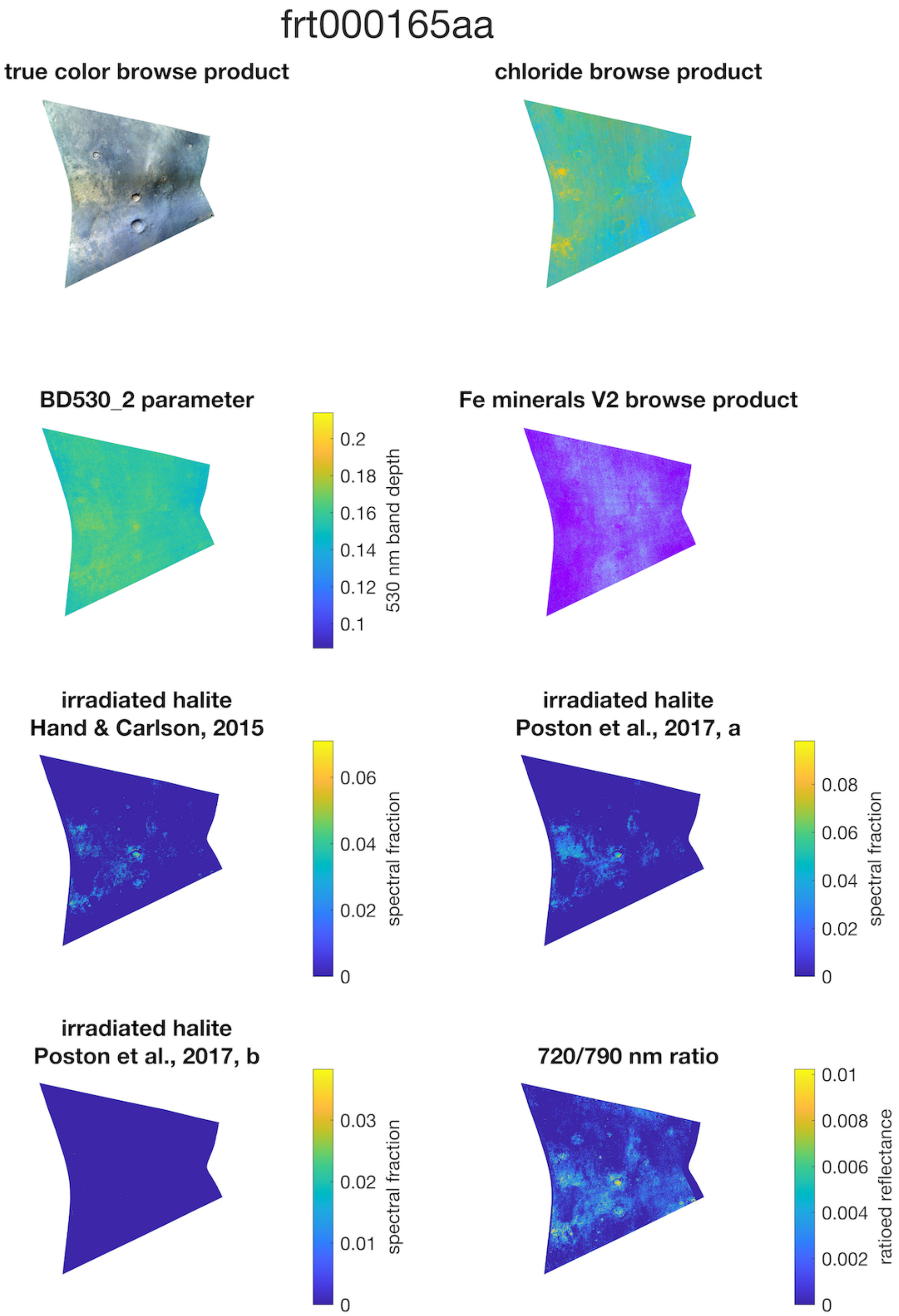
frt000165aa
true color browse product
chloride browse product
BD530_2 parameter
0.2
0.18
0.16
0.14
0.12
0.1
530 nm band depth
Fe minerals V2 browse product
irradiated halite
Hand & Carlson, 2015
0.06
0.04
0.02
0
spectral fraction
irradiated halite
Poston et al., 2017, a
0.08
0.06
0.04
0.02
0
spectral fraction
irradiated halite
Poston et al., 2017, b
0.03
0.02
0.01
0
spectral fraction
720/790 nm ratio
0.01
0.008
0.006
0.004
0.002
0
ratioed reflectance

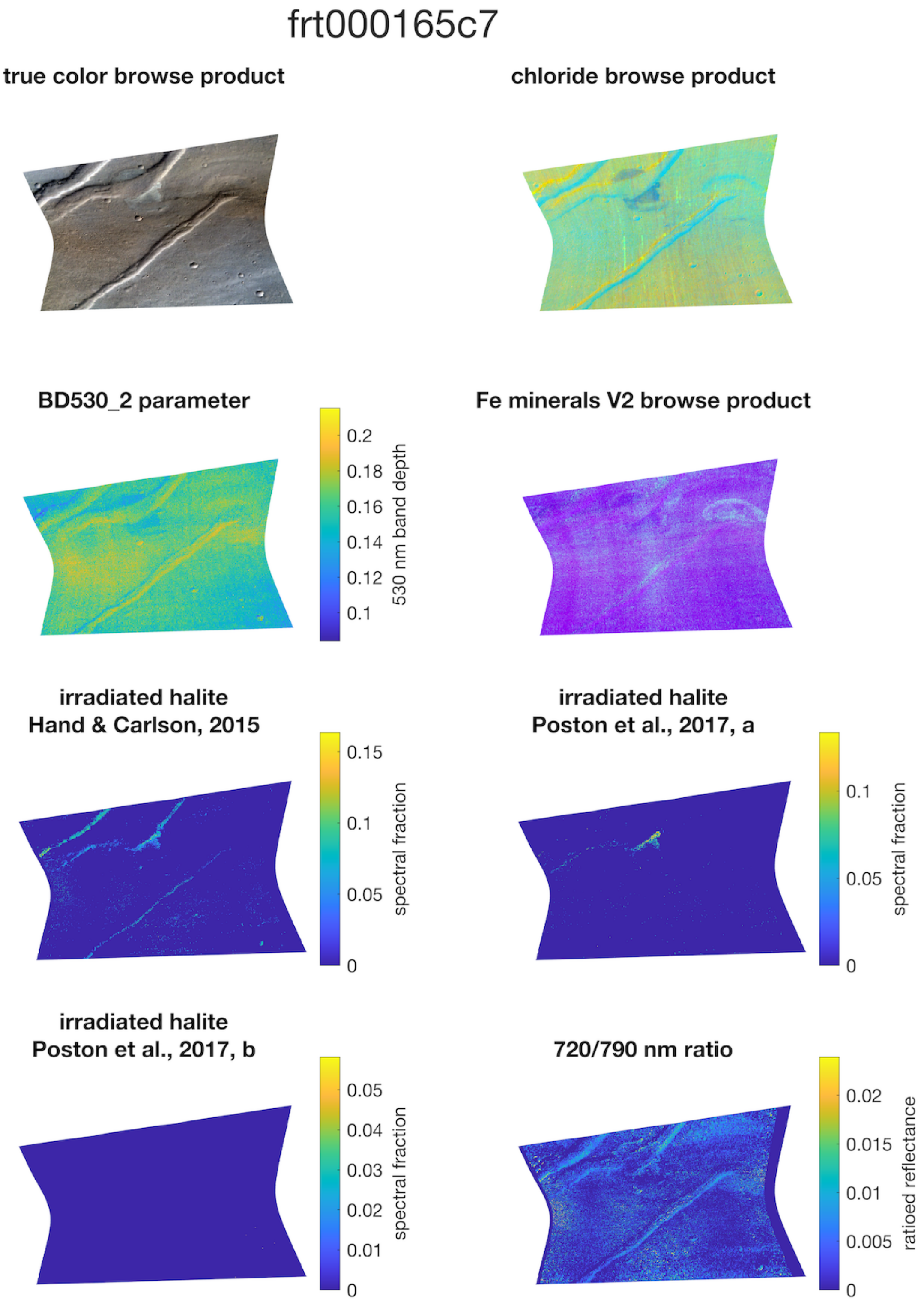
frt000165c7
true color browse product
chloride browse product
BD530_2 parameter
530 nm band depth
0.2
0.18
0.16
0.14
0.12
0.1
Fe minerals V2 browse product
irradiated halite
Hand & Carlson, 2015
spectral fraction
0.15
0.1
0.05
0
irradiated halite
Poston et al., 2017, a
spectral fraction
0.1
0.05
0
irradiated halite
Poston et al., 2017, b
spectral fraction
0.05
0.04
0.03
0.02
0.01
0
720/790 nm ratio
ratioed reflectance
0.02
0.015
0.01
0.005
0

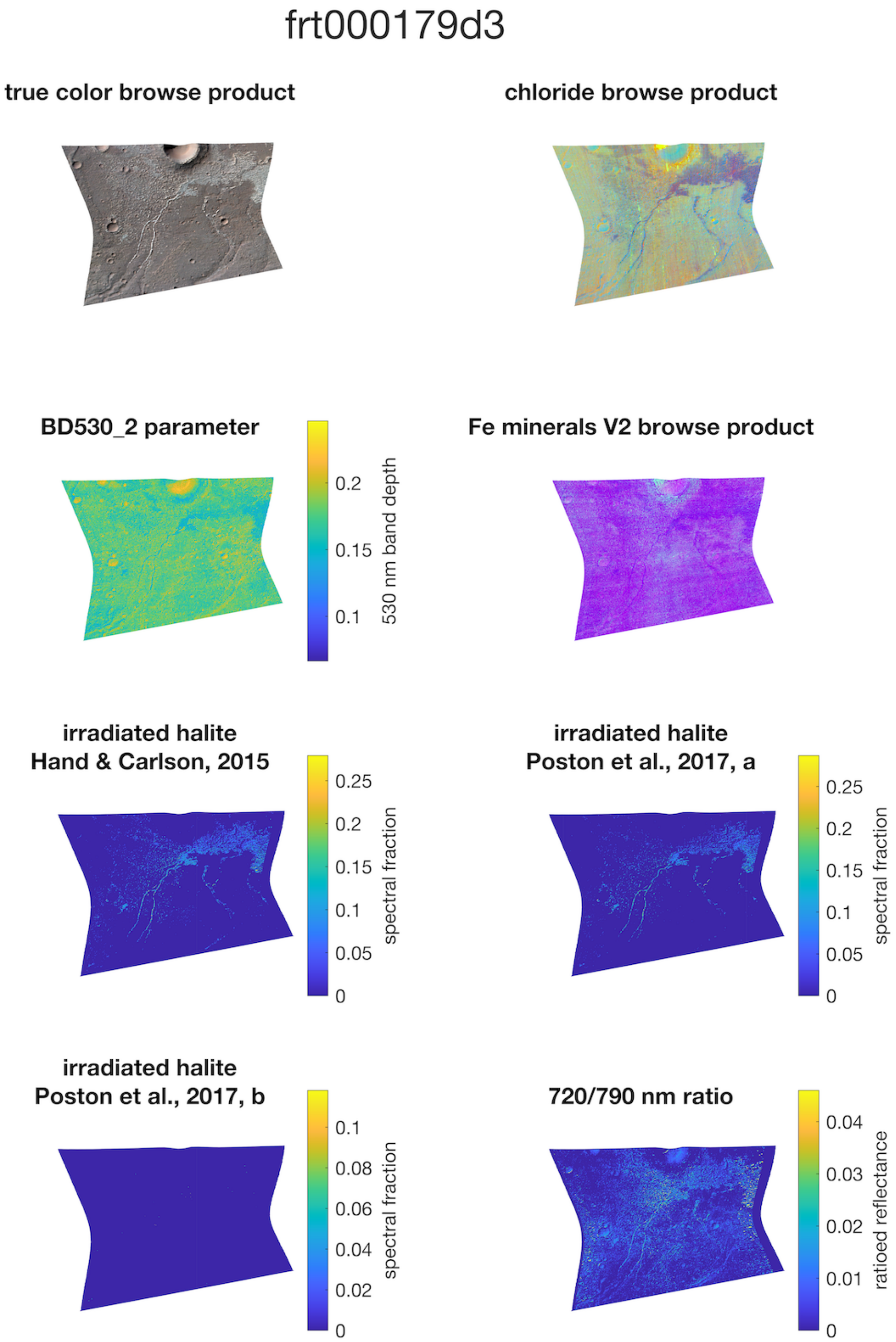
frt000179d3
true color browse product
chloride browse product
BD530_2 parameter
0.2
0.15
0.1
530 nm band depth
Fe minerals V2 browse product
irradiated halite
Hand & Carlson, 2015
0.25
0.2
0.15
0.1
0.05
0
spectral fraction
irradiated halite
Poston et al., 2017, a
0.25
0.2
0.15
0.1
0.05
0
spectral fraction
irradiated halite
Poston et al., 2017, b
0.1
0.08
0.06
0.04
0.02
0
spectral fraction
720/790 nm ratio
0.04
0.03
0.02
0.01
0
ratioed reflectance

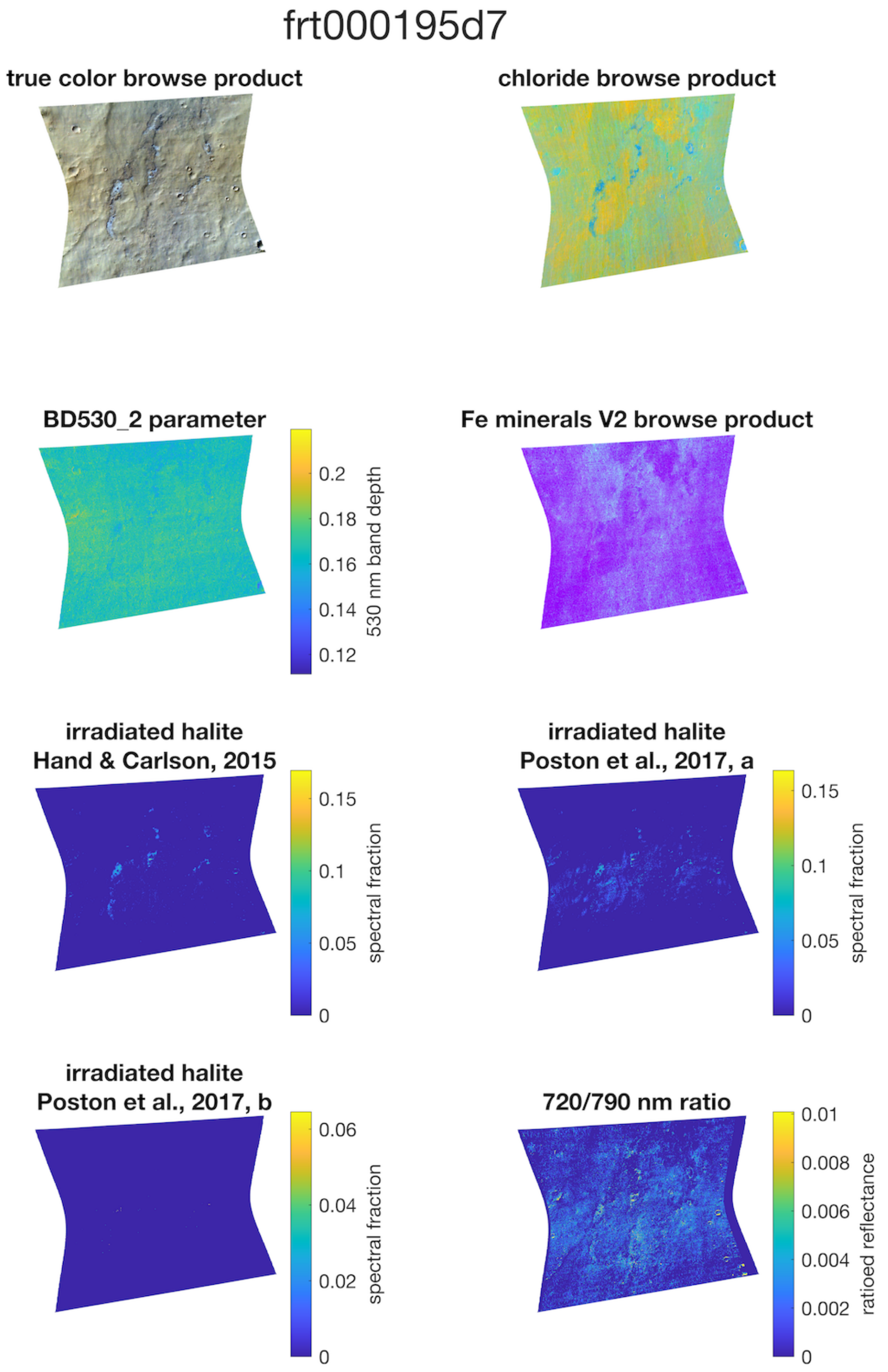
frt000195d7
true color browse product
chloride browse product
BD530_2 parameter
0.2
0.18
0.16
0.14
0.12
530 nm band depth
Fe minerals V2 browse product
irradiated halite
Hand & Carlson, 2015
0.15
0.1
0.05
0
spectral fraction
irradiated halite
Poston et al., 2017, a
0.15
0.1
0.05
0
spectral fraction
irradiated halite
Poston et al., 2017, b
0.06
0.04
0.02
0
spectral fraction
720/790 nm ratio
0.01
0.008
0.006
0.004
0.002
0
ratioed reflectance

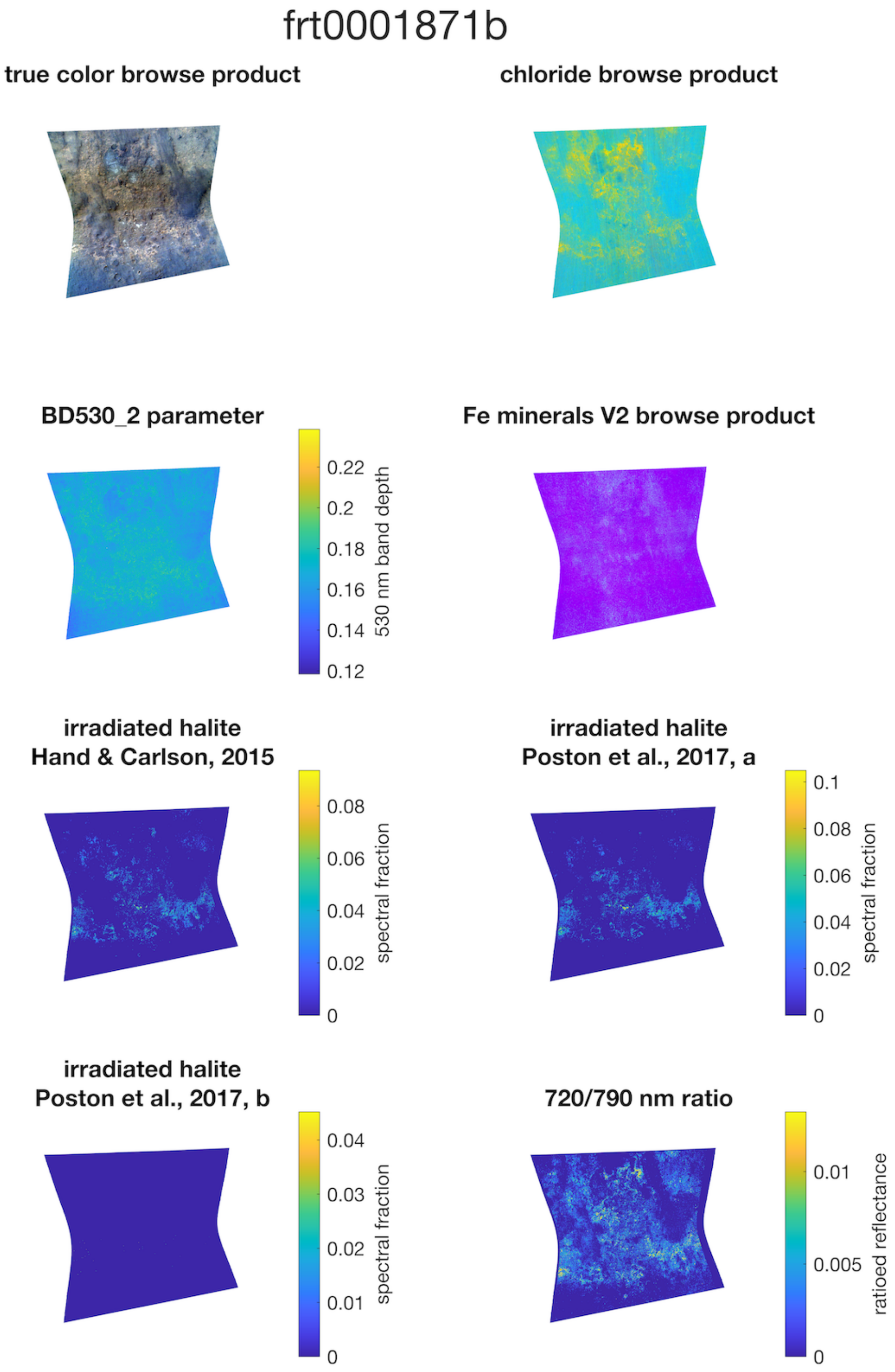

frt0001871b
true color browse product
chloride browse product
BD530_2 parameter
530 nm band depth
0.22
0.2
0.18
0.16
0.14
0.12
Fe minerals V2 browse product
irradiated halite
Hand & Carlson, 2015
spectral fraction
0.08
0.06
0.04
0.02
0
irradiated halite
Poston et al., 2017, a
spectral fraction
0.1
0.08
0.06
0.04
0.02
0
irradiated halite
Poston et al., 2017, b
spectral fraction
0.04
0.03
0.02
0.01
0
720/790 nm ratio
ratioed reflectance
0.01
0.005
0

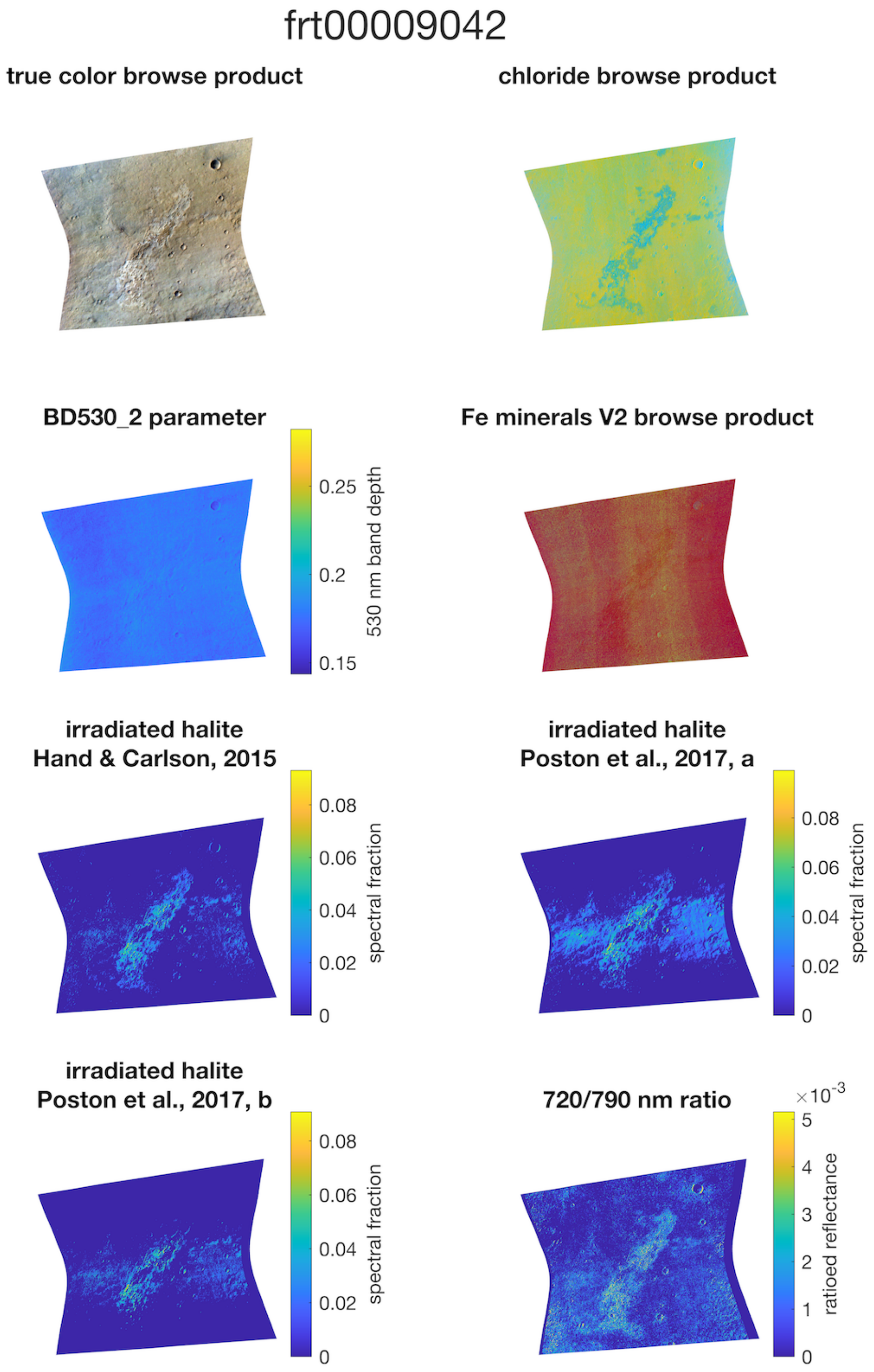
frt00009042
true color browse product
chloride browse product
BD530_2 parameter
530 nm band depth
0.15
0.2
0.25
Fe minerals V2 browse product
irradiated halite
Hand & Carlson, 2015
spectral fraction
0
0.02
0.04
0.06
0.08
irradiated halite
Poston et al., 2017, a
spectral fraction
0
0.02
0.04
0.06
0.08
irradiated halite
Poston et al., 2017, b
spectral fraction
0
0.02
0.04
0.06
0.08
720/790 nm ratio
×10^-3
ratioed reflectance
0
1
2
3
4
5

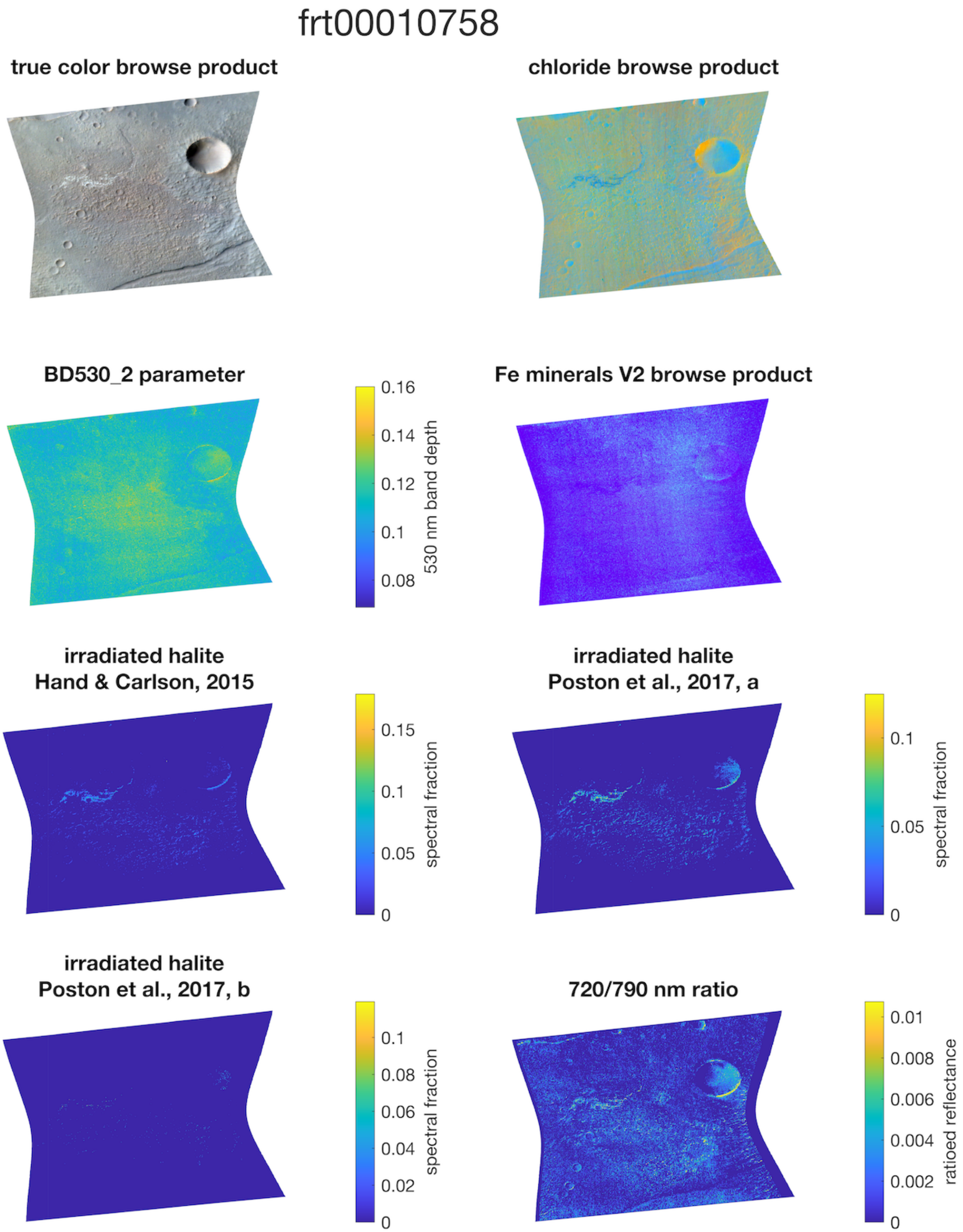

frt00010758
true color browse product
chloride browse product
BD530_2 parameter
0.16
0.14
0.12
0.1
0.08
530 nm band depth
Fe minerals V2 browse product
irradiated halite
Hand & Carlson, 2015
0.15
0.1
0.05
0
spectral fraction
irradiated halite
Poston et al., 2017, a
0.1
0.05
0
spectral fraction
irradiated halite
Poston et al., 2017, b
0.1
0.08
0.06
0.04
0.02
0
spectral fraction
720/790 nm ratio
0.01
0.008
0.006
0.004
0.002
0
ratioed reflectance

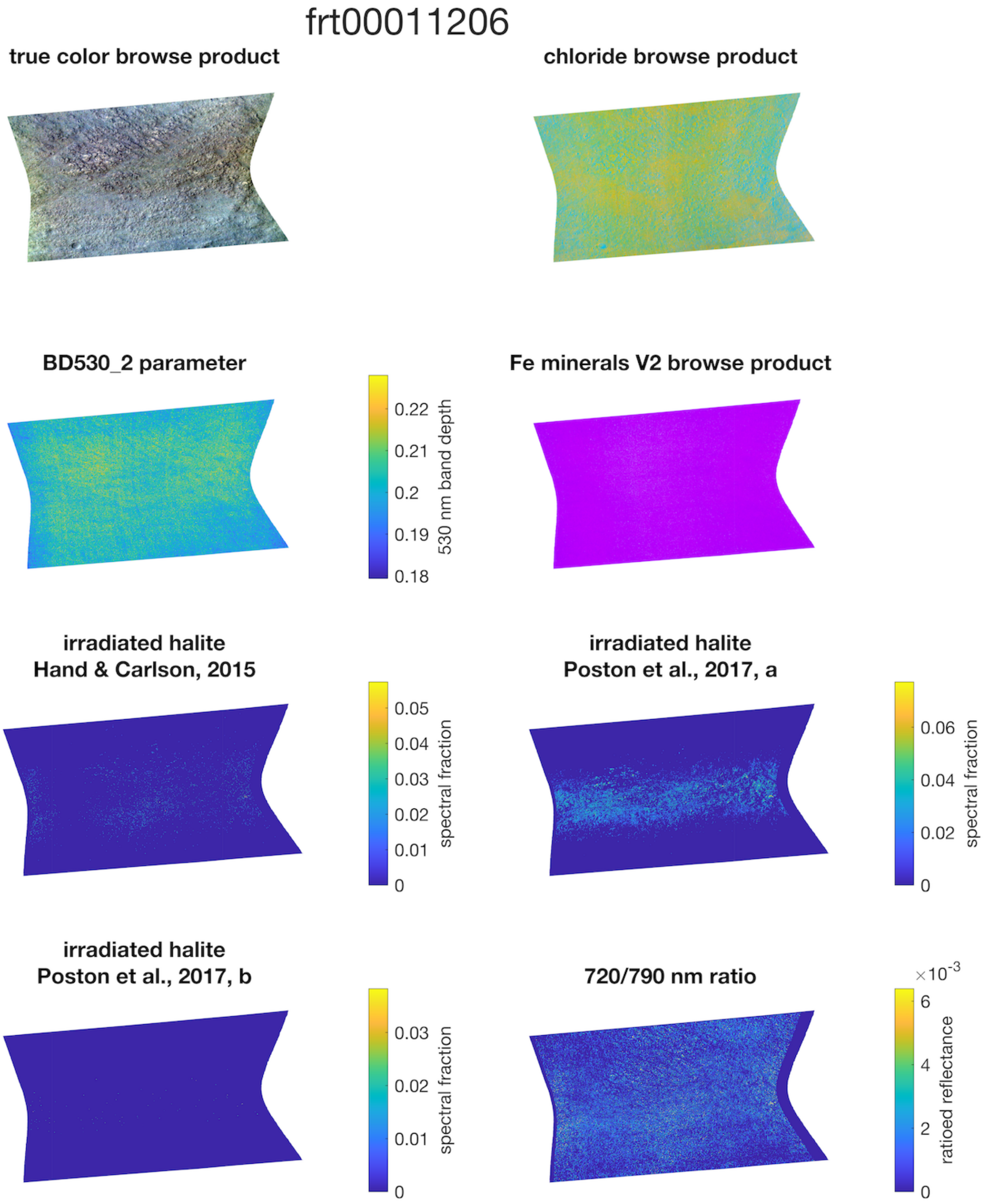
frt00011206
true color browse product
chloride browse product
BD530_2 parameter
0.22
0.21
0.2
0.19
0.18
530 nm band depth
Fe minerals V2 browse product
irradiated halite
Hand & Carlson, 2015
0.05
0.04
0.03
0.02
0.01
0
spectral fraction
irradiated halite
Poston et al., 2017, a
0.06
0.04
0.02
0
spectral fraction
irradiated halite
Poston et al., 2017, b
0.03
0.02
0.01
0
spectral fraction
720/790 nm ratio
×10^-3
6
4
2
0
ratioed reflectance

frt00011228

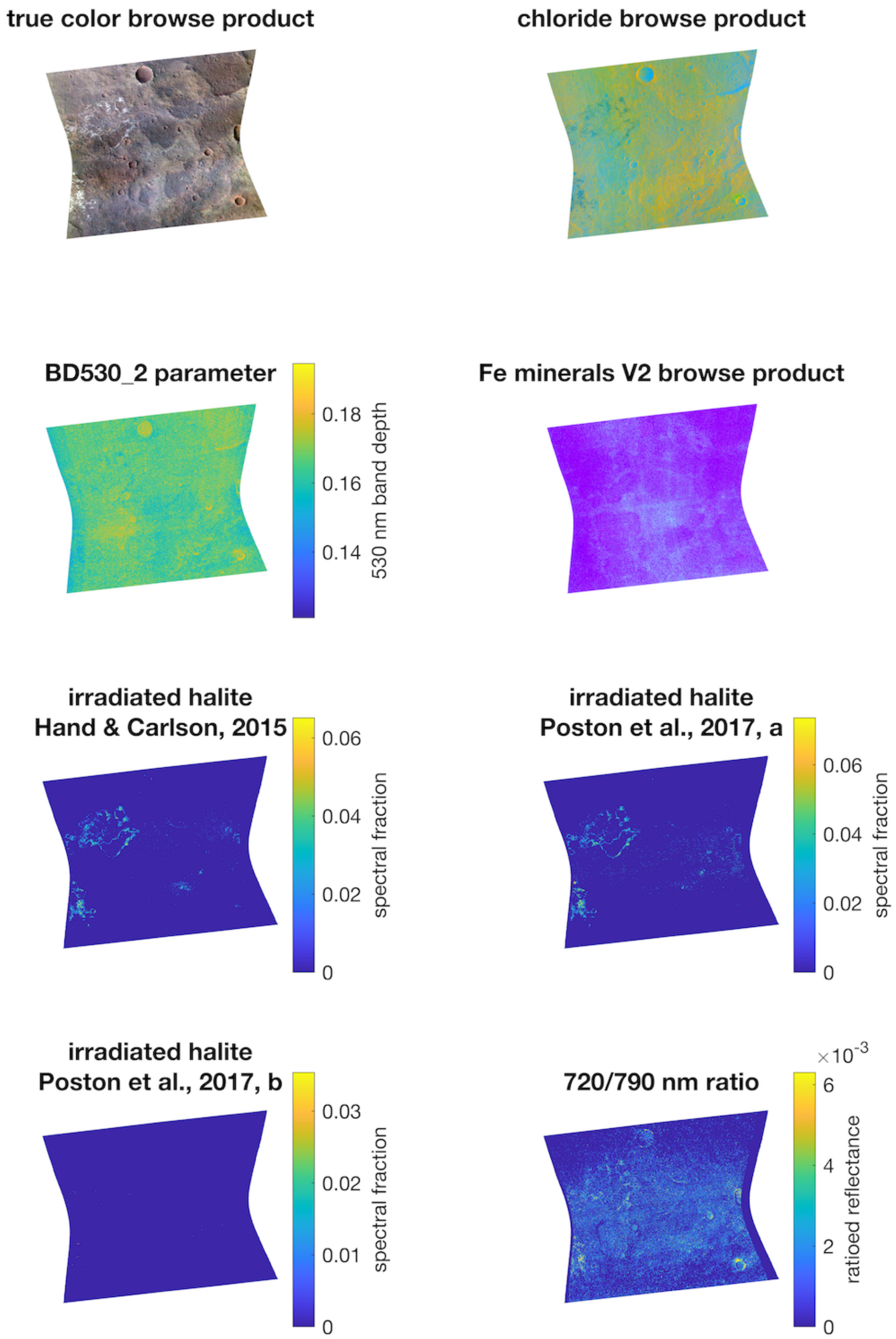

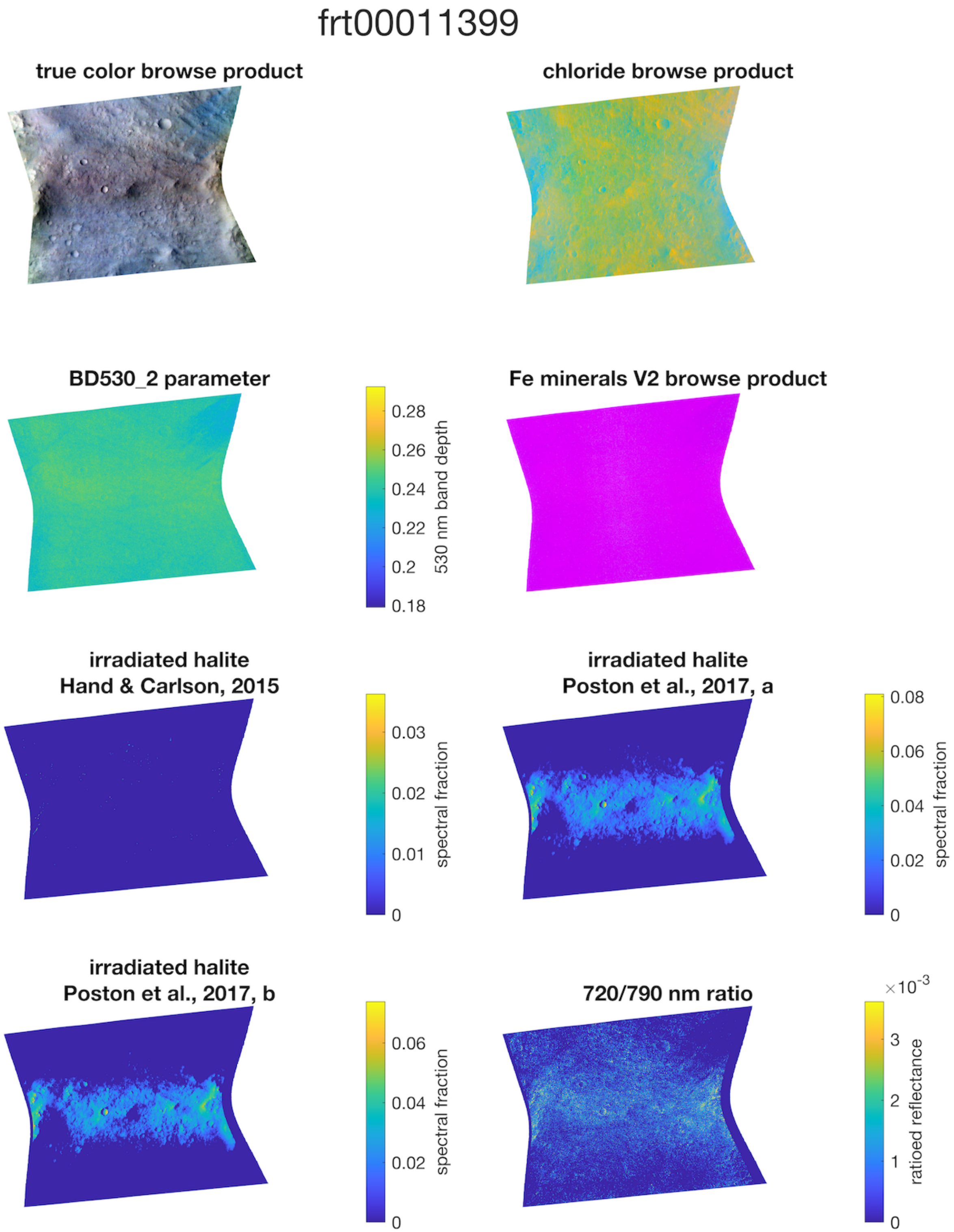
frt00011399
true color browse product
chloride browse product
BD530_2 parameter
0.28
0.26
0.24
0.22
0.2
0.18
530 nm band depth
Fe minerals V2 browse product
irradiated halite
Hand & Carlson, 2015
0.03
0.02
0.01
0
spectral fraction
irradiated halite
Poston et al., 2017, a
0.08
0.06
0.04
0.02
0
spectral fraction
irradiated halite
Poston et al., 2017, b
0.06
0.04
0.02
0
spectral fraction
720/790 nm ratio
×10^-3
3
2
1
0
ratioed reflectance

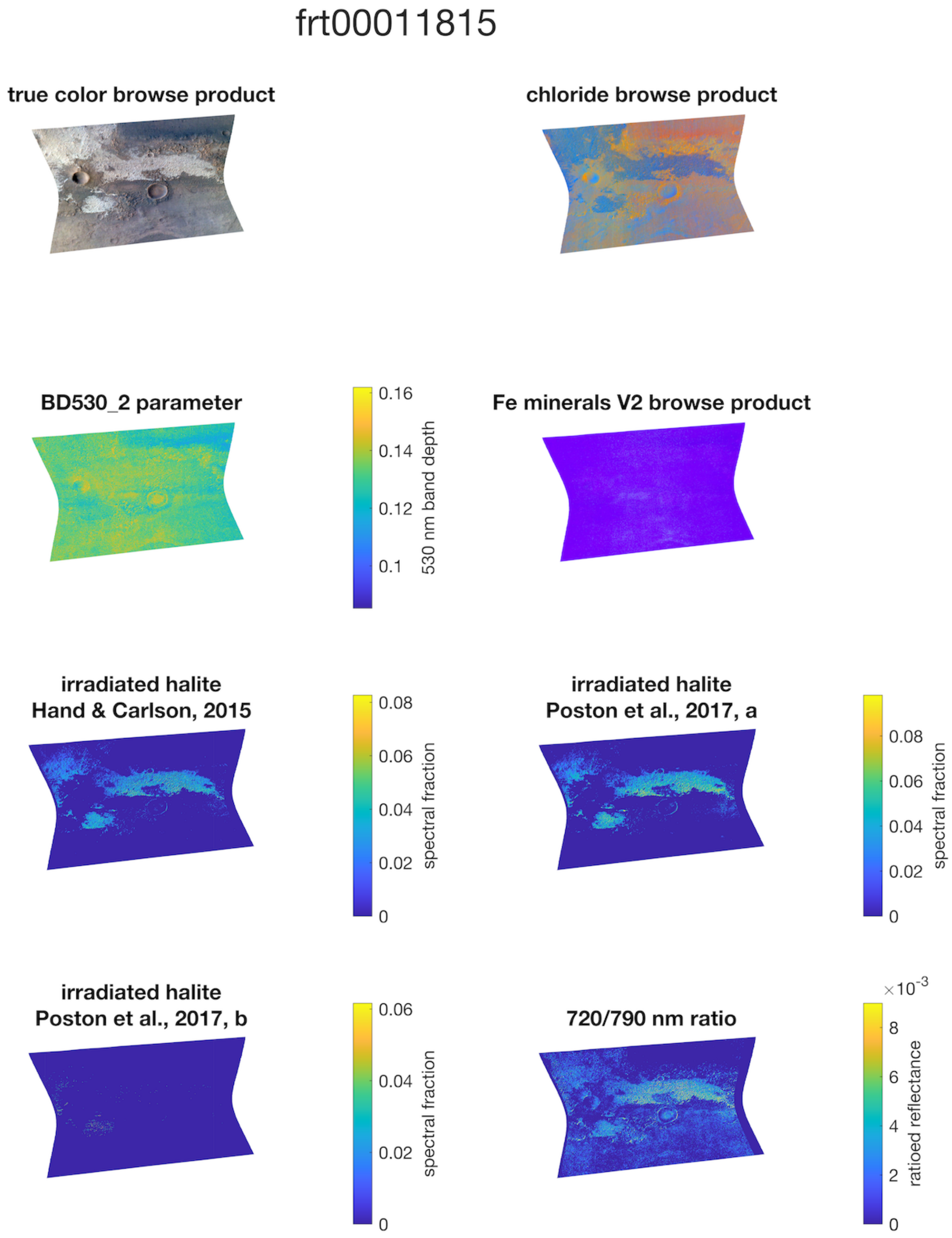
frt00011815
true color browse product
chloride browse product
BD530_2 parameter
0.16
0.14
0.12
0.1
530 nm band depth
Fe minerals V2 browse product
irradiated halite
Hand & Carlson, 2015
0.08
0.06
0.04
0.02
0
spectral fraction
irradiated halite
Poston et al., 2017, a
0.08
0.06
0.04
0.02
0
spectral fraction
irradiated halite
Poston et al., 2017, b
0.06
0.04
0.02
0
spectral fraction
720/790 nm ratio
×10-3
8
6
4
2
0
ratioed reflectance

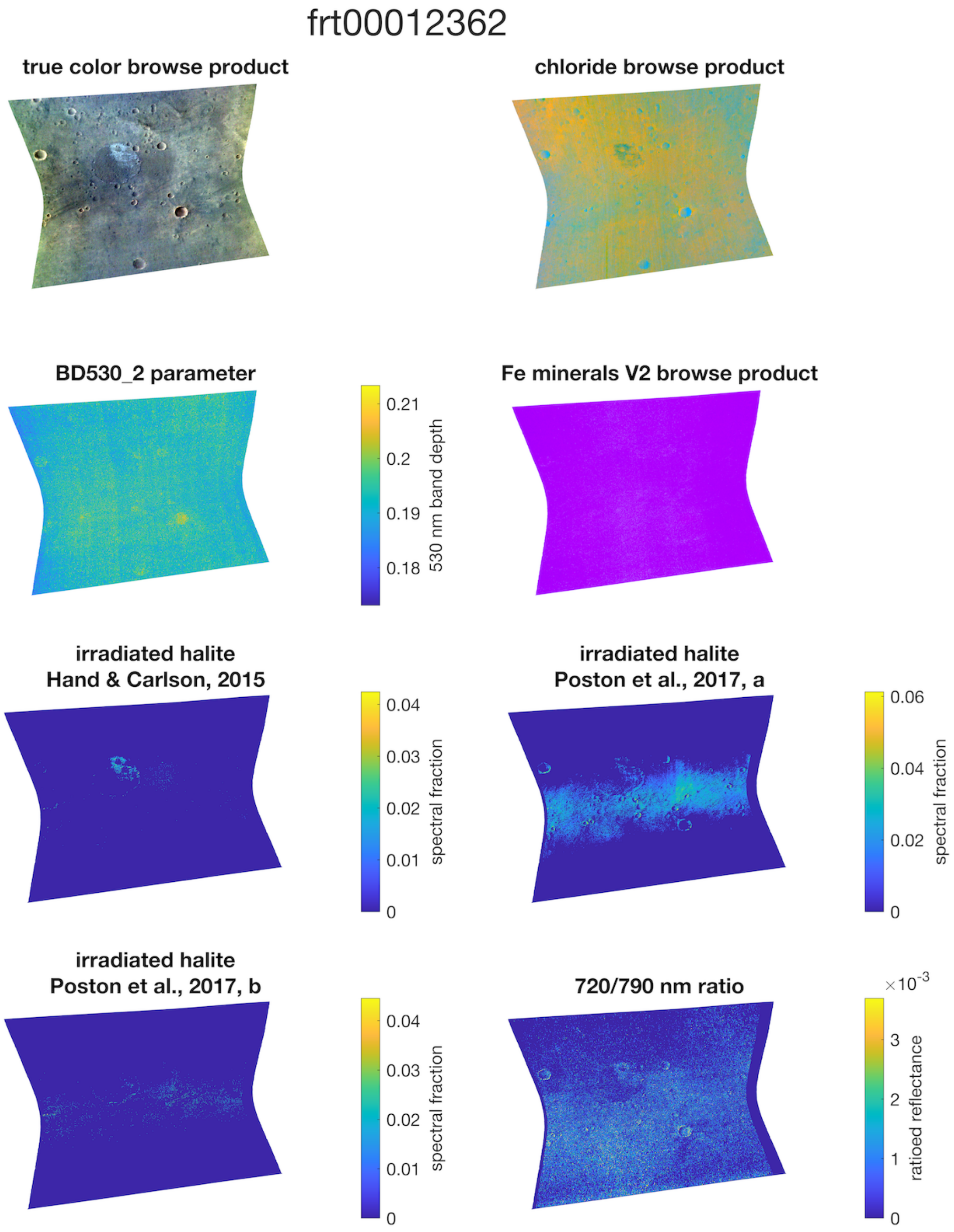
frt00012362
true color browse product
chloride browse product
BD530_2 parameter
530 nm band depth
0.21
0.2
0.19
0.18
Fe minerals V2 browse product
irradiated halite
Hand & Carlson, 2015
spectral fraction
0.04
0.03
0.02
0.01
0
irradiated halite
Poston et al., 2017, a
spectral fraction
0.06
0.04
0.02
0
irradiated halite
Poston et al., 2017, b
spectral fraction
0.04
0.03
0.02
0.01
0
720/790 nm ratio
×10-3
ratioed reflectance
3
2
1
0

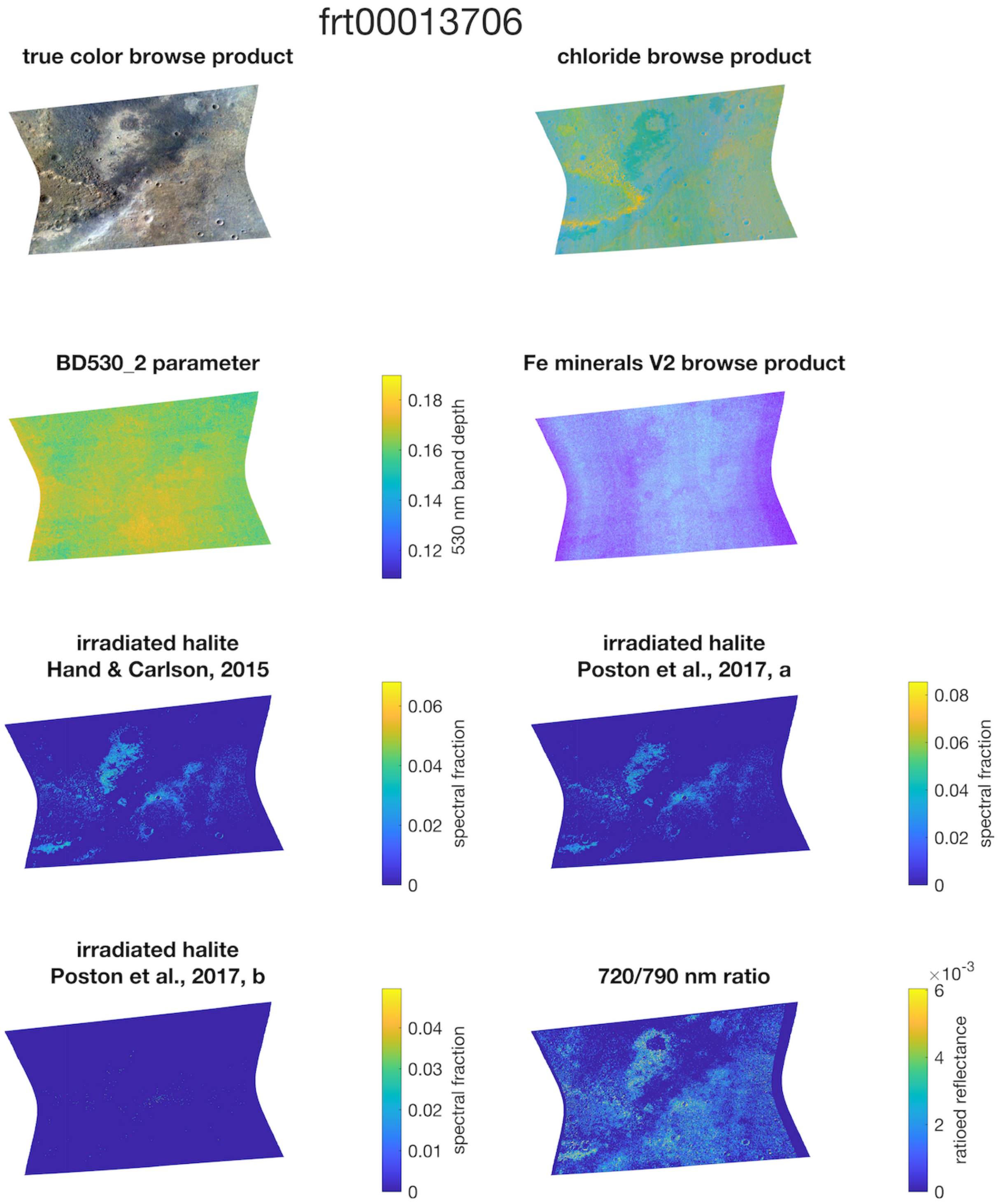
frt00013706
true color browse product
chloride browse product
BD530_2 parameter
0.18
0.16
0.14
0.12
530 nm band depth
Fe minerals V2 browse product
irradiated halite
Hand & Carlson, 2015
0.06
0.04
0.02
0
spectral fraction
irradiated halite
Poston et al., 2017, a
0.08
0.06
0.04
0.02
0
spectral fraction
irradiated halite
Poston et al., 2017, b
0.04
0.03
0.02
0.01
0
spectral fraction
720/790 nm ratio
×10^-3
6
4
2
0
ratioed reflectance

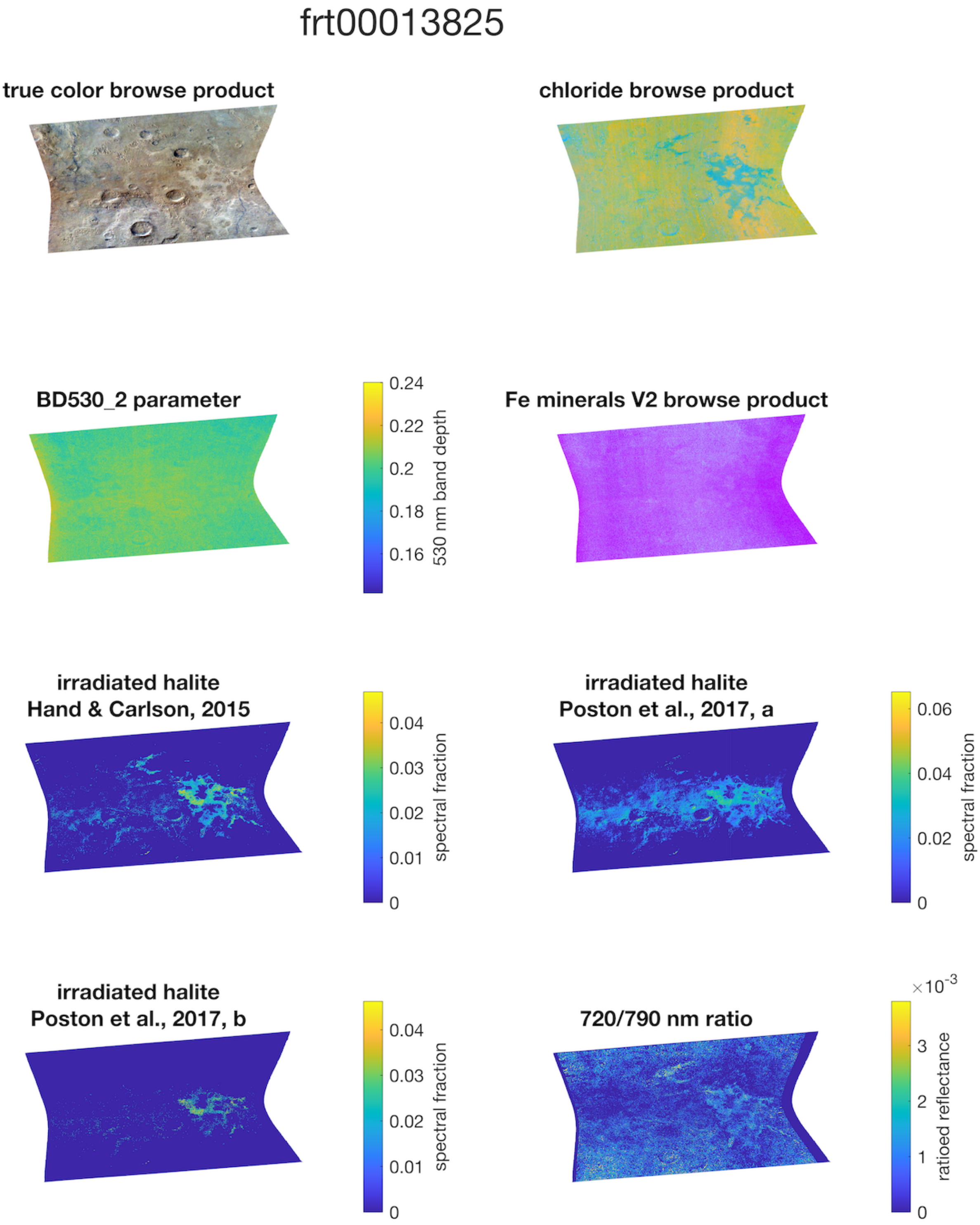

frt00013825
true color browse product
chloride browse product
BD530_2 parameter
0.24
0.22
0.2
0.18
0.16
530 nm band depth
Fe minerals V2 browse product
irradiated halite
Hand & Carlson, 2015
0.04
0.03
0.02
0.01
0
spectral fraction
irradiated halite
Poston et al., 2017, a
0.06
0.04
0.02
0
spectral fraction
irradiated halite
Poston et al., 2017, b
0.04
0.03
0.02
0.01
0
spectral fraction
720/790 nm ratio
×10^-3
3
2
1
0
ratioed reflectance

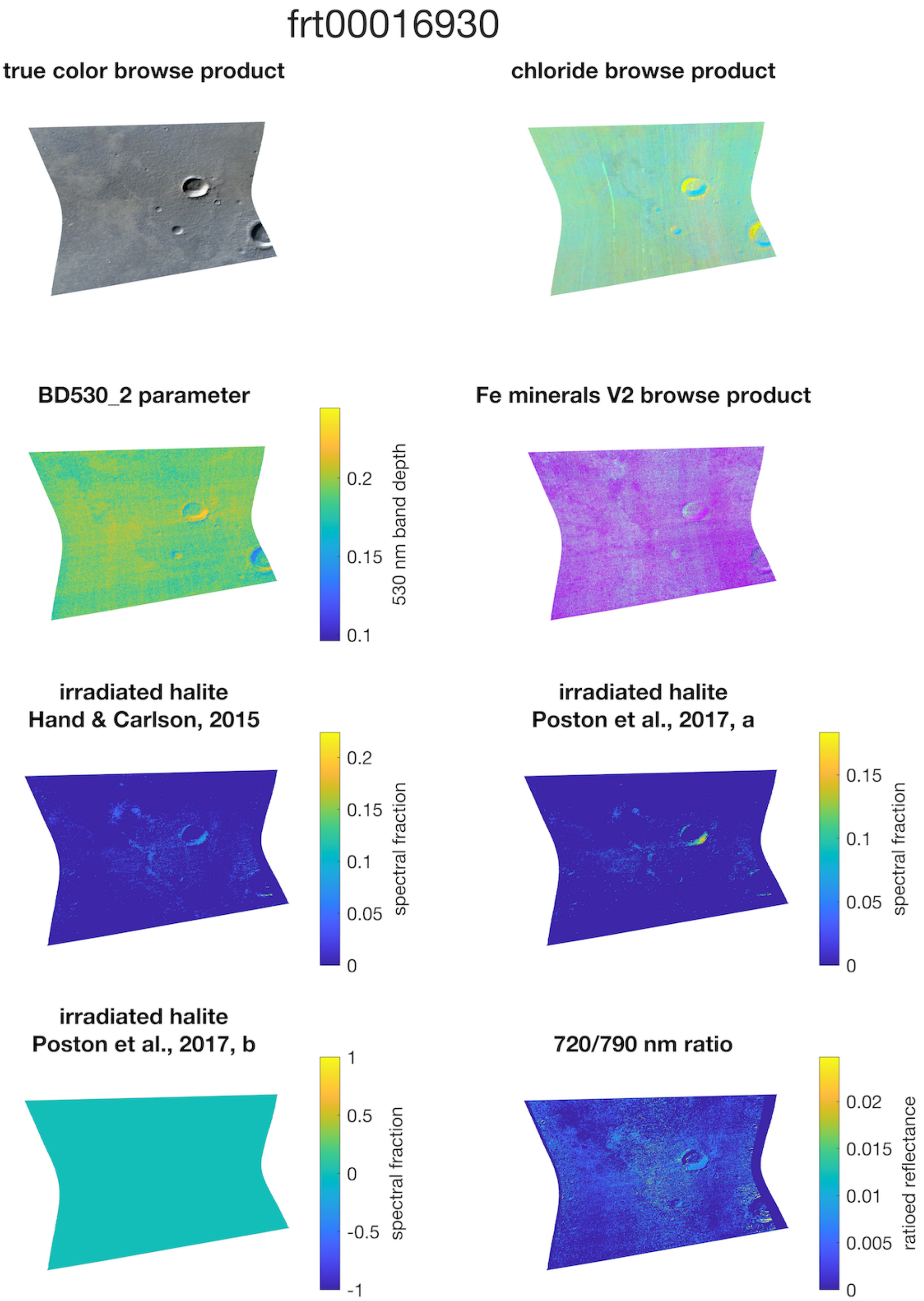
frt00016930
true color browse product
chloride browse product
BD530_2 parameter
530 nm band depth
0.2
0.15
0.1
Fe minerals V2 browse product
irradiated halite
Hand & Carlson, 2015
spectral fraction
0.2
0.15
0.1
0.05
0
irradiated halite
Poston et al., 2017, a
spectral fraction
0.15
0.1
0.05
0
irradiated halite
Poston et al., 2017, b
spectral fraction
1
0.5
0
-0.5
-1
720/790 nm ratio
ratioed reflectance
0.02
0.015
0.01
0.005
0

hrl0000cee3

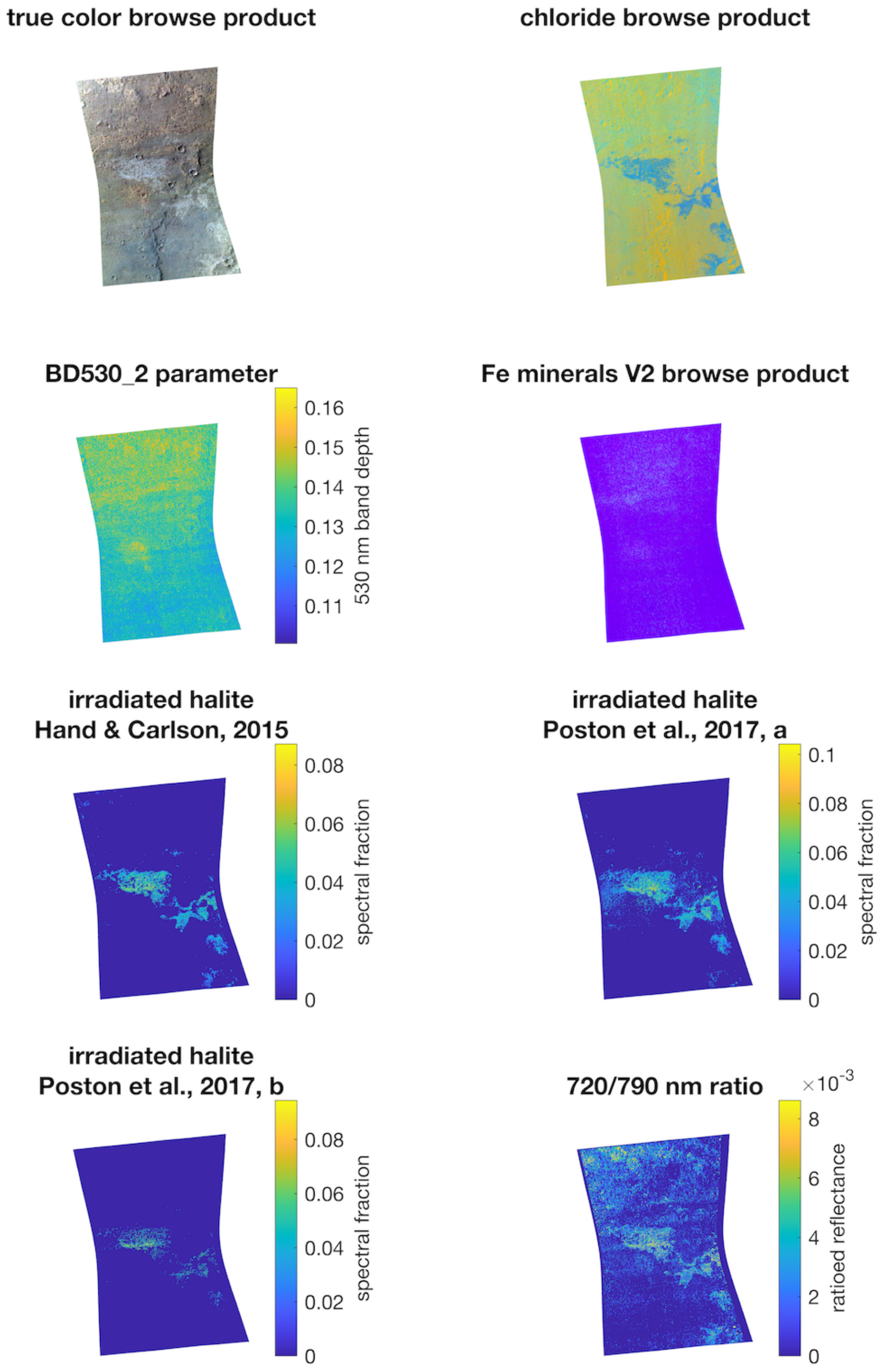

hrl0000cf71

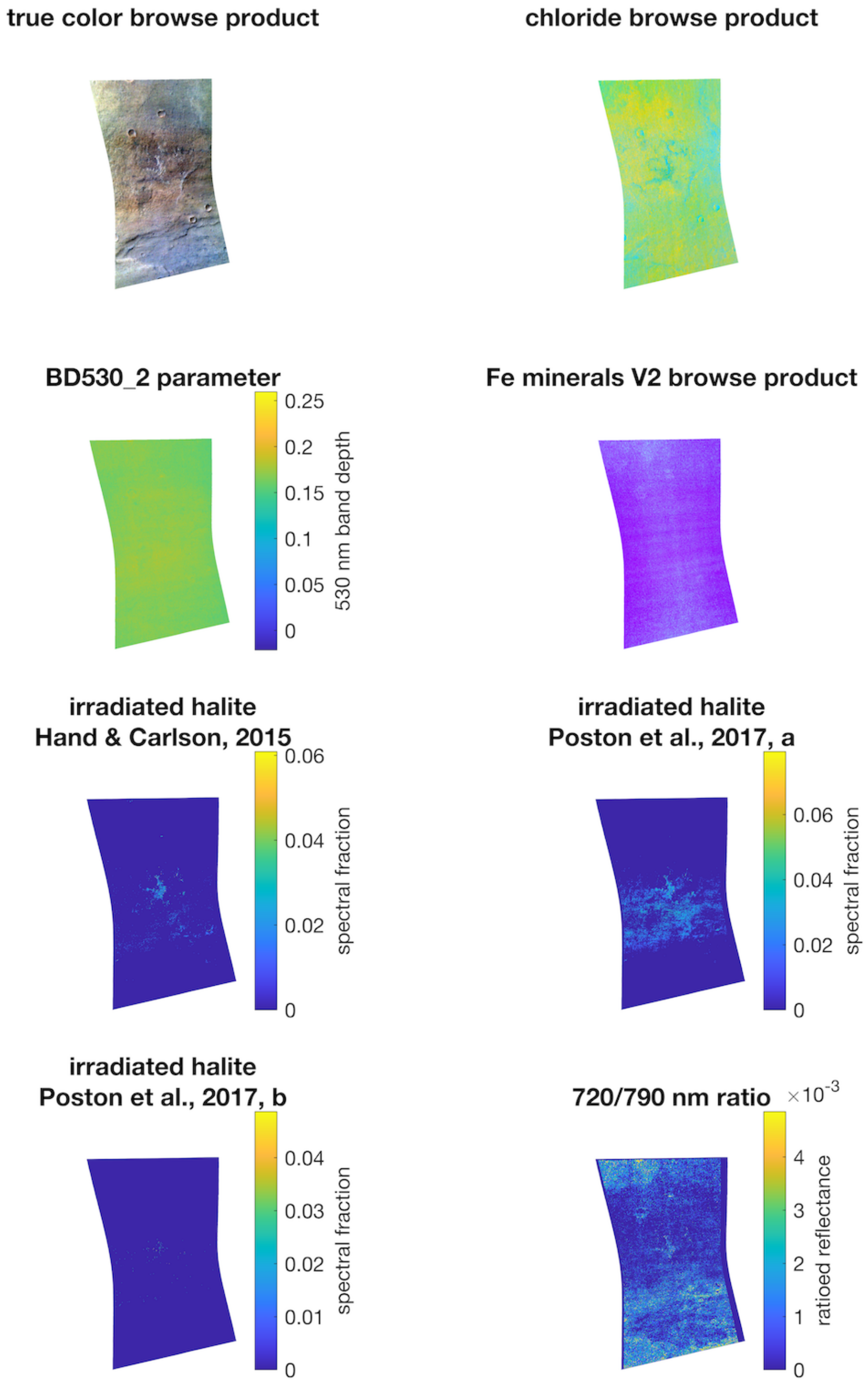

hrl0000d1c7

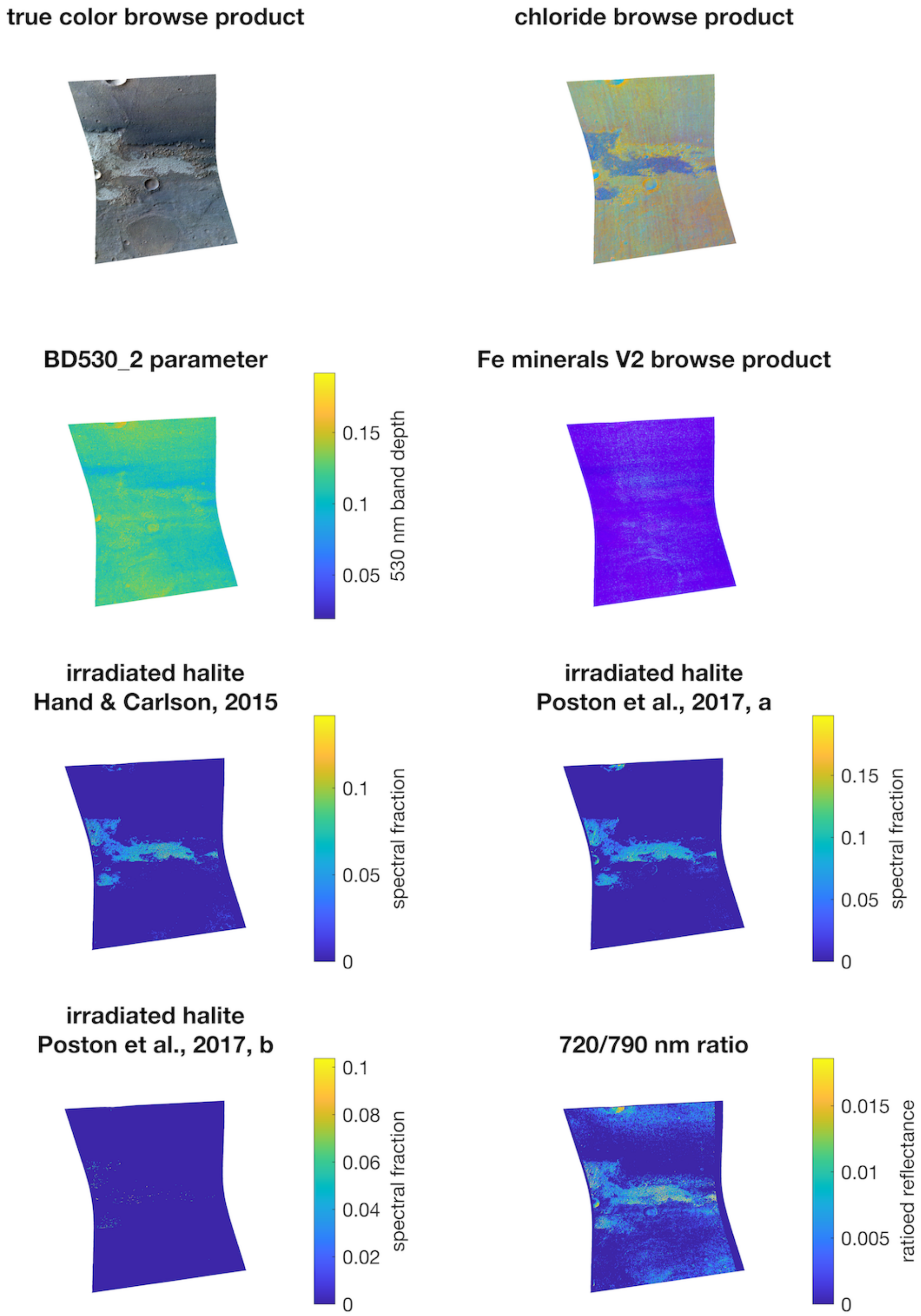

hrl0000d2f1

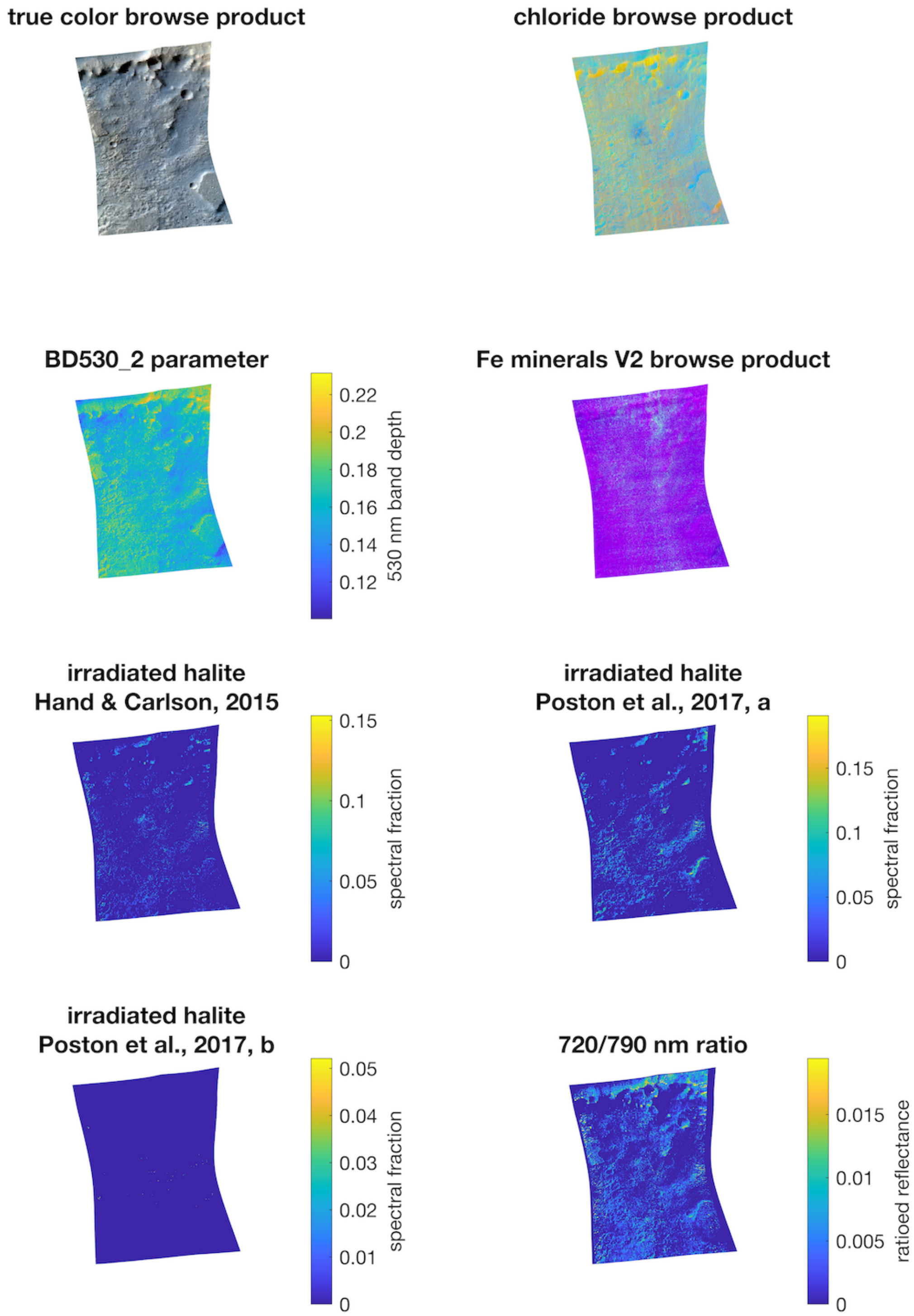

hrl0000d086

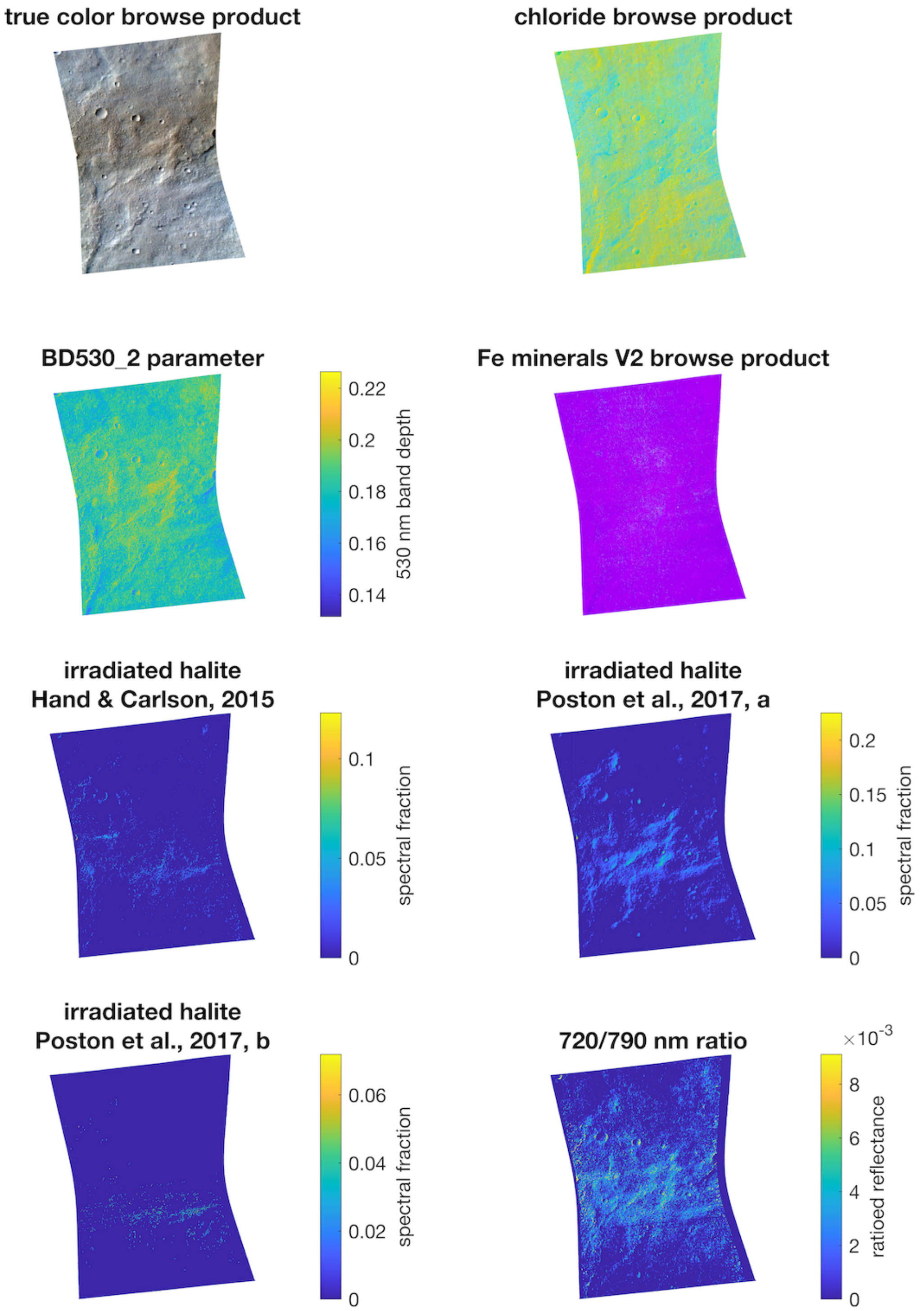

hrl0001b96e

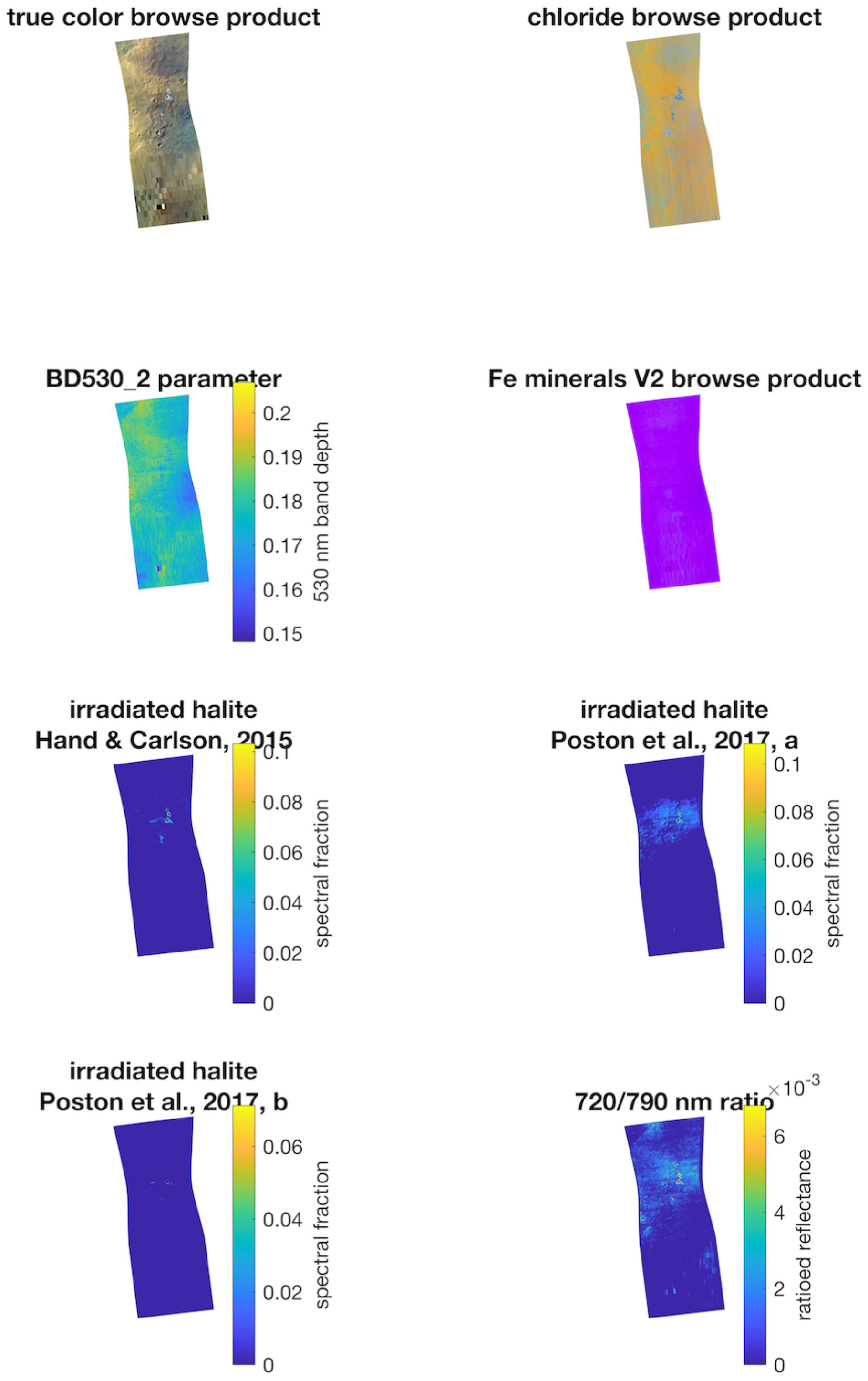

hrl00007c95

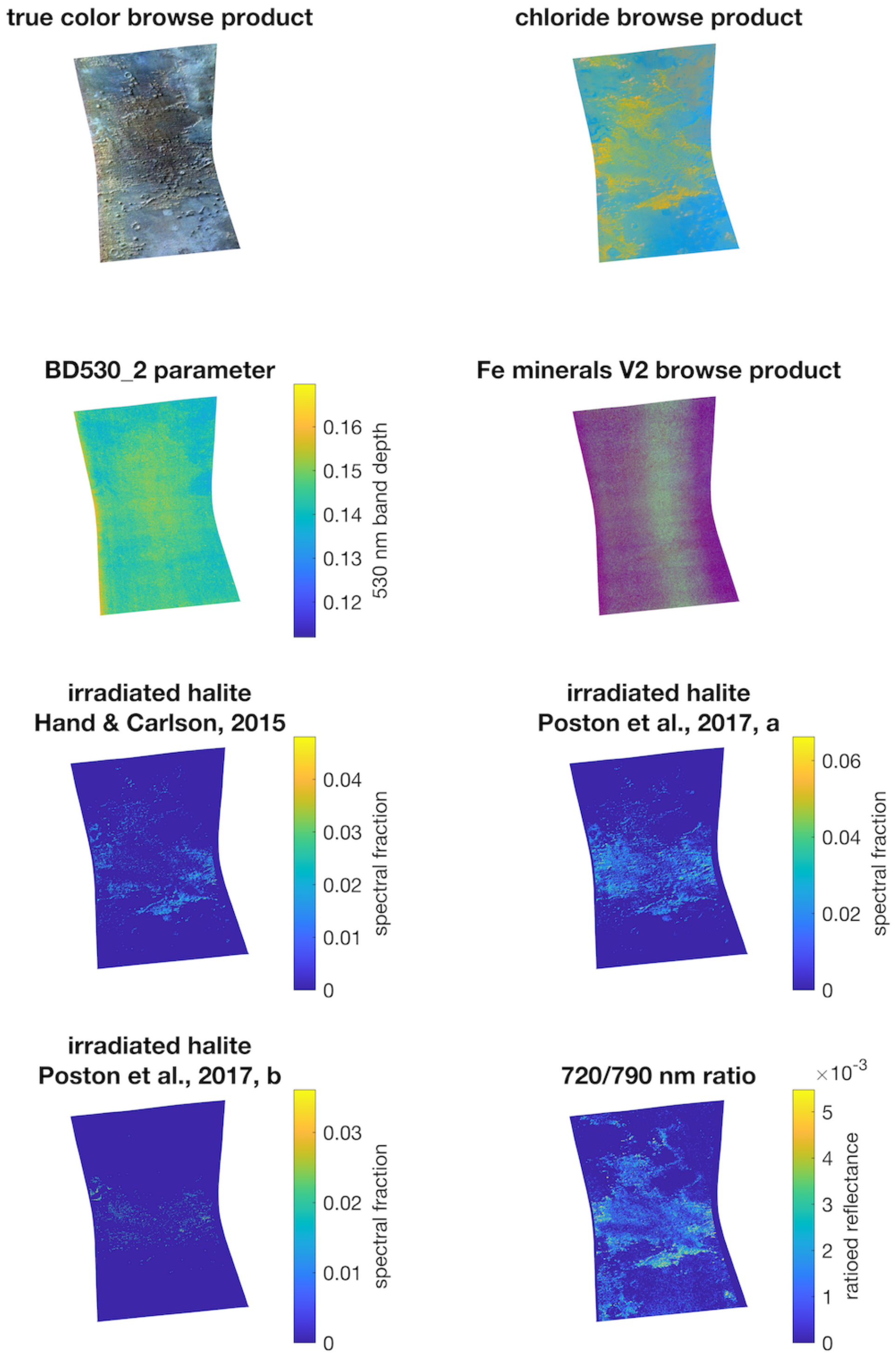

hrl000082da

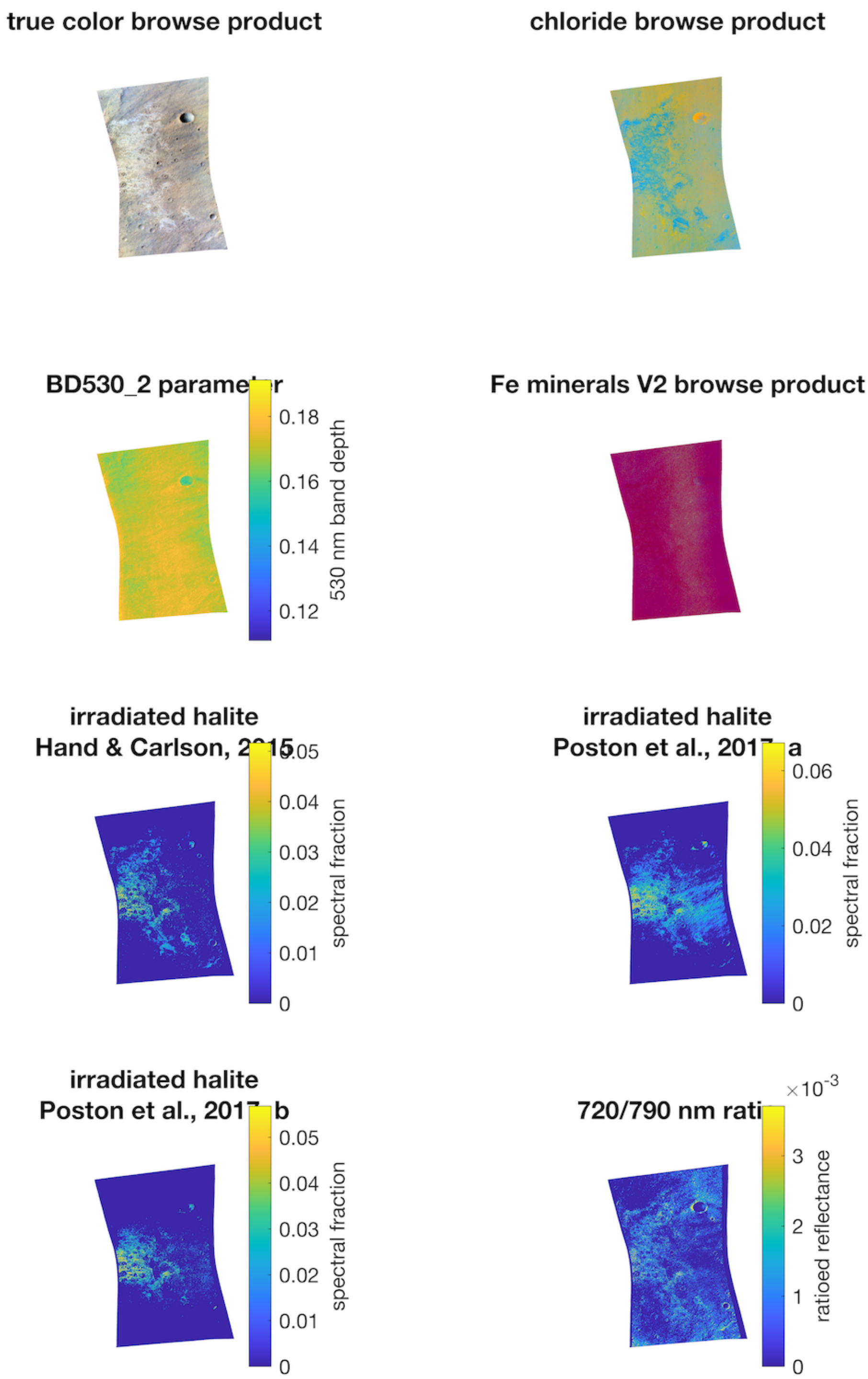

hrl0001082c

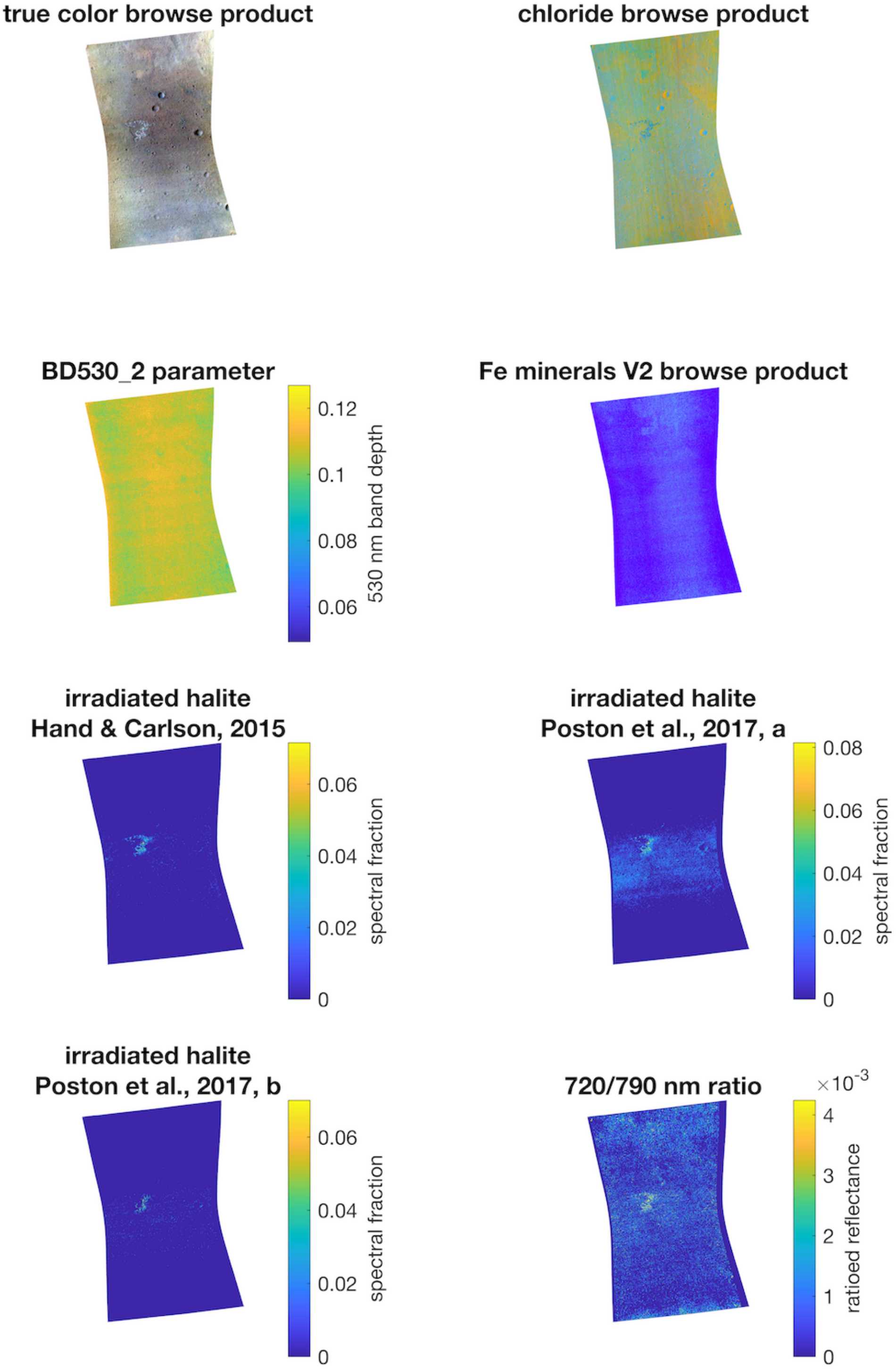

hrl00013922

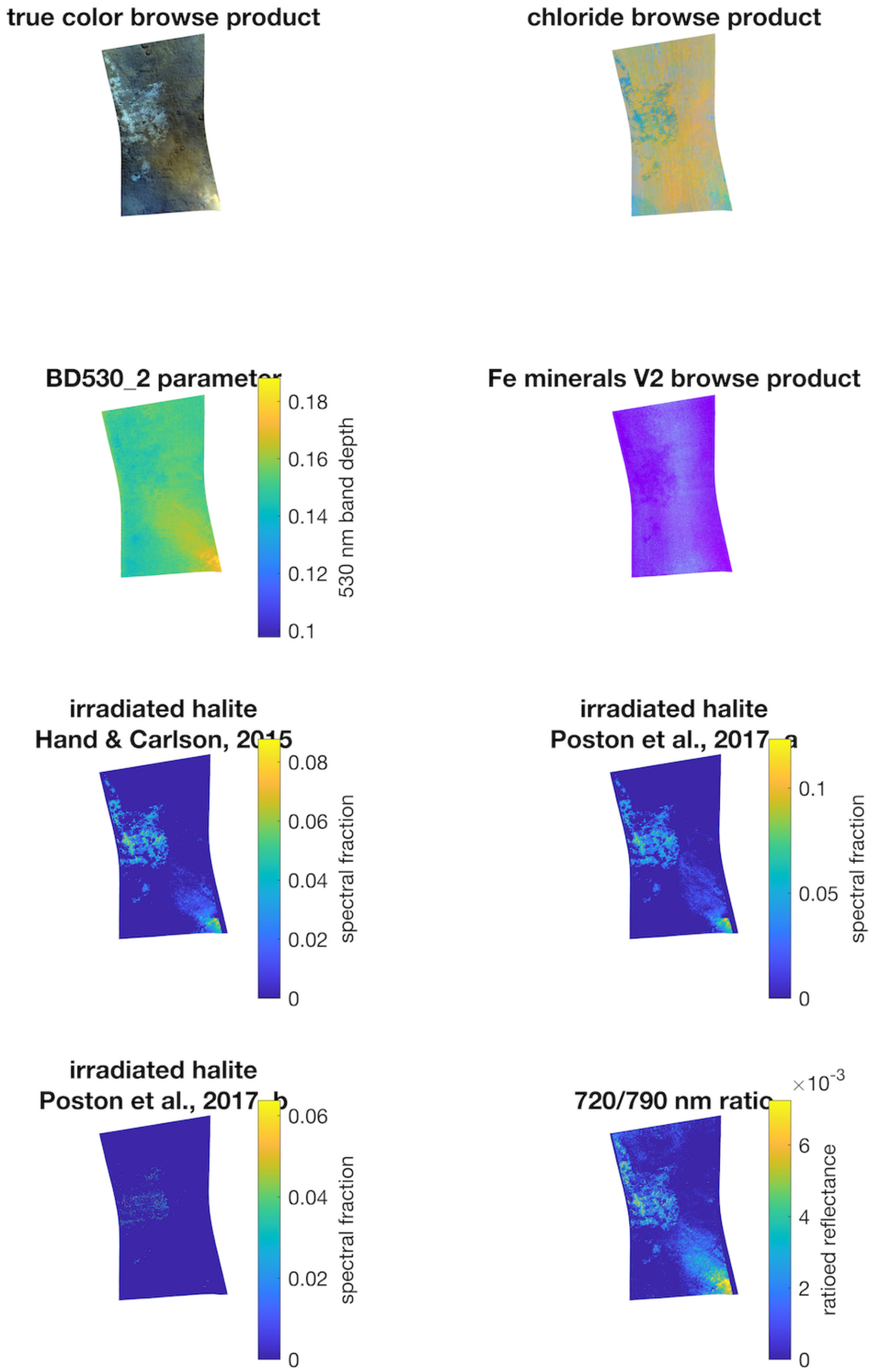

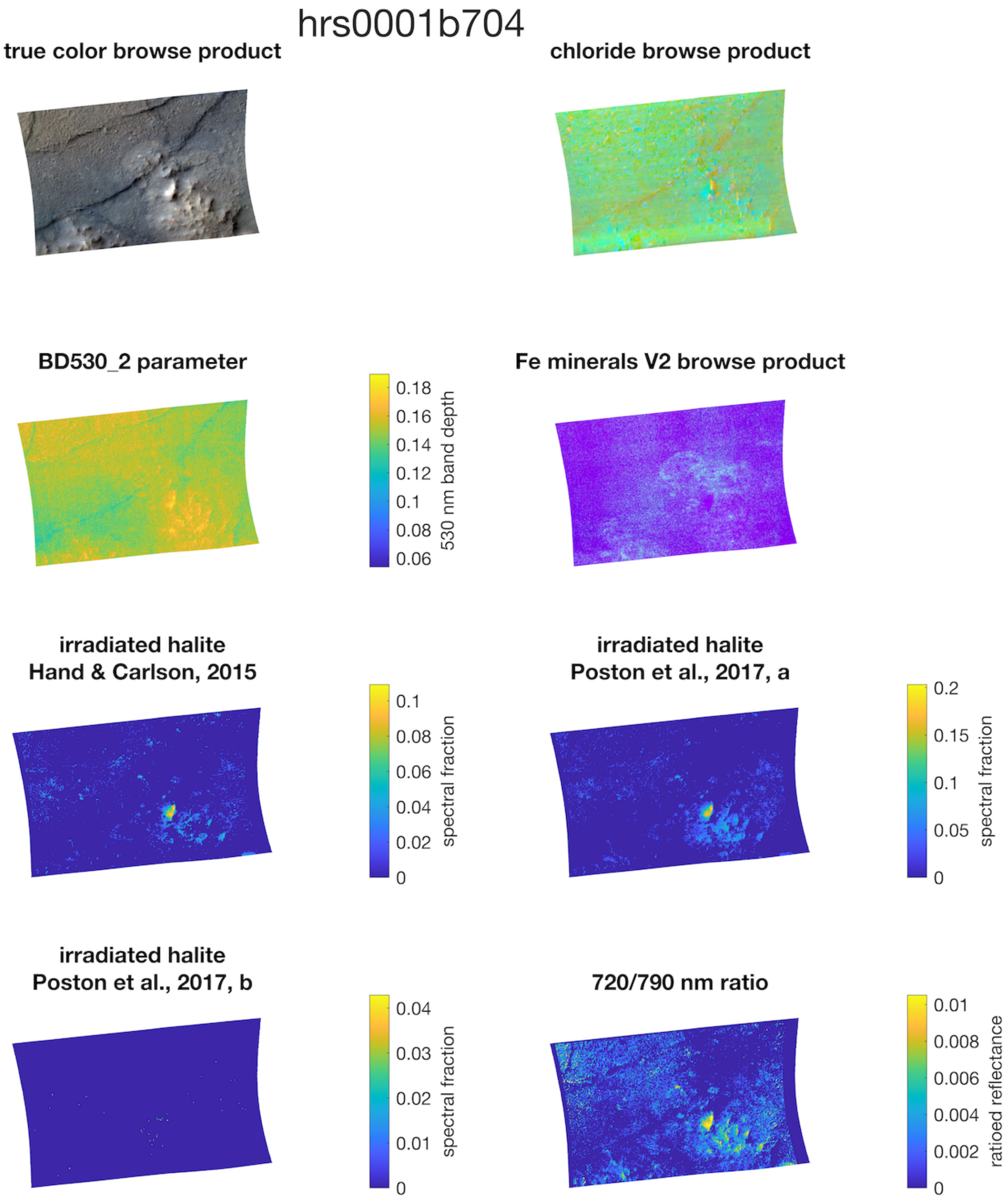
hrs0001b704
true color browse product
chloride browse product
BD530_2 parameter
530 nm band depth
0.18
0.16
0.14
0.12
0.1
0.08
0.06
Fe minerals V2 browse product
irradiated halite
Hand & Carlson, 2015
spectral fraction
0.1
0.08
0.06
0.04
0.02
0
irradiated halite
Poston et al., 2017, a
spectral fraction
0.2
0.15
0.1
0.05
0
irradiated halite
Poston et al., 2017, b
spectral fraction
0.04
0.03
0.02
0.01
0
720/790 nm ratio
ratioed reflectance
0.01
0.008
0.006
0.004
0.002
0

hrs00011174

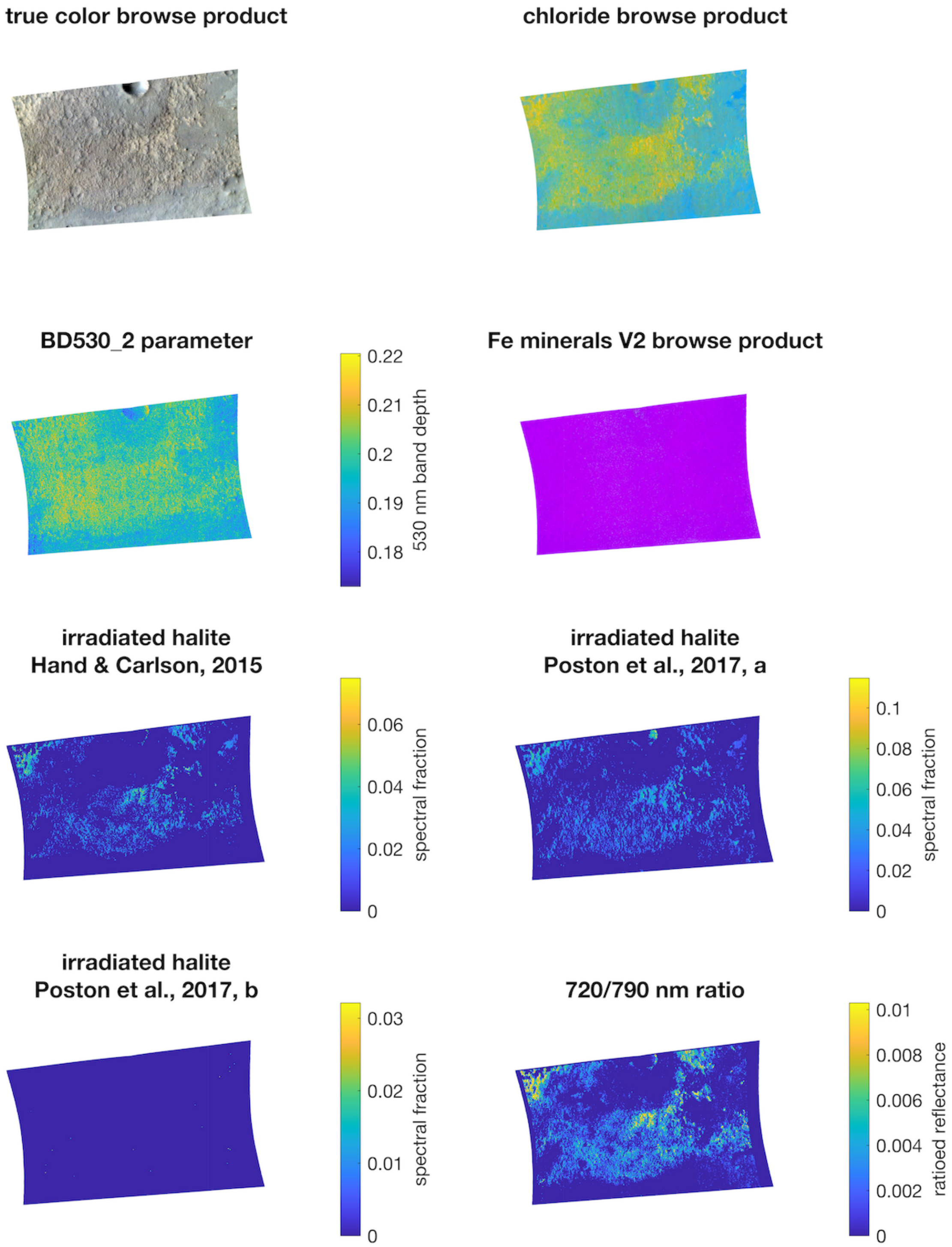

Supplement: Supplementary file 2 — Supplementary Information 2. [file 41598_2024_55979_MOESM2_ESM.pdf]
